# Construction and validation of a hypoxia-related gene signature to predict the prognosis of breast cancer

**DOI:** 10.1186/s12885-024-12182-0

**Published:** 2024-04-01

**Authors:** Chaoran Qiu, Wenjun Wang, Shengshan Xu, Yong Li, Jingtao Zhu, Yiwen Zhang, Chuqian Lei, Weiwen Li, Hongsheng Li, Xiaoping Li

**Affiliations:** 1 Department of Breast, Jiangmen Central Hospital, Jiangmen, Guangdong China; 2 The Sixth Affiliated Hospital of Jinan University(Dongguan Eastern Central Hospital), Dongguan, China; 3 Department of Thoracic Surgery, Jiangmen Central Hospital, Jiangmen, China; 4 Department of Breast Surgery, Foshan Fosun Chancheng Hospital, Foshan, China; 5 Department of Breast Surgery, Guangzhou Medical University Affiliated Cancer Hospital, Guangzhou, China

**Keywords:** Breast Cancer, Prognostic Model, Gene Signature, Hypoxia, Bioinformatics

## Abstract

**Background:**

Among the most common forms of cancer worldwide, breast cancer posed a serious threat to women. Recent research revealed a lack of oxygen, known as hypoxia, was crucial in forming breast cancer. This research aimed to create a robust signature with hypoxia-related genes to predict the prognosis of breast cancer patients. The function of hypoxia genes was further studied through cell line experiments.

**Materials and methods:**

In the bioinformatic part, transcriptome and clinical information of breast cancer were obtained from The Cancer Genome Atlas(TCGA). Hypoxia-related genes were downloaded from the Genecards Platform. Differentially expressed hypoxia-related genes (DEHRGs) were identified. The TCGA filtered data was evenly split, ensuring a 1:1 distribution between the training and testing sets. Prognostic-related DEHRGs were identified through Cox regression. The signature was established through the training set. Then, it was validated using the test set and external validation set GSE131769 from Gene Expression Omnibus (GEO). The nomogram was created by incorporating the signature and clinicopathological characteristics. The predictive value of the nomogram was evaluated by C-index and receiver operating characteristiccurve. Immune microenvironment and mutation burden were also examined. In the experiment part, the function of the two most significant hypoxia-related genes were further explored by cell-line experiments.

**Results:**

In the bioinformatic part, 141 up-regulated and 157 down-regulated DEHRGs were screened out. A prognostic signature was constructed containing nine hypoxia genes (ALOX15B, CA9, CD24, CHEK1, FOXM1, HOTAIR, KCNJ11, NEDD9, PSME2) in the training set. Low-risk patients exhibited a much more favorable prognosis than higher-risk ones (*P* < 0.001). The signature was double-validated in the test set and GSE131769 (*P* = 0.006 and *P* = 0.001). The nomogram showed excellent predictive value with 1-year OS AUC: 0.788, 3-year OS AUC: 0.783, and 5-year OS AUC: 0.817. Patients in the high-risk group had a higher tumor mutation burden when compared to the low-risk group. In the experiment part, the down-regulation of PSME2 inhibited cell growth ability and clone formation capability of breast cancer cells, while the down-regulation of KCNJ11 did not have any functions.

**Conclusion:**

Based on 9 DEHRGs, a reliable signature was established through the bioinformatic method. It could accurately predict the prognosis of breast cancer patients. Cell line experiment indicated that PSME2 played a protective role. Summarily, we provided a new insight to predict the prognosis of breast cancer by hypoxia-related genes.

**Supplementary Information:**

The online version contains supplementary material available at 10.1186/s12885-024-12182-0.

## Introduction

Globally, breast cancer is the most common cancer among women [1–4]. Despite progress in diagnosing and treating breast cancer, approximately 12% of patients ultimately developed tumor metastasis [4]. Of all cancer-related fatalities, breast cancer contributed to 23%, posing a substantial threat to women’s well-being among malignant diseases [5–6].

 Now, the main treatment methods include chemotherapy, targeted therapy, immunotherapy, surgery, endocrine therapy, and radiotherapy [7]. The therapeutic choice depends on clinical-pathological risks and molecular sub-type. However, genetic analysis advancement has offered more precise decision-making to cope with the diversity of breast cancer [8].

Most tumors exhibited hypoxia, which meant an imbalance between oxygen consumption and supply [9–10]. Hypoxia was related to stem cell characteristics, angiogenesis, extracellular matrix organization, protein ubiquitination, immune evasion, and cancer cell metastasis [9–15]. New insights had been gained from recent research on intricate cellular and genomic regulation networks involved in the hypoxic response. This included the epigenetic regulation of transcriptional coregulators, histone, chromatin modifications by hypoxia-inducible factor (HIF), and the expression of various non-coding RNAs [16]. Almost all solid tumors exhibited hypoxia as a typical tumor micro-environmental (TME) feature due to the uncontrolled and rapid proliferation of tumors. Besides, hypoxia, a sign of TME, was essential in drug resistance. Hypoxia-induced drug resistance was closely related to these signaling pathways, including autophagy, drug efflux, and mitochondrial activity [17].

Current studies showed that hypoxia stimulation could initiate epithelial-mesenchymal transition (EMT), which played a key rold in cancer progression [18]. On one hand, EMT was associated with the acquisition of stem cell characteristics from breast cancer cells, which led to drug resistance and poor prognosis [19–20]. On the other hand, EMT was involved in the formation of immunosuppressive microenvironment, resulting the impairment of anti-tumor immunity. For example, EMT destroed the immune synapses of breast cancer cells, which changed the susceptibility of cancer cells to T cell-mediated immune surveillance. It ultimately brought about the weakening of cellular immune function and immune escape [21]. Moreover, Lei Xiang et al. revealed that the expression of Hypoxia-Inducible Factor-2a (HIF-2a) was significantly correlated with higher histology-grade and Ki67 index of breast cancer [22]. As a result, hypoxia might be a hidden prognostic factor for breast cancer.

Previous studies had delineated a prognostic signature in breast cancer using hypoxia-related genes [23–24]. However, these studies had some limitations, including needing more validation and so on. In this research, we aimed to construct a robust signature to predict the outcome of breast cancer patients with hypoxia genes and to explore the cell line function of these genes.

## Materials and methods

### Bioinformatic part

#### Database and DEHRGs

1072 breast cancer samples and 99 adjacent normal tissues were downloaded from The Cancer Genome Atlas (TCGA, https://www.cancer.gov). They were applied for training and testing the signature in a ratio of 1:1. 293 breast cancer cases downloaded from Gene Expression Omnibus (GEO, http://www.ncbi.nlm.nih.gov/geo) were used for external validation. Gene sets related to hypoxia were obtained from the GeneCards website (https://www.gsea-msigdb.org/gsea/index.jsp) with a correlation cutoff value > 1.0. DEHRGs were acquired by comparing tumor and adjacent normal tissues with a cutoff value *p* < 0.05 and|logFC|>1.2. Limma package for R language was employed to analyze DEHRGs. Pheatmap package and ggplot2 package were applied for visualization of DEHRGs.

#### Construction and validation of a signature

Breast cancer patients with complete survival information and follow-up time for at least 30 days were included. The training set and test set were randomly assigned at a 1:1 ratio. Univariate and multivariate Cox regression were utilized to identify prognostic-related DEHRGs by using the equation

$$ RiskScore={\sum }_{1}^{i}\left(coefi*expri\right)$$

(coefi and expi were coefficiency and expressed level for each gene respectively).Calculations were made to determine the risk score of each patient.

Using the median risk score as cut-off value, patients were divided into low-risk and high-risk groups. Statistical analysis was performed on both cohorts using Kaplan-Meier survival curves. The receiver operating characteristic (ROC) was introduced to evaluate the signature’s effectiveness. Subsequently, the test set was used to reconfirm the performance of the signature. “Survival” package, “survminer” package and “timeROC” package for R language were applied.

#### Construction of the nomogram

The expectation of overall survival after 1 year, 3 years, and 5 years were predicted using a nomogram incorporating risk score, age, and TNM stage. To assess the performance of the nomogram, calibration plot, receiver operating characteristic curve, and C index were employed.

#### Functional enrichment analysis

To further investigate the molecular functional processes, Gene Ontology (GO) and Kyoto Encyclopedia of Genes and Genomes (KEGG) were conducted. GO was categorized into biological processes (BP), cellular components (CC), and molecular functions (MF). KEGG was employed to identify the relevant pathways of the genes.

#### Analysis of tumor immune microenvironment and tumor mutation burden

The TME score of every breast cancer patient was calculated by the ESTIMATE algorithm. The proportion of immune cells was quantified with the help of the CIBERSORT algorithm. To acquire information on somatic mutations from breast cancer patients, the tumor mutation burden(TMB) score was analyzed through the “maftools” R package.

### Experimental part

#### Cell culture and transfection

Breast cancer cell lines of human origin, including MDA-MB-231 and MCF7, were acquired from the American Type Culture Collection (Manassas, VA, USA). Cells were incubated at 37 ◦C in a humidified atmosphere with 5% CO_2_ after being cultured in Dulbecco’s modified Eagle medium (DMEM, Gibco, USA) supplemented with 10% fetal bovine serum (FBS, Gibco, USA). Following digestion with trypsin, cells were seeded in 24-well plates at a density of 7 × 10^4 cells per well. RiboBio (Guangzhou, China) synthesized PSME-shRNA and KCNJ11-shRNA (sh-PSME and sh-KCNJ11) and NC (sh-NC) in vivo experiment, with a concentration of 10^4 cells/500 mL per well. Lentiviruses were used to infect MDA-MB-231 and MCF7 cells. All shRNAs and negative control were transfected into cells using Lipofectamine 2000 according to the manufacturer’s instructions.

#### Western blots

The cells were broken down in ice-cold RIPA buffer with PMSF, cocktail, and phosphatase inhibitor, followed by bicinchoninic acid (BCA) assay for protein concentration quantitation. Polyvinylidene difluoride membranes received electro-transferred sodium dodecyl sulfate-polyacrylamide (SDS) gel electrophoresis of 20 µg protein lysates. Membranes were blocked with 5% skimmed milk for 30 min at room temperature. Then, specific primary antibodies, such as CDK2 (CST, #2546), CDK4 (CST, #12,790), CDK6 (CST, #3136), P21 (CST, #2947), CyclinD3 (CST, #2936),Bcl-2 (Proteintech, 12789-1-AP), Bax (Proteintech, 50599-2-Ig), and GAPDH (CST, #2118) (1:1,000 and 1:2,000 in 1% BSA/TBS-T), were then added and incubated overnight at 4℃. Incubation of secondary antibodies was carried out for a duration of 1 hour at room temperature. Finally, the enhanced chemiluminescence (ECL) reagent was used to visualize the protein bands. CDK2/4/6 P21 and CyclinD3 are cell cycle-related proteins, which regulate cell proliferation. Bcl and Bax are apoptosis-related proteins. Cell proliferation and apoptosis are assessed by detecting those proteins’ levels. To analyze the western blot results, the bands were assessed with the Quantity One 1-D Analysis Software (Bio-Rad, Hercules, CA).Targeting protein expression levels were measured in the same sample and compared to ACTB/GAPDH levels, then normalized to a control group set at 1.

#### Cell counting kit-8 (CCK8) analysis

CCK8 assay was conducted following the manufacturer’s instructions (meilunbio, MA0218). In summary, 96-well plates were seeded with 2000 cells, and 10 µl/well of CCK-8 reagent was added on day-1 -2, -3, -4, and − 5. Following the inclusion of CCK-8 solution, the cells were left to incubate for an additional 2 hours at a temperature of 37 °C, and the optical density (OD) was gauged at 450 nm. CCK8 analysis was used to evaluate the growth and proliferation ability of cells.

#### Colony formation

A total of one thousand cells were placed in a 12-well dish and grown for a period of two weeks. Then the cells were first treated with 4% paraformaldehyde and then stained with 0.5% crystal violet for an hour. The number of colonies was counted for analysis. Colony formation was used to evaluate the proliferation ability of cells.

#### 5-Ethynyl-2’-deoxyuridine (EdU) staining

Following the guidelines provided by Beyotime (C0078S), the EdU test was performed. Briefly, cells were seeded on the glass slide. Cells were labeled with 10 µM EdU reagent for 1 hour at 37 °C when cells reached 80% confluence. Next, the cells were rinsed using PBS and treated with 4% paraformaldehyde for 20 min at room temperature. After that, the nuclei were stained with DAPI. To visualize the count of cells positive for EdU, Nikon Ti2 was used to capture images. Edu staining was used to detect the proliferation rate of cells.

#### Wound healing assay

Cells were seeded into 12-well plates and cultured with a complete medium. When cells reached 80% confluence, straight wounds were made by using 10-µl pipette tips and then cultured cells in a medium with 1% FBS. The wound gaps were photographed at regular intervals (0, 24, and 48 h) using Nikon Ti2 microscope. Wound healing assay was used to assess the ability of cell migration and repair ability.

#### Statistical analysis

Continuous variables conforming a normal distribution were expressed as mean ± standard deviation (SD). The difference between groups was evaluated using Student’s t-test or one-way ANOVAs. Continuous variables that did not conform a normal distribution were represented as median and interquartile range (IQR). Wilcoxon test was used to compare the difference of the groups. Categorical variables were presented as frequency and proportion [n (%)] and χ^2^ test or fisher exact test was used to identify the groups difference. The overall survival (OS) was estimated using Kaplan-Meier. The log-rank test was used to compare the median survival time between groups. Cox proportional hazard model was established to identify independent prognostic factors for OS. The prognostic value was assessed using the operating characteristic curve (ROC) and area under the curve (AUC) was calculated. All the statistical analysis was performed by GraphPad Prism V6.0 (GraphPad Software, Inc., La Jolla, CA, USA) and R language. The experiments were repeated thrice independently. A two-sided *P* < 0.05 was considered statistically significant.

## Results

In this study, we tried to make use of TCGA and the Genecards platform to identify prognostic-related hypoxia genes for breast cancer. Then the predictive signature was established and doubled validated based on these hypoxia genes. Finally, the cellular function of the two genes (KCNJ11 and PSME2) were explored through in vitro experiment.

### Database and DEHRGs

**Fig. 1:**
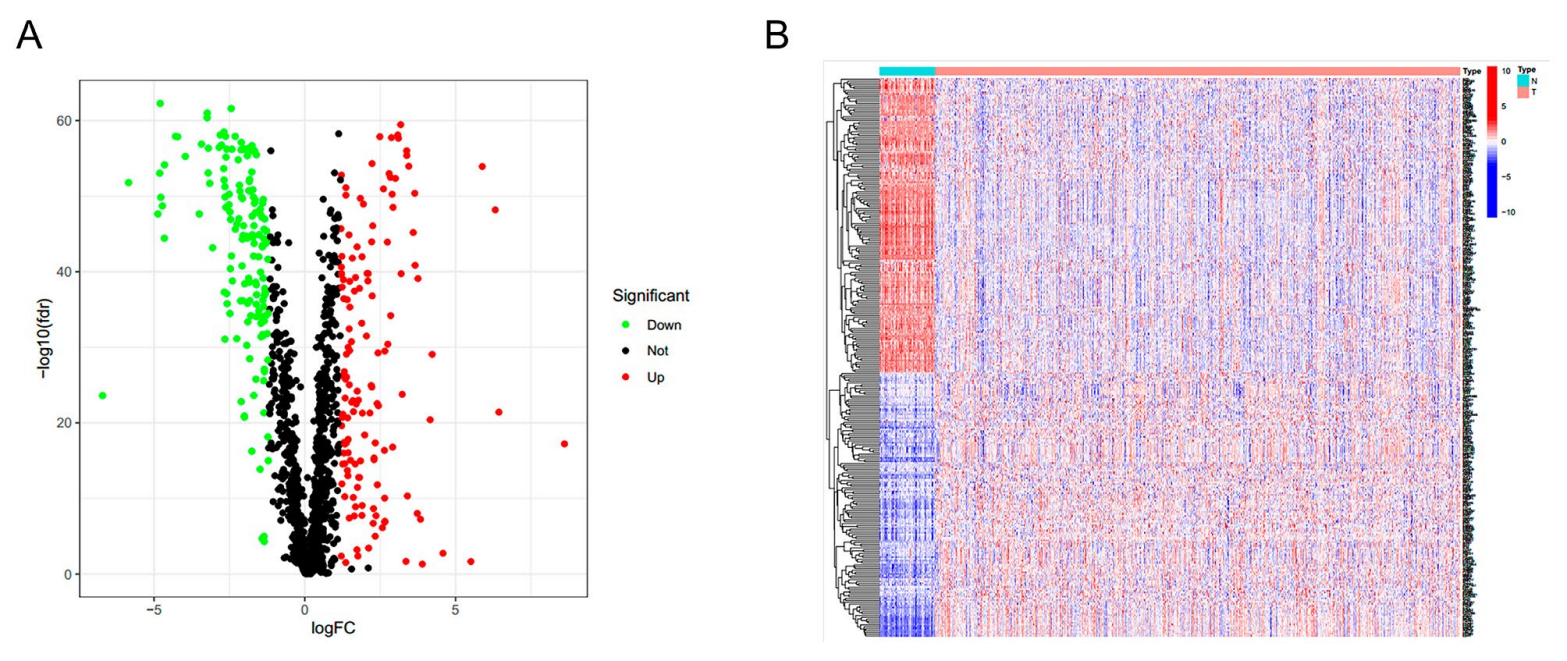
Differential expression of hypoxia-related genes in tumor and adjacent normal tissues. **(A)** The volcano map showed the up-regulated and down-regulated DEGs (*P* < 0.05). **(B)** The heatmap showed 298 DEGs expressed in tumor and adjacent normal tissues

1072 breast cancer samples and 99 adjacent normal tissues were retrieved from TCGA. 1607 hypoxia-related genes were acquired from GeneCards, with a correlation cutoff value>1.0 (Supplementary Table 1). 141 up-regulated and 157 down-regulated DEHRGs were screened out through analyzing the RNA-Seq data of the tumor and adjacent normal tissue. (Figure 1A and B, and Supplementary Table 2).

### Construction and validation of the risk-score model

After excluding samples with incomplete clinical and follow-up information, 861 breast cancer cases were including in model construction. They were divided into training set and test set in a 1:1 ratio. As a result, the training set contained 432 samples and the test set included 429 samples (Supplementary Tables 3 and Table 4). In the training set, nine prognosis-related hypoxia genes were screened out, including six tumor suppressor genes (ALOX15B, CA9, FOXM1, KCNJ11, NEDD9, and PSME2) [Hazard ratio(HR)<1] and three oncogenic genes (CD24, CHEK1, and HOTAIR) [Hazard ratio(HR)>1] (Supplementary Table 5). Then the prognostic signature was established. The risk score of every breast cancer patient was calculated according to the formula.

$$ \left( \begin{gathered} Risk{\text{ }}score = 0.124*CD24 + 0.683*CHEK1 + \hfill \\\,\,\,\,\,\,\,\,\,\,\,\,\,\,\,\,\,\,\,\,\,\,\,\,\,\,\,\,\,\,\,0.260*HOTAIR - 0.227*ALOX15B - \hfill \\\,\,\,\,\,\,\,\,\,\,\,\,\,\,\,\,\,\,\,\,\,\,\,\,\,\,\,\,\,\,\,\,\,0.137*CA9 - 0.331*FOXM1 - 0.236* \hfill \\\,\,\,\,\,\,\,\,\,\,\,\,\,\,\,\,\,\,\,\,\,\,\,\,\,\,\,\,\,KCNJ11 - 0.539*NEDD9 - 0.387*PSM2 \hfill \\ \end{gathered} \right)$$

**Fig. 2:**
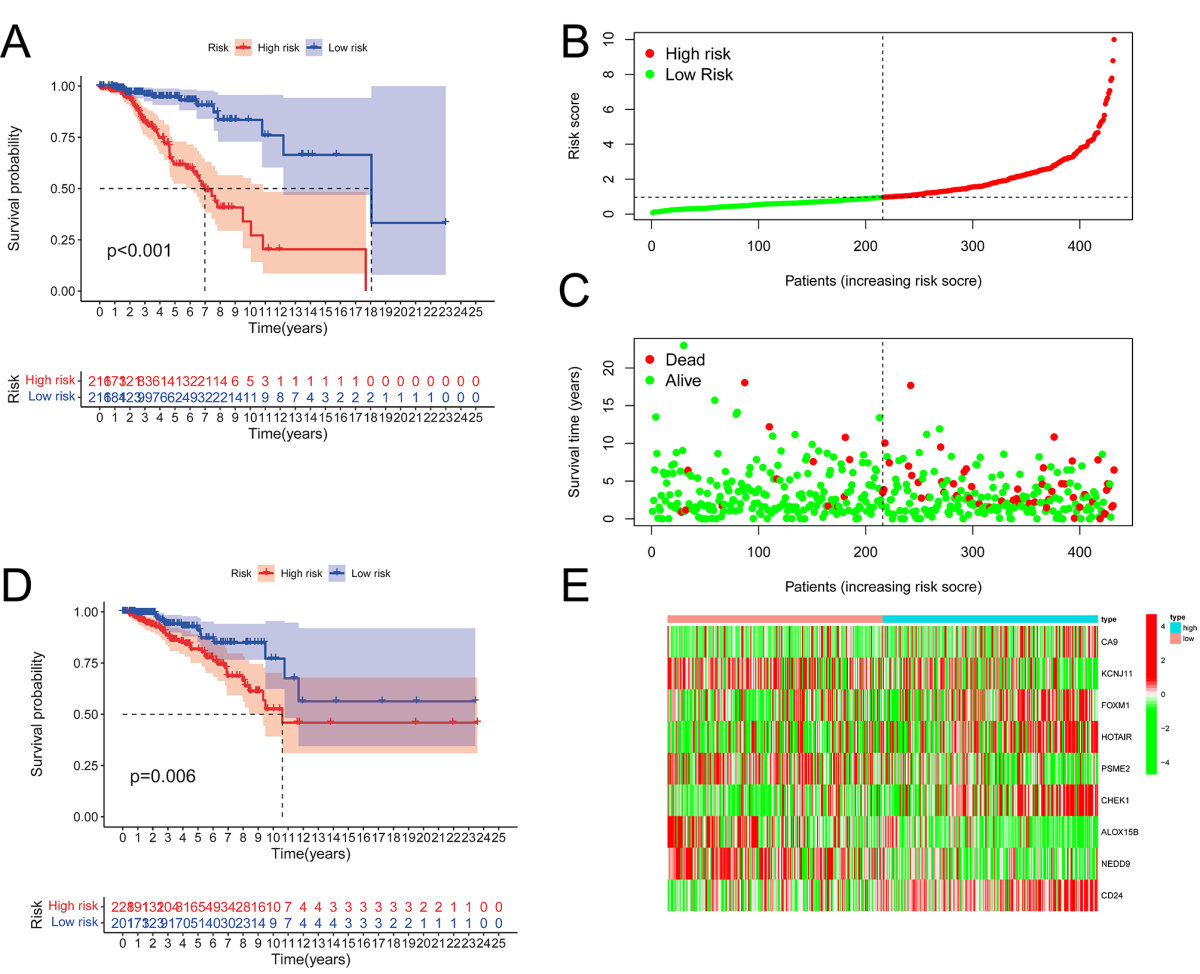
Construction of the prognostic signature. **(A)** Kaplan–Meier Survival curves of the high-risk and low-risk groups in the training set (*P* < 0.001). **(B, C)** The distributions of the risk score and survival status in the training set. **(D)** Kaplan–Meier survival curves in the validation set. **(E)** Heatmap demonstrated the expression of nine prognosis-related hypoxia genes in the two groups

Based on the median values; the patients were divided into high risk and low risk groups based on the level of risk score. Principal component analysis (PCA) and t-distributed stochastic neighbor embedding (t-SNE) showed that the distribution of the two group was different (Figure A1A, A1B). The low-risk group demonstrated a more favorable prognosis than the high-risk group with *P* < 0.001 (Fig. 2A). As the patient’s risk score rises, the likelihood of mortality also increases(Fig. 2B and C). Heatmaps were applied to illustrate the representative of these 9 genes in high-risk and low-risk patients (Fig. 2E). The signature was double-verified in the test set and GSE131769, which implied the robust prediction value (*P* = 0.006 and *P* = 0.001, Fig. 2D and Supplementary Fig. 1).

### Independent prognostic factors and nomogram establishment

**Fig. 3:**
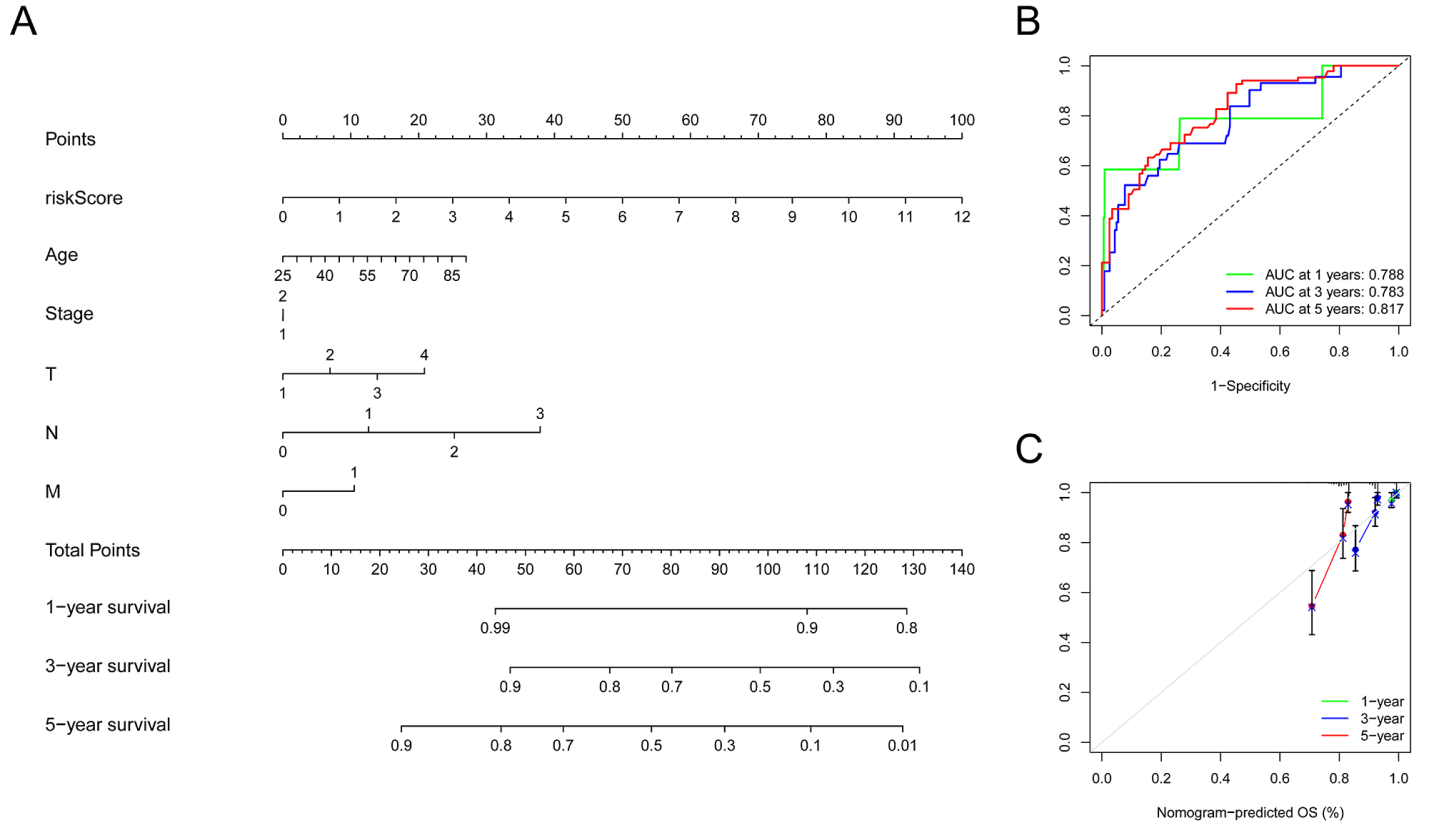
Establishment and evaluation of the nomogram. **(A)** The nomogram was applied to predict the OS for breast cancer patients. **(B)** ROC curve analysis was used to predict accuracy for 1-year, 3-year, and 5-year OS. **(C)** Calibration curves for 1-year, 3-year, and 5-year OS

Through univariate and multivariate COX regression, age and risk-score signatures (the nine hypoxia-related-gene signature) were considered independent prognostic factors (Figure A2A, A2B). A nomogram incorporating age, TNM stage and the signature was created to predict 1 year, 3-year and 5-year survival for breast cancer patients (Fig. 3A). The ROC curve was applied to evaluate the prediction performance of the nomogram. It showed that 1-year-OS AUC was 0.788, 3-year-OS AUC was 0.783, and 5-year-OS AUC was 0.817, which implied the excellent predictive performance of the nomogram (Fig. 3B). Through calibration curve, it was found that 1-year nomogram predicted OS showed the best reliability for model prediction (Fig. 3C).

### Functional enrichment and tumor immune microenvironment analysis

In order to interpret gene products, functional characteristics, and potential signaling pathways, we conduct the analysis of GO, KEGG, and Gene Set Enrichment Analysis (GSEA). TME is closely related to the prognosis and treatment response of patients. So, we analyze infiltrating immune cells, TME score, and TMB score of TME.

In biological process, “organelle fission” and “nuclear division” ranked the top two position. While in the CC category, the DEHRGs were enriched in the “chromosomal region” and “condensed chromosome”. “Microtubule binding” and “catalytic activity” were the main function in the MF category (Supplementary Fig. 2A). KEGG analysis showed “cell cycle” and “cellular senescence” were the most critical pathways [25] (Supplementary Fig. 2B). GSEA analysis revealed the top 5 significant pathways in high-risk and low-risk groups (Supplementary Fig. 2C, 2D).

In the high-risk group, the expression of naive B cells, plasma cells, resting memory CD4 T cells, and resting mast cells were found to be higher than the low-risk group in the train set. While follicular helper T cells and M0 macrophages were lower (Figure A3A).

In the test set, M0 macrophages were significantly lower in the low-risk group; while resting mast cells showed the opposite situation (Figure A3B). By calculateing the score through ESTIMATE algorithm, the low-risk group exhibited higher stromal scores, immune scores, and ESTIMATE scores (*P* < 0.01) (Figure A4A-C). The high-risk group had a significantly higher burden of tumor mutations (Figure A4D). Limma package and estimate package for R language were employed for statistics.

### Knockdown of PSME2 represses cell growth and clone formation in MDA-MB-231 cells and MCF-7 cells

**Fig. 4:**
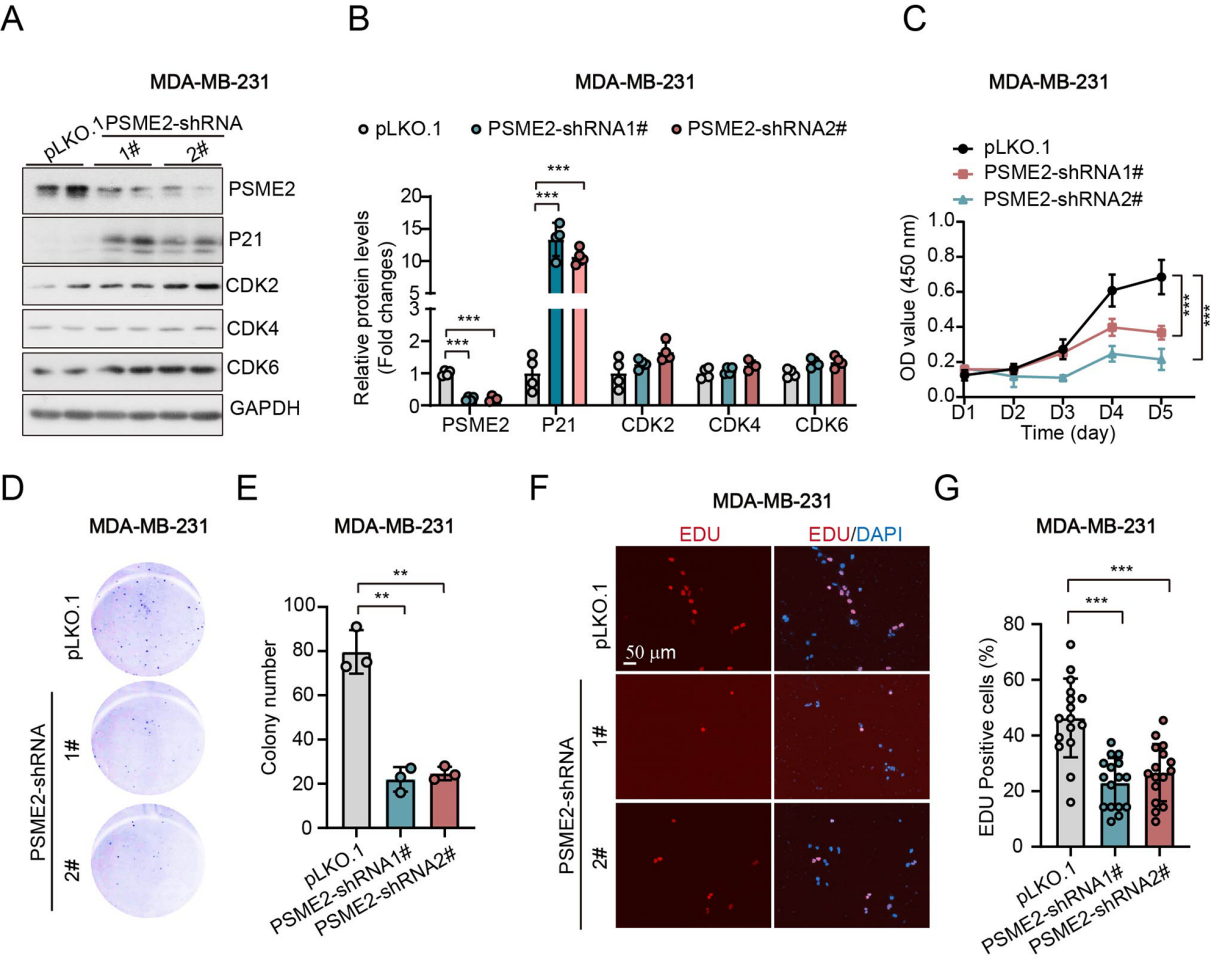
Knockdown of PSME2 represses cell growth and clone formation in MDA-MB-231 cells. **(A-B)** Western blots and quantitative results of PSME2 and the indicated proteins in MCF-7 cells. **(C)** Cell viability measurement by CCK8. **(D-E)** Images and quantitative results of cell clone. **(F-G)** Images of EDU staining and the quantitative results. pLKO.1: the normal control cells; PSME-shRNA1/2: two PSME2 knockdown cell lines. (**: *p* < 0.01; ***: *p* < 0.001). (Western blot results in Fig. 4A were cropped from the raw data in Supplementary Info File)

Among these nine screened genes, most of them were well-studied in breast cancer, except PSME2 and KCNJ11. To investigate whether PSME2 and KCNJ11 played roles in breast cancer development. Here, we knock-downed of PSME2 and KCNJ11 in two breast cancer cell lines, MDA-MB-231 and MCF-7 cells. In MDA-MB-231 cells, knockdown of PSME2 significantly promoted the of expression of cell cycle inhibitor P21 (Fig. 4A-B). While, CDKs (cylcine-dependent kinase), the key regulators of cell cycle, such as, CDK2, CDK4 and CDK6 had little changes after knockdown of PSME2 (Fig. 4A-B). The CCK8 assay showed that knockdown of PSME2 repressed the cell viability of MDA-MB-231 cells (Fig. 4C). Consistently, the clone assay also showed that deficiency of PSME2 suppressed the clone formation (Fig. 4D-E). Thus, we wondered whether PSME2 regulated the cell proliferation of MDA-MB-231 cells. EDU staining was used to measurement the cell proliferation. We found that knockdown of PSME2 significantly reduced the EDU positive cells (Fig. 4F-G), indicating that knockdown of PSME2 repressed cell proliferation in MDA-MB-231 cells.

**Fig. 5:**
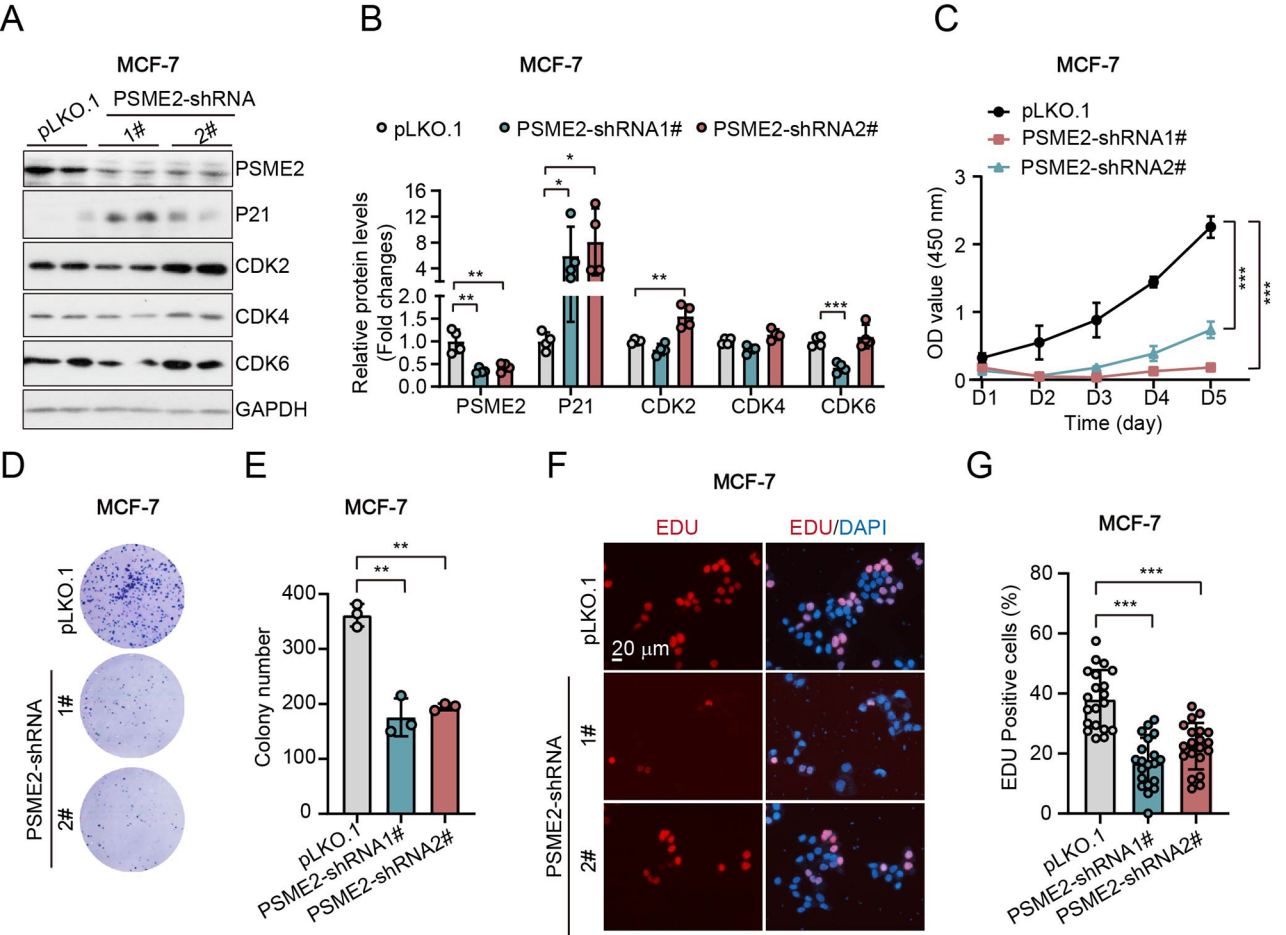
Knockdown of PSME2 represses cell growth and clone formation in MCF-7 cells. **(A-B)** Western blots and quantitative results of PSME2 and the indicated proteins in MCF-7 cells. **(C)** Cell viability measurement by CCK8. **(D-E)** Images and quantitative results of cell clones. **(F-G)** Images of EDU staining and the quantitative results. pLKO.1: the normal control cells; PSME-shRNA1/2: two PSME2 knockdown cell lines. (**: *p* < 0.01; ***: *p* < 0.001). (Western blot results in Fig. 5A were cropped from the raw data in Supplementary Info File)

To further conform that deficiency of PSME2 repressed breast cancer proliferation, we used another breast cancer cell line, MCF-7. Consistent with MDA-MB-231, knockdown of PSME2 also significantly increased the expression of P21 (Fig. 1A-B). Compared to the control group (pLKO.1), the expression levels of CDK2, CDK4, and CDK6 were reduced in PSME2-shRNA-1# (Fig. 5A-B). While the expression of CDK4 and CDK6 did not change in PSME2-shRNA-2#, only the CDK2 showed a mild increase (Fig. 5A-B). We also found that the knockdown of PSME2 significantly repressed cell viability and clone formation in MCF-7 (Fig. 5C-E). Similarly, the EDU staining results demonstrated that the knockdown of PSME2 suppressed the cell proliferation of MCF-7 (Fig. 5F-G). Together, these data indicated that the knockdown of PSME2 repressed cell proliferation and clone formation in breast cancer cell lines.

### Knockdown of KCNJ11 does not affect cell growth and migration in MCF-7 cells

**Fig. 6:**
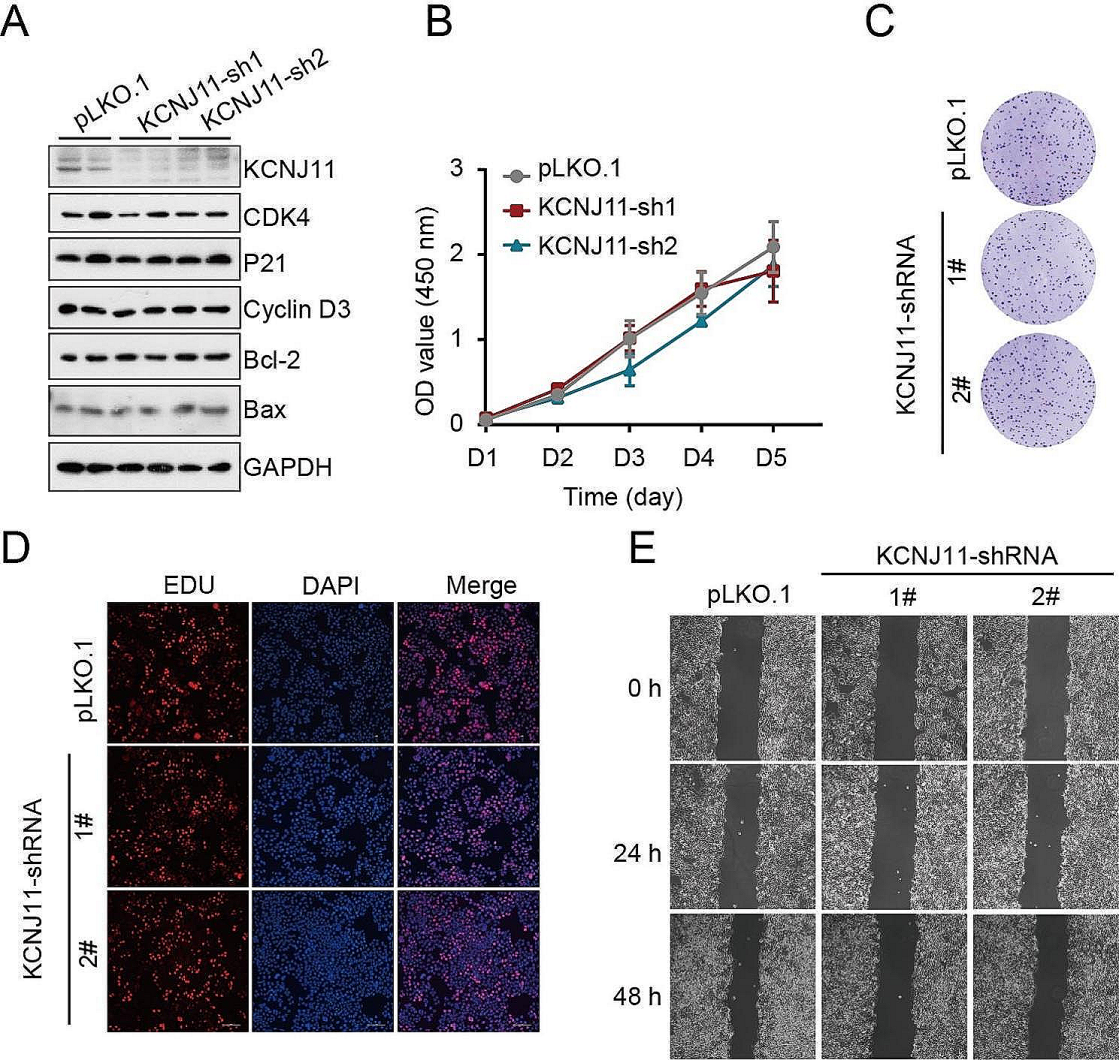
Knockdown of KCNJ11 does not affect cell growth and migration in MCF-7 cells. **(A)** Western blots of KCNJ11 and the indicated proteins in MCF-7 cells. **(B)** Cell viability measurement by CCK8. **(C-D)** Images of cell clones and EDU staining. **(E)** Images of wound healing assay to evaluate migration rate at 24 and 48 h. pLKO.1: the normal control cells; KCNJ11-sh1/2: KCNJ11 knockdown cells used KCNJ11 shRNA1 and KCNJ11-shRNA2

Next, to investigate whether KCNJ11 influenced the cell viability, clone formation, or migration of breast cancer cells, KCNJ11 was knockdown in MCF-7. It was found that the knockdown of KCNJ11 had no effects on the expression of cell cycle proteins (CDK4, P21, and Cyclin D3), as well as apoptosis proteins (Bcl-2 and Bax) (Fig. 6A). The cell growth cure showed no difference between the control group (pLKO.1) and KCNJ11knockdown groups (KCNJ11-sh1 and KCNJ11-sh2) (Fig. 6B), indicating that knockdown of KCNJ11 had no roles on the cell viability of MCF-7. The clone formation and EDU staining results showed that the knockdown of KCNJ11 did not affect the clone formation and proliferation of MCF-7 (Fig. 6C-D). Additionally, we performed the wound healing assay and found that the knockdown of KCNJ11 also did not affect the rate of wound healing (Fig. 6E); it appeared that KCNJ11 did not regulate MCF-7 migration. The data presented here indicated that KCNJ11 played no role in the breast cancer cell’s growth and migrate.

## Discussion

Our research successfully established a nine hypoxia-related gene signature through TCGA database. The robust of the signature was validated by external dataset GSE131769. Through in vitro experiment, we found PSME2 played an anti-tumor role in breast cancer.

The incidence of breast cancer has increased dramatically in the past decade [26]. As a marker of TME, hypoxia was involved in various aspects of tumor progression [9, 27–28]. Moreover, hypoxia was associated with drug resistance in the treatment of breast cancer [29–30]. According to our findings, hypoxia-related genes might be useful as biomarkers in predicting the long-term prognosis of breast cancer patients. Some scientists have tried to create risk models to elucidate the outcome of breast cancer patients [31–33]. Nonetheless, these models either lacked clinical validation or experimental validation. As a result, a robust signature was urgently needed.

This study constructed a hypoxia-related signature containing nine genes in the TCGA training set. This signature could distinguish the outcome of high-risk and low-risk patients. It revealed excellent performance in the training set. The robustness of the model was double-validated by the test and external independent validation set (GSE131769).

To explore the function of DEGs. GO enrichment analysis was utilized. As a result, the DEGs were mainly involved in the nuclear division, chromosomal region, and division organelle fission, providing further insight into the main underlying mechanisms. KEGG pathway analysis indicated that the most significant pathway was the cell cycle and cellular senescence. According to Druker’s research, hypoxia played an essential role on the cell cycle at the transcriptome level [34]. Protein synthesis could also be modified in the hypoxia environment [34]. Thus, hypoxia-induced changes in the cell cycle were influenced by several factors.

Among the nine prognostic hypoxia genes, most of them were *widely reported in previous studies, except KCNJ11 and PSME2.* CD24, CHEK1, and HOTAIR were possibly oncogenic genes. Cluster of differentiation 24 (CD24) was a glycosyl-phosphatidyl-inositol-anchored glycoprotein [35]. Barkal et al. demonstrated that a role for tumor-expressed CD24 in promoting immune evasion through its interaction with the inhibitory receptor sialic-acid-binding Ig-like lectin 10 [36]. In a meta-analysis, Wang et al. revealed that the putative stem cell marker CD24 was significantly associated with worse survival based on 5697 BC cases [37]. In conclusion, the negative role of CD24 in breast cancer was evident.

CHEK1 was a checkpoint kinase. Previous studies reported that CHEK1 played a critical role in maintaining genomic stability and preventing the accumulation of DNA damage during cell division [38]. Xu et al. found that CHK1 inhibition would enhance adriamycin (ADR) chemosensitivity [39]. In addition, lncRNA HOTAIR was engaged in cellular metastasis in various cancers, such as colorectal cancer, hepatocellular cancer, and non-small-cell lung cancer [40–42]. Notably, HOTAIR could serve as a molecular sponge for miR-20a-5p, promoting breast cancer progression and tumorigenesis by activating the expression of the HMGA2 protein [43]. As a result, CHEK1 and HOTAIR also served as oncogenic roles in breast cancer.

ALOX15B, CA9, FOXM1, KCNJ11, NEDD9, and PSME2 might be protective factors in the hypoxia signature. Evidence has shown that carbonic anhydrase 9 (CA9), a glycoprotein of the zinc-containing enzyme family, was an inducible expressed gene in response to hypoxia in cancers [44–45]. The exterior cellular acidity of CA9 has a supportive effect on carcinoma cells [46].

For FOXM1 (forkhead box protein M1), previous research articles showed it played a fundamental role in tumorigenesis, which was mainly related to the regulation of cell cycle progression [47–48]. For NEDD9, Hu et al. discovered histone deacetylase inhibitors promoted breast cancer metastasis by elevating NEDD9 expression [49]. Also, NEDD9 over-expression caused hyper-proliferation of luminal cells and cooperated with the HER2 oncogene in tumor initiation [50]. So, both CA9 and FOXM1 were novel markers of poor prognosis for breast cancer patients.

Proteasome activator subunit 2 (PSME2) was a protein involved in the regulation of the proteasome [51–52]. According to prior research, PSME2 served as an indicator of the metastasis of tumors [53]. In breast cancer, PSME2 had the potential to identify immune hot tumors and predict the response to immunotherapy [54]. But the function of PSME2 in breast cancer cell lines was still unclear. When searching in Pubmed, the research about KCNJ11 and breast cancer was rare. To further investigate the function of PSME2 and KCNJ11 in breast cancer, cell line experiment was performed. MDA-MB-231 and MCF7 breast cancer cell lines were introduced. Our study demonstrated that reducing PSME2 expression with siRNA could inhibited cell viability, weakened colony formation, and suppressed invasion in BC cells. The cell line experiment further validated the tumor suppression role of PSME2. Nonetheless, the decrease in KCNJ11 expression had no notable impact on the survival of the cells. Multiple factors influenced the result. Cell experiments could not fully reflect the situation of KCNJ11 in the human, as the human body was a sophisticated system. The formation and progression of cancer was a complicated procedure that involved numerous genes and environmental factors. Also, the alterations in cancer cells were not solely caused by a single gene variation.

By analyzing the prognostic-related hypoxia genes mentioned above, it was found that these genes could affect tumor behavior in terms of causing changes and regulating immune response in tumor microenvironment, which would lead to drug resistance of tumor cells. Thus, the expression of hypoxia-related genes could predict the prognosis of breast cancer patients. Similarly, Jianxin wang et al. also successfully constructed a diagnostic signature with hypoxia related genes in the TCGA and GEO databases [55]. However, they did not confirm their results or elucidate the molecular mechanisms through cell experiments. Interestingly, they also found that the HIF-1 signaling pathway was involved in the activation of cancer stem cells during tumor occurrence and development. Currently, more and more research tried to target the cellular response to hypoxia in human cancers. It appeared that inhibiting HIF-1 alpha activation was the primary approach and might enhance chemotherapy response. Xia Yang et al [56] developed a signature by combining hypoxa and immune genes to predict prognosis of breast cancer. But they just focused on triple-negative breast cancer subtype. Also this research lacked of wet experiment.

There were some limitations of this study. Firstly, some important clinical factors, such as chemotherapy regime and duration of endocrine therapy were missing, which would lower the accuracy of the signature. Chemotherapy and endocrine therapy regimens were important factors affecting the therapeutic efficacy of patients. Secondly, the signature was established based on retrospective data. The fundamental flaw of retrospective data was the existence of various biases. These bias may led to a decrease in the authority of the model. A prospective study will be needed to verify the model. Thirdly, further exploration of the function of hypoxia genes in vivo is needed. Animal experiment could better simulate the internal environment of human body. Nonetheless, our research successfully developed a robust prognostic signature. In clinical practice, oncologist would face a dilemma whether the patient should offer intensive treatment. With the help of this signature to predict the risk of recurrence, oncologist could offer personalized treatment strategies according to the risk score. What’s more, PSME2 would be an indicator of good prognosis for breast cancer patients. Through further research, PSME2 may develop a kit to identify breast cancer patients with good prognosis and avoid over-treatment in the future.

## Conclusion

To conclude, we have successfully developed a risk-score signature based on nine hypoxia-related genes to predict the prognosis of breast cancer patients. The robustness of the signature was double-validated. Cell line experiment demonstrated that PSME2 played a protective role in breast cancer. We hoped this study would provide useful knowledge for improving the accuracy of predicting the prognosis of breast cancer patients.

### Electronic supplementary material

Below is the link to the electronic supplementary material.

Supplementary Material 1. All the other data supporting the findings of this study are available within the Article and its Supplementary Information files. A Reporting Summary is available as a Supplementary Information file.

Supplementary Material 2

Supplementary Material 3

## Data Availability

The data could be downloaded at (https://www.cancer.gov; http://www.ncbi.nlm.nih.gov/geo; https://www.genecards.org) and the code used during the current study is available from the corresponding author on reasonable request.
