# Supplementary material for: Construction and validation of a hypoxia-related gene signature to predict the prognosis of breast cancer
Source: BMC Cancer. 2024 Apr 1;24:402. doi: 10.1186/s12885-024-12182-0 (PMC10986118; doi:10.1186/s12885-024-12182-0)
Supplement: Supplementary file 1 — Supplementary Material 1. All the other data supporting the findings of this study are available within the Article and its Supplementary Information files. A Reporting Summary is available as a Supplementary Information file. [file 12885_2024_12182_MOESM1_ESM.docx]

**Appendix A Figure**

**
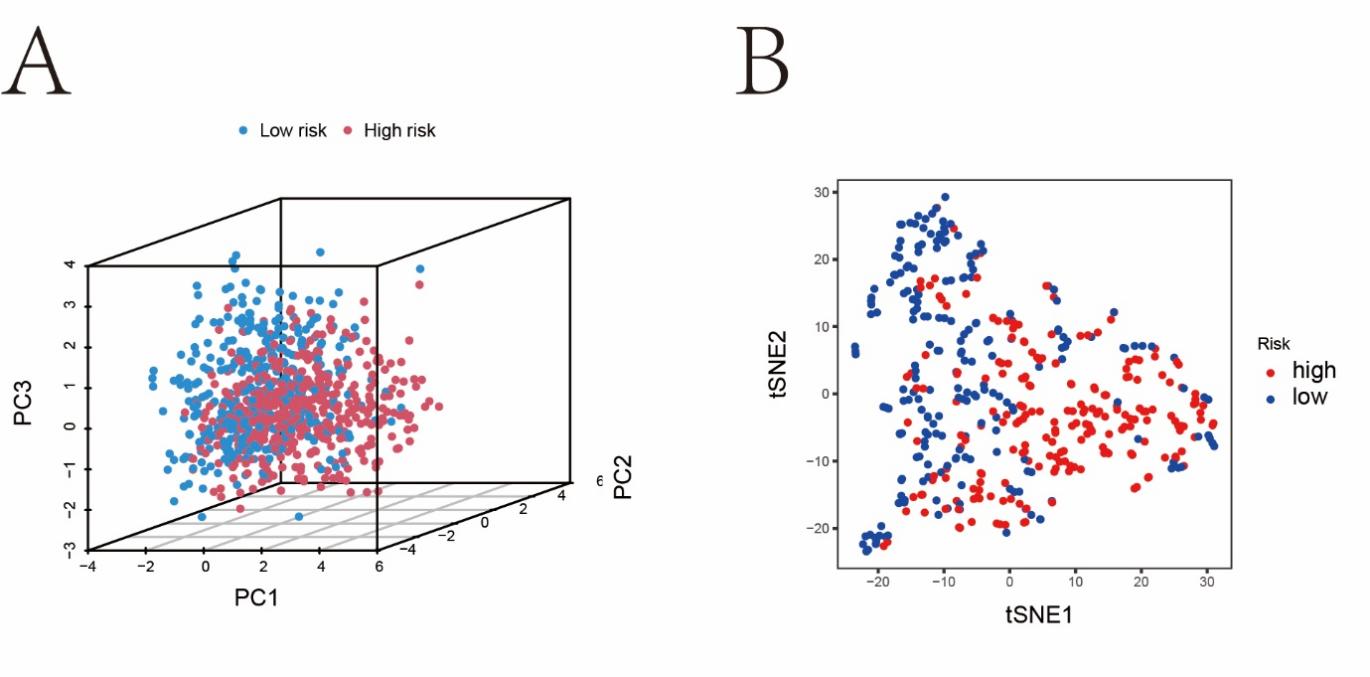
**

**Figure A1** | Sample distribution according to the risk score. (A, B) PCA and t-SNE based on the expression profiles of the 9 prognostic hypoxia genes separated by high-risk and low-risk groups.

**
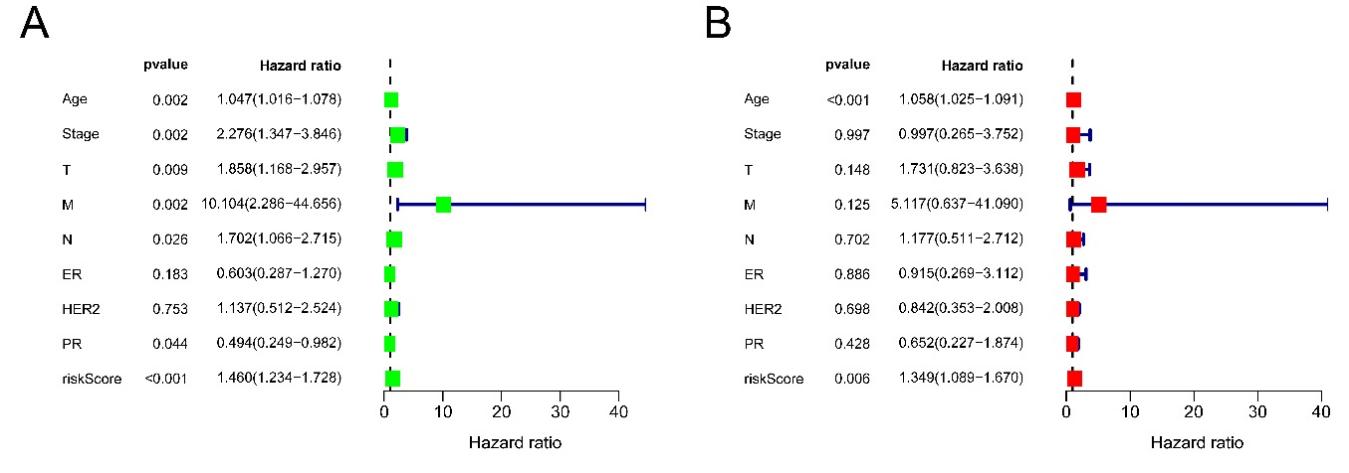
**

**Figure A2** | Exploration of the independent risk factor for breast IDC patients. (A) the risk-score signature and clinical features (age, stage) were statistical in Univariate Cox regression. (B) Multivariate Cox regression found that signature and age were independent risk factors.

**
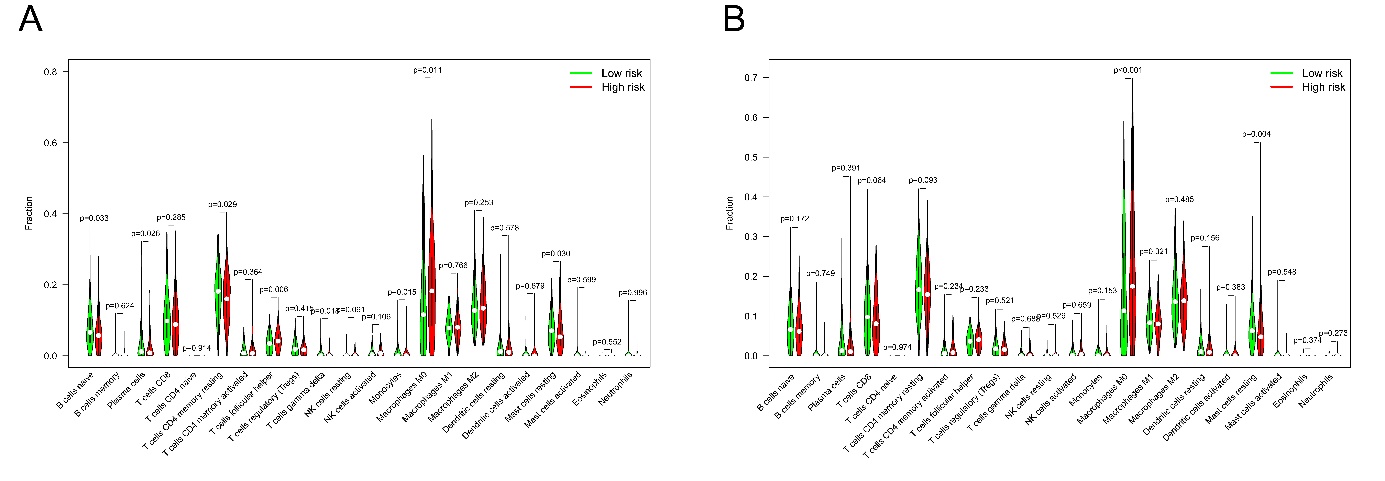
**

**Figure A3** | Relationship between the risk score and infiltrating immune cells (A-TCGA training set; B-TCGA validation dataset). Violin plots represented the expression of infiltrating immune cells between the two groups.

**
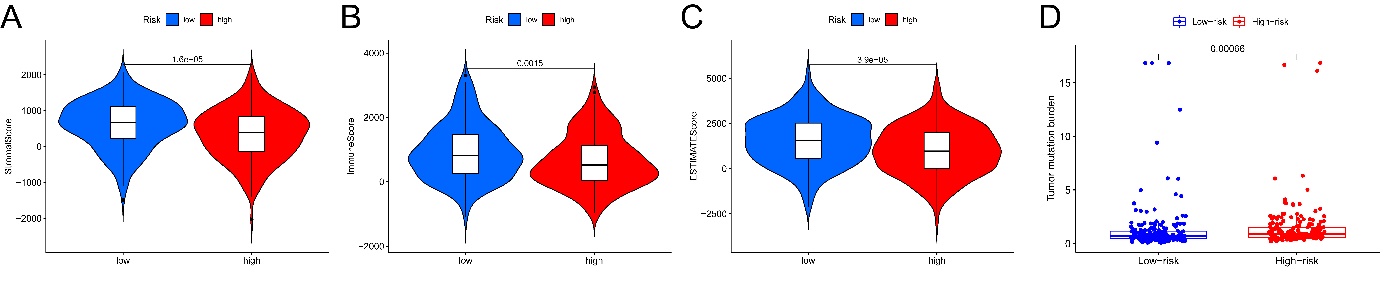
**

**Figure A4** | The stromal and immune scores were calculated, and TMB status was evaluated. (A) Stromal score. (B) Immune score. (C) ESTIMATE score. (D) The box plot reflects the correlation between the risk score and the TMB score.
