# Supplementary material for: Construction and validation of a hypoxia-related gene signature to predict the prognosis of breast cancer
Source: BMC Cancer. 2024 Apr 1;24:402. doi: 10.1186/s12885-024-12182-0 (PMC10986118; doi:10.1186/s12885-024-12182-0)
Supplement: Supplementary file 3 — Supplementary Material 3 [file 12885_2024_12182_MOESM3_ESM.docx]

Supplementary table 1. Hypoxia-related genes obtained from GeneCards with correlation > 1.0

| Gene Symbol | Description | Category | Gifts | GC Id | Relevance score | GeneCards Link |
| --- | --- | --- | --- | --- | --- | --- |
| HIF1A | Hypoxia Inducible Factor 1 Subunit Alpha | Protein Coding | 45 | GC14P061695 | 75.59578 | https://www.genecards.org/cgi-bin/carddisp.pl?gene=HIF1A |
| EGLN1 | Egl-9 Family Hypoxia Inducible Factor 1 | Protein Coding | 47 | GC01M231363 | 33.29502 | https://www.genecards.org/cgi-bin/carddisp.pl?gene=EGLN1 |
| HIF3A | Hypoxia Inducible Factor 3 Subunit Alpha | Protein Coding | 38 | GC19P046297 | 31.10758 | https://www.genecards.org/cgi-bin/carddisp.pl?gene=HIF3A |
| HIF1AN | Hypoxia Inducible Factor 1 Subunit Alpha Inhibitor | Protein Coding | 43 | GC10P100529 | 29.76139 | https://www.genecards.org/cgi-bin/carddisp.pl?gene=HIF1AN |
| EGLN3 | Egl-9 Family Hypoxia Inducible Factor 3 | Protein Coding | 44 | GC14M033924 | 29.00042 | https://www.genecards.org/cgi-bin/carddisp.pl?gene=EGLN3 |
| EGLN2 | Egl-9 Family Hypoxia Inducible Factor 2 | Protein Coding | 43 | GC19P045026 | 28.16361 | https://www.genecards.org/cgi-bin/carddisp.pl?gene=EGLN2 |
| VEGFA | Vascular Endothelial Growth Factor A | Protein Coding | 46 | GC06P043770 | 25.94184 | https://www.genecards.org/cgi-bin/carddisp.pl?gene=VEGFA |
| EPAS1 | Endothelial PAS Domain Protein 1 | Protein Coding | 47 | GC02P046293 | 24.85666 | https://www.genecards.org/cgi-bin/carddisp.pl?gene=EPAS1 |
| HYOU1 | Hypoxia Up-Regulated 1 | Protein Coding | 42 | GC11M119121 | 24.16202 | https://www.genecards.org/cgi-bin/carddisp.pl?gene=HYOU1 |
| VHL | Von Hippel-Lindau Tumor Suppressor | Protein Coding | 44 | GC03P011651 | 23.87787 | https://www.genecards.org/cgi-bin/carddisp.pl?gene=VHL |
| HILPDA | Hypoxia Inducible Lipid Droplet Associated | Protein Coding | 31 | GC07P130957 | 22.61833 | https://www.genecards.org/cgi-bin/carddisp.pl?gene=HILPDA |
| ARNT | Aryl Hydrocarbon Receptor Nuclear Translocator | Protein Coding | 42 | GC01M150809 | 22.54662 | https://www.genecards.org/cgi-bin/carddisp.pl?gene=ARNT |
| HIGD1A | HIG1 Hypoxia Inducible Domain Family Member 1A | Protein Coding | 36 | GC03M042782 | 20.38959 | https://www.genecards.org/cgi-bin/carddisp.pl?gene=HIGD1A |
| CA9 | Carbonic Anhydrase 9 | Protein Coding | 42 | GC09P035673 | 17.85834 | https://www.genecards.org/cgi-bin/carddisp.pl?gene=CA9 |
| SETD2 | SET Domain Containing 2, Histone Lysine Methyltransferase | Protein Coding | 44 | GC03M047033 | 17.7366 | https://www.genecards.org/cgi-bin/carddisp.pl?gene=SETD2 |
| HIGD2A | HIG1 Hypoxia Inducible Domain Family Member 2A | Protein Coding | 32 | GC05P176388 | 17.69774 | https://www.genecards.org/cgi-bin/carddisp.pl?gene=HIGD2A |
| HIGD1B | HIG1 Hypoxia Inducible Domain Family Member 1B | Protein Coding | 29 | GC17P048058 | 17.38243 | https://www.genecards.org/cgi-bin/carddisp.pl?gene=HIGD1B |
| EP300 | E1A Binding Protein P300 | Protein Coding | 48 | GC22P041091 | 15.14089 | https://www.genecards.org/cgi-bin/carddisp.pl?gene=EP300 |
| EPO | Erythropoietin | Protein Coding | 38 | GC07P100720 | 14.82588 | https://www.genecards.org/cgi-bin/carddisp.pl?gene=EPO |
| TP53 | Tumor Protein P53 | Protein Coding | 50 | GC17M007661 | 14.70023 | https://www.genecards.org/cgi-bin/carddisp.pl?gene=TP53 |
| P4HTM | Prolyl 4-Hydroxylase, Transmembrane | Protein Coding | 34 | GC03P049437 | 13.80423 | https://www.genecards.org/cgi-bin/carddisp.pl?gene=P4HTM |
| MTOR | Mechanistic Target Of Rapamycin Kinase | Protein Coding | 51 | GC01M011106 | 13.03731 | https://www.genecards.org/cgi-bin/carddisp.pl?gene=MTOR |
| CITED2 | Cbp/P300 Interacting Transactivator With Glu/Asp Rich Carboxy-Terminal Domain 2 | Protein Coding | 42 | GC06M139371 | 11.4959 | https://www.genecards.org/cgi-bin/carddisp.pl?gene=CITED2 |
| PDK1 | Pyruvate Dehydrogenase Kinase 1 | Protein Coding | 44 | GC02P172555 | 11.46154 | https://www.genecards.org/cgi-bin/carddisp.pl?gene=PDK1 |
| HIGD1C | HIG1 Hypoxia Inducible Domain Family Member 1C | Protein Coding | 27 | GC12P050936 | 11.27018 | https://www.genecards.org/cgi-bin/carddisp.pl?gene=HIGD1C |
| CREB1 | CAMP Responsive Element Binding Protein 1 | Protein Coding | 47 | GC02P207529 | 11.26891 | https://www.genecards.org/cgi-bin/carddisp.pl?gene=CREB1 |
| CREBBP | CREB Binding Protein | Protein Coding | 50 | GC16M005641 | 11.18599 | https://www.genecards.org/cgi-bin/carddisp.pl?gene=CREBBP |
| HIGD2B | HIG1 Hypoxia Inducible Domain Family Member 2B | Protein Coding | 22 | GC15M072675 | 11.03349 | https://www.genecards.org/cgi-bin/carddisp.pl?gene=HIGD2B |
| TIGAR | TP53 Induced Glycolysis Regulatory Phosphatase | Protein Coding | 35 | GC12P016342 | 10.48112 | https://www.genecards.org/cgi-bin/carddisp.pl?gene=TIGAR |
| JUN | Jun Proto-Oncogene, AP-1 Transcription Factor Subunit | Protein Coding | 46 | GC01M058780 | 10.44021 | https://www.genecards.org/cgi-bin/carddisp.pl?gene=JUN |
| CUL2 | Cullin 2 | Protein Coding | 40 | GC10M035046 | 10.43208 | https://www.genecards.org/cgi-bin/carddisp.pl?gene=CUL2 |
| HIGD1AP1 | HIG1 Hypoxia Inducible Domain Family Member 1A Pseudogene 1 | Pseudogene | 6 | GC12P053141 | 10.26927 | https://www.genecards.org/cgi-bin/carddisp.pl?gene=HIGD1AP1 |
| CASP3 | Caspase 3 | Protein Coding | 47 | GC04M184627 | 9.899831 | https://www.genecards.org/cgi-bin/carddisp.pl?gene=CASP3 |
| REST | RE1 Silencing Transcription Factor | Protein Coding | 42 | GC04P056907 | 9.829272 | https://www.genecards.org/cgi-bin/carddisp.pl?gene=REST |
| SLC2A1 | Solute Carrier Family 2 Member 1 | Protein Coding | 50 | GC01M042925 | 9.809032 | https://www.genecards.org/cgi-bin/carddisp.pl?gene=SLC2A1 |
| MGARP | Mitochondria Localized Glutamic Acid Rich Protein | Protein Coding | 31 | GC04M139266 | 9.458321 | https://www.genecards.org/cgi-bin/carddisp.pl?gene=MGARP |
| PSMA7 | Proteasome 20S Subunit Alpha 7 | Protein Coding | 42 | GC20M062136 | 9.3877 | https://www.genecards.org/cgi-bin/carddisp.pl?gene=PSMA7 |
| HIGD1AP18 | HIG1 Hypoxia Inducible Domain Family Member 1A Pseudogene 18 | Pseudogene | 7 | GC08M077013 | 9.292888 | https://www.genecards.org/cgi-bin/carddisp.pl?gene=HIGD1AP18 |
| HIGD1AP10 | HIG1 Hypoxia Inducible Domain Family Member 1A Pseudogene 10 | Pseudogene | 6 | GC11M079990 | 9.292888 | https://www.genecards.org/cgi-bin/carddisp.pl?gene=HIGD1AP10 |
| HIGD1AP11 | HIG1 Hypoxia Inducible Domain Family Member 1A Pseudogene 11 | Pseudogene | 6 | GC01M053075 | 9.292888 | https://www.genecards.org/cgi-bin/carddisp.pl?gene=HIGD1AP11 |
| HIGD1AP13 | HIG1 Hypoxia Inducible Domain Family Member 1A Pseudogene 13 | Pseudogene | 6 | GC04M078648 | 9.292888 | https://www.genecards.org/cgi-bin/carddisp.pl?gene=HIGD1AP13 |
| HIGD1AP16 | HIG1 Hypoxia Inducible Domain Family Member 1A Pseudogene 16 | Pseudogene | 6 | GC20P035879 | 9.292888 | https://www.genecards.org/cgi-bin/carddisp.pl?gene=HIGD1AP16 |
| HIGD1AP5 | HIG1 Hypoxia Inducible Domain Family Member 1A Pseudogene 5 | Pseudogene | 6 | GC11P018106 | 9.292888 | https://www.genecards.org/cgi-bin/carddisp.pl?gene=HIGD1AP5 |
| HIGD1AP12 | HIG1 Hypoxia Inducible Domain Family Member 1A Pseudogene 12 | Pseudogene | 5 | GC01P111380 | 9.292888 | https://www.genecards.org/cgi-bin/carddisp.pl?gene=HIGD1AP12 |
| HIGD1AP14 | HIG1 Hypoxia Inducible Domain Family Member 1A Pseudogene 14 | Pseudogene | 5 | GC04P109673 | 9.292888 | https://www.genecards.org/cgi-bin/carddisp.pl?gene=HIGD1AP14 |
| HIGD1AP15 | HIG1 Hypoxia Inducible Domain Family Member 1A Pseudogene 15 | Pseudogene | 5 | GC20P010334 | 9.292888 | https://www.genecards.org/cgi-bin/carddisp.pl?gene=HIGD1AP15 |
| HIGD1AP17 | HIG1 Hypoxia Inducible Domain Family Member 1A Pseudogene 17 | Pseudogene | 5 | GC14P030964 | 9.292888 | https://www.genecards.org/cgi-bin/carddisp.pl?gene=HIGD1AP17 |
| HIGD1AP2 | HIG1 Hypoxia Inducible Domain Family Member 1A Pseudogene 2 | Pseudogene | 5 | GC13P081272 | 9.292888 | https://www.genecards.org/cgi-bin/carddisp.pl?gene=HIGD1AP2 |
| HIGD1AP3 | HIG1 Hypoxia Inducible Domain Family Member 1A Pseudogene 3 | Pseudogene | 5 | GC05P174632 | 9.292888 | https://www.genecards.org/cgi-bin/carddisp.pl?gene=HIGD1AP3 |
| HIGD1AP4 | HIG1 Hypoxia Inducible Domain Family Member 1A Pseudogene 4 | Pseudogene | 5 | GC02M223398 | 9.292888 | https://www.genecards.org/cgi-bin/carddisp.pl?gene=HIGD1AP4 |
| HIGD1AP6 | HIG1 Hypoxia Inducible Domain Family Member 1A Pseudogene 6 | Pseudogene | 5 | GC08P075302 | 9.292888 | https://www.genecards.org/cgi-bin/carddisp.pl?gene=HIGD1AP6 |
| HIGD1AP8 | HIG1 Hypoxia Inducible Domain Family Member 1A Pseudogene 8 | Pseudogene | 5 | GC12P011481 | 9.292888 | https://www.genecards.org/cgi-bin/carddisp.pl?gene=HIGD1AP8 |
| HIGD1AP9 | HIG1 Hypoxia Inducible Domain Family Member 1A Pseudogene 9 | Pseudogene | 5 | GC12P049619 | 9.292888 | https://www.genecards.org/cgi-bin/carddisp.pl?gene=HIGD1AP9 |
| HIGD1AP7 | HIG1 Hypoxia Inducible Domain Family Member 1A Pseudogene 7 | Pseudogene | 3 | GC07P040115 | 9.292888 | https://www.genecards.org/cgi-bin/carddisp.pl?gene=HIGD1AP7 |
| SESN2 | Sestrin 2 | Protein Coding | 36 | GC01P028270 | 8.723431 | https://www.genecards.org/cgi-bin/carddisp.pl?gene=SESN2 |
| CTNNB1 | Catenin Beta 1 | Protein Coding | 51 | GC03P041236 | 8.694365 | https://www.genecards.org/cgi-bin/carddisp.pl?gene=CTNNB1 |
| EGLN3P1 | Egl-9 Family Hypoxia-Inducible Factor 3 Pseudogene 1 | Pseudogene | 5 | GC12M015971 | 8.602478 | https://www.genecards.org/cgi-bin/carddisp.pl?gene=EGLN3P1 |
| EGLN1P1 | Egl-9 Family Hypoxia Inducible Factor 1 Pseudogene 1 | Pseudogene | 3 | GC15P084633 | 8.602478 | https://www.genecards.org/cgi-bin/carddisp.pl?gene=EGLN1P1 |
| ENSG00000254591 | HIG1 Hypoxia Inducible Domain Family (HIG1) Pseudogene | Pseudogene | 3 | GC11P127657 | 8.602478 | https://www.genecards.org/cgi-bin/carddisp.pl?gene=ENSG00000254591 |
| ENSG00000271291 | HIG1 Hypoxia Inducible Domain Family, Member 1C (HIGD1C) Pseudogene | Pseudogene | 2 | GC01M145269 | 8.602478 | https://www.genecards.org/cgi-bin/carddisp.pl?gene=ENSG00000271291 |
| ENSG00000271455 | HIG1 Hypoxia Inducible Domain Family, Member 1A (HIGD1A) Pseudogene | Pseudogene | 2 | GC05P050603 | 8.602478 | https://www.genecards.org/cgi-bin/carddisp.pl?gene=ENSG00000271455 |
| ENSG00000270577 | HIG1 Hypoxia Inducible Domain Family, Member 1A (HIGD1A) Pseudogene | Pseudogene | 1 | GC07P040349 | 8.602478 | https://www.genecards.org/cgi-bin/carddisp.pl?gene=ENSG00000270577 |
| ENSG00000271439 | HIG1 Hypoxia Inducible Domain Family, Member 1C (HIGD1C) Pseudogene | Pseudogene | 1 | GC01M144401 | 8.602478 | https://www.genecards.org/cgi-bin/carddisp.pl?gene=ENSG00000271439 |
| IGF1 | Insulin Like Growth Factor 1 | Protein Coding | 47 | GC12M102395 | 7.632929 | https://www.genecards.org/cgi-bin/carddisp.pl?gene=IGF1 |
| BNIP3 | BCL2 Interacting Protein 3 | Protein Coding | 40 | GC10M131966 | 7.615863 | https://www.genecards.org/cgi-bin/carddisp.pl?gene=BNIP3 |
| NOS2 | Nitric Oxide Synthase 2 | Protein Coding | 47 | GC17M027756 | 7.541178 | https://www.genecards.org/cgi-bin/carddisp.pl?gene=NOS2 |
| CASP9 | Caspase 9 | Protein Coding | 46 | GC01M015491 | 7.519491 | https://www.genecards.org/cgi-bin/carddisp.pl?gene=CASP9 |
| MIR210 | MicroRNA 210 | RNA Gene | 21 | GC11M002025 | 7.196059 | https://www.genecards.org/cgi-bin/carddisp.pl?gene=MIR210 |
| NOS1 | Nitric Oxide Synthase 1 | Protein Coding | 47 | GC12M117208 | 6.971766 | https://www.genecards.org/cgi-bin/carddisp.pl?gene=NOS1 |
| IL6 | Interleukin 6 | Protein Coding | 47 | GC07P022725 | 6.955602 | https://www.genecards.org/cgi-bin/carddisp.pl?gene=IL6 |
| BCL2 | BCL2 Apoptosis Regulator | Protein Coding | 48 | GC18M063123 | 6.951686 | https://www.genecards.org/cgi-bin/carddisp.pl?gene=BCL2 |
| EDN1 | Endothelin 1 | Protein Coding | 45 | GC06P012256 | 6.924145 | https://www.genecards.org/cgi-bin/carddisp.pl?gene=EDN1 |
| HMOX1 | Heme Oxygenase 1 | Protein Coding | 50 | GC22P035380 | 6.889862 | https://www.genecards.org/cgi-bin/carddisp.pl?gene=HMOX1 |
| TNF | Tumor Necrosis Factor | Protein Coding | 48 | GC06P070143 | 6.772481 | https://www.genecards.org/cgi-bin/carddisp.pl?gene=TNF |
| MAPK1 | Mitogen-Activated Protein Kinase 1 | Protein Coding | 50 | GC22M021759 | 6.506578 | https://www.genecards.org/cgi-bin/carddisp.pl?gene=MAPK1 |
| AKT1 | AKT Serine/Threonine Kinase 1 | Protein Coding | 51 | GC14M104769 | 6.503428 | https://www.genecards.org/cgi-bin/carddisp.pl?gene=AKT1 |
| CXCL8 | C-X-C Motif Chemokine Ligand 8 | Protein Coding | 39 | GC04P073740 | 6.456062 | https://www.genecards.org/cgi-bin/carddisp.pl?gene=CXCL8 |
| PTK2 | Protein Tyrosine Kinase 2 | Protein Coding | 45 | GC08M140657 | 6.355196 | https://www.genecards.org/cgi-bin/carddisp.pl?gene=PTK2 |
| FOS | Fos Proto-Oncogene, AP-1 Transcription Factor Subunit | Protein Coding | 48 | GC14P075278 | 6.230042 | https://www.genecards.org/cgi-bin/carddisp.pl?gene=FOS |
| PTGS2 | Prostaglandin-Endoperoxide Synthase 2 | Protein Coding | 46 | GC01M186640 | 6.164941 | https://www.genecards.org/cgi-bin/carddisp.pl?gene=PTGS2 |
| DDIT4 | DNA Damage Inducible Transcript 4 | Protein Coding | 41 | GC10P072273 | 6.093686 | https://www.genecards.org/cgi-bin/carddisp.pl?gene=DDIT4 |
| SOD1 | Superoxide Dismutase 1 | Protein Coding | 49 | GC21P031659 | 6.0699 | https://www.genecards.org/cgi-bin/carddisp.pl?gene=SOD1 |
| NOS3 | Nitric Oxide Synthase 3 | Protein Coding | 49 | GC07P150990 | 6.058536 | https://www.genecards.org/cgi-bin/carddisp.pl?gene=NOS3 |
| HIPK2 | Homeodomain Interacting Protein Kinase 2 | Protein Coding | 43 | GC07M139561 | 5.969734 | https://www.genecards.org/cgi-bin/carddisp.pl?gene=HIPK2 |
| TGFB1 | Transforming Growth Factor Beta 1 | Protein Coding | 49 | GC19M041301 | 5.931787 | https://www.genecards.org/cgi-bin/carddisp.pl?gene=TGFB1 |
| CYB5R3 | Cytochrome B5 Reductase 3 | Protein Coding | 43 | GC22M042617 | 5.829235 | https://www.genecards.org/cgi-bin/carddisp.pl?gene=CYB5R3 |
| IL1B | Interleukin 1 Beta | Protein Coding | 46 | GC02M112829 | 5.75903 | https://www.genecards.org/cgi-bin/carddisp.pl?gene=IL1B |
| KDR | Kinase Insert Domain Receptor | Protein Coding | 50 | GC04M055078 | 5.724223 | https://www.genecards.org/cgi-bin/carddisp.pl?gene=KDR |
| RORA | RAR Related Orphan Receptor A | Protein Coding | 47 | GC15M060488 | 5.684119 | https://www.genecards.org/cgi-bin/carddisp.pl?gene=RORA |
| VEGFC | Vascular Endothelial Growth Factor C | Protein Coding | 44 | GC04M176683 | 5.663883 | https://www.genecards.org/cgi-bin/carddisp.pl?gene=VEGFC |
| XDH | Xanthine Dehydrogenase | Protein Coding | 44 | GC02M031334 | 5.581778 | https://www.genecards.org/cgi-bin/carddisp.pl?gene=XDH |
| KMT2A | Lysine Methyltransferase 2A | Protein Coding | 43 | GC11P118436 | 5.577713 | https://www.genecards.org/cgi-bin/carddisp.pl?gene=KMT2A |
| NDRG1 | N-Myc Downstream Regulated 1 | Protein Coding | 43 | GC08M133237 | 5.564857 | https://www.genecards.org/cgi-bin/carddisp.pl?gene=NDRG1 |
| PGF | Placental Growth Factor | Protein Coding | 40 | GC14M074941 | 5.528645 | https://www.genecards.org/cgi-bin/carddisp.pl?gene=PGF |
| CXCR4 | C-X-C Motif Chemokine Receptor 4 | Protein Coding | 50 | GC02M136114 | 5.506914 | https://www.genecards.org/cgi-bin/carddisp.pl?gene=CXCR4 |
| ADM | Adrenomedullin | Protein Coding | 42 | GC11P010304 | 5.437563 | https://www.genecards.org/cgi-bin/carddisp.pl?gene=ADM |
| HSP90AA1 | Heat Shock Protein 90 Alpha Family Class A Member 1 | Protein Coding | 45 | GC14M102080 | 5.421844 | https://www.genecards.org/cgi-bin/carddisp.pl?gene=HSP90AA1 |
| ITGAV | Integrin Subunit Alpha V | Protein Coding | 44 | GC02P186589 | 5.35995 | https://www.genecards.org/cgi-bin/carddisp.pl?gene=ITGAV |
| LRP5 | LDL Receptor Related Protein 5 | Protein Coding | 46 | GC11P068298 | 5.332996 | https://www.genecards.org/cgi-bin/carddisp.pl?gene=LRP5 |
| EGFR | Epidermal Growth Factor Receptor | Protein Coding | 51 | GC07P055019 | 5.316895 | https://www.genecards.org/cgi-bin/carddisp.pl?gene=EGFR |
| BDNF | Brain Derived Neurotrophic Factor | Protein Coding | 44 | GC11M027654 | 5.291331 | https://www.genecards.org/cgi-bin/carddisp.pl?gene=BDNF |
| ANGPT2 | Angiopoietin 2 | Protein Coding | 43 | GC08M006499 | 5.286413 | https://www.genecards.org/cgi-bin/carddisp.pl?gene=ANGPT2 |
| ITGB1 | Integrin Subunit Beta 1 | Protein Coding | 47 | GC10M032916 | 5.266937 | https://www.genecards.org/cgi-bin/carddisp.pl?gene=ITGB1 |
| ANGPTL4 | Angiopoietin Like 4 | Protein Coding | 42 | GC19P008363 | 5.243695 | https://www.genecards.org/cgi-bin/carddisp.pl?gene=ANGPTL4 |
| NDP | Norrin Cystine Knot Growth Factor NDP | Protein Coding | 40 | GC0XM043948 | 5.214407 | https://www.genecards.org/cgi-bin/carddisp.pl?gene=NDP |
| MYC | MYC Proto-Oncogene, BHLH Transcription Factor | Protein Coding | 48 | GC08P127735 | 5.20533 | https://www.genecards.org/cgi-bin/carddisp.pl?gene=MYC |
| SIAH2 | Siah E3 Ubiquitin Protein Ligase 2 | Protein Coding | 43 | GC03M150741 | 5.1879 | https://www.genecards.org/cgi-bin/carddisp.pl?gene=SIAH2 |
| EGR1 | Early Growth Response 1 | Protein Coding | 41 | GC05P138465 | 5.135728 | https://www.genecards.org/cgi-bin/carddisp.pl?gene=EGR1 |
| ICAM1 | Intercellular Adhesion Molecule 1 | Protein Coding | 47 | GC19P010272 | 5.123058 | https://www.genecards.org/cgi-bin/carddisp.pl?gene=ICAM1 |
| LEP | Leptin | Protein Coding | 44 | GC07P128241 | 5.106453 | https://www.genecards.org/cgi-bin/carddisp.pl?gene=LEP |
| CCL2 | C-C Motif Chemokine Ligand 2 | Protein Coding | 46 | GC17P034255 | 5.053148 | https://www.genecards.org/cgi-bin/carddisp.pl?gene=CCL2 |
| ELOC | Elongin C | Protein Coding | 31 | GC08M073939 | 5.052884 | https://www.genecards.org/cgi-bin/carddisp.pl?gene=ELOC |
| FLT1 | Fms Related Receptor Tyrosine Kinase 1 | Protein Coding | 48 | GC13M028300 | 5.049433 | https://www.genecards.org/cgi-bin/carddisp.pl?gene=FLT1 |
| MIR186 | MicroRNA 186 | RNA Gene | 18 | GC01M071067 | 5.048089 | https://www.genecards.org/cgi-bin/carddisp.pl?gene=MIR186 |
| MMP2 | Matrix Metallopeptidase 2 | Protein Coding | 50 | GC16P055390 | 5.032588 | https://www.genecards.org/cgi-bin/carddisp.pl?gene=MMP2 |
| MAPK14 | Mitogen-Activated Protein Kinase 14 | Protein Coding | 49 | GC06P070283 | 4.997003 | https://www.genecards.org/cgi-bin/carddisp.pl?gene=MAPK14 |
| CAMK4 | Calcium/Calmodulin Dependent Protein Kinase IV | Protein Coding | 44 | GC05P111223 | 4.990553 | https://www.genecards.org/cgi-bin/carddisp.pl?gene=CAMK4 |
| SERPINE1 | Serpin Family E Member 1 | Protein Coding | 47 | GC07P101127 | 4.940152 | https://www.genecards.org/cgi-bin/carddisp.pl?gene=SERPINE1 |
| NOTCH1 | Notch Receptor 1 | Protein Coding | 48 | GC09M137034 | 4.924378 | https://www.genecards.org/cgi-bin/carddisp.pl?gene=NOTCH1 |
| SP1 | Sp1 Transcription Factor | Protein Coding | 42 | GC12P053380 | 4.856959 | https://www.genecards.org/cgi-bin/carddisp.pl?gene=SP1 |
| CXCL12 | C-X-C Motif Chemokine Ligand 12 | Protein Coding | 43 | GC10M044294 | 4.849091 | https://www.genecards.org/cgi-bin/carddisp.pl?gene=CXCL12 |
| STAT3 | Signal Transducer And Activator Of Transcription 3 | Protein Coding | 50 | GC17M042313 | 4.819866 | https://www.genecards.org/cgi-bin/carddisp.pl?gene=STAT3 |
| SIRT2 | Sirtuin 2 | Protein Coding | 45 | GC19M038878 | 4.796113 | https://www.genecards.org/cgi-bin/carddisp.pl?gene=SIRT2 |
| EPOR | Erythropoietin Receptor | Protein Coding | 45 | GC19M011377 | 4.784822 | https://www.genecards.org/cgi-bin/carddisp.pl?gene=EPOR |
| NFKB1 | Nuclear Factor Kappa B Subunit 1 | Protein Coding | 50 | GC04P102501 | 4.755958 | https://www.genecards.org/cgi-bin/carddisp.pl?gene=NFKB1 |
| TEK | TEK Receptor Tyrosine Kinase | Protein Coding | 48 | GC09P027109 | 4.719521 | https://www.genecards.org/cgi-bin/carddisp.pl?gene=TEK |
| SFTPC | Surfactant Protein C | Protein Coding | 42 | GC08P022156 | 4.705863 | https://www.genecards.org/cgi-bin/carddisp.pl?gene=SFTPC |
| SIRT1 | Sirtuin 1 | Protein Coding | 46 | GC10P067884 | 4.67352 | https://www.genecards.org/cgi-bin/carddisp.pl?gene=SIRT1 |
| LDHA | Lactate Dehydrogenase A | Protein Coding | 48 | GC11P018394 | 4.652626 | https://www.genecards.org/cgi-bin/carddisp.pl?gene=LDHA |
| FGF2 | Fibroblast Growth Factor 2 | Protein Coding | 44 | GC04P122826 | 4.647456 | https://www.genecards.org/cgi-bin/carddisp.pl?gene=FGF2 |
| ELOB | Elongin B | Protein Coding | 31 | GC16M005548 | 4.643801 | https://www.genecards.org/cgi-bin/carddisp.pl?gene=ELOB |
| MMP9 | Matrix Metallopeptidase 9 | Protein Coding | 51 | GC20P046008 | 4.588744 | https://www.genecards.org/cgi-bin/carddisp.pl?gene=MMP9 |
| PTEN | Phosphatase And Tensin Homolog | Protein Coding | 50 | GC10P087863 | 4.571723 | https://www.genecards.org/cgi-bin/carddisp.pl?gene=PTEN |
| HK2 | Hexokinase 2 | Protein Coding | 45 | GC02P074833 | 4.560611 | https://www.genecards.org/cgi-bin/carddisp.pl?gene=HK2 |
| PIK3CG | Phosphatidylinositol-4,5-Bisphosphate 3-Kinase Catalytic Subunit Gamma | Protein Coding | 46 | GC07P106865 | 4.540969 | https://www.genecards.org/cgi-bin/carddisp.pl?gene=PIK3CG |
| BAX | BCL2 Associated X, Apoptosis Regulator | Protein Coding | 46 | GC19P048954 | 4.522242 | https://www.genecards.org/cgi-bin/carddisp.pl?gene=BAX |
| PFKFB4 | 6-Phosphofructo-2-Kinase/Fructose-2,6-Biphosphatase 4 | Protein Coding | 39 | GC03M048517 | 4.488513 | https://www.genecards.org/cgi-bin/carddisp.pl?gene=PFKFB4 |
| THBS1 | Thrombospondin 1 | Protein Coding | 42 | GC15P039581 | 4.479963 | https://www.genecards.org/cgi-bin/carddisp.pl?gene=THBS1 |
| TLR4 | Toll Like Receptor 4 | Protein Coding | 48 | GC09P117704 | 4.458732 | https://www.genecards.org/cgi-bin/carddisp.pl?gene=TLR4 |
| C1QTNF3 | C1q And TNF Related 3 | Protein Coding | 35 | GC05M034017 | 4.379675 | https://www.genecards.org/cgi-bin/carddisp.pl?gene=C1QTNF3 |
| INS | Insulin | Protein Coding | 45 | GC11M002159 | 4.332901 | https://www.genecards.org/cgi-bin/carddisp.pl?gene=INS |
| NFE2L2 | NFE2 Like BZIP Transcription Factor 2 | Protein Coding | 46 | GC02M177227 | 4.301895 | https://www.genecards.org/cgi-bin/carddisp.pl?gene=NFE2L2 |
| BCAR1 | BCAR1 Scaffold Protein, Cas Family Member | Protein Coding | 42 | GC16M075228 | 4.300124 | https://www.genecards.org/cgi-bin/carddisp.pl?gene=BCAR1 |
| RWDD3 | RWD Domain Containing 3 | Protein Coding | 34 | GC01P095171 | 4.293846 | https://www.genecards.org/cgi-bin/carddisp.pl?gene=RWDD3 |
| FUNDC1 | FUN14 Domain Containing 1 | Protein Coding | 32 | GC0XM044523 | 4.282744 | https://www.genecards.org/cgi-bin/carddisp.pl?gene=FUNDC1 |
| CYB5A | Cytochrome B5 Type A | Protein Coding | 42 | GC18M074250 | 4.275006 | https://www.genecards.org/cgi-bin/carddisp.pl?gene=CYB5A |
| ARNT2 | Aryl Hydrocarbon Receptor Nuclear Translocator 2 | Protein Coding | 42 | GC15P080404 | 4.264707 | https://www.genecards.org/cgi-bin/carddisp.pl?gene=ARNT2 |
| SRC | SRC Proto-Oncogene, Non-Receptor Tyrosine Kinase | Protein Coding | 49 | GC20P037344 | 4.263458 | https://www.genecards.org/cgi-bin/carddisp.pl?gene=SRC |
| RHOA | Ras Homolog Family Member A | Protein Coding | 45 | GC03M049359 | 4.24055 | https://www.genecards.org/cgi-bin/carddisp.pl?gene=RHOA |
| ETS1 | ETS Proto-Oncogene 1, Transcription Factor | Protein Coding | 46 | GC11M128458 | 4.238978 | https://www.genecards.org/cgi-bin/carddisp.pl?gene=ETS1 |
| MIF | Macrophage Migration Inhibitory Factor | Protein Coding | 47 | GC22P023894 | 4.229108 | https://www.genecards.org/cgi-bin/carddisp.pl?gene=MIF |
| MDM2 | MDM2 Proto-Oncogene | Protein Coding | 50 | GC12P068808 | 4.200527 | https://www.genecards.org/cgi-bin/carddisp.pl?gene=MDM2 |
| PRKAA2 | Protein Kinase AMP-Activated Catalytic Subunit Alpha 2 | Protein Coding | 48 | GC01P056645 | 4.192748 | https://www.genecards.org/cgi-bin/carddisp.pl?gene=PRKAA2 |
| OIP5-AS1 | OIP5 Antisense RNA 1 | RNA Gene | 18 | GC15P041300 | 4.188402 | https://www.genecards.org/cgi-bin/carddisp.pl?gene=OIP5-AS1 |
| ENG | Endoglin | Protein Coding | 44 | GC09M127815 | 4.179848 | https://www.genecards.org/cgi-bin/carddisp.pl?gene=ENG |
| LOXL2 | Lysyl Oxidase Like 2 | Protein Coding | 42 | GC08M023296 | 4.150548 | https://www.genecards.org/cgi-bin/carddisp.pl?gene=LOXL2 |
| FAM162A | Family With Sequence Similarity 162 Member A | Protein Coding | 32 | GC03P122384 | 4.141071 | https://www.genecards.org/cgi-bin/carddisp.pl?gene=FAM162A |
| CRP | C-Reactive Protein | Protein Coding | 44 | GC01M159723 | 4.13706 | https://www.genecards.org/cgi-bin/carddisp.pl?gene=CRP |
| TSPAN12 | Tetraspanin 12 | Protein Coding | 42 | GC07M120787 | 4.127964 | https://www.genecards.org/cgi-bin/carddisp.pl?gene=TSPAN12 |
| ZNF408 | Zinc Finger Protein 408 | Protein Coding | 35 | GC11P046700 | 4.127964 | https://www.genecards.org/cgi-bin/carddisp.pl?gene=ZNF408 |
| NAA10 | N-Alpha-Acetyltransferase 10, NatA Catalytic Subunit | Protein Coding | 40 | GC0XM153929 | 4.079876 | https://www.genecards.org/cgi-bin/carddisp.pl?gene=NAA10 |
| NOX4 | NADPH Oxidase 4 | Protein Coding | 40 | GC11M089324 | 4.061869 | https://www.genecards.org/cgi-bin/carddisp.pl?gene=NOX4 |
| ADA | Adenosine Deaminase | Protein Coding | 48 | GC20M044620 | 4.06013 | https://www.genecards.org/cgi-bin/carddisp.pl?gene=ADA |
| ACE | Angiotensin I Converting Enzyme | Protein Coding | 47 | GC17P063477 | 4.057723 | https://www.genecards.org/cgi-bin/carddisp.pl?gene=ACE |
| SPP1 | Secreted Phosphoprotein 1 | Protein Coding | 43 | GC04P087975 | 4.050842 | https://www.genecards.org/cgi-bin/carddisp.pl?gene=SPP1 |
| FLCN | Folliculin | Protein Coding | 39 | GC17M017206 | 4.027118 | https://www.genecards.org/cgi-bin/carddisp.pl?gene=FLCN |
| FZD4 | Frizzled Class Receptor 4 | Protein Coding | 48 | GC11M086945 | 4.02584 | https://www.genecards.org/cgi-bin/carddisp.pl?gene=FZD4 |
| MAPK8 | Mitogen-Activated Protein Kinase 8 | Protein Coding | 47 | GC10P048306 | 4.014141 | https://www.genecards.org/cgi-bin/carddisp.pl?gene=MAPK8 |
| LOC106728418 | LEP 5' Regulatory Region | Biological Region | 1 | GC07P128238 | 3.992956 | https://www.genecards.org/cgi-bin/carddisp.pl?gene=LOC106728418 |
| MIR21 | MicroRNA 21 | RNA Gene | 23 | GC17P059841 | 3.967147 | https://www.genecards.org/cgi-bin/carddisp.pl?gene=MIR21 |
| PKM | Pyruvate Kinase M1/2 | Protein Coding | 43 | GC15M072199 | 3.961989 | https://www.genecards.org/cgi-bin/carddisp.pl?gene=PKM |
| UBC | Ubiquitin C | Protein Coding | 40 | GC12M124911 | 3.944076 | https://www.genecards.org/cgi-bin/carddisp.pl?gene=UBC |
| ENO1 | Enolase 1 | Protein Coding | 45 | GC01M008861 | 3.934587 | https://www.genecards.org/cgi-bin/carddisp.pl?gene=ENO1 |
| SOD2 | Superoxide Dismutase 2 | Protein Coding | 48 | GC06M159669 | 3.92256 | https://www.genecards.org/cgi-bin/carddisp.pl?gene=SOD2 |
| PLK3 | Polo Like Kinase 3 | Protein Coding | 41 | GC01P044799 | 3.921823 | https://www.genecards.org/cgi-bin/carddisp.pl?gene=PLK3 |
| BHLHE40 | Basic Helix-Loop-Helix Family Member E40 | Protein Coding | 39 | GC03P004980 | 3.920456 | https://www.genecards.org/cgi-bin/carddisp.pl?gene=BHLHE40 |
| IGFBP1 | Insulin Like Growth Factor Binding Protein 1 | Protein Coding | 41 | GC07P047280 | 3.904173 | https://www.genecards.org/cgi-bin/carddisp.pl?gene=IGFBP1 |
| HLA-DRB1 | Major Histocompatibility Complex, Class II, DR Beta 1 | Protein Coding | 45 | GC06M032578 | 3.894995 | https://www.genecards.org/cgi-bin/carddisp.pl?gene=HLA-DRB1 |
| BECN1 | Beclin 1 | Protein Coding | 43 | GC17M042810 | 3.892993 | https://www.genecards.org/cgi-bin/carddisp.pl?gene=BECN1 |
| CDH1 | Cadherin 1 | Protein Coding | 47 | GC16P068737 | 3.877492 | https://www.genecards.org/cgi-bin/carddisp.pl?gene=CDH1 |
| PRKAA1 | Protein Kinase AMP-Activated Catalytic Subunit Alpha 1 | Protein Coding | 45 | GC05M040759 | 3.874086 | https://www.genecards.org/cgi-bin/carddisp.pl?gene=PRKAA1 |
| CHRDL1 | Chordin Like 1 | Protein Coding | 38 | GC0XM110674 | 3.833978 | https://www.genecards.org/cgi-bin/carddisp.pl?gene=CHRDL1 |
| ZFP36 | ZFP36 Ring Finger Protein | Protein Coding | 38 | GC19P039406 | 3.816728 | https://www.genecards.org/cgi-bin/carddisp.pl?gene=ZFP36 |
| AHR | Aryl Hydrocarbon Receptor | Protein Coding | 46 | GC07P016916 | 3.804995 | https://www.genecards.org/cgi-bin/carddisp.pl?gene=AHR |
| IFNG | Interferon Gamma | Protein Coding | 45 | GC12M068154 | 3.80328 | https://www.genecards.org/cgi-bin/carddisp.pl?gene=IFNG |
| CCN2 | Cellular Communication Network Factor 2 | Protein Coding | 36 | GC06M131948 | 3.79803 | https://www.genecards.org/cgi-bin/carddisp.pl?gene=CCN2 |
| MAPK3 | Mitogen-Activated Protein Kinase 3 | Protein Coding | 47 | GC16M034315 | 3.785044 | https://www.genecards.org/cgi-bin/carddisp.pl?gene=MAPK3 |
| CAT | Catalase | Protein Coding | 47 | GC11P034460 | 3.761861 | https://www.genecards.org/cgi-bin/carddisp.pl?gene=CAT |
| TH | Tyrosine Hydroxylase | Protein Coding | 50 | GC11M002163 | 3.741168 | https://www.genecards.org/cgi-bin/carddisp.pl?gene=TH |
| IGFBP3 | Insulin Like Growth Factor Binding Protein 3 | Protein Coding | 42 | GC07M045912 | 3.739616 | https://www.genecards.org/cgi-bin/carddisp.pl?gene=IGFBP3 |
| PGK1 | Phosphoglycerate Kinase 1 | Protein Coding | 46 | GC0XP077961 | 3.697083 | https://www.genecards.org/cgi-bin/carddisp.pl?gene=PGK1 |
| EIF4EBP1 | Eukaryotic Translation Initiation Factor 4E Binding Protein 1 | Protein Coding | 44 | GC08P038236 | 3.693162 | https://www.genecards.org/cgi-bin/carddisp.pl?gene=EIF4EBP1 |
| VEGFB | Vascular Endothelial Growth Factor B | Protein Coding | 42 | GC11P064234 | 3.688035 | https://www.genecards.org/cgi-bin/carddisp.pl?gene=VEGFB |
| ANGPT1 | Angiopoietin 1 | Protein Coding | 44 | GC08M107246 | 3.647147 | https://www.genecards.org/cgi-bin/carddisp.pl?gene=ANGPT1 |
| IL10 | Interleukin 10 | Protein Coding | 44 | GC01M206767 | 3.642148 | https://www.genecards.org/cgi-bin/carddisp.pl?gene=IL10 |
| NOL3 | Nucleolar Protein 3 | Protein Coding | 41 | GC16P067191 | 3.638356 | https://www.genecards.org/cgi-bin/carddisp.pl?gene=NOL3 |
| PLAUR | Plasminogen Activator, Urokinase Receptor | Protein Coding | 42 | GC19M043646 | 3.617465 | https://www.genecards.org/cgi-bin/carddisp.pl?gene=PLAUR |
| CYCS | Cytochrome C, Somatic | Protein Coding | 46 | GC07M025118 | 3.606098 | https://www.genecards.org/cgi-bin/carddisp.pl?gene=CYCS |
| ESR1 | Estrogen Receptor 1 | Protein Coding | 50 | GC06P151656 | 3.600837 | https://www.genecards.org/cgi-bin/carddisp.pl?gene=ESR1 |
| RBX1 | Ring-Box 1 | Protein Coding | 40 | GC22P040951 | 3.595835 | https://www.genecards.org/cgi-bin/carddisp.pl?gene=RBX1 |
| DPP4 | Dipeptidyl Peptidase 4 | Protein Coding | 47 | GC02M161992 | 3.592291 | https://www.genecards.org/cgi-bin/carddisp.pl?gene=DPP4 |
| TWIST1 | Twist Family BHLH Transcription Factor 1 | Protein Coding | 44 | GC07M019020 | 3.591008 | https://www.genecards.org/cgi-bin/carddisp.pl?gene=TWIST1 |
| AR | Androgen Receptor | Protein Coding | 50 | GC0XP067544 | 3.588771 | https://www.genecards.org/cgi-bin/carddisp.pl?gene=AR |
| NDN | Necdin, MAGE Family Member | Protein Coding | 40 | GC15M023686 | 3.577944 | https://www.genecards.org/cgi-bin/carddisp.pl?gene=NDN |
| PIK3CA | Phosphatidylinositol-4,5-Bisphosphate 3-Kinase Catalytic Subunit Alpha | Protein Coding | 51 | GC03P179148 | 3.575959 | https://www.genecards.org/cgi-bin/carddisp.pl?gene=PIK3CA |
| HSPA4 | Heat Shock Protein Family A (Hsp70) Member 4 | Protein Coding | 40 | GC05P133051 | 3.5632 | https://www.genecards.org/cgi-bin/carddisp.pl?gene=HSPA4 |
| RAC1 | Rac Family Small GTPase 1 | Protein Coding | 47 | GC07P006377 | 3.562739 | https://www.genecards.org/cgi-bin/carddisp.pl?gene=RAC1 |
| SIAH1 | Siah E3 Ubiquitin Protein Ligase 1 | Protein Coding | 45 | GC16M048357 | 3.541292 | https://www.genecards.org/cgi-bin/carddisp.pl?gene=SIAH1 |
| SDHB | Succinate Dehydrogenase Complex Iron Sulfur Subunit B | Protein Coding | 46 | GC01M017638 | 3.534531 | https://www.genecards.org/cgi-bin/carddisp.pl?gene=SDHB |
| SLC2A3 | Solute Carrier Family 2 Member 3 | Protein Coding | 46 | GC12M007919 | 3.530287 | https://www.genecards.org/cgi-bin/carddisp.pl?gene=SLC2A3 |
| ERBB2 | Erb-B2 Receptor Tyrosine Kinase 2 | Protein Coding | 51 | GC17P039687 | 3.505382 | https://www.genecards.org/cgi-bin/carddisp.pl?gene=ERBB2 |
| USP28 | Ubiquitin Specific Peptidase 28 | Protein Coding | 38 | GC11M113797 | 3.499873 | https://www.genecards.org/cgi-bin/carddisp.pl?gene=USP28 |
| BNIP3L | BCL2 Interacting Protein 3 Like | Protein Coding | 40 | GC08P026296 | 3.499449 | https://www.genecards.org/cgi-bin/carddisp.pl?gene=BNIP3L |
| DICER1 | Dicer 1, Ribonuclease III | Protein Coding | 46 | GC14M095086 | 3.496871 | https://www.genecards.org/cgi-bin/carddisp.pl?gene=DICER1 |
| LOX | Lysyl Oxidase | Protein Coding | 43 | GC05M122063 | 3.458662 | https://www.genecards.org/cgi-bin/carddisp.pl?gene=LOX |
| ZEB1 | Zinc Finger E-Box Binding Homeobox 1 | Protein Coding | 46 | GC10P031318 | 3.456419 | https://www.genecards.org/cgi-bin/carddisp.pl?gene=ZEB1 |
| ALKBH5 | AlkB Homolog 5, RNA Demethylase | Protein Coding | 32 | GC17P018183 | 3.439524 | https://www.genecards.org/cgi-bin/carddisp.pl?gene=ALKBH5 |
| KCNB1 | Potassium Voltage-Gated Channel Subfamily B Member 1 | Protein Coding | 46 | GC20M049293 | 3.434923 | https://www.genecards.org/cgi-bin/carddisp.pl?gene=KCNB1 |
| WDR83 | WD Repeat Domain 83 | Protein Coding | 35 | GC19P012666 | 3.433867 | https://www.genecards.org/cgi-bin/carddisp.pl?gene=WDR83 |
| UBE2D1 | Ubiquitin Conjugating Enzyme E2 D1 | Protein Coding | 43 | GC10P058334 | 3.428715 | https://www.genecards.org/cgi-bin/carddisp.pl?gene=UBE2D1 |
| PPARG | Peroxisome Proliferator Activated Receptor Gamma | Protein Coding | 50 | GC03P012287 | 3.415056 | https://www.genecards.org/cgi-bin/carddisp.pl?gene=PPARG |
| HGF | Hepatocyte Growth Factor | Protein Coding | 49 | GC07M081699 | 3.392186 | https://www.genecards.org/cgi-bin/carddisp.pl?gene=HGF |
| CDH5 | Cadherin 5 | Protein Coding | 44 | GC16P066366 | 3.39064 | https://www.genecards.org/cgi-bin/carddisp.pl?gene=CDH5 |
| SLC16A1 | Solute Carrier Family 16 Member 1 | Protein Coding | 47 | GC01M112947 | 3.38913 | https://www.genecards.org/cgi-bin/carddisp.pl?gene=SLC16A1 |
| ERO1A | Endoplasmic Reticulum Oxidoreductase 1 Alpha | Protein Coding | 32 | GC14M052640 | 3.37508 | https://www.genecards.org/cgi-bin/carddisp.pl?gene=ERO1A |
| RBPJ | Recombination Signal Binding Protein For Immunoglobulin Kappa J Region | Protein Coding | 45 | GC04P026165 | 3.374254 | https://www.genecards.org/cgi-bin/carddisp.pl?gene=RBPJ |
| NPPA | Natriuretic Peptide A | Protein Coding | 44 | GC01M011846 | 3.371777 | https://www.genecards.org/cgi-bin/carddisp.pl?gene=NPPA |
| SERPINF1 | Serpin Family F Member 1 | Protein Coding | 42 | GC17P001761 | 3.370211 | https://www.genecards.org/cgi-bin/carddisp.pl?gene=SERPINF1 |
| PDGFB | Platelet Derived Growth Factor Subunit B | Protein Coding | 47 | GC22M053523 | 3.370089 | https://www.genecards.org/cgi-bin/carddisp.pl?gene=PDGFB |
| SMAD3 | SMAD Family Member 3 | Protein Coding | 47 | GC15P067063 | 3.361651 | https://www.genecards.org/cgi-bin/carddisp.pl?gene=SMAD3 |
| CDKN1A | Cyclin Dependent Kinase Inhibitor 1A | Protein Coding | 46 | GC06P070291 | 3.341463 | https://www.genecards.org/cgi-bin/carddisp.pl?gene=CDKN1A |
| KCNJ5 | Potassium Inwardly Rectifying Channel Subfamily J Member 5 | Protein Coding | 45 | GC11P128891 | 3.340906 | https://www.genecards.org/cgi-bin/carddisp.pl?gene=KCNJ5 |
| TYMP | Thymidine Phosphorylase | Protein Coding | 44 | GC22M050525 | 3.318821 | https://www.genecards.org/cgi-bin/carddisp.pl?gene=TYMP |
| ABCB1 | ATP Binding Cassette Subfamily B Member 1 | Protein Coding | 49 | GC07M087504 | 3.312148 | https://www.genecards.org/cgi-bin/carddisp.pl?gene=ABCB1 |
| MB | Myoglobin | Protein Coding | 42 | GC22M035606 | 3.306379 | https://www.genecards.org/cgi-bin/carddisp.pl?gene=MB |
| TXN | Thioredoxin | Protein Coding | 43 | GC09M110243 | 3.294459 | https://www.genecards.org/cgi-bin/carddisp.pl?gene=TXN |
| ADORA2B | Adenosine A2b Receptor | Protein Coding | 45 | GC17P015927 | 3.292248 | https://www.genecards.org/cgi-bin/carddisp.pl?gene=ADORA2B |
| CD36 | CD36 Molecule | Protein Coding | 46 | GC07P080369 | 3.283334 | https://www.genecards.org/cgi-bin/carddisp.pl?gene=CD36 |
| SNAI1 | Snail Family Transcriptional Repressor 1 | Protein Coding | 42 | GC20P049982 | 3.281973 | https://www.genecards.org/cgi-bin/carddisp.pl?gene=SNAI1 |
| KLHL20 | Kelch Like Family Member 20 | Protein Coding | 37 | GC01P173714 | 3.274997 | https://www.genecards.org/cgi-bin/carddisp.pl?gene=KLHL20 |
| S100B | S100 Calcium Binding Protein B | Protein Coding | 43 | GC21M049546 | 3.258669 | https://www.genecards.org/cgi-bin/carddisp.pl?gene=S100B |
| USP19 | Ubiquitin Specific Peptidase 19 | Protein Coding | 38 | GC03M050581 | 3.252656 | https://www.genecards.org/cgi-bin/carddisp.pl?gene=USP19 |
| FOXO3 | Forkhead Box O3 | Protein Coding | 42 | GC06P108559 | 3.24894 | https://www.genecards.org/cgi-bin/carddisp.pl?gene=FOXO3 |
| NFKBIA | NFKB Inhibitor Alpha | Protein Coding | 48 | GC14M035401 | 3.248831 | https://www.genecards.org/cgi-bin/carddisp.pl?gene=NFKBIA |
| ADORA1 | Adenosine A1 Receptor | Protein Coding | 45 | GC01P203090 | 3.248551 | https://www.genecards.org/cgi-bin/carddisp.pl?gene=ADORA1 |
| FN1 | Fibronectin 1 | Protein Coding | 48 | GC02M215360 | 3.246318 | https://www.genecards.org/cgi-bin/carddisp.pl?gene=FN1 |
| DDIT3 | DNA Damage Inducible Transcript 3 | Protein Coding | 43 | GC12M057516 | 3.241561 | https://www.genecards.org/cgi-bin/carddisp.pl?gene=DDIT3 |
| HDAC1 | Histone Deacetylase 1 | Protein Coding | 47 | GC01P032292 | 3.241211 | https://www.genecards.org/cgi-bin/carddisp.pl?gene=HDAC1 |
| SART1 | Spliceosome Associated Factor 1, Recruiter Of U4/U6.U5 Tri-SnRNP | Protein Coding | 38 | GC11P068146 | 3.211614 | https://www.genecards.org/cgi-bin/carddisp.pl?gene=SART1 |
| PPARA | Peroxisome Proliferator Activated Receptor Alpha | Protein Coding | 44 | GC22P046150 | 3.210533 | https://www.genecards.org/cgi-bin/carddisp.pl?gene=PPARA |
| ATF4 | Activating Transcription Factor 4 | Protein Coding | 44 | GC22P039584 | 3.193306 | https://www.genecards.org/cgi-bin/carddisp.pl?gene=ATF4 |
| NGB | Neuroglobin | Protein Coding | 36 | GC14M077265 | 3.188759 | https://www.genecards.org/cgi-bin/carddisp.pl?gene=NGB |
| MMP14 | Matrix Metallopeptidase 14 | Protein Coding | 48 | GC14P029125 | 3.185422 | https://www.genecards.org/cgi-bin/carddisp.pl?gene=MMP14 |
| SLC8A1 | Solute Carrier Family 8 Member A1 | Protein Coding | 43 | GC02M040078 | 3.182996 | https://www.genecards.org/cgi-bin/carddisp.pl?gene=SLC8A1 |
| LOC107303340 | 3p25 Von Hippel-Lindau Tumor Suppressor, E3 Ubiquitin Protein Ligase Alu-Mediated Recombination Region | Biological Region | 2 | GC03P011657 | 3.157767 | https://www.genecards.org/cgi-bin/carddisp.pl?gene=LOC107303340 |
| ADIPOQ | Adiponectin, C1Q And Collagen Domain Containing | Protein Coding | 42 | GC03P186842 | 3.157212 | https://www.genecards.org/cgi-bin/carddisp.pl?gene=ADIPOQ |
| TGFB3 | Transforming Growth Factor Beta 3 | Protein Coding | 44 | GC14M075958 | 3.140905 | https://www.genecards.org/cgi-bin/carddisp.pl?gene=TGFB3 |
| BAD | BCL2 Associated Agonist Of Cell Death | Protein Coding | 43 | GC11M079946 | 3.135208 | https://www.genecards.org/cgi-bin/carddisp.pl?gene=BAD |
| SNRPB | Small Nuclear Ribonucleoprotein Polypeptides B And B1 | Protein Coding | 42 | GC20M002461 | 3.13488 | https://www.genecards.org/cgi-bin/carddisp.pl?gene=SNRPB |
| PDK3 | Pyruvate Dehydrogenase Kinase 3 | Protein Coding | 46 | GC0XP024465 | 3.126187 | https://www.genecards.org/cgi-bin/carddisp.pl?gene=PDK3 |
| SENP1 | SUMO Specific Peptidase 1 | Protein Coding | 42 | GC12M048042 | 3.123264 | https://www.genecards.org/cgi-bin/carddisp.pl?gene=SENP1 |
| MET | MET Proto-Oncogene, Receptor Tyrosine Kinase | Protein Coding | 51 | GC07P116672 | 3.119965 | https://www.genecards.org/cgi-bin/carddisp.pl?gene=MET |
| RPS27A | Ribosomal Protein S27a | Protein Coding | 40 | GC02P055231 | 3.119819 | https://www.genecards.org/cgi-bin/carddisp.pl?gene=RPS27A |
| RELA | RELA Proto-Oncogene, NF-KB Subunit | Protein Coding | 48 | GC11M065653 | 3.114623 | https://www.genecards.org/cgi-bin/carddisp.pl?gene=RELA |
| VEGFD | Vascular Endothelial Growth Factor D | Protein Coding | 31 | GC0XM015345 | 3.094404 | https://www.genecards.org/cgi-bin/carddisp.pl?gene=VEGFD |
| BCL2L1 | BCL2 Like 1 | Protein Coding | 44 | GC20M031664 | 3.09414 | https://www.genecards.org/cgi-bin/carddisp.pl?gene=BCL2L1 |
| BIRC5 | Baculoviral IAP Repeat Containing 5 | Protein Coding | 44 | GC17P078214 | 3.089706 | https://www.genecards.org/cgi-bin/carddisp.pl?gene=BIRC5 |
| ATM | ATM Serine/Threonine Kinase | Protein Coding | 50 | GC11P108222 | 3.088498 | https://www.genecards.org/cgi-bin/carddisp.pl?gene=ATM |
| ALB | Albumin | Protein Coding | 47 | GC04P073397 | 3.075107 | https://www.genecards.org/cgi-bin/carddisp.pl?gene=ALB |
| PRKCA | Protein Kinase C Alpha | Protein Coding | 48 | GC17P066302 | 3.073063 | https://www.genecards.org/cgi-bin/carddisp.pl?gene=PRKCA |
| STC1 | Stanniocalcin 1 | Protein Coding | 39 | GC08M023841 | 3.071695 | https://www.genecards.org/cgi-bin/carddisp.pl?gene=STC1 |
| IGF2 | Insulin Like Growth Factor 2 | Protein Coding | 45 | GC11M002261 | 3.068542 | https://www.genecards.org/cgi-bin/carddisp.pl?gene=IGF2 |
| FURIN | Furin, Paired Basic Amino Acid Cleaving Enzyme | Protein Coding | 44 | GC15P090868 | 3.065678 | https://www.genecards.org/cgi-bin/carddisp.pl?gene=FURIN |
| HMOX2 | Heme Oxygenase 2 | Protein Coding | 45 | GC16P004474 | 3.063962 | https://www.genecards.org/cgi-bin/carddisp.pl?gene=HMOX2 |
| PPARGC1A | PPARG Coactivator 1 Alpha | Protein Coding | 44 | GC04M023755 | 3.058233 | https://www.genecards.org/cgi-bin/carddisp.pl?gene=PPARGC1A |
| HMGB1 | High Mobility Group Box 1 | Protein Coding | 43 | GC13M030456 | 3.049551 | https://www.genecards.org/cgi-bin/carddisp.pl?gene=HMGB1 |
| APLN | Apelin | Protein Coding | 35 | GC0XM129645 | 3.027525 | https://www.genecards.org/cgi-bin/carddisp.pl?gene=APLN |
| ATR | ATR Serine/Threonine Kinase | Protein Coding | 50 | GC03M142449 | 3.026081 | https://www.genecards.org/cgi-bin/carddisp.pl?gene=ATR |
| BSG | Basigin (Ok Blood Group) | Protein Coding | 42 | GC19P000571 | 3.021889 | https://www.genecards.org/cgi-bin/carddisp.pl?gene=BSG |
| ING4 | Inhibitor Of Growth Family Member 4 | Protein Coding | 36 | GC12M006650 | 2.99895 | https://www.genecards.org/cgi-bin/carddisp.pl?gene=ING4 |
| PLAU | Plasminogen Activator, Urokinase | Protein Coding | 48 | GC10P073909 | 2.983077 | https://www.genecards.org/cgi-bin/carddisp.pl?gene=PLAU |
| MANF | Mesencephalic Astrocyte Derived Neurotrophic Factor | Protein Coding | 37 | GC03P051385 | 2.980338 | https://www.genecards.org/cgi-bin/carddisp.pl?gene=MANF |
| HERC2 | HECT And RLD Domain Containing E3 Ubiquitin Protein Ligase 2 | Protein Coding | 42 | GC15M028111 | 2.974412 | https://www.genecards.org/cgi-bin/carddisp.pl?gene=HERC2 |
| MALAT1 | Metastasis Associated Lung Adenocarcinoma Transcript 1 | RNA Gene | 23 | GC11P068100 | 2.970547 | https://www.genecards.org/cgi-bin/carddisp.pl?gene=MALAT1 |
| TRIB3 | Tribbles Pseudokinase 3 | Protein Coding | 40 | GC20P000361 | 2.968949 | https://www.genecards.org/cgi-bin/carddisp.pl?gene=TRIB3 |
| LOC110973015 | NOS3 5' Regulatory Region | Biological Region | 2 | GC07P150988 | 2.964442 | https://www.genecards.org/cgi-bin/carddisp.pl?gene=LOC110973015 |
| KDM3A | Lysine Demethylase 3A | Protein Coding | 39 | GC02P086440 | 2.95775 | https://www.genecards.org/cgi-bin/carddisp.pl?gene=KDM3A |
| ANG | Angiogenin | Protein Coding | 43 | GC14P028926 | 2.953753 | https://www.genecards.org/cgi-bin/carddisp.pl?gene=ANG |
| PLOD2 | Procollagen-Lysine,2-Oxoglutarate 5-Dioxygenase 2 | Protein Coding | 43 | GC03M146069 | 2.949893 | https://www.genecards.org/cgi-bin/carddisp.pl?gene=PLOD2 |
| ACE2 | Angiotensin Converting Enzyme 2 | Protein Coding | 46 | GC0XM015494 | 2.939526 | https://www.genecards.org/cgi-bin/carddisp.pl?gene=ACE2 |
| RGS5 | Regulator Of G Protein Signaling 5 | Protein Coding | 39 | GC01M163111 | 2.935082 | https://www.genecards.org/cgi-bin/carddisp.pl?gene=RGS5 |
| TFRC | Transferrin Receptor | Protein Coding | 46 | GC03M196027 | 2.929524 | https://www.genecards.org/cgi-bin/carddisp.pl?gene=TFRC |
| NCBP2AS2 | NCBP2 Antisense 2 (Head To Head) | Protein Coding | 14 | GC03P196944 | 2.928246 | https://www.genecards.org/cgi-bin/carddisp.pl?gene=NCBP2AS2 |
| GDF15 | Growth Differentiation Factor 15 | Protein Coding | 39 | GC19P031561 | 2.925227 | https://www.genecards.org/cgi-bin/carddisp.pl?gene=GDF15 |
| HSPA5 | Heat Shock Protein Family A (Hsp70) Member 5 | Protein Coding | 44 | GC09M125234 | 2.92484 | https://www.genecards.org/cgi-bin/carddisp.pl?gene=HSPA5 |
| EIF2S1 | Eukaryotic Translation Initiation Factor 2 Subunit Alpha | Protein Coding | 42 | GC14P067359 | 2.924679 | https://www.genecards.org/cgi-bin/carddisp.pl?gene=EIF2S1 |
| NRP1 | Neuropilin 1 | Protein Coding | 44 | GC10M033177 | 2.922473 | https://www.genecards.org/cgi-bin/carddisp.pl?gene=NRP1 |
| ARNTL2 | Aryl Hydrocarbon Receptor Nuclear Translocator Like 2 | Protein Coding | 37 | GC12P027332 | 2.915415 | https://www.genecards.org/cgi-bin/carddisp.pl?gene=ARNTL2 |
| VCAM1 | Vascular Cell Adhesion Molecule 1 | Protein Coding | 43 | GC01P100719 | 2.915205 | https://www.genecards.org/cgi-bin/carddisp.pl?gene=VCAM1 |
| LIMD1 | LIM Domain Containing 1 | Protein Coding | 38 | GC03P045555 | 2.899101 | https://www.genecards.org/cgi-bin/carddisp.pl?gene=LIMD1 |
| MAP2K1 | Mitogen-Activated Protein Kinase Kinase 1 | Protein Coding | 51 | GC15P066386 | 2.895241 | https://www.genecards.org/cgi-bin/carddisp.pl?gene=MAP2K1 |
| NPPB | Natriuretic Peptide B | Protein Coding | 42 | GC01M011858 | 2.891223 | https://www.genecards.org/cgi-bin/carddisp.pl?gene=NPPB |
| CCND1 | Cyclin D1 | Protein Coding | 50 | GC11P069641 | 2.889664 | https://www.genecards.org/cgi-bin/carddisp.pl?gene=CCND1 |
| EIF4E | Eukaryotic Translation Initiation Factor 4E | Protein Coding | 47 | GC04M098879 | 2.889417 | https://www.genecards.org/cgi-bin/carddisp.pl?gene=EIF4E |
| TIMP1 | TIMP Metallopeptidase Inhibitor 1 | Protein Coding | 42 | GC0XP047583 | 2.879516 | https://www.genecards.org/cgi-bin/carddisp.pl?gene=TIMP1 |
| HP1BP3 | Heterochromatin Protein 1 Binding Protein 3 | Protein Coding | 35 | GC01M020742 | 2.870732 | https://www.genecards.org/cgi-bin/carddisp.pl?gene=HP1BP3 |
| PRSS23 | Serine Protease 23 | Protein Coding | 37 | GC11P086791 | 2.869455 | https://www.genecards.org/cgi-bin/carddisp.pl?gene=PRSS23 |
| NDP-AS1 | NDP Antisense RNA 1 | RNA Gene | 11 | GC0XP043949 | 2.869455 | https://www.genecards.org/cgi-bin/carddisp.pl?gene=NDP-AS1 |
| CMTS | Chronic Mountain Sickness, Susceptibility To | Genetic Locus | 1 | GC12U902081 | 2.869455 | https://www.genecards.org/cgi-bin/carddisp.pl?gene=CMTS |
| PSMC6 | Proteasome 26S Subunit, ATPase 6 | Protein Coding | 38 | GC14P052707 | 2.868642 | https://www.genecards.org/cgi-bin/carddisp.pl?gene=PSMC6 |
| PSMD10 | Proteasome 26S Subunit, Non-ATPase 10 | Protein Coding | 38 | GC0XM108084 | 2.865093 | https://www.genecards.org/cgi-bin/carddisp.pl?gene=PSMD10 |
| AQP1 | Aquaporin 1 (Colton Blood Group) | Protein Coding | 44 | GC07P030911 | 2.86161 | https://www.genecards.org/cgi-bin/carddisp.pl?gene=AQP1 |
| MMP1 | Matrix Metallopeptidase 1 | Protein Coding | 48 | GC11M102810 | 2.855419 | https://www.genecards.org/cgi-bin/carddisp.pl?gene=MMP1 |
| HSP90B1 | Heat Shock Protein 90 Beta Family Member 1 | Protein Coding | 43 | GC12P103930 | 2.851137 | https://www.genecards.org/cgi-bin/carddisp.pl?gene=HSP90B1 |
| CYGB | Cytoglobin | Protein Coding | 36 | GC17M076527 | 2.836492 | https://www.genecards.org/cgi-bin/carddisp.pl?gene=CYGB |
| FH | Fumarate Hydratase | Protein Coding | 44 | GC01M241499 | 2.831905 | https://www.genecards.org/cgi-bin/carddisp.pl?gene=FH |
| GAPDH | Glyceraldehyde-3-Phosphate Dehydrogenase | Protein Coding | 46 | GC12P016412 | 2.817594 | https://www.genecards.org/cgi-bin/carddisp.pl?gene=GAPDH |
| F3 | Coagulation Factor III, Tissue Factor | Protein Coding | 42 | GC01M094546 | 2.81644 | https://www.genecards.org/cgi-bin/carddisp.pl?gene=F3 |
| MAPK12 | Mitogen-Activated Protein Kinase 12 | Protein Coding | 46 | GC22M053193 | 2.815499 | https://www.genecards.org/cgi-bin/carddisp.pl?gene=MAPK12 |
| H19 | H19 Imprinted Maternally Expressed Transcript | RNA Gene | 27 | GC11M001995 | 2.812749 | https://www.genecards.org/cgi-bin/carddisp.pl?gene=H19 |
| AURKA | Aurora Kinase A | Protein Coding | 48 | GC20M056370 | 2.811463 | https://www.genecards.org/cgi-bin/carddisp.pl?gene=AURKA |
| CCL5 | C-C Motif Chemokine Ligand 5 | Protein Coding | 41 | GC17M035871 | 2.811304 | https://www.genecards.org/cgi-bin/carddisp.pl?gene=CCL5 |
| CCN1 | Cellular Communication Network Factor 1 | Protein Coding | 29 | GC01P085581 | 2.803486 | https://www.genecards.org/cgi-bin/carddisp.pl?gene=CCN1 |
| TM9SF4 | Transmembrane 9 Superfamily Member 4 | Protein Coding | 35 | GC20P032109 | 2.801794 | https://www.genecards.org/cgi-bin/carddisp.pl?gene=TM9SF4 |
| PCNA | Proliferating Cell Nuclear Antigen | Protein Coding | 48 | GC20M005114 | 2.783921 | https://www.genecards.org/cgi-bin/carddisp.pl?gene=PCNA |
| PTK2B | Protein Tyrosine Kinase 2 Beta | Protein Coding | 47 | GC08P027311 | 2.781255 | https://www.genecards.org/cgi-bin/carddisp.pl?gene=PTK2B |
| APEX1 | Apurinic/Apyrimidinic Endodeoxyribonuclease 1 | Protein Coding | 42 | GC14P020455 | 2.779365 | https://www.genecards.org/cgi-bin/carddisp.pl?gene=APEX1 |
| CAD | Carbamoyl-Phosphate Synthetase 2, Aspartate Transcarbamylase, And Dihydroorotase | Protein Coding | 48 | GC02P027217 | 2.765301 | https://www.genecards.org/cgi-bin/carddisp.pl?gene=CAD |
| ERN1 | Endoplasmic Reticulum To Nucleus Signaling 1 | Protein Coding | 43 | GC17M064039 | 2.762794 | https://www.genecards.org/cgi-bin/carddisp.pl?gene=ERN1 |
| APP | Amyloid Beta Precursor Protein | Protein Coding | 48 | GC21M025880 | 2.760781 | https://www.genecards.org/cgi-bin/carddisp.pl?gene=APP |
| SNRPN | Small Nuclear Ribonucleoprotein Polypeptide N | Protein Coding | 43 | GC15P024823 | 2.757516 | https://www.genecards.org/cgi-bin/carddisp.pl?gene=SNRPN |
| OCA2 | OCA2 Melanosomal Transmembrane Protein | Protein Coding | 41 | GC15M027754 | 2.757516 | https://www.genecards.org/cgi-bin/carddisp.pl?gene=OCA2 |
| MKRN3 | Makorin Ring Finger Protein 3 | Protein Coding | 38 | GC15P035913 | 2.757516 | https://www.genecards.org/cgi-bin/carddisp.pl?gene=MKRN3 |
| MAGEL2 | MAGE Family Member L2 | Protein Coding | 35 | GC15M023643 | 2.757516 | https://www.genecards.org/cgi-bin/carddisp.pl?gene=MAGEL2 |
| NPAP1 | Nuclear Pore Associated Protein 1 | Protein Coding | 26 | GC15P024675 | 2.757516 | https://www.genecards.org/cgi-bin/carddisp.pl?gene=NPAP1 |
| PWRN1 | Prader-Willi Region Non-Protein Coding RNA 1 | RNA Gene | 19 | GC15P036237 | 2.757516 | https://www.genecards.org/cgi-bin/carddisp.pl?gene=PWRN1 |
| IPW | Imprinted In Prader-Willi Syndrome | RNA Gene | 15 | GC15P025116 | 2.757516 | https://www.genecards.org/cgi-bin/carddisp.pl?gene=IPW |
| SNORD115-1 | Small Nucleolar RNA, C/D Box 115-1 | RNA Gene | 14 | GC15P035111 | 2.757516 | https://www.genecards.org/cgi-bin/carddisp.pl?gene=SNORD115-1 |
| SNORD116-1 | Small Nucleolar RNA, C/D Box 116-1 | RNA Gene | 14 | GC15P035103 | 2.757516 | https://www.genecards.org/cgi-bin/carddisp.pl?gene=SNORD116-1 |
| PWAR1 | Prader Willi/Angelman Region RNA 1 | RNA Gene | 14 | GC15P025135 | 2.757516 | https://www.genecards.org/cgi-bin/carddisp.pl?gene=PWAR1 |
| MKRN3-AS1 | MKRN3 Antisense RNA 1 | RNA Gene | 6 | GC15U901326 | 2.757516 | https://www.genecards.org/cgi-bin/carddisp.pl?gene=MKRN3-AS1 |
| SNORD116@ | Small Nucleolar RNA, C/D Box 116 Cluster | Gene Cluster | 6 | GC15P025051 | 2.757516 | https://www.genecards.org/cgi-bin/carddisp.pl?gene=SNORD116%40 |
| MHS4 | Malignant Hyperthermia Susceptibility 4 | Genetic Locus | 2 | GC03U990051 | 2.757516 | https://www.genecards.org/cgi-bin/carddisp.pl?gene=MHS4 |
| IGF1R | Insulin Like Growth Factor 1 Receptor | Protein Coding | 52 | GC15P098648 | 2.748327 | https://www.genecards.org/cgi-bin/carddisp.pl?gene=IGF1R |
| TRPC6 | Transient Receptor Potential Cation Channel Subfamily C Member 6 | Protein Coding | 46 | GC11M101451 | 2.745102 | https://www.genecards.org/cgi-bin/carddisp.pl?gene=TRPC6 |
| ACO1 | Aconitase 1 | Protein Coding | 42 | GC09P032374 | 2.743627 | https://www.genecards.org/cgi-bin/carddisp.pl?gene=ACO1 |
| DDIT4L | DNA Damage Inducible Transcript 4 Like | Protein Coding | 35 | GC04M100185 | 2.740173 | https://www.genecards.org/cgi-bin/carddisp.pl?gene=DDIT4L |
| EIF2AK3 | Eukaryotic Translation Initiation Factor 2 Alpha Kinase 3 | Protein Coding | 47 | GC02M088556 | 2.730628 | https://www.genecards.org/cgi-bin/carddisp.pl?gene=EIF2AK3 |
| ADAM17 | ADAM Metallopeptidase Domain 17 | Protein Coding | 48 | GC02M009488 | 2.722687 | https://www.genecards.org/cgi-bin/carddisp.pl?gene=ADAM17 |
| TERT | Telomerase Reverse Transcriptase | Protein Coding | 49 | GC05M001253 | 2.720192 | https://www.genecards.org/cgi-bin/carddisp.pl?gene=TERT |
| PARP1 | Poly(ADP-Ribose) Polymerase 1 | Protein Coding | 47 | GC01M226360 | 2.719892 | https://www.genecards.org/cgi-bin/carddisp.pl?gene=PARP1 |
| PSMD13 | Proteasome 26S Subunit, Non-ATPase 13 | Protein Coding | 38 | GC11P000236 | 2.716498 | https://www.genecards.org/cgi-bin/carddisp.pl?gene=PSMD13 |
| CHCHD2 | Coiled-Coil-Helix-Coiled-Coil-Helix Domain Containing 2 | Protein Coding | 37 | GC07M056101 | 2.712056 | https://www.genecards.org/cgi-bin/carddisp.pl?gene=CHCHD2 |
| CASP8 | Caspase 8 | Protein Coding | 50 | GC02P201233 | 2.69436 | https://www.genecards.org/cgi-bin/carddisp.pl?gene=CASP8 |
| PSMC5 | Proteasome 26S Subunit, ATPase 5 | Protein Coding | 40 | GC17P063827 | 2.691256 | https://www.genecards.org/cgi-bin/carddisp.pl?gene=PSMC5 |
| RAF1 | Raf-1 Proto-Oncogene, Serine/Threonine Kinase | Protein Coding | 51 | GC03M012583 | 2.686998 | https://www.genecards.org/cgi-bin/carddisp.pl?gene=RAF1 |
| ALDOA | Aldolase, Fructose-Bisphosphate A | Protein Coding | 46 | GC16P030064 | 2.684085 | https://www.genecards.org/cgi-bin/carddisp.pl?gene=ALDOA |
| ENO2 | Enolase 2 | Protein Coding | 45 | GC12P006913 | 2.67751 | https://www.genecards.org/cgi-bin/carddisp.pl?gene=ENO2 |
| KAT2B | Lysine Acetyltransferase 2B | Protein Coding | 46 | GC03P020043 | 2.675699 | https://www.genecards.org/cgi-bin/carddisp.pl?gene=KAT2B |
| MUC1 | Mucin 1, Cell Surface Associated | Protein Coding | 46 | GC01M155185 | 2.671762 | https://www.genecards.org/cgi-bin/carddisp.pl?gene=MUC1 |
| UGDH | UDP-Glucose 6-Dehydrogenase | Protein Coding | 44 | GC04M039502 | 2.665574 | https://www.genecards.org/cgi-bin/carddisp.pl?gene=UGDH |
| PRKAB1 | Protein Kinase AMP-Activated Non-Catalytic Subunit Beta 1 | Protein Coding | 45 | GC12P119632 | 2.658883 | https://www.genecards.org/cgi-bin/carddisp.pl?gene=PRKAB1 |
| PFKFB3 | 6-Phosphofructo-2-Kinase/Fructose-2,6-Biphosphatase 3 | Protein Coding | 43 | GC10P006144 | 2.655287 | https://www.genecards.org/cgi-bin/carddisp.pl?gene=PFKFB3 |
| MIR155 | MicroRNA 155 | RNA Gene | 18 | GC21P025573 | 2.653181 | https://www.genecards.org/cgi-bin/carddisp.pl?gene=MIR155 |
| PSMB1 | Proteasome 20S Subunit Beta 1 | Protein Coding | 42 | GC06M170535 | 2.647602 | https://www.genecards.org/cgi-bin/carddisp.pl?gene=PSMB1 |
| POSTN | Periostin | Protein Coding | 41 | GC13M037562 | 2.638323 | https://www.genecards.org/cgi-bin/carddisp.pl?gene=POSTN |
| LEPR | Leptin Receptor | Protein Coding | 47 | GC01P065421 | 2.637357 | https://www.genecards.org/cgi-bin/carddisp.pl?gene=LEPR |
| FOXO4 | Forkhead Box O4 | Protein Coding | 40 | GC0XP071095 | 2.626768 | https://www.genecards.org/cgi-bin/carddisp.pl?gene=FOXO4 |
| TXNIP | Thioredoxin Interacting Protein | Protein Coding | 35 | GC01M145992 | 2.626292 | https://www.genecards.org/cgi-bin/carddisp.pl?gene=TXNIP |
| SUMO1 | Small Ubiquitin Like Modifier 1 | Protein Coding | 43 | GC02M202206 | 2.625155 | https://www.genecards.org/cgi-bin/carddisp.pl?gene=SUMO1 |
| EDNRA | Endothelin Receptor Type A | Protein Coding | 47 | GC04P147480 | 2.622576 | https://www.genecards.org/cgi-bin/carddisp.pl?gene=EDNRA |
| SLC9A1 | Solute Carrier Family 9 Member A1 | Protein Coding | 48 | GC01M027109 | 2.621688 | https://www.genecards.org/cgi-bin/carddisp.pl?gene=SLC9A1 |
| TGFB2 | Transforming Growth Factor Beta 2 | Protein Coding | 48 | GC01P218345 | 2.618666 | https://www.genecards.org/cgi-bin/carddisp.pl?gene=TGFB2 |
| ILK | Integrin Linked Kinase | Protein Coding | 44 | GC11P006604 | 2.615919 | https://www.genecards.org/cgi-bin/carddisp.pl?gene=ILK |
| CIRBP | Cold Inducible RNA Binding Protein | Protein Coding | 36 | GC19P001259 | 2.614792 | https://www.genecards.org/cgi-bin/carddisp.pl?gene=CIRBP |
| TNFSF10 | TNF Superfamily Member 10 | Protein Coding | 43 | GC03M172505 | 2.614678 | https://www.genecards.org/cgi-bin/carddisp.pl?gene=TNFSF10 |
| CDKN1B | Cyclin Dependent Kinase Inhibitor 1B | Protein Coding | 45 | GC12P016655 | 2.611403 | https://www.genecards.org/cgi-bin/carddisp.pl?gene=CDKN1B |
| PECAM1 | Platelet And Endothelial Cell Adhesion Molecule 1 | Protein Coding | 38 | GC17M064319 | 2.611391 | https://www.genecards.org/cgi-bin/carddisp.pl?gene=PECAM1 |
| KCNA5 | Potassium Voltage-Gated Channel Subfamily A Member 5 | Protein Coding | 44 | GC12P005043 | 2.609283 | https://www.genecards.org/cgi-bin/carddisp.pl?gene=KCNA5 |
| NPAS3 | Neuronal PAS Domain Protein 3 | Protein Coding | 38 | GC14P032934 | 2.601408 | https://www.genecards.org/cgi-bin/carddisp.pl?gene=NPAS3 |
| OS9 | OS9 Endoplasmic Reticulum Lectin | Protein Coding | 39 | GC12P057693 | 2.600515 | https://www.genecards.org/cgi-bin/carddisp.pl?gene=OS9 |
| PSMC1 | Proteasome 26S Subunit, ATPase 1 | Protein Coding | 40 | GC14P090256 | 2.600439 | https://www.genecards.org/cgi-bin/carddisp.pl?gene=PSMC1 |
| AIMP1 | Aminoacyl TRNA Synthetase Complex Interacting Multifunctional Protein 1 | Protein Coding | 41 | GC04P106315 | 2.600386 | https://www.genecards.org/cgi-bin/carddisp.pl?gene=AIMP1 |
| CTSB | Cathepsin B | Protein Coding | 48 | GC08M011842 | 2.596596 | https://www.genecards.org/cgi-bin/carddisp.pl?gene=CTSB |
| ESR2 | Estrogen Receptor 2 | Protein Coding | 47 | GC14M064084 | 2.596423 | https://www.genecards.org/cgi-bin/carddisp.pl?gene=ESR2 |
| NDUFA4L2 | NDUFA4 Mitochondrial Complex Associated Like 2 | Protein Coding | 34 | GC12M057234 | 2.593074 | https://www.genecards.org/cgi-bin/carddisp.pl?gene=NDUFA4L2 |
| FASN | Fatty Acid Synthase | Protein Coding | 47 | GC17M082078 | 2.580713 | https://www.genecards.org/cgi-bin/carddisp.pl?gene=FASN |
| DUSP1 | Dual Specificity Phosphatase 1 | Protein Coding | 45 | GC05M172768 | 2.574995 | https://www.genecards.org/cgi-bin/carddisp.pl?gene=DUSP1 |
| NPAS1 | Neuronal PAS Domain Protein 1 | Protein Coding | 32 | GC19P047019 | 2.552993 | https://www.genecards.org/cgi-bin/carddisp.pl?gene=NPAS1 |
| IDH1 | Isocitrate Dehydrogenase (NADP(+)) 1 | Protein Coding | 50 | GC02M208236 | 2.552582 | https://www.genecards.org/cgi-bin/carddisp.pl?gene=IDH1 |
| DIPK2A | Divergent Protein Kinase Domain 2A | Protein Coding | 26 | GC03P143973 | 2.550712 | https://www.genecards.org/cgi-bin/carddisp.pl?gene=DIPK2A |
| EGF | Epidermal Growth Factor | Protein Coding | 48 | GC04P109912 | 2.5494 | https://www.genecards.org/cgi-bin/carddisp.pl?gene=EGF |
| ELAVL1 | ELAV Like RNA Binding Protein 1 | Protein Coding | 40 | GC19M007958 | 2.546901 | https://www.genecards.org/cgi-bin/carddisp.pl?gene=ELAVL1 |
| MIR17 | MicroRNA 17 | RNA Gene | 21 | GC13P091350 | 2.54537 | https://www.genecards.org/cgi-bin/carddisp.pl?gene=MIR17 |
| AK4 | Adenylate Kinase 4 | Protein Coding | 38 | GC01P065147 | 2.544802 | https://www.genecards.org/cgi-bin/carddisp.pl?gene=AK4 |
| KITLG | KIT Ligand | Protein Coding | 42 | GC12M088492 | 2.541453 | https://www.genecards.org/cgi-bin/carddisp.pl?gene=KITLG |
| KCNMA1 | Potassium Calcium-Activated Channel Subfamily M Alpha 1 | Protein Coding | 47 | GC10M076869 | 2.537641 | https://www.genecards.org/cgi-bin/carddisp.pl?gene=KCNMA1 |
| FMN2 | Formin 2 | Protein Coding | 38 | GC01P240014 | 2.53752 | https://www.genecards.org/cgi-bin/carddisp.pl?gene=FMN2 |
| CAV1 | Caveolin 1 | Protein Coding | 46 | GC07P116524 | 2.52985 | https://www.genecards.org/cgi-bin/carddisp.pl?gene=CAV1 |
| STC2 | Stanniocalcin 2 | Protein Coding | 39 | GC05M173314 | 2.52832 | https://www.genecards.org/cgi-bin/carddisp.pl?gene=STC2 |
| LINC-ROR | Long Intergenic Non-Protein Coding RNA, Regulator Of Reprogramming | RNA Gene | 17 | GC18M057054 | 2.523904 | https://www.genecards.org/cgi-bin/carddisp.pl?gene=LINC-ROR |
| TLR2 | Toll Like Receptor 2 | Protein Coding | 48 | GC04P153684 | 2.52354 | https://www.genecards.org/cgi-bin/carddisp.pl?gene=TLR2 |
| RPS6KB1 | Ribosomal Protein S6 Kinase B1 | Protein Coding | 47 | GC17P059893 | 2.516904 | https://www.genecards.org/cgi-bin/carddisp.pl?gene=RPS6KB1 |
| PSMA4 | Proteasome 20S Subunit Alpha 4 | Protein Coding | 42 | GC15P078540 | 2.513219 | https://www.genecards.org/cgi-bin/carddisp.pl?gene=PSMA4 |
| HSPB1 | Heat Shock Protein Family B (Small) Member 1 | Protein Coding | 49 | GC07P076302 | 2.510943 | https://www.genecards.org/cgi-bin/carddisp.pl?gene=HSPB1 |
| FAS | Fas Cell Surface Death Receptor | Protein Coding | 47 | GC10P089158 | 2.499755 | https://www.genecards.org/cgi-bin/carddisp.pl?gene=FAS |
| IFNA1 | Interferon Alpha 1 | Protein Coding | 38 | GC09P021562 | 2.496895 | https://www.genecards.org/cgi-bin/carddisp.pl?gene=IFNA1 |
| VRK2 | VRK Serine/Threonine Kinase 2 | Protein Coding | 40 | GC02P057907 | 2.488752 | https://www.genecards.org/cgi-bin/carddisp.pl?gene=VRK2 |
| SLC16A4 | Solute Carrier Family 16 Member 4 | Protein Coding | 38 | GC01M110362 | 2.483752 | https://www.genecards.org/cgi-bin/carddisp.pl?gene=SLC16A4 |
| PSMC3 | Proteasome 26S Subunit, ATPase 3 | Protein Coding | 42 | GC11M079659 | 2.475733 | https://www.genecards.org/cgi-bin/carddisp.pl?gene=PSMC3 |
| PHLDA2 | Pleckstrin Homology Like Domain Family A Member 2 | Protein Coding | 35 | GC11M002928 | 2.468993 | https://www.genecards.org/cgi-bin/carddisp.pl?gene=PHLDA2 |
| CASR | Calcium Sensing Receptor | Protein Coding | 48 | GC03P122183 | 2.465766 | https://www.genecards.org/cgi-bin/carddisp.pl?gene=CASR |
| IFNB1 | Interferon Beta 1 | Protein Coding | 39 | GC09M021077 | 2.464812 | https://www.genecards.org/cgi-bin/carddisp.pl?gene=IFNB1 |
| RACK1 | Receptor For Activated C Kinase 1 | Protein Coding | 35 | GC05M181534 | 2.460382 | https://www.genecards.org/cgi-bin/carddisp.pl?gene=RACK1 |
| POU5F1 | POU Class 5 Homeobox 1 | Protein Coding | 44 | GC06M056454 | 2.458093 | https://www.genecards.org/cgi-bin/carddisp.pl?gene=POU5F1 |
| MT3 | Metallothionein 3 | Protein Coding | 38 | GC16P056589 | 2.455825 | https://www.genecards.org/cgi-bin/carddisp.pl?gene=MT3 |
| CYP1A1 | Cytochrome P450 Family 1 Subfamily A Member 1 | Protein Coding | 45 | GC15M074719 | 2.453723 | https://www.genecards.org/cgi-bin/carddisp.pl?gene=CYP1A1 |
| PSMD3 | Proteasome 26S Subunit, Non-ATPase 3 | Protein Coding | 40 | GC17P039980 | 2.443001 | https://www.genecards.org/cgi-bin/carddisp.pl?gene=PSMD3 |
| BHLHE41 | Basic Helix-Loop-Helix Family Member E41 | Protein Coding | 39 | GC12M026120 | 2.44105 | https://www.genecards.org/cgi-bin/carddisp.pl?gene=BHLHE41 |
| SOX9 | SRY-Box Transcription Factor 9 | Protein Coding | 45 | GC17P072121 | 2.440288 | https://www.genecards.org/cgi-bin/carddisp.pl?gene=SOX9 |
| GJA1 | Gap Junction Protein Alpha 1 | Protein Coding | 48 | GC06P121436 | 2.438506 | https://www.genecards.org/cgi-bin/carddisp.pl?gene=GJA1 |
| LOC107832851 | SIRT1 Promoter Region | Biological Region | 1 | GC10P067881 | 2.437186 | https://www.genecards.org/cgi-bin/carddisp.pl?gene=LOC107832851 |
| FBXL14 | F-Box And Leucine Rich Repeat Protein 14 | Protein Coding | 31 | GC12M001583 | 2.436692 | https://www.genecards.org/cgi-bin/carddisp.pl?gene=FBXL14 |
| LINC01355 | Long Intergenic Non-Protein Coding RNA 1355 | RNA Gene | 13 | GC01M023281 | 2.427766 | https://www.genecards.org/cgi-bin/carddisp.pl?gene=LINC01355 |
| PSMC4 | Proteasome 26S Subunit, ATPase 4 | Protein Coding | 39 | GC19P044962 | 2.427496 | https://www.genecards.org/cgi-bin/carddisp.pl?gene=PSMC4 |
| VIM | Vimentin | Protein Coding | 47 | GC10P017227 | 2.426753 | https://www.genecards.org/cgi-bin/carddisp.pl?gene=VIM |
| CD274 | CD274 Molecule | Protein Coding | 42 | GC09P005450 | 2.422304 | https://www.genecards.org/cgi-bin/carddisp.pl?gene=CD274 |
| MIR214 | MicroRNA 214 | RNA Gene | 20 | GC01M172234 | 2.421704 | https://www.genecards.org/cgi-bin/carddisp.pl?gene=MIR214 |
| ANGPT4 | Angiopoietin 4 | Protein Coding | 38 | GC20M000869 | 2.417932 | https://www.genecards.org/cgi-bin/carddisp.pl?gene=ANGPT4 |
| SORL1 | Sortilin Related Receptor 1 | Protein Coding | 43 | GC11P121452 | 2.399315 | https://www.genecards.org/cgi-bin/carddisp.pl?gene=SORL1 |
| PSMD2 | Proteasome 26S Subunit Ubiquitin Receptor, Non-ATPase 2 | Protein Coding | 42 | GC03P184298 | 2.391987 | https://www.genecards.org/cgi-bin/carddisp.pl?gene=PSMD2 |
| HDAC2 | Histone Deacetylase 2 | Protein Coding | 48 | GC06M113933 | 2.387294 | https://www.genecards.org/cgi-bin/carddisp.pl?gene=HDAC2 |
| CARD16 | Caspase Recruitment Domain Family Member 16 | Protein Coding | 34 | GC11M105041 | 2.379267 | https://www.genecards.org/cgi-bin/carddisp.pl?gene=CARD16 |
| PSMA5 | Proteasome 20S Subunit Alpha 5 | Protein Coding | 41 | GC01M109399 | 2.37443 | https://www.genecards.org/cgi-bin/carddisp.pl?gene=PSMA5 |
| PSMD14 | Proteasome 26S Subunit, Non-ATPase 14 | Protein Coding | 41 | GC02P161308 | 2.37443 | https://www.genecards.org/cgi-bin/carddisp.pl?gene=PSMD14 |
| PSMD11 | Proteasome 26S Subunit, Non-ATPase 11 | Protein Coding | 39 | GC17P032444 | 2.37443 | https://www.genecards.org/cgi-bin/carddisp.pl?gene=PSMD11 |
| PSMD4 | Proteasome 26S Subunit Ubiquitin Receptor, Non-ATPase 4 | Protein Coding | 42 | GC01P151256 | 2.370819 | https://www.genecards.org/cgi-bin/carddisp.pl?gene=PSMD4 |
| SDHD | Succinate Dehydrogenase Complex Subunit D | Protein Coding | 42 | GC11P112087 | 2.367392 | https://www.genecards.org/cgi-bin/carddisp.pl?gene=SDHD |
| BMP2 | Bone Morphogenetic Protein 2 | Protein Coding | 44 | GC20P006696 | 2.365574 | https://www.genecards.org/cgi-bin/carddisp.pl?gene=BMP2 |
| DEPP1 | DEPP1 Autophagy Regulator | Protein Coding | 23 | GC10M044971 | 2.360084 | https://www.genecards.org/cgi-bin/carddisp.pl?gene=DEPP1 |
| APAF1 | Apoptotic Peptidase Activating Factor 1 | Protein Coding | 44 | GC12P098645 | 2.359442 | https://www.genecards.org/cgi-bin/carddisp.pl?gene=APAF1 |
| PROM1 | Prominin 1 | Protein Coding | 43 | GC04M015965 | 2.359262 | https://www.genecards.org/cgi-bin/carddisp.pl?gene=PROM1 |
| HIF1AP1 | HIF1AP Pseudogene 1 | Pseudogene | 6 | GC14M075116 | 2.357205 | https://www.genecards.org/cgi-bin/carddisp.pl?gene=HIF1AP1 |
| POR | Cytochrome P450 Oxidoreductase | Protein Coding | 47 | GC07P075899 | 2.35701 | https://www.genecards.org/cgi-bin/carddisp.pl?gene=POR |
| SLC11A2 | Solute Carrier Family 11 Member 2 | Protein Coding | 45 | GC12M050952 | 2.356753 | https://www.genecards.org/cgi-bin/carddisp.pl?gene=SLC11A2 |
| SLC29A1 | Solute Carrier Family 29 Member 1 (Augustine Blood Group) | Protein Coding | 46 | GC06P044219 | 2.356164 | https://www.genecards.org/cgi-bin/carddisp.pl?gene=SLC29A1 |
| MCL1 | MCL1 Apoptosis Regulator, BCL2 Family Member | Protein Coding | 44 | GC01M150561 | 2.350431 | https://www.genecards.org/cgi-bin/carddisp.pl?gene=MCL1 |
| MVP | Major Vault Protein | Protein Coding | 39 | GC16P037734 | 2.349076 | https://www.genecards.org/cgi-bin/carddisp.pl?gene=MVP |
| KCNJ11 | Potassium Inwardly Rectifying Channel Subfamily J Member 11 | Protein Coding | 46 | GC11M017437 | 2.348992 | https://www.genecards.org/cgi-bin/carddisp.pl?gene=KCNJ11 |
| YAP1 | Yes1 Associated Transcriptional Regulator | Protein Coding | 46 | GC11P102110 | 2.347953 | https://www.genecards.org/cgi-bin/carddisp.pl?gene=YAP1 |
| PRDX1 | Peroxiredoxin 1 | Protein Coding | 46 | GC01M045511 | 2.34146 | https://www.genecards.org/cgi-bin/carddisp.pl?gene=PRDX1 |
| AGER | Advanced Glycosylation End-Product Specific Receptor | Protein Coding | 42 | GC06M032180 | 2.336048 | https://www.genecards.org/cgi-bin/carddisp.pl?gene=AGER |
| PSMD1 | Proteasome 26S Subunit, Non-ATPase 1 | Protein Coding | 38 | GC02P231056 | 2.334614 | https://www.genecards.org/cgi-bin/carddisp.pl?gene=PSMD1 |
| HIF1A-AS2 | HIF1A Antisense RNA 2 | RNA Gene | 15 | GC14M061747 | 2.329239 | https://www.genecards.org/cgi-bin/carddisp.pl?gene=HIF1A-AS2 |
| CXCL10 | C-X-C Motif Chemokine Ligand 10 | Protein Coding | 42 | GC04M076021 | 2.31902 | https://www.genecards.org/cgi-bin/carddisp.pl?gene=CXCL10 |
| LOC111589215 | BRCA1 Promoter Region | Biological Region | 1 | GC17P048374 | 2.313751 | https://www.genecards.org/cgi-bin/carddisp.pl?gene=LOC111589215 |
| EHMT2 | Euchromatic Histone Lysine Methyltransferase 2 | Protein Coding | 43 | GC06M031879 | 2.309749 | https://www.genecards.org/cgi-bin/carddisp.pl?gene=EHMT2 |
| SIRT6 | Sirtuin 6 | Protein Coding | 43 | GC19M004174 | 2.309203 | https://www.genecards.org/cgi-bin/carddisp.pl?gene=SIRT6 |
| CEBPA | CCAAT Enhancer Binding Protein Alpha | Protein Coding | 45 | GC19M033299 | 2.308295 | https://www.genecards.org/cgi-bin/carddisp.pl?gene=CEBPA |
| NQO1 | NAD(P)H Quinone Dehydrogenase 1 | Protein Coding | 46 | GC16M069706 | 2.303932 | https://www.genecards.org/cgi-bin/carddisp.pl?gene=NQO1 |
| PSMB5 | Proteasome 20S Subunit Beta 5 | Protein Coding | 42 | GC14M023016 | 2.301449 | https://www.genecards.org/cgi-bin/carddisp.pl?gene=PSMB5 |
| HPSE | Heparanase | Protein Coding | 42 | GC04M083292 | 2.300862 | https://www.genecards.org/cgi-bin/carddisp.pl?gene=HPSE |
| HAMP | Hepcidin Antimicrobial Peptide | Protein Coding | 42 | GC19P044720 | 2.298669 | https://www.genecards.org/cgi-bin/carddisp.pl?gene=HAMP |
| P4HB | Prolyl 4-Hydroxylase Subunit Beta | Protein Coding | 46 | GC17M081843 | 2.298466 | https://www.genecards.org/cgi-bin/carddisp.pl?gene=P4HB |
| E2F1 | E2F Transcription Factor 1 | Protein Coding | 42 | GC20M033675 | 2.291383 | https://www.genecards.org/cgi-bin/carddisp.pl?gene=E2F1 |
| ARG2 | Arginase 2 | Protein Coding | 43 | GC14P067619 | 2.290464 | https://www.genecards.org/cgi-bin/carddisp.pl?gene=ARG2 |
| UBE2T | Ubiquitin Conjugating Enzyme E2 T | Protein Coding | 41 | GC01M202332 | 2.289065 | https://www.genecards.org/cgi-bin/carddisp.pl?gene=UBE2T |
| PSMA6 | Proteasome 20S Subunit Alpha 6 | Protein Coding | 44 | GC14P035278 | 2.284589 | https://www.genecards.org/cgi-bin/carddisp.pl?gene=PSMA6 |
| PSMB6 | Proteasome 20S Subunit Beta 6 | Protein Coding | 39 | GC17P004796 | 2.284589 | https://www.genecards.org/cgi-bin/carddisp.pl?gene=PSMB6 |
| MAPK10 | Mitogen-Activated Protein Kinase 10 | Protein Coding | 48 | GC04M085990 | 2.279369 | https://www.genecards.org/cgi-bin/carddisp.pl?gene=MAPK10 |
| FOXM1 | Forkhead Box M1 | Protein Coding | 42 | GC12M002857 | 2.273062 | https://www.genecards.org/cgi-bin/carddisp.pl?gene=FOXM1 |
| FLT4 | Fms Related Receptor Tyrosine Kinase 4 | Protein Coding | 49 | GC05M180607 | 2.265313 | https://www.genecards.org/cgi-bin/carddisp.pl?gene=FLT4 |
| PRKN | Parkin RBR E3 Ubiquitin Protein Ligase | Protein Coding | 37 | GC06M161348 | 2.255014 | https://www.genecards.org/cgi-bin/carddisp.pl?gene=PRKN |
| GATA2 | GATA Binding Protein 2 | Protein Coding | 46 | GC03M128479 | 2.251574 | https://www.genecards.org/cgi-bin/carddisp.pl?gene=GATA2 |
| AJUBA | Ajuba LIM Protein | Protein Coding | 35 | GC14M022971 | 2.249679 | https://www.genecards.org/cgi-bin/carddisp.pl?gene=AJUBA |
| UBE2D2 | Ubiquitin Conjugating Enzyme E2 D2 | Protein Coding | 42 | GC05P139526 | 2.245862 | https://www.genecards.org/cgi-bin/carddisp.pl?gene=UBE2D2 |
| CEMP1 | Cementum Protein 1 | Protein Coding | 27 | GC16M005520 | 2.241318 | https://www.genecards.org/cgi-bin/carddisp.pl?gene=CEMP1 |
| PIK3R1 | Phosphoinositide-3-Kinase Regulatory Subunit 1 | Protein Coding | 48 | GC05P068215 | 2.240723 | https://www.genecards.org/cgi-bin/carddisp.pl?gene=PIK3R1 |
| TUFT1 | Tuftelin 1 | Protein Coding | 38 | GC01P151513 | 2.239079 | https://www.genecards.org/cgi-bin/carddisp.pl?gene=TUFT1 |
| NGF | Nerve Growth Factor | Protein Coding | 47 | GC01M115285 | 2.23692 | https://www.genecards.org/cgi-bin/carddisp.pl?gene=NGF |
| UCP2 | Uncoupling Protein 2 | Protein Coding | 44 | GC11M073974 | 2.230703 | https://www.genecards.org/cgi-bin/carddisp.pl?gene=UCP2 |
| TET1 | Tet Methylcytosine Dioxygenase 1 | Protein Coding | 36 | GC10P068560 | 2.223847 | https://www.genecards.org/cgi-bin/carddisp.pl?gene=TET1 |
| GPI | Glucose-6-Phosphate Isomerase | Protein Coding | 44 | GC19P034360 | 2.221459 | https://www.genecards.org/cgi-bin/carddisp.pl?gene=GPI |
| ABCG2 | ATP Binding Cassette Subfamily G Member 2 (Junior Blood Group) | Protein Coding | 48 | GC04M088090 | 2.220474 | https://www.genecards.org/cgi-bin/carddisp.pl?gene=ABCG2 |
| ELK1 | ETS Transcription Factor ELK1 | Protein Coding | 42 | GC0XM047635 | 2.217265 | https://www.genecards.org/cgi-bin/carddisp.pl?gene=ELK1 |
| MTA1 | Metastasis Associated 1 | Protein Coding | 41 | GC14P105419 | 2.207824 | https://www.genecards.org/cgi-bin/carddisp.pl?gene=MTA1 |
| AIFM1 | Apoptosis Inducing Factor Mitochondria Associated 1 | Protein Coding | 46 | GC0XM130129 | 2.206393 | https://www.genecards.org/cgi-bin/carddisp.pl?gene=AIFM1 |
| UBE2D3 | Ubiquitin Conjugating Enzyme E2 D3 | Protein Coding | 44 | GC04M102794 | 2.202357 | https://www.genecards.org/cgi-bin/carddisp.pl?gene=UBE2D3 |
| CTBP1 | C-Terminal Binding Protein 1 | Protein Coding | 46 | GC04M001211 | 2.201222 | https://www.genecards.org/cgi-bin/carddisp.pl?gene=CTBP1 |
| HSPA8 | Heat Shock Protein Family A (Hsp70) Member 8 | Protein Coding | 45 | GC11M123057 | 2.200404 | https://www.genecards.org/cgi-bin/carddisp.pl?gene=HSPA8 |
| MIR34A | MicroRNA 34a | RNA Gene | 22 | GC01M009151 | 2.192262 | https://www.genecards.org/cgi-bin/carddisp.pl?gene=MIR34A |
| SDHC | Succinate Dehydrogenase Complex Subunit C | Protein Coding | 43 | GC01P161314 | 2.190789 | https://www.genecards.org/cgi-bin/carddisp.pl?gene=SDHC |
| ATG9B | Autophagy Related 9B | Protein Coding | 34 | GC07M151012 | 2.186985 | https://www.genecards.org/cgi-bin/carddisp.pl?gene=ATG9B |
| LOC110386947 | CYP19A1 Promoter I.1 | Biological Region | 1 | GC15P051714 | 2.186081 | https://www.genecards.org/cgi-bin/carddisp.pl?gene=LOC110386947 |
| IRF3 | Interferon Regulatory Factor 3 | Protein Coding | 44 | GC19M049659 | 2.175197 | https://www.genecards.org/cgi-bin/carddisp.pl?gene=IRF3 |
| CA12 | Carbonic Anhydrase 12 | Protein Coding | 44 | GC15M063321 | 2.169284 | https://www.genecards.org/cgi-bin/carddisp.pl?gene=CA12 |
| TXN2 | Thioredoxin 2 | Protein Coding | 43 | GC22M036467 | 2.161202 | https://www.genecards.org/cgi-bin/carddisp.pl?gene=TXN2 |
| BRCA1 | BRCA1 DNA Repair Associated | Protein Coding | 48 | GC17M043044 | 2.156832 | https://www.genecards.org/cgi-bin/carddisp.pl?gene=BRCA1 |
| CP | Ceruloplasmin | Protein Coding | 46 | GC03M149162 | 2.155941 | https://www.genecards.org/cgi-bin/carddisp.pl?gene=CP |
| IL1A | Interleukin 1 Alpha | Protein Coding | 41 | GC02M112773 | 2.152053 | https://www.genecards.org/cgi-bin/carddisp.pl?gene=IL1A |
| CD34 | CD34 Molecule | Protein Coding | 40 | GC01M207880 | 2.148513 | https://www.genecards.org/cgi-bin/carddisp.pl?gene=CD34 |
| PFKL | Phosphofructokinase, Liver Type | Protein Coding | 43 | GC21P044300 | 2.146549 | https://www.genecards.org/cgi-bin/carddisp.pl?gene=PFKL |
| CDKN3 | Cyclin Dependent Kinase Inhibitor 3 | Protein Coding | 40 | GC14P054398 | 2.143115 | https://www.genecards.org/cgi-bin/carddisp.pl?gene=CDKN3 |
| BCL2L11 | BCL2 Like 11 | Protein Coding | 42 | GC02P111119 | 2.126715 | https://www.genecards.org/cgi-bin/carddisp.pl?gene=BCL2L11 |
| CCR7 | C-C Motif Chemokine Receptor 7 | Protein Coding | 43 | GC17M040556 | 2.124369 | https://www.genecards.org/cgi-bin/carddisp.pl?gene=CCR7 |
| MIR145 | MicroRNA 145 | RNA Gene | 21 | GC05P149430 | 2.1229 | https://www.genecards.org/cgi-bin/carddisp.pl?gene=MIR145 |
| ALAS2 | 5'-Aminolevulinate Synthase 2 | Protein Coding | 43 | GC0XM055009 | 2.121696 | https://www.genecards.org/cgi-bin/carddisp.pl?gene=ALAS2 |
| PRKG1 | Protein Kinase CGMP-Dependent 1 | Protein Coding | 48 | GC10P050991 | 2.121661 | https://www.genecards.org/cgi-bin/carddisp.pl?gene=PRKG1 |
| SMAD4 | SMAD Family Member 4 | Protein Coding | 48 | GC18P051028 | 2.118133 | https://www.genecards.org/cgi-bin/carddisp.pl?gene=SMAD4 |
| BID | BH3 Interacting Domain Death Agonist | Protein Coding | 43 | GC22M017734 | 2.116937 | https://www.genecards.org/cgi-bin/carddisp.pl?gene=BID |
| LGALS3 | Galectin 3 | Protein Coding | 42 | GC14P055124 | 2.116371 | https://www.genecards.org/cgi-bin/carddisp.pl?gene=LGALS3 |
| MIR199A1 | MicroRNA 199a-1 | RNA Gene | 18 | GC19M010817 | 2.110257 | https://www.genecards.org/cgi-bin/carddisp.pl?gene=MIR199A1 |
| OXT | Oxytocin/Neurophysin I Prepropeptide | Protein Coding | 38 | GC20P003068 | 2.107114 | https://www.genecards.org/cgi-bin/carddisp.pl?gene=OXT |
| MIR20A | MicroRNA 20a | RNA Gene | 19 | GC13P091506 | 2.106834 | https://www.genecards.org/cgi-bin/carddisp.pl?gene=MIR20A |
| MIR126 | MicroRNA 126 | RNA Gene | 22 | GC09P136670 | 2.106083 | https://www.genecards.org/cgi-bin/carddisp.pl?gene=MIR126 |
| TSC2 | TSC Complex Subunit 2 | Protein Coding | 47 | GC16P008040 | 2.105459 | https://www.genecards.org/cgi-bin/carddisp.pl?gene=TSC2 |
| MKI67 | Marker Of Proliferation Ki-67 | Protein Coding | 43 | GC10M128096 | 2.101211 | https://www.genecards.org/cgi-bin/carddisp.pl?gene=MKI67 |
| AGT | Angiotensinogen | Protein Coding | 47 | GC01M230702 | 2.099175 | https://www.genecards.org/cgi-bin/carddisp.pl?gene=AGT |
| GATA6 | GATA Binding Protein 6 | Protein Coding | 46 | GC18P022169 | 2.097404 | https://www.genecards.org/cgi-bin/carddisp.pl?gene=GATA6 |
| SPHK1 | Sphingosine Kinase 1 | Protein Coding | 45 | GC17P076376 | 2.095792 | https://www.genecards.org/cgi-bin/carddisp.pl?gene=SPHK1 |
| ROCK2 | Rho Associated Coiled-Coil Containing Protein Kinase 2 | Protein Coding | 45 | GC02M011276 | 2.09506 | https://www.genecards.org/cgi-bin/carddisp.pl?gene=ROCK2 |
| BACH1 | BTB Domain And CNC Homolog 1 | Protein Coding | 39 | GC21P029194 | 2.094181 | https://www.genecards.org/cgi-bin/carddisp.pl?gene=BACH1 |
| PMAIP1 | Phorbol-12-Myristate-13-Acetate-Induced Protein 1 | Protein Coding | 38 | GC18P059899 | 2.091819 | https://www.genecards.org/cgi-bin/carddisp.pl?gene=PMAIP1 |
| PRKCD | Protein Kinase C Delta | Protein Coding | 51 | GC03P053156 | 2.091445 | https://www.genecards.org/cgi-bin/carddisp.pl?gene=PRKCD |
| CTSL | Cathepsin L | Protein Coding | 44 | GC09P087725 | 2.09123 | https://www.genecards.org/cgi-bin/carddisp.pl?gene=CTSL |
| STAT5B | Signal Transducer And Activator Of Transcription 5B | Protein Coding | 47 | GC17M042199 | 2.08878 | https://www.genecards.org/cgi-bin/carddisp.pl?gene=STAT5B |
| BMP6 | Bone Morphogenetic Protein 6 | Protein Coding | 41 | GC06P007726 | 2.086342 | https://www.genecards.org/cgi-bin/carddisp.pl?gene=BMP6 |
| RBM3 | RNA Binding Motif Protein 3 | Protein Coding | 36 | GC0XP048574 | 2.084678 | https://www.genecards.org/cgi-bin/carddisp.pl?gene=RBM3 |
| LGALS1 | Galectin 1 | Protein Coding | 41 | GC22P037675 | 2.084226 | https://www.genecards.org/cgi-bin/carddisp.pl?gene=LGALS1 |
| WDR26 | WD Repeat Domain 26 | Protein Coding | 39 | GC01M224385 | 2.083879 | https://www.genecards.org/cgi-bin/carddisp.pl?gene=WDR26 |
| RAMP2 | Receptor Activity Modifying Protein 2 | Protein Coding | 40 | GC17P042758 | 2.079008 | https://www.genecards.org/cgi-bin/carddisp.pl?gene=RAMP2 |
| GSK3B | Glycogen Synthase Kinase 3 Beta | Protein Coding | 47 | GC03M119821 | 2.075107 | https://www.genecards.org/cgi-bin/carddisp.pl?gene=GSK3B |
| SMAD2 | SMAD Family Member 2 | Protein Coding | 46 | GC18M047809 | 2.07202 | https://www.genecards.org/cgi-bin/carddisp.pl?gene=SMAD2 |
| POMC | Proopiomelanocortin | Protein Coding | 45 | GC02M025160 | 2.070915 | https://www.genecards.org/cgi-bin/carddisp.pl?gene=POMC |
| RAB4B-EGLN2 | RAB4B-EGLN2 Readthrough (NMD Candidate) | RNA Gene | 14 | GC19P040778 | 2.069268 | https://www.genecards.org/cgi-bin/carddisp.pl?gene=RAB4B-EGLN2 |
| PRNP | Prion Protein | Protein Coding | 44 | GC20P004686 | 2.069219 | https://www.genecards.org/cgi-bin/carddisp.pl?gene=PRNP |
| IREB2 | Iron Responsive Element Binding Protein 2 | Protein Coding | 43 | GC15P078437 | 2.06911 | https://www.genecards.org/cgi-bin/carddisp.pl?gene=IREB2 |
| MECP2 | Methyl-CpG Binding Protein 2 | Protein Coding | 43 | GC0XM154021 | 2.068136 | https://www.genecards.org/cgi-bin/carddisp.pl?gene=MECP2 |
| DNM1L | Dynamin 1 Like | Protein Coding | 45 | GC12P032679 | 2.063782 | https://www.genecards.org/cgi-bin/carddisp.pl?gene=DNM1L |
| APOLD1 | Apolipoprotein L Domain Containing 1 | Protein Coding | 32 | GC12P012725 | 2.062705 | https://www.genecards.org/cgi-bin/carddisp.pl?gene=APOLD1 |
| SRF | Serum Response Factor | Protein Coding | 39 | GC06P043171 | 2.061151 | https://www.genecards.org/cgi-bin/carddisp.pl?gene=SRF |
| SLC38A1 | Solute Carrier Family 38 Member 1 | Protein Coding | 40 | GC12M046183 | 2.059483 | https://www.genecards.org/cgi-bin/carddisp.pl?gene=SLC38A1 |
| NOX1 | NADPH Oxidase 1 | Protein Coding | 40 | GC0XM100843 | 2.058232 | https://www.genecards.org/cgi-bin/carddisp.pl?gene=NOX1 |
| KRAS | KRAS Proto-Oncogene, GTPase | Protein Coding | 48 | GC12M025204 | 2.05382 | https://www.genecards.org/cgi-bin/carddisp.pl?gene=KRAS |
| CYBB | Cytochrome B-245 Beta Chain | Protein Coding | 44 | GC0XP037780 | 2.051008 | https://www.genecards.org/cgi-bin/carddisp.pl?gene=CYBB |
| HLA-G | Major Histocompatibility Complex, Class I, G | Protein Coding | 42 | GC06P070094 | 2.049037 | https://www.genecards.org/cgi-bin/carddisp.pl?gene=HLA-G |
| MTF1 | Metal Regulatory Transcription Factor 1 | Protein Coding | 40 | GC01M037810 | 2.048877 | https://www.genecards.org/cgi-bin/carddisp.pl?gene=MTF1 |
| STK25 | Serine/Threonine Kinase 25 | Protein Coding | 39 | GC02M241492 | 2.047875 | https://www.genecards.org/cgi-bin/carddisp.pl?gene=STK25 |
| DDX58 | DExD/H-Box Helicase 58 | Protein Coding | 44 | GC09M032455 | 2.047021 | https://www.genecards.org/cgi-bin/carddisp.pl?gene=DDX58 |
| ADORA2A | Adenosine A2a Receptor | Protein Coding | 44 | GC22P024417 | 2.046466 | https://www.genecards.org/cgi-bin/carddisp.pl?gene=ADORA2A |
| ABCC8 | ATP Binding Cassette Subfamily C Member 8 | Protein Coding | 44 | GC11M017392 | 2.045436 | https://www.genecards.org/cgi-bin/carddisp.pl?gene=ABCC8 |
| WTIP | WT1 Interacting Protein | Protein Coding | 33 | GC19P034481 | 2.04463 | https://www.genecards.org/cgi-bin/carddisp.pl?gene=WTIP |
| SERPINB5 | Serpin Family B Member 5 | Protein Coding | 40 | GC18P063476 | 2.042528 | https://www.genecards.org/cgi-bin/carddisp.pl?gene=SERPINB5 |
| CKB | Creatine Kinase B | Protein Coding | 43 | GC14M103519 | 2.041214 | https://www.genecards.org/cgi-bin/carddisp.pl?gene=CKB |
| STAT1 | Signal Transducer And Activator Of Transcription 1 | Protein Coding | 50 | GC02M190908 | 2.040866 | https://www.genecards.org/cgi-bin/carddisp.pl?gene=STAT1 |
| UBE2I | Ubiquitin Conjugating Enzyme E2 I | Protein Coding | 45 | GC16P007982 | 2.040793 | https://www.genecards.org/cgi-bin/carddisp.pl?gene=UBE2I |
| TRAF3 | TNF Receptor Associated Factor 3 | Protein Coding | 45 | GC14P107912 | 2.040216 | https://www.genecards.org/cgi-bin/carddisp.pl?gene=TRAF3 |
| OPRD1 | Opioid Receptor Delta 1 | Protein Coding | 42 | GC01P028812 | 2.038893 | https://www.genecards.org/cgi-bin/carddisp.pl?gene=OPRD1 |
| HBEGF | Heparin Binding EGF Like Growth Factor | Protein Coding | 40 | GC05M140332 | 2.036453 | https://www.genecards.org/cgi-bin/carddisp.pl?gene=HBEGF |
| TXNDC5 | Thioredoxin Domain Containing 5 | Protein Coding | 36 | GC06M007893 | 2.033114 | https://www.genecards.org/cgi-bin/carddisp.pl?gene=TXNDC5 |
| PRKDC | Protein Kinase, DNA-Activated, Catalytic Subunit | Protein Coding | 47 | GC08M047773 | 2.030743 | https://www.genecards.org/cgi-bin/carddisp.pl?gene=PRKDC |
| ROBO4 | Roundabout Guidance Receptor 4 | Protein Coding | 40 | GC11M124883 | 2.027367 | https://www.genecards.org/cgi-bin/carddisp.pl?gene=ROBO4 |
| SELE | Selectin E | Protein Coding | 41 | GC01M169722 | 2.026412 | https://www.genecards.org/cgi-bin/carddisp.pl?gene=SELE |
| STUB1 | STIP1 Homology And U-Box Containing Protein 1 | Protein Coding | 43 | GC16P007975 | 2.025384 | https://www.genecards.org/cgi-bin/carddisp.pl?gene=STUB1 |
| NDRG2 | NDRG Family Member 2 | Protein Coding | 36 | GC14M021016 | 2.019799 | https://www.genecards.org/cgi-bin/carddisp.pl?gene=NDRG2 |
| ID2 | Inhibitor Of DNA Binding 2 | Protein Coding | 42 | GC02P008678 | 2.018492 | https://www.genecards.org/cgi-bin/carddisp.pl?gene=ID2 |
| ABCC1 | ATP Binding Cassette Subfamily C Member 1 | Protein Coding | 47 | GC16P015949 | 2.017437 | https://www.genecards.org/cgi-bin/carddisp.pl?gene=ABCC1 |
| DAXX | Death Domain Associated Protein | Protein Coding | 42 | GC06M033318 | 2.016431 | https://www.genecards.org/cgi-bin/carddisp.pl?gene=DAXX |
| HSPD1 | Heat Shock Protein Family D (Hsp60) Member 1 | Protein Coding | 45 | GC02M197486 | 2.015325 | https://www.genecards.org/cgi-bin/carddisp.pl?gene=HSPD1 |
| DDAH1 | Dimethylarginine Dimethylaminohydrolase 1 | Protein Coding | 41 | GC01M085318 | 2.012722 | https://www.genecards.org/cgi-bin/carddisp.pl?gene=DDAH1 |
| DLK1 | Delta Like Non-Canonical Notch Ligand 1 | Protein Coding | 42 | GC14P107984 | 2.008295 | https://www.genecards.org/cgi-bin/carddisp.pl?gene=DLK1 |
| BRAF | B-Raf Proto-Oncogene, Serine/Threonine Kinase | Protein Coding | 51 | GC07M140726 | 1.998297 | https://www.genecards.org/cgi-bin/carddisp.pl?gene=BRAF |
| CAPN2 | Calpain 2 | Protein Coding | 45 | GC01P223701 | 1.997195 | https://www.genecards.org/cgi-bin/carddisp.pl?gene=CAPN2 |
| RUNX2 | RUNX Family Transcription Factor 2 | Protein Coding | 46 | GC06P070369 | 1.996891 | https://www.genecards.org/cgi-bin/carddisp.pl?gene=RUNX2 |
| PROK1 | Prokineticin 1 | Protein Coding | 34 | GC01P110451 | 1.994058 | https://www.genecards.org/cgi-bin/carddisp.pl?gene=PROK1 |
| MITF | Melanocyte Inducing Transcription Factor | Protein Coding | 46 | GC03P069788 | 1.993352 | https://www.genecards.org/cgi-bin/carddisp.pl?gene=MITF |
| ATP7A | ATPase Copper Transporting Alpha | Protein Coding | 44 | GC0XP077960 | 1.989318 | https://www.genecards.org/cgi-bin/carddisp.pl?gene=ATP7A |
| KCNK2 | Potassium Two Pore Domain Channel Subfamily K Member 2 | Protein Coding | 38 | GC01P215005 | 1.986192 | https://www.genecards.org/cgi-bin/carddisp.pl?gene=KCNK2 |
| SQSTM1 | Sequestosome 1 | Protein Coding | 46 | GC05P179806 | 1.975608 | https://www.genecards.org/cgi-bin/carddisp.pl?gene=SQSTM1 |
| CCNA2 | Cyclin A2 | Protein Coding | 42 | GC04M121816 | 1.971488 | https://www.genecards.org/cgi-bin/carddisp.pl?gene=CCNA2 |
| NPY | Neuropeptide Y | Protein Coding | 43 | GC07P024290 | 1.969071 | https://www.genecards.org/cgi-bin/carddisp.pl?gene=NPY |
| LRP1 | LDL Receptor Related Protein 1 | Protein Coding | 45 | GC12P057128 | 1.967048 | https://www.genecards.org/cgi-bin/carddisp.pl?gene=LRP1 |
| SFTPB | Surfactant Protein B | Protein Coding | 42 | GC02M085657 | 1.962455 | https://www.genecards.org/cgi-bin/carddisp.pl?gene=SFTPB |
| HSP90AB1 | Heat Shock Protein 90 Alpha Family Class B Member 1 | Protein Coding | 44 | GC06P044246 | 1.960973 | https://www.genecards.org/cgi-bin/carddisp.pl?gene=HSP90AB1 |
| CFLAR | CASP8 And FADD Like Apoptosis Regulator | Protein Coding | 43 | GC02P201117 | 1.956234 | https://www.genecards.org/cgi-bin/carddisp.pl?gene=CFLAR |
| MTDH | Metadherin | Protein Coding | 39 | GC08P097643 | 1.955656 | https://www.genecards.org/cgi-bin/carddisp.pl?gene=MTDH |
| TERC | Telomerase RNA Component | RNA Gene | 27 | GC03M169765 | 1.953491 | https://www.genecards.org/cgi-bin/carddisp.pl?gene=TERC |
| TF | Transferrin | Protein Coding | 46 | GC03P133666 | 1.950361 | https://www.genecards.org/cgi-bin/carddisp.pl?gene=TF |
| HOTAIR | HOX Transcript Antisense RNA | RNA Gene | 25 | GC12M053962 | 1.949738 | https://www.genecards.org/cgi-bin/carddisp.pl?gene=HOTAIR |
| PTPA | Protein Phosphatase 2 Phosphatase Activator | Protein Coding | 33 | GC09P129111 | 1.94929 | https://www.genecards.org/cgi-bin/carddisp.pl?gene=PTPA |
| IKBKB | Inhibitor Of Nuclear Factor Kappa B Kinase Subunit Beta | Protein Coding | 50 | GC08P042271 | 1.947777 | https://www.genecards.org/cgi-bin/carddisp.pl?gene=IKBKB |
| ASCL2 | Achaete-Scute Family BHLH Transcription Factor 2 | Protein Coding | 36 | GC11M002274 | 1.94688 | https://www.genecards.org/cgi-bin/carddisp.pl?gene=ASCL2 |
| LCK | LCK Proto-Oncogene, Src Family Tyrosine Kinase | Protein Coding | 50 | GC01P032251 | 1.944429 | https://www.genecards.org/cgi-bin/carddisp.pl?gene=LCK |
| TUBB3 | Tubulin Beta 3 Class III | Protein Coding | 46 | GC16P090783 | 1.943321 | https://www.genecards.org/cgi-bin/carddisp.pl?gene=TUBB3 |
| NR4A1 | Nuclear Receptor Subfamily 4 Group A Member 1 | Protein Coding | 44 | GC12P052022 | 1.937572 | https://www.genecards.org/cgi-bin/carddisp.pl?gene=NR4A1 |
| VWF | Von Willebrand Factor | Protein Coding | 46 | GC12M005917 | 1.935508 | https://www.genecards.org/cgi-bin/carddisp.pl?gene=VWF |
| CALCRL | Calcitonin Receptor Like Receptor | Protein Coding | 43 | GC02M187341 | 1.933038 | https://www.genecards.org/cgi-bin/carddisp.pl?gene=CALCRL |
| MDM4 | MDM4 Regulator Of P53 | Protein Coding | 42 | GC01P204516 | 1.932485 | https://www.genecards.org/cgi-bin/carddisp.pl?gene=MDM4 |
| RYR1 | Ryanodine Receptor 1 | Protein Coding | 44 | GC19P044882 | 1.929678 | https://www.genecards.org/cgi-bin/carddisp.pl?gene=RYR1 |
| ADGRD1 | Adhesion G Protein-Coupled Receptor D1 | Protein Coding | 34 | GC12P130953 | 1.928936 | https://www.genecards.org/cgi-bin/carddisp.pl?gene=ADGRD1 |
| HSPA1A | Heat Shock Protein Family A (Hsp70) Member 1A | Protein Coding | 42 | GC06P070160 | 1.928329 | https://www.genecards.org/cgi-bin/carddisp.pl?gene=HSPA1A |
| PLAT | Plasminogen Activator, Tissue Type | Protein Coding | 46 | GC08M042174 | 1.928239 | https://www.genecards.org/cgi-bin/carddisp.pl?gene=PLAT |
| TNFRSF10B | TNF Receptor Superfamily Member 10b | Protein Coding | 48 | GC08M023020 | 1.927714 | https://www.genecards.org/cgi-bin/carddisp.pl?gene=TNFRSF10B |
| RPS6 | Ribosomal Protein S6 | Protein Coding | 42 | GC09M019375 | 1.922947 | https://www.genecards.org/cgi-bin/carddisp.pl?gene=RPS6 |
| SNAI2 | Snail Family Transcriptional Repressor 2 | Protein Coding | 42 | GC08M048917 | 1.917892 | https://www.genecards.org/cgi-bin/carddisp.pl?gene=SNAI2 |
| HDAC4 | Histone Deacetylase 4 | Protein Coding | 49 | GC02M239048 | 1.915036 | https://www.genecards.org/cgi-bin/carddisp.pl?gene=HDAC4 |
| PSMB4 | Proteasome 20S Subunit Beta 4 | Protein Coding | 43 | GC01P151372 | 1.91343 | https://www.genecards.org/cgi-bin/carddisp.pl?gene=PSMB4 |
| JAK2 | Janus Kinase 2 | Protein Coding | 50 | GC09P004985 | 1.91113 | https://www.genecards.org/cgi-bin/carddisp.pl?gene=JAK2 |
| PTBP1 | Polypyrimidine Tract Binding Protein 1 | Protein Coding | 41 | GC19P000797 | 1.909424 | https://www.genecards.org/cgi-bin/carddisp.pl?gene=PTBP1 |
| HTR2B | 5-Hydroxytryptamine Receptor 2B | Protein Coding | 41 | GC02M231108 | 1.909343 | https://www.genecards.org/cgi-bin/carddisp.pl?gene=HTR2B |
| HSD11B2 | Hydroxysteroid 11-Beta Dehydrogenase 2 | Protein Coding | 44 | GC16P067433 | 1.906295 | https://www.genecards.org/cgi-bin/carddisp.pl?gene=HSD11B2 |
| ZFP36L1 | ZFP36 Ring Finger Protein Like 1 | Protein Coding | 41 | GC14M068787 | 1.903153 | https://www.genecards.org/cgi-bin/carddisp.pl?gene=ZFP36L1 |
| COL2A1 | Collagen Type II Alpha 1 Chain | Protein Coding | 46 | GC12M047972 | 1.89596 | https://www.genecards.org/cgi-bin/carddisp.pl?gene=COL2A1 |
| PRPF19 | Pre-MRNA Processing Factor 19 | Protein Coding | 35 | GC11M060890 | 1.895542 | https://www.genecards.org/cgi-bin/carddisp.pl?gene=PRPF19 |
| GSR | Glutathione-Disulfide Reductase | Protein Coding | 47 | GC08M030678 | 1.895339 | https://www.genecards.org/cgi-bin/carddisp.pl?gene=GSR |
| TNC | Tenascin C | Protein Coding | 47 | GC09M115019 | 1.894332 | https://www.genecards.org/cgi-bin/carddisp.pl?gene=TNC |
| CHRNA7 | Cholinergic Receptor Nicotinic Alpha 7 Subunit | Protein Coding | 43 | GC15P031923 | 1.894305 | https://www.genecards.org/cgi-bin/carddisp.pl?gene=CHRNA7 |
| NT5E | 5'-Nucleotidase Ecto | Protein Coding | 48 | GC06P085449 | 1.893504 | https://www.genecards.org/cgi-bin/carddisp.pl?gene=NT5E |
| CD44 | CD44 Molecule (Indian Blood Group) | Protein Coding | 44 | GC11P035139 | 1.891539 | https://www.genecards.org/cgi-bin/carddisp.pl?gene=CD44 |
| NOTCH3 | Notch Receptor 3 | Protein Coding | 47 | GC19M015159 | 1.887036 | https://www.genecards.org/cgi-bin/carddisp.pl?gene=NOTCH3 |
| TMSB4X | Thymosin Beta 4 X-Linked | Protein Coding | 39 | GC0XP012975 | 1.885609 | https://www.genecards.org/cgi-bin/carddisp.pl?gene=TMSB4X |
| SIM2 | SIM BHLH Transcription Factor 2 | Protein Coding | 37 | GC21P036699 | 1.885077 | https://www.genecards.org/cgi-bin/carddisp.pl?gene=SIM2 |
| TLR7 | Toll Like Receptor 7 | Protein Coding | 45 | GC0XP012867 | 1.884009 | https://www.genecards.org/cgi-bin/carddisp.pl?gene=TLR7 |
| PTGIS | Prostaglandin I2 Synthase | Protein Coding | 46 | GC20M049503 | 1.882334 | https://www.genecards.org/cgi-bin/carddisp.pl?gene=PTGIS |
| NDUFS2 | NADH:Ubiquinone Oxidoreductase Core Subunit S2 | Protein Coding | 42 | GC01P161197 | 1.880957 | https://www.genecards.org/cgi-bin/carddisp.pl?gene=NDUFS2 |
| TGFA | Transforming Growth Factor Alpha | Protein Coding | 43 | GC02M070447 | 1.879537 | https://www.genecards.org/cgi-bin/carddisp.pl?gene=TGFA |
| TFF3 | Trefoil Factor 3 | Protein Coding | 39 | GC21M042311 | 1.876657 | https://www.genecards.org/cgi-bin/carddisp.pl?gene=TFF3 |
| TRPA1 | Transient Receptor Potential Cation Channel Subfamily A Member 1 | Protein Coding | 43 | GC08M072019 | 1.876278 | https://www.genecards.org/cgi-bin/carddisp.pl?gene=TRPA1 |
| TCF4 | Transcription Factor 4 | Protein Coding | 44 | GC18M055222 | 1.870531 | https://www.genecards.org/cgi-bin/carddisp.pl?gene=TCF4 |
| UCA1 | Urothelial Cancer Associated 1 | RNA Gene | 22 | GC19P015828 | 1.869832 | https://www.genecards.org/cgi-bin/carddisp.pl?gene=UCA1 |
| GFAP | Glial Fibrillary Acidic Protein | Protein Coding | 45 | GC17M044905 | 1.866554 | https://www.genecards.org/cgi-bin/carddisp.pl?gene=GFAP |
| NF1 | Neurofibromin 1 | Protein Coding | 47 | GC17P031094 | 1.864204 | https://www.genecards.org/cgi-bin/carddisp.pl?gene=NF1 |
| EDNRB | Endothelin Receptor Type B | Protein Coding | 47 | GC13M077895 | 1.863971 | https://www.genecards.org/cgi-bin/carddisp.pl?gene=EDNRB |
| GHRL | Ghrelin And Obestatin Prepropeptide | Protein Coding | 41 | GC03M010285 | 1.861633 | https://www.genecards.org/cgi-bin/carddisp.pl?gene=GHRL |
| MXI1 | MAX Interactor 1, Dimerization Protein | Protein Coding | 40 | GC10P110208 | 1.860634 | https://www.genecards.org/cgi-bin/carddisp.pl?gene=MXI1 |
| CLOCK | Clock Circadian Regulator | Protein Coding | 42 | GC04M055427 | 1.858966 | https://www.genecards.org/cgi-bin/carddisp.pl?gene=CLOCK |
| PDP1 | Pyruvate Dehydrogenase Phosphatase Catalytic Subunit 1 | Protein Coding | 44 | GC08P093857 | 1.858204 | https://www.genecards.org/cgi-bin/carddisp.pl?gene=PDP1 |
| APLNR | Apelin Receptor | Protein Coding | 42 | GC11M057233 | 1.858204 | https://www.genecards.org/cgi-bin/carddisp.pl?gene=APLNR |
| ATF2 | Activating Transcription Factor 2 | Protein Coding | 44 | GC02M175072 | 1.856452 | https://www.genecards.org/cgi-bin/carddisp.pl?gene=ATF2 |
| S100A4 | S100 Calcium Binding Protein A4 | Protein Coding | 42 | GC01M153543 | 1.855865 | https://www.genecards.org/cgi-bin/carddisp.pl?gene=S100A4 |
| FKBP8 | FKBP Prolyl Isomerase 8 | Protein Coding | 41 | GC19M018503 | 1.854102 | https://www.genecards.org/cgi-bin/carddisp.pl?gene=FKBP8 |
| TRPC1 | Transient Receptor Potential Cation Channel Subfamily C Member 1 | Protein Coding | 40 | GC03P142724 | 1.851798 | https://www.genecards.org/cgi-bin/carddisp.pl?gene=TRPC1 |
| MIR200B | MicroRNA 200b | RNA Gene | 20 | GC01P001167 | 1.851769 | https://www.genecards.org/cgi-bin/carddisp.pl?gene=MIR200B |
| PINK1 | PTEN Induced Kinase 1 | Protein Coding | 44 | GC01P020634 | 1.850279 | https://www.genecards.org/cgi-bin/carddisp.pl?gene=PINK1 |
| KLF5 | Kruppel Like Factor 5 | Protein Coding | 43 | GC13P073054 | 1.84918 | https://www.genecards.org/cgi-bin/carddisp.pl?gene=KLF5 |
| ITGA5 | Integrin Subunit Alpha 5 | Protein Coding | 46 | GC12M054683 | 1.848713 | https://www.genecards.org/cgi-bin/carddisp.pl?gene=ITGA5 |
| RECK | Reversion Inducing Cysteine Rich Protein With Kazal Motifs | Protein Coding | 39 | GC09P036036 | 1.848713 | https://www.genecards.org/cgi-bin/carddisp.pl?gene=RECK |
| IGFBP2 | Insulin Like Growth Factor Binding Protein 2 | Protein Coding | 41 | GC02P216632 | 1.848265 | https://www.genecards.org/cgi-bin/carddisp.pl?gene=IGFBP2 |
| FOXO1 | Forkhead Box O1 | Protein Coding | 46 | GC13M040555 | 1.84752 | https://www.genecards.org/cgi-bin/carddisp.pl?gene=FOXO1 |
| NDNF | Neuron Derived Neurotrophic Factor | Protein Coding | 34 | GC04M121029 | 1.845809 | https://www.genecards.org/cgi-bin/carddisp.pl?gene=NDNF |
| PLEKHN1 | Pleckstrin Homology Domain Containing N1 | Protein Coding | 28 | GC01P002919 | 1.845809 | https://www.genecards.org/cgi-bin/carddisp.pl?gene=PLEKHN1 |
| USF1 | Upstream Transcription Factor 1 | Protein Coding | 42 | GC01M161039 | 1.845036 | https://www.genecards.org/cgi-bin/carddisp.pl?gene=USF1 |
| LONP1 | Lon Peptidase 1, Mitochondrial | Protein Coding | 42 | GC19M005691 | 1.843274 | https://www.genecards.org/cgi-bin/carddisp.pl?gene=LONP1 |
| EDN2 | Endothelin 2 | Protein Coding | 38 | GC01M041478 | 1.843097 | https://www.genecards.org/cgi-bin/carddisp.pl?gene=EDN2 |
| KCNK3 | Potassium Two Pore Domain Channel Subfamily K Member 3 | Protein Coding | 47 | GC02P026692 | 1.842865 | https://www.genecards.org/cgi-bin/carddisp.pl?gene=KCNK3 |
| PSMB7 | Proteasome 20S Subunit Beta 7 | Protein Coding | 43 | GC09M124353 | 1.839575 | https://www.genecards.org/cgi-bin/carddisp.pl?gene=PSMB7 |
| PSMD9 | Proteasome 26S Subunit, Non-ATPase 9 | Protein Coding | 41 | GC12P124808 | 1.839575 | https://www.genecards.org/cgi-bin/carddisp.pl?gene=PSMD9 |
| MAP2 | Microtubule Associated Protein 2 | Protein Coding | 41 | GC02P209424 | 1.837296 | https://www.genecards.org/cgi-bin/carddisp.pl?gene=MAP2 |
| STAT5A | Signal Transducer And Activator Of Transcription 5A | Protein Coding | 44 | GC17P042287 | 1.835003 | https://www.genecards.org/cgi-bin/carddisp.pl?gene=STAT5A |
| ADORA3 | Adenosine A3 Receptor | Protein Coding | 44 | GC01M111499 | 1.834685 | https://www.genecards.org/cgi-bin/carddisp.pl?gene=ADORA3 |
| COL1A1 | Collagen Type I Alpha 1 Chain | Protein Coding | 48 | GC17M050183 | 1.834407 | https://www.genecards.org/cgi-bin/carddisp.pl?gene=COL1A1 |
| AQP3 | Aquaporin 3 (Gill Blood Group) | Protein Coding | 45 | GC09M033431 | 1.833549 | https://www.genecards.org/cgi-bin/carddisp.pl?gene=AQP3 |
| SLC6A4 | Solute Carrier Family 6 Member 4 | Protein Coding | 46 | GC17M030194 | 1.830672 | https://www.genecards.org/cgi-bin/carddisp.pl?gene=SLC6A4 |
| VDAC1 | Voltage Dependent Anion Channel 1 | Protein Coding | 43 | GC05M133975 | 1.828888 | https://www.genecards.org/cgi-bin/carddisp.pl?gene=VDAC1 |
| RNF4 | Ring Finger Protein 4 | Protein Coding | 39 | GC04P002462 | 1.828156 | https://www.genecards.org/cgi-bin/carddisp.pl?gene=RNF4 |
| PDGFRB | Platelet Derived Growth Factor Receptor Beta | Protein Coding | 51 | GC05M150113 | 1.827466 | https://www.genecards.org/cgi-bin/carddisp.pl?gene=PDGFRB |
| ULK1 | Unc-51 Like Autophagy Activating Kinase 1 | Protein Coding | 43 | GC12P131894 | 1.821881 | https://www.genecards.org/cgi-bin/carddisp.pl?gene=ULK1 |
| LOC108942766 | NANOG 5' Regulatory Region | Biological Region | 1 | GC12P016952 | 1.819079 | https://www.genecards.org/cgi-bin/carddisp.pl?gene=LOC108942766 |
| XRCC6 | X-Ray Repair Cross Complementing 6 | Protein Coding | 43 | GC22P041622 | 1.816552 | https://www.genecards.org/cgi-bin/carddisp.pl?gene=XRCC6 |
| ID1 | Inhibitor Of DNA Binding 1, HLH Protein | Protein Coding | 40 | GC20P031605 | 1.815602 | https://www.genecards.org/cgi-bin/carddisp.pl?gene=ID1 |
| DLL4 | Delta Like Canonical Notch Ligand 4 | Protein Coding | 43 | GC15P040929 | 1.812033 | https://www.genecards.org/cgi-bin/carddisp.pl?gene=DLL4 |
| GPX1 | Glutathione Peroxidase 1 | Protein Coding | 45 | GC03M050609 | 1.810771 | https://www.genecards.org/cgi-bin/carddisp.pl?gene=GPX1 |
| RB1 | RB Transcriptional Corepressor 1 | Protein Coding | 46 | GC13P048303 | 1.808024 | https://www.genecards.org/cgi-bin/carddisp.pl?gene=RB1 |
| PSME2 | Proteasome Activator Subunit 2 | Protein Coding | 40 | GC14M024143 | 1.806852 | https://www.genecards.org/cgi-bin/carddisp.pl?gene=PSME2 |
| APOE | Apolipoprotein E | Protein Coding | 47 | GC19P045397 | 1.80333 | https://www.genecards.org/cgi-bin/carddisp.pl?gene=APOE |
| KDM4B | Lysine Demethylase 4B | Protein Coding | 44 | GC19P004969 | 1.799212 | https://www.genecards.org/cgi-bin/carddisp.pl?gene=KDM4B |
| HBG2 | Hemoglobin Subunit Gamma 2 | Protein Coding | 39 | GC11M006038 | 1.798418 | https://www.genecards.org/cgi-bin/carddisp.pl?gene=HBG2 |
| HDAC9 | Histone Deacetylase 9 | Protein Coding | 45 | GC07P018086 | 1.797511 | https://www.genecards.org/cgi-bin/carddisp.pl?gene=HDAC9 |
| BMPR2 | Bone Morphogenetic Protein Receptor Type 2 | Protein Coding | 47 | GC02P202376 | 1.793688 | https://www.genecards.org/cgi-bin/carddisp.pl?gene=BMPR2 |
| HBB | Hemoglobin Subunit Beta | Protein Coding | 43 | GC11M006025 | 1.793313 | https://www.genecards.org/cgi-bin/carddisp.pl?gene=HBB |
| KNG1 | Kininogen 1 | Protein Coding | 43 | GC03P186717 | 1.791265 | https://www.genecards.org/cgi-bin/carddisp.pl?gene=KNG1 |
| SLC8A3 | Solute Carrier Family 8 Member A3 | Protein Coding | 41 | GC14M070044 | 1.79105 | https://www.genecards.org/cgi-bin/carddisp.pl?gene=SLC8A3 |
| SLC29A2 | Solute Carrier Family 29 Member 2 | Protein Coding | 43 | GC11M080069 | 1.790613 | https://www.genecards.org/cgi-bin/carddisp.pl?gene=SLC29A2 |
| CTRL | Chymotrypsin Like | Protein Coding | 37 | GC16M067927 | 1.787755 | https://www.genecards.org/cgi-bin/carddisp.pl?gene=CTRL |
| IL18 | Interleukin 18 | Protein Coding | 41 | GC11M112143 | 1.784541 | https://www.genecards.org/cgi-bin/carddisp.pl?gene=IL18 |
| ADAM10 | ADAM Metallopeptidase Domain 10 | Protein Coding | 50 | GC15M058588 | 1.783144 | https://www.genecards.org/cgi-bin/carddisp.pl?gene=ADAM10 |
| SOD3 | Superoxide Dismutase 3 | Protein Coding | 39 | GC04P024798 | 1.782682 | https://www.genecards.org/cgi-bin/carddisp.pl?gene=SOD3 |
| MIR338 | MicroRNA 338 | RNA Gene | 17 | GC17M081126 | 1.778036 | https://www.genecards.org/cgi-bin/carddisp.pl?gene=MIR338 |
| PSME3 | Proteasome Activator Subunit 3 | Protein Coding | 40 | GC17P042824 | 1.7766 | https://www.genecards.org/cgi-bin/carddisp.pl?gene=PSME3 |
| PSMC2 | Proteasome 26S Subunit, ATPase 2 | Protein Coding | 39 | GC07P103344 | 1.7766 | https://www.genecards.org/cgi-bin/carddisp.pl?gene=PSMC2 |
| P2RX2 | Purinergic Receptor P2X 2 | Protein Coding | 42 | GC12P132618 | 1.776098 | https://www.genecards.org/cgi-bin/carddisp.pl?gene=P2RX2 |
| PLD2 | Phospholipase D2 | Protein Coding | 46 | GC17P004808 | 1.774746 | https://www.genecards.org/cgi-bin/carddisp.pl?gene=PLD2 |
| CCL26 | C-C Motif Chemokine Ligand 26 | Protein Coding | 35 | GC07M075769 | 1.771493 | https://www.genecards.org/cgi-bin/carddisp.pl?gene=CCL26 |
| TMPRSS2 | Transmembrane Serine Protease 2 | Protein Coding | 43 | GC21M041464 | 1.770504 | https://www.genecards.org/cgi-bin/carddisp.pl?gene=TMPRSS2 |
| CHEK1 | Checkpoint Kinase 1 | Protein Coding | 47 | GC11P125625 | 1.767633 | https://www.genecards.org/cgi-bin/carddisp.pl?gene=CHEK1 |
| MIR124-3 | MicroRNA 124-3 | RNA Gene | 17 | GC20P063181 | 1.764055 | https://www.genecards.org/cgi-bin/carddisp.pl?gene=MIR124-3 |
| MIR301A | MicroRNA 301a | RNA Gene | 19 | GC17M059151 | 1.763825 | https://www.genecards.org/cgi-bin/carddisp.pl?gene=MIR301A |
| SST | Somatostatin | Protein Coding | 40 | GC03M187668 | 1.763473 | https://www.genecards.org/cgi-bin/carddisp.pl?gene=SST |
| CD24 | CD24 Molecule | Protein Coding | 33 | GC06M106969 | 1.761938 | https://www.genecards.org/cgi-bin/carddisp.pl?gene=CD24 |
| CCNB1 | Cyclin B1 | Protein Coding | 44 | GC05P069167 | 1.761528 | https://www.genecards.org/cgi-bin/carddisp.pl?gene=CCNB1 |
| HNF4A | Hepatocyte Nuclear Factor 4 Alpha | Protein Coding | 48 | GC20P044355 | 1.760041 | https://www.genecards.org/cgi-bin/carddisp.pl?gene=HNF4A |
| SYK | Spleen Associated Tyrosine Kinase | Protein Coding | 48 | GC09P091689 | 1.7593 | https://www.genecards.org/cgi-bin/carddisp.pl?gene=SYK |
| PTPRB | Protein Tyrosine Phosphatase Receptor Type B | Protein Coding | 43 | GC12M070516 | 1.758645 | https://www.genecards.org/cgi-bin/carddisp.pl?gene=PTPRB |
| ARNTL | Aryl Hydrocarbon Receptor Nuclear Translocator Like | Protein Coding | 40 | GC11P013276 | 1.758588 | https://www.genecards.org/cgi-bin/carddisp.pl?gene=ARNTL |
| CX3CR1 | C-X3-C Motif Chemokine Receptor 1 | Protein Coding | 43 | GC03M039279 | 1.758167 | https://www.genecards.org/cgi-bin/carddisp.pl?gene=CX3CR1 |
| MAP1LC3B | Microtubule Associated Protein 1 Light Chain 3 Beta | Protein Coding | 40 | GC16P087384 | 1.756896 | https://www.genecards.org/cgi-bin/carddisp.pl?gene=MAP1LC3B |
| MAP3K5 | Mitogen-Activated Protein Kinase Kinase Kinase 5 | Protein Coding | 45 | GC06M136557 | 1.755214 | https://www.genecards.org/cgi-bin/carddisp.pl?gene=MAP3K5 |
| KEAP1 | Kelch Like ECH Associated Protein 1 | Protein Coding | 46 | GC19M010486 | 1.754551 | https://www.genecards.org/cgi-bin/carddisp.pl?gene=KEAP1 |
| PIK3CB | Phosphatidylinositol-4,5-Bisphosphate 3-Kinase Catalytic Subunit Beta | Protein Coding | 47 | GC03M138652 | 1.753565 | https://www.genecards.org/cgi-bin/carddisp.pl?gene=PIK3CB |
| F2 | Coagulation Factor II, Thrombin | Protein Coding | 46 | GC11P046720 | 1.750603 | https://www.genecards.org/cgi-bin/carddisp.pl?gene=F2 |
| NDUFS4 | NADH:Ubiquinone Oxidoreductase Subunit S4 | Protein Coding | 42 | GC05P053560 | 1.749858 | https://www.genecards.org/cgi-bin/carddisp.pl?gene=NDUFS4 |
| SLC1A3 | Solute Carrier Family 1 Member 3 | Protein Coding | 48 | GC05P036632 | 1.747999 | https://www.genecards.org/cgi-bin/carddisp.pl?gene=SLC1A3 |
| KCNMB1 | Potassium Calcium-Activated Channel Subfamily M Regulatory Beta Subunit 1 | Protein Coding | 40 | GC05M170374 | 1.746039 | https://www.genecards.org/cgi-bin/carddisp.pl?gene=KCNMB1 |
| ROCK1 | Rho Associated Coiled-Coil Containing Protein Kinase 1 | Protein Coding | 48 | GC18M020946 | 1.742837 | https://www.genecards.org/cgi-bin/carddisp.pl?gene=ROCK1 |
| NTN1 | Netrin 1 | Protein Coding | 42 | GC17P009021 | 1.742705 | https://www.genecards.org/cgi-bin/carddisp.pl?gene=NTN1 |
| ATG5 | Autophagy Related 5 | Protein Coding | 42 | GC06M106045 | 1.739394 | https://www.genecards.org/cgi-bin/carddisp.pl?gene=ATG5 |
| VHLL | VHL Like | Protein Coding | 25 | GC01M156298 | 1.737673 | https://www.genecards.org/cgi-bin/carddisp.pl?gene=VHLL |
| CEBPB | CCAAT Enhancer Binding Protein Beta | Protein Coding | 42 | GC20P050190 | 1.736847 | https://www.genecards.org/cgi-bin/carddisp.pl?gene=CEBPB |
| NRG1 | Neuregulin 1 | Protein Coding | 44 | GC08P031639 | 1.734483 | https://www.genecards.org/cgi-bin/carddisp.pl?gene=NRG1 |
| ACAN | Aggrecan | Protein Coding | 45 | GC15P088827 | 1.733026 | https://www.genecards.org/cgi-bin/carddisp.pl?gene=ACAN |
| BMP4 | Bone Morphogenetic Protein 4 | Protein Coding | 47 | GC14M053949 | 1.730582 | https://www.genecards.org/cgi-bin/carddisp.pl?gene=BMP4 |
| KLK3 | Kallikrein Related Peptidase 3 | Protein Coding | 43 | GC19P050854 | 1.730582 | https://www.genecards.org/cgi-bin/carddisp.pl?gene=KLK3 |
| PSMD7 | Proteasome 26S Subunit, Non-ATPase 7 | Protein Coding | 42 | GC16P074296 | 1.727853 | https://www.genecards.org/cgi-bin/carddisp.pl?gene=PSMD7 |
| PSME1 | Proteasome Activator Subunit 1 | Protein Coding | 40 | GC14P024136 | 1.727853 | https://www.genecards.org/cgi-bin/carddisp.pl?gene=PSME1 |
| PSMA2 | Proteasome 20S Subunit Alpha 2 | Protein Coding | 40 | GC07M042916 | 1.727853 | https://www.genecards.org/cgi-bin/carddisp.pl?gene=PSMA2 |
| PSMD12 | Proteasome 26S Subunit, Non-ATPase 12 | Protein Coding | 40 | GC17M067337 | 1.727853 | https://www.genecards.org/cgi-bin/carddisp.pl?gene=PSMD12 |
| PSMB3 | Proteasome 20S Subunit Beta 3 | Protein Coding | 39 | GC17P038752 | 1.727853 | https://www.genecards.org/cgi-bin/carddisp.pl?gene=PSMB3 |
| PSMB2 | Proteasome 20S Subunit Beta 2 | Protein Coding | 39 | GC01M035599 | 1.727853 | https://www.genecards.org/cgi-bin/carddisp.pl?gene=PSMB2 |
| PSMD8 | Proteasome 26S Subunit, Non-ATPase 8 | Protein Coding | 38 | GC19P038374 | 1.727853 | https://www.genecards.org/cgi-bin/carddisp.pl?gene=PSMD8 |
| PSMF1 | Proteasome Inhibitor Subunit 1 | Protein Coding | 37 | GC20P001113 | 1.727853 | https://www.genecards.org/cgi-bin/carddisp.pl?gene=PSMF1 |
| PSMD6 | Proteasome 26S Subunit, Non-ATPase 6 | Protein Coding | 36 | GC03M063973 | 1.727853 | https://www.genecards.org/cgi-bin/carddisp.pl?gene=PSMD6 |
| FABP1 | Fatty Acid Binding Protein 1 | Protein Coding | 40 | GC02M088122 | 1.726936 | https://www.genecards.org/cgi-bin/carddisp.pl?gene=FABP1 |
| NR3C1 | Nuclear Receptor Subfamily 3 Group C Member 1 | Protein Coding | 48 | GC05M143277 | 1.724157 | https://www.genecards.org/cgi-bin/carddisp.pl?gene=NR3C1 |
| MIR429 | MicroRNA 429 | RNA Gene | 19 | GC01P002925 | 1.721913 | https://www.genecards.org/cgi-bin/carddisp.pl?gene=MIR429 |
| COL1A2 | Collagen Type I Alpha 2 Chain | Protein Coding | 45 | GC07P094394 | 1.717826 | https://www.genecards.org/cgi-bin/carddisp.pl?gene=COL1A2 |
| MAP3K7 | Mitogen-Activated Protein Kinase Kinase Kinase 7 | Protein Coding | 49 | GC06M090513 | 1.716353 | https://www.genecards.org/cgi-bin/carddisp.pl?gene=MAP3K7 |
| NBN | Nibrin | Protein Coding | 45 | GC08M089933 | 1.715665 | https://www.genecards.org/cgi-bin/carddisp.pl?gene=NBN |
| NTS | Neurotensin | Protein Coding | 39 | GC12P085876 | 1.714935 | https://www.genecards.org/cgi-bin/carddisp.pl?gene=NTS |
| LMNA | Lamin A/C | Protein Coding | 46 | GC01P156082 | 1.714195 | https://www.genecards.org/cgi-bin/carddisp.pl?gene=LMNA |
| LCN2 | Lipocalin 2 | Protein Coding | 40 | GC09P128149 | 1.712896 | https://www.genecards.org/cgi-bin/carddisp.pl?gene=LCN2 |
| KLF2 | Kruppel Like Factor 2 | Protein Coding | 39 | GC19P031515 | 1.709848 | https://www.genecards.org/cgi-bin/carddisp.pl?gene=KLF2 |
| SLC2A4 | Solute Carrier Family 2 Member 4 | Protein Coding | 44 | GC17P010044 | 1.709834 | https://www.genecards.org/cgi-bin/carddisp.pl?gene=SLC2A4 |
| MIR138-1 | MicroRNA 138-1 | RNA Gene | 19 | GC03P044115 | 1.709119 | https://www.genecards.org/cgi-bin/carddisp.pl?gene=MIR138-1 |
| NCOA1 | Nuclear Receptor Coactivator 1 | Protein Coding | 42 | GC02P024492 | 1.708711 | https://www.genecards.org/cgi-bin/carddisp.pl?gene=NCOA1 |
| ERVW-1 | Endogenous Retrovirus Group W Member 1, Envelope | Protein Coding | 32 | GC07M092468 | 1.707337 | https://www.genecards.org/cgi-bin/carddisp.pl?gene=ERVW-1 |
| PPIG | Peptidylprolyl Isomerase G | Protein Coding | 39 | GC02P169584 | 1.70299 | https://www.genecards.org/cgi-bin/carddisp.pl?gene=PPIG |
| PPP1R12A | Protein Phosphatase 1 Regulatory Subunit 12A | Protein Coding | 42 | GC12M079773 | 1.70172 | https://www.genecards.org/cgi-bin/carddisp.pl?gene=PPP1R12A |
| CASP1 | Caspase 1 | Protein Coding | 48 | GC11M105025 | 1.701222 | https://www.genecards.org/cgi-bin/carddisp.pl?gene=CASP1 |
| GPER1 | G Protein-Coupled Estrogen Receptor 1 | Protein Coding | 36 | GC07P001698 | 1.70017 | https://www.genecards.org/cgi-bin/carddisp.pl?gene=GPER1 |
| CHKA | Choline Kinase Alpha | Protein Coding | 40 | GC11M068052 | 1.69998 | https://www.genecards.org/cgi-bin/carddisp.pl?gene=CHKA |
| PLIN2 | Perilipin 2 | Protein Coding | 40 | GC09M019149 | 1.699573 | https://www.genecards.org/cgi-bin/carddisp.pl?gene=PLIN2 |
| P4HA1 | Prolyl 4-Hydroxylase Subunit Alpha 1 | Protein Coding | 39 | GC10M073007 | 1.699373 | https://www.genecards.org/cgi-bin/carddisp.pl?gene=P4HA1 |
| ACKR3 | Atypical Chemokine Receptor 3 | Protein Coding | 40 | GC02P236537 | 1.697024 | https://www.genecards.org/cgi-bin/carddisp.pl?gene=ACKR3 |
| GATA1 | GATA Binding Protein 1 | Protein Coding | 45 | GC0XP048786 | 1.696917 | https://www.genecards.org/cgi-bin/carddisp.pl?gene=GATA1 |
| TRAF6 | TNF Receptor Associated Factor 6 | Protein Coding | 44 | GC11M036467 | 1.696917 | https://www.genecards.org/cgi-bin/carddisp.pl?gene=TRAF6 |
| H2AX | H2A.X Variant Histone | Protein Coding | 34 | GC11M119118 | 1.694266 | https://www.genecards.org/cgi-bin/carddisp.pl?gene=H2AX |
| NEAT1 | Nuclear Paraspeckle Assembly Transcript 1 | RNA Gene | 23 | GC11P068094 | 1.694187 | https://www.genecards.org/cgi-bin/carddisp.pl?gene=NEAT1 |
| SKP1 | S-Phase Kinase Associated Protein 1 | Protein Coding | 40 | GC05M134148 | 1.692825 | https://www.genecards.org/cgi-bin/carddisp.pl?gene=SKP1 |
| TAL1 | TAL BHLH Transcription Factor 1, Erythroid Differentiation Factor | Protein Coding | 44 | GC01M047216 | 1.691559 | https://www.genecards.org/cgi-bin/carddisp.pl?gene=TAL1 |
| MYCN | MYCN Proto-Oncogene, BHLH Transcription Factor | Protein Coding | 44 | GC02P015949 | 1.688876 | https://www.genecards.org/cgi-bin/carddisp.pl?gene=MYCN |
| DUSP2 | Dual Specificity Phosphatase 2 | Protein Coding | 39 | GC02M097184 | 1.688193 | https://www.genecards.org/cgi-bin/carddisp.pl?gene=DUSP2 |
| HMGCR | 3-Hydroxy-3-Methylglutaryl-CoA Reductase | Protein Coding | 44 | GC05P075336 | 1.686514 | https://www.genecards.org/cgi-bin/carddisp.pl?gene=HMGCR |
| PLA2G6 | Phospholipase A2 Group VI | Protein Coding | 46 | GC22M054005 | 1.68503 | https://www.genecards.org/cgi-bin/carddisp.pl?gene=PLA2G6 |
| HDC | Histidine Decarboxylase | Protein Coding | 43 | GC15M050241 | 1.684862 | https://www.genecards.org/cgi-bin/carddisp.pl?gene=HDC |
| KIT | KIT Proto-Oncogene, Receptor Tyrosine Kinase | Protein Coding | 50 | GC04P054657 | 1.683694 | https://www.genecards.org/cgi-bin/carddisp.pl?gene=KIT |
| RHO | Rhodopsin | Protein Coding | 45 | GC03P132741 | 1.682337 | https://www.genecards.org/cgi-bin/carddisp.pl?gene=RHO |
| NPEPPS | Aminopeptidase Puromycin Sensitive | Protein Coding | 40 | GC17P047522 | 1.681894 | https://www.genecards.org/cgi-bin/carddisp.pl?gene=NPEPPS |
| NTRK1 | Neurotrophic Receptor Tyrosine Kinase 1 | Protein Coding | 47 | GC01P156815 | 1.681345 | https://www.genecards.org/cgi-bin/carddisp.pl?gene=NTRK1 |
| WT1 | WT1 Transcription Factor | Protein Coding | 47 | GC11M032365 | 1.681102 | https://www.genecards.org/cgi-bin/carddisp.pl?gene=WT1 |
| INHBA | Inhibin Subunit Beta A | Protein Coding | 42 | GC07M041668 | 1.681102 | https://www.genecards.org/cgi-bin/carddisp.pl?gene=INHBA |
| PIAS4 | Protein Inhibitor Of Activated STAT 4 | Protein Coding | 40 | GC19P004007 | 1.680548 | https://www.genecards.org/cgi-bin/carddisp.pl?gene=PIAS4 |
| MMP7 | Matrix Metallopeptidase 7 | Protein Coding | 46 | GC11M102425 | 1.671661 | https://www.genecards.org/cgi-bin/carddisp.pl?gene=MMP7 |
| EEF2 | Eukaryotic Translation Elongation Factor 2 | Protein Coding | 46 | GC19M003976 | 1.670328 | https://www.genecards.org/cgi-bin/carddisp.pl?gene=EEF2 |
| CRH | Corticotropin Releasing Hormone | Protein Coding | 42 | GC08M066176 | 1.669194 | https://www.genecards.org/cgi-bin/carddisp.pl?gene=CRH |
| MMP3 | Matrix Metallopeptidase 3 | Protein Coding | 48 | GC11M102835 | 1.668717 | https://www.genecards.org/cgi-bin/carddisp.pl?gene=MMP3 |
| SLC16A7 | Solute Carrier Family 16 Member 7 | Protein Coding | 42 | GC12P059596 | 1.667963 | https://www.genecards.org/cgi-bin/carddisp.pl?gene=SLC16A7 |
| PLA2G4A | Phospholipase A2 Group IVA | Protein Coding | 47 | GC01P186798 | 1.667169 | https://www.genecards.org/cgi-bin/carddisp.pl?gene=PLA2G4A |
| ITGAM | Integrin Subunit Alpha M | Protein Coding | 44 | GC16P037846 | 1.667169 | https://www.genecards.org/cgi-bin/carddisp.pl?gene=ITGAM |
| TDRD9 | Tudor Domain Containing 9 | Protein Coding | 38 | GC14P107929 | 1.666796 | https://www.genecards.org/cgi-bin/carddisp.pl?gene=TDRD9 |
| ZNF195 | Zinc Finger Protein 195 | Protein Coding | 36 | GC11M003336 | 1.666796 | https://www.genecards.org/cgi-bin/carddisp.pl?gene=ZNF195 |
| HIGD2AP1 | HIGD2A Pseudogene 1 | Pseudogene | 3 | GC02M231443 | 1.666796 | https://www.genecards.org/cgi-bin/carddisp.pl?gene=HIGD2AP1 |
| PIN1 | Peptidylprolyl Cis/Trans Isomerase, NIMA-Interacting 1 | Protein Coding | 44 | GC19P009835 | 1.666076 | https://www.genecards.org/cgi-bin/carddisp.pl?gene=PIN1 |
| GH1 | Growth Hormone 1 | Protein Coding | 42 | GC17M063917 | 1.664922 | https://www.genecards.org/cgi-bin/carddisp.pl?gene=GH1 |
| HNRNPM | Heterogeneous Nuclear Ribonucleoprotein M | Protein Coding | 38 | GC19P008444 | 1.659563 | https://www.genecards.org/cgi-bin/carddisp.pl?gene=HNRNPM |
| HDAC5 | Histone Deacetylase 5 | Protein Coding | 45 | GC17M044076 | 1.658497 | https://www.genecards.org/cgi-bin/carddisp.pl?gene=HDAC5 |
| DRD2 | Dopamine Receptor D2 | Protein Coding | 48 | GC11M113409 | 1.657447 | https://www.genecards.org/cgi-bin/carddisp.pl?gene=DRD2 |
| MIR106B | MicroRNA 106b | RNA Gene | 21 | GC07M101076 | 1.656004 | https://www.genecards.org/cgi-bin/carddisp.pl?gene=MIR106B |
| SMARCA4 | SWI/SNF Related, Matrix Associated, Actin Dependent Regulator Of Chromatin, Subfamily A, Member 4 | Protein Coding | 47 | GC19P010932 | 1.655858 | https://www.genecards.org/cgi-bin/carddisp.pl?gene=SMARCA4 |
| USF2 | Upstream Transcription Factor 2, C-Fos Interacting | Protein Coding | 38 | GC19P035268 | 1.654359 | https://www.genecards.org/cgi-bin/carddisp.pl?gene=USF2 |
| SLC1A5 | Solute Carrier Family 1 Member 5 | Protein Coding | 42 | GC19M057269 | 1.654234 | https://www.genecards.org/cgi-bin/carddisp.pl?gene=SLC1A5 |
| TSC1 | TSC Complex Subunit 1 | Protein Coding | 45 | GC09M132891 | 1.649167 | https://www.genecards.org/cgi-bin/carddisp.pl?gene=TSC1 |
| USP20 | Ubiquitin Specific Peptidase 20 | Protein Coding | 39 | GC09P129834 | 1.648554 | https://www.genecards.org/cgi-bin/carddisp.pl?gene=USP20 |
| UBA52 | Ubiquitin A-52 Residue Ribosomal Protein Fusion Product 1 | Protein Coding | 39 | GC19P018563 | 1.647788 | https://www.genecards.org/cgi-bin/carddisp.pl?gene=UBA52 |
| SIRT3 | Sirtuin 3 | Protein Coding | 45 | GC11M000215 | 1.647709 | https://www.genecards.org/cgi-bin/carddisp.pl?gene=SIRT3 |
| GZMB | Granzyme B | Protein Coding | 43 | GC14M024630 | 1.645035 | https://www.genecards.org/cgi-bin/carddisp.pl?gene=GZMB |
| HSF1 | Heat Shock Transcription Factor 1 | Protein Coding | 43 | GC08P144291 | 1.644949 | https://www.genecards.org/cgi-bin/carddisp.pl?gene=HSF1 |
| PAK1 | P21 (RAC1) Activated Kinase 1 | Protein Coding | 46 | GC11M080433 | 1.642998 | https://www.genecards.org/cgi-bin/carddisp.pl?gene=PAK1 |
| CGA | Glycoprotein Hormones, Alpha Polypeptide | Protein Coding | 40 | GC06M087085 | 1.642694 | https://www.genecards.org/cgi-bin/carddisp.pl?gene=CGA |
| IVL | Involucrin | Protein Coding | 36 | GC01P152881 | 1.642694 | https://www.genecards.org/cgi-bin/carddisp.pl?gene=IVL |
| ITGB3 | Integrin Subunit Beta 3 | Protein Coding | 47 | GC17P048246 | 1.638306 | https://www.genecards.org/cgi-bin/carddisp.pl?gene=ITGB3 |
| ANXA1 | Annexin A1 | Protein Coding | 47 | GC09P073151 | 1.632186 | https://www.genecards.org/cgi-bin/carddisp.pl?gene=ANXA1 |
| SP3 | Sp3 Transcription Factor | Protein Coding | 41 | GC02M173882 | 1.632186 | https://www.genecards.org/cgi-bin/carddisp.pl?gene=SP3 |
| TOP3A | DNA Topoisomerase III Alpha | Protein Coding | 39 | GC17M018271 | 1.632186 | https://www.genecards.org/cgi-bin/carddisp.pl?gene=TOP3A |
| TIMP2 | TIMP Metallopeptidase Inhibitor 2 | Protein Coding | 42 | GC17M078852 | 1.630774 | https://www.genecards.org/cgi-bin/carddisp.pl?gene=TIMP2 |
| HDAC7 | Histone Deacetylase 7 | Protein Coding | 43 | GC12M047782 | 1.630486 | https://www.genecards.org/cgi-bin/carddisp.pl?gene=HDAC7 |
| AGTR1 | Angiotensin II Receptor Type 1 | Protein Coding | 48 | GC03P148697 | 1.630309 | https://www.genecards.org/cgi-bin/carddisp.pl?gene=AGTR1 |
| GBE1 | 1,4-Alpha-Glucan Branching Enzyme 1 | Protein Coding | 42 | GC03M081489 | 1.628397 | https://www.genecards.org/cgi-bin/carddisp.pl?gene=GBE1 |
| RPSA | Ribosomal Protein SA | Protein Coding | 42 | GC03P039406 | 1.626665 | https://www.genecards.org/cgi-bin/carddisp.pl?gene=RPSA |
| SHH | Sonic Hedgehog Signaling Molecule | Protein Coding | 47 | GC07M155799 | 1.623101 | https://www.genecards.org/cgi-bin/carddisp.pl?gene=SHH |
| CDKN2A | Cyclin Dependent Kinase Inhibitor 2A | Protein Coding | 48 | GC09M021967 | 1.622865 | https://www.genecards.org/cgi-bin/carddisp.pl?gene=CDKN2A |
| G6PD | Glucose-6-Phosphate Dehydrogenase | Protein Coding | 47 | GC0XM154531 | 1.620813 | https://www.genecards.org/cgi-bin/carddisp.pl?gene=G6PD |
| COX4I2 | Cytochrome C Oxidase Subunit 4I2 | Protein Coding | 40 | GC20P031637 | 1.620061 | https://www.genecards.org/cgi-bin/carddisp.pl?gene=COX4I2 |
| TAGLN | Transgelin | Protein Coding | 41 | GC11P117199 | 1.616145 | https://www.genecards.org/cgi-bin/carddisp.pl?gene=TAGLN |
| EIF4G1 | Eukaryotic Translation Initiation Factor 4 Gamma 1 | Protein Coding | 44 | GC03P184314 | 1.615278 | https://www.genecards.org/cgi-bin/carddisp.pl?gene=EIF4G1 |
| ANXA5 | Annexin A5 | Protein Coding | 44 | GC04M121667 | 1.612838 | https://www.genecards.org/cgi-bin/carddisp.pl?gene=ANXA5 |
| COL18A1 | Collagen Type XVIII Alpha 1 Chain | Protein Coding | 43 | GC21P045405 | 1.61212 | https://www.genecards.org/cgi-bin/carddisp.pl?gene=COL18A1 |
| PIM1 | Pim-1 Proto-Oncogene, Serine/Threonine Kinase | Protein Coding | 47 | GC06P070299 | 1.609417 | https://www.genecards.org/cgi-bin/carddisp.pl?gene=PIM1 |
| MBP | Myelin Basic Protein | Protein Coding | 43 | GC18M076978 | 1.606885 | https://www.genecards.org/cgi-bin/carddisp.pl?gene=MBP |
| NAMPT | Nicotinamide Phosphoribosyltransferase | Protein Coding | 45 | GC07M106248 | 1.606306 | https://www.genecards.org/cgi-bin/carddisp.pl?gene=NAMPT |
| NRF1 | Nuclear Respiratory Factor 1 | Protein Coding | 41 | GC07P129611 | 1.605597 | https://www.genecards.org/cgi-bin/carddisp.pl?gene=NRF1 |
| AGO2 | Argonaute RISC Catalytic Component 2 | Protein Coding | 39 | GC08M140522 | 1.604445 | https://www.genecards.org/cgi-bin/carddisp.pl?gene=AGO2 |
| KDM1A | Lysine Demethylase 1A | Protein Coding | 46 | GC01P023019 | 1.602724 | https://www.genecards.org/cgi-bin/carddisp.pl?gene=KDM1A |
| ACTB | Actin Beta | Protein Coding | 47 | GC07M005527 | 1.602608 | https://www.genecards.org/cgi-bin/carddisp.pl?gene=ACTB |
| CBFA2T3 | CBFA2/RUNX1 Partner Transcriptional Co-Repressor 3 | Protein Coding | 40 | GC16M088874 | 1.601741 | https://www.genecards.org/cgi-bin/carddisp.pl?gene=CBFA2T3 |
| ENDOG | Endonuclease G | Protein Coding | 40 | GC09P128818 | 1.601306 | https://www.genecards.org/cgi-bin/carddisp.pl?gene=ENDOG |
| MUC5AC | Mucin 5AC, Oligomeric Mucus/Gel-Forming | Protein Coding | 36 | GC11P001396 | 1.598648 | https://www.genecards.org/cgi-bin/carddisp.pl?gene=MUC5AC |
| SOX2 | SRY-Box Transcription Factor 2 | Protein Coding | 44 | GC03P181711 | 1.598466 | https://www.genecards.org/cgi-bin/carddisp.pl?gene=SOX2 |
| LOC111258525 | NOS1 1f And 1g Alternate Promoter Region | Biological Region | 1 | GC12P117361 | 1.59754 | https://www.genecards.org/cgi-bin/carddisp.pl?gene=LOC111258525 |
| KCNH2 | Potassium Voltage-Gated Channel Subfamily H Member 2 | Protein Coding | 48 | GC07M150944 | 1.596468 | https://www.genecards.org/cgi-bin/carddisp.pl?gene=KCNH2 |
| SNRPD1 | Small Nuclear Ribonucleoprotein D1 Polypeptide | Protein Coding | 36 | GC18P021612 | 1.594915 | https://www.genecards.org/cgi-bin/carddisp.pl?gene=SNRPD1 |
| ATP2A2 | ATPase Sarcoplasmic/Endoplasmic Reticulum Ca2+ Transporting 2 | Protein Coding | 49 | GC12P110280 | 1.591322 | https://www.genecards.org/cgi-bin/carddisp.pl?gene=ATP2A2 |
| CDC42 | Cell Division Cycle 42 | Protein Coding | 48 | GC01P022208 | 1.590821 | https://www.genecards.org/cgi-bin/carddisp.pl?gene=CDC42 |
| CX3CL1 | C-X3-C Motif Chemokine Ligand 1 | Protein Coding | 39 | GC16P057372 | 1.590213 | https://www.genecards.org/cgi-bin/carddisp.pl?gene=CX3CL1 |
| NLRP3 | NLR Family Pyrin Domain Containing 3 | Protein Coding | 45 | GC01P247415 | 1.585022 | https://www.genecards.org/cgi-bin/carddisp.pl?gene=NLRP3 |
| VLDLR | Very Low Density Lipoprotein Receptor | Protein Coding | 47 | GC09P002611 | 1.584579 | https://www.genecards.org/cgi-bin/carddisp.pl?gene=VLDLR |
| LOC110806306 | Telomerase RNA Component (TERC) Promoter | Biological Region | 2 | GC03P169764 | 1.584303 | https://www.genecards.org/cgi-bin/carddisp.pl?gene=LOC110806306 |
| ALOX15B | Arachidonate 15-Lipoxygenase Type B | Protein Coding | 40 | GC17P008039 | 1.583976 | https://www.genecards.org/cgi-bin/carddisp.pl?gene=ALOX15B |
| MIR191 | MicroRNA 191 | RNA Gene | 21 | GC03M050578 | 1.583976 | https://www.genecards.org/cgi-bin/carddisp.pl?gene=MIR191 |
| VASP | Vasodilator Stimulated Phosphoprotein | Protein Coding | 41 | GC19P045512 | 1.579078 | https://www.genecards.org/cgi-bin/carddisp.pl?gene=VASP |
| IFI27 | Interferon Alpha Inducible Protein 27 | Protein Coding | 37 | GC14P094104 | 1.576443 | https://www.genecards.org/cgi-bin/carddisp.pl?gene=IFI27 |
| HSP90AA2P | Heat Shock Protein 90 Alpha Family Class A Member 2, Pseudogene | Pseudogene | 18 | GC11M027888 | 1.576443 | https://www.genecards.org/cgi-bin/carddisp.pl?gene=HSP90AA2P |
| EZH2 | Enhancer Of Zeste 2 Polycomb Repressive Complex 2 Subunit | Protein Coding | 51 | GC07M148807 | 1.576423 | https://www.genecards.org/cgi-bin/carddisp.pl?gene=EZH2 |
| OPA1 | OPA1 Mitochondrial Dynamin Like GTPase | Protein Coding | 42 | GC03P193594 | 1.576293 | https://www.genecards.org/cgi-bin/carddisp.pl?gene=OPA1 |
| COL3A1 | Collagen Type III Alpha 1 Chain | Protein Coding | 44 | GC02P188974 | 1.575298 | https://www.genecards.org/cgi-bin/carddisp.pl?gene=COL3A1 |
| PRL | Prolactin | Protein Coding | 40 | GC06M022287 | 1.573894 | https://www.genecards.org/cgi-bin/carddisp.pl?gene=PRL |
| ISCU | Iron-Sulfur Cluster Assembly Enzyme | Protein Coding | 42 | GC12P108561 | 1.573577 | https://www.genecards.org/cgi-bin/carddisp.pl?gene=ISCU |
| XBP1 | X-Box Binding Protein 1 | Protein Coding | 43 | GC22M028794 | 1.572018 | https://www.genecards.org/cgi-bin/carddisp.pl?gene=XBP1 |
| MLH1 | MutL Homolog 1 | Protein Coding | 46 | GC03P036993 | 1.56877 | https://www.genecards.org/cgi-bin/carddisp.pl?gene=MLH1 |
| ADAMTS1 | ADAM Metallopeptidase With Thrombospondin Type 1 Motif 1 | Protein Coding | 43 | GC21M026835 | 1.566119 | https://www.genecards.org/cgi-bin/carddisp.pl?gene=ADAMTS1 |
| MIR199A2 | MicroRNA 199a-2 | RNA Gene | 19 | GC01M172235 | 1.564632 | https://www.genecards.org/cgi-bin/carddisp.pl?gene=MIR199A2 |
| NCOA2 | Nuclear Receptor Coactivator 2 | Protein Coding | 43 | GC08M070109 | 1.564605 | https://www.genecards.org/cgi-bin/carddisp.pl?gene=NCOA2 |
| CDK2 | Cyclin Dependent Kinase 2 | Protein Coding | 49 | GC12P055966 | 1.557907 | https://www.genecards.org/cgi-bin/carddisp.pl?gene=CDK2 |
| MIR146A | MicroRNA 146a | RNA Gene | 22 | GC05P160485 | 1.556757 | https://www.genecards.org/cgi-bin/carddisp.pl?gene=MIR146A |
| MIR124-1 | MicroRNA 124-1 | RNA Gene | 21 | GC08M009903 | 1.554556 | https://www.genecards.org/cgi-bin/carddisp.pl?gene=MIR124-1 |
| RPA1 | Replication Protein A1 | Protein Coding | 44 | GC17P001829 | 1.55382 | https://www.genecards.org/cgi-bin/carddisp.pl?gene=RPA1 |
| MMP13 | Matrix Metallopeptidase 13 | Protein Coding | 48 | GC11M102942 | 1.551636 | https://www.genecards.org/cgi-bin/carddisp.pl?gene=MMP13 |
| F12 | Coagulation Factor XII | Protein Coding | 46 | GC05M177402 | 1.551636 | https://www.genecards.org/cgi-bin/carddisp.pl?gene=F12 |
| PRKCE | Protein Kinase C Epsilon | Protein Coding | 47 | GC02P045651 | 1.546382 | https://www.genecards.org/cgi-bin/carddisp.pl?gene=PRKCE |
| BAK1 | BCL2 Antagonist/Killer 1 | Protein Coding | 42 | GC06M033572 | 1.546144 | https://www.genecards.org/cgi-bin/carddisp.pl?gene=BAK1 |
| NR4A3 | Nuclear Receptor Subfamily 4 Group A Member 3 | Protein Coding | 43 | GC09P099821 | 1.545939 | https://www.genecards.org/cgi-bin/carddisp.pl?gene=NR4A3 |
| TFAP2A | Transcription Factor AP-2 Alpha | Protein Coding | 46 | GC06M010393 | 1.545865 | https://www.genecards.org/cgi-bin/carddisp.pl?gene=TFAP2A |
| TMEM204 | Transmembrane Protein 204 | Protein Coding | 31 | GC16P001528 | 1.545793 | https://www.genecards.org/cgi-bin/carddisp.pl?gene=TMEM204 |
| RGCC | Regulator Of Cell Cycle | Protein Coding | 33 | GC13P041457 | 1.545042 | https://www.genecards.org/cgi-bin/carddisp.pl?gene=RGCC |
| TNFSF11 | TNF Superfamily Member 11 | Protein Coding | 44 | GC13P042562 | 1.544818 | https://www.genecards.org/cgi-bin/carddisp.pl?gene=TNFSF11 |
| TGM2 | Transglutaminase 2 | Protein Coding | 45 | GC20M038127 | 1.544074 | https://www.genecards.org/cgi-bin/carddisp.pl?gene=TGM2 |
| MIR224 | MicroRNA 224 | RNA Gene | 17 | GC0XM151958 | 1.543861 | https://www.genecards.org/cgi-bin/carddisp.pl?gene=MIR224 |
| IDH2 | Isocitrate Dehydrogenase (NADP(+)) 2 | Protein Coding | 50 | GC15M090083 | 1.542912 | https://www.genecards.org/cgi-bin/carddisp.pl?gene=IDH2 |
| IL33 | Interleukin 33 | Protein Coding | 36 | GC09P006226 | 1.541089 | https://www.genecards.org/cgi-bin/carddisp.pl?gene=IL33 |
| CITED4 | Cbp/P300 Interacting Transactivator With Glu/Asp Rich Carboxy-Terminal Domain 4 | Protein Coding | 29 | GC01M040861 | 1.53867 | https://www.genecards.org/cgi-bin/carddisp.pl?gene=CITED4 |
| ALOX15 | Arachidonate 15-Lipoxygenase | Protein Coding | 42 | GC17M004630 | 1.538322 | https://www.genecards.org/cgi-bin/carddisp.pl?gene=ALOX15 |
| CSNK2A1 | Casein Kinase 2 Alpha 1 | Protein Coding | 49 | GC20M000472 | 1.537537 | https://www.genecards.org/cgi-bin/carddisp.pl?gene=CSNK2A1 |
| HDAC3 | Histone Deacetylase 3 | Protein Coding | 47 | GC05M141583 | 1.537537 | https://www.genecards.org/cgi-bin/carddisp.pl?gene=HDAC3 |
| REN | Renin | Protein Coding | 45 | GC01M204154 | 1.537537 | https://www.genecards.org/cgi-bin/carddisp.pl?gene=REN |
| BACE1 | Beta-Secretase 1 | Protein Coding | 45 | GC11M117285 | 1.536491 | https://www.genecards.org/cgi-bin/carddisp.pl?gene=BACE1 |
| MIR101-1 | MicroRNA 101-1 | RNA Gene | 18 | GC01M065058 | 1.536279 | https://www.genecards.org/cgi-bin/carddisp.pl?gene=MIR101-1 |
| HSPA9 | Heat Shock Protein Family A (Hsp70) Member 9 | Protein Coding | 45 | GC05M138554 | 1.533966 | https://www.genecards.org/cgi-bin/carddisp.pl?gene=HSPA9 |
| PHB2 | Prohibitin 2 | Protein Coding | 39 | GC12M006965 | 1.532803 | https://www.genecards.org/cgi-bin/carddisp.pl?gene=PHB2 |
| MIR27A | MicroRNA 27a | RNA Gene | 22 | GC19M014293 | 1.530847 | https://www.genecards.org/cgi-bin/carddisp.pl?gene=MIR27A |
| FADD | Fas Associated Via Death Domain | Protein Coding | 44 | GC11P070203 | 1.530341 | https://www.genecards.org/cgi-bin/carddisp.pl?gene=FADD |
| HK1 | Hexokinase 1 | Protein Coding | 48 | GC10P069269 | 1.529018 | https://www.genecards.org/cgi-bin/carddisp.pl?gene=HK1 |
| DARS1-AS1 | DARS1 Antisense RNA 1 | RNA Gene | 11 | GC02P135986 | 1.522867 | https://www.genecards.org/cgi-bin/carddisp.pl?gene=DARS1-AS1 |
| HLA-A | Major Histocompatibility Complex, Class I, A | Protein Coding | 45 | GC06P070103 | 1.521338 | https://www.genecards.org/cgi-bin/carddisp.pl?gene=HLA-A |
| SEPTIN9 | Septin 9 | Protein Coding | 33 | GC17P077282 | 1.520693 | https://www.genecards.org/cgi-bin/carddisp.pl?gene=SEPTIN9 |
| SCN2A | Sodium Voltage-Gated Channel Alpha Subunit 2 | Protein Coding | 45 | GC02P165194 | 1.516303 | https://www.genecards.org/cgi-bin/carddisp.pl?gene=SCN2A |
| TAC1 | Tachykinin Precursor 1 | Protein Coding | 40 | GC07P097731 | 1.514718 | https://www.genecards.org/cgi-bin/carddisp.pl?gene=TAC1 |
| NEDD8 | NEDD8 Ubiquitin Like Modifier | Protein Coding | 39 | GC14M024216 | 1.514337 | https://www.genecards.org/cgi-bin/carddisp.pl?gene=NEDD8 |
| NTRK2 | Neurotrophic Receptor Tyrosine Kinase 2 | Protein Coding | 50 | GC09P084668 | 1.513865 | https://www.genecards.org/cgi-bin/carddisp.pl?gene=NTRK2 |
| TKT | Transketolase | Protein Coding | 45 | GC03M053224 | 1.513865 | https://www.genecards.org/cgi-bin/carddisp.pl?gene=TKT |
| SREBF1 | Sterol Regulatory Element Binding Transcription Factor 1 | Protein Coding | 44 | GC17M017810 | 1.513865 | https://www.genecards.org/cgi-bin/carddisp.pl?gene=SREBF1 |
| MIR424 | MicroRNA 424 | RNA Gene | 17 | GC0XM134677 | 1.513434 | https://www.genecards.org/cgi-bin/carddisp.pl?gene=MIR424 |
| NMBR | Neuromedin B Receptor | Protein Coding | 40 | GC06M142059 | 1.511421 | https://www.genecards.org/cgi-bin/carddisp.pl?gene=NMBR |
| PSMB8 | Proteasome 20S Subunit Beta 8 | Protein Coding | 47 | GC06M032840 | 1.510957 | https://www.genecards.org/cgi-bin/carddisp.pl?gene=PSMB8 |
| PSMB9 | Proteasome 20S Subunit Beta 9 | Protein Coding | 44 | GC06P070174 | 1.510957 | https://www.genecards.org/cgi-bin/carddisp.pl?gene=PSMB9 |
| PSMB10 | Proteasome 20S Subunit Beta 10 | Protein Coding | 43 | GC16M067937 | 1.510957 | https://www.genecards.org/cgi-bin/carddisp.pl?gene=PSMB10 |
| PSMA3 | Proteasome 20S Subunit Alpha 3 | Protein Coding | 42 | GC14P058244 | 1.510957 | https://www.genecards.org/cgi-bin/carddisp.pl?gene=PSMA3 |
| PSMA1 | Proteasome 20S Subunit Alpha 1 | Protein Coding | 42 | GC11M014505 | 1.510957 | https://www.genecards.org/cgi-bin/carddisp.pl?gene=PSMA1 |
| UBB | Ubiquitin B | Protein Coding | 42 | GC17P016380 | 1.510957 | https://www.genecards.org/cgi-bin/carddisp.pl?gene=UBB |
| PSME4 | Proteasome Activator Subunit 4 | Protein Coding | 38 | GC02M053864 | 1.510957 | https://www.genecards.org/cgi-bin/carddisp.pl?gene=PSME4 |
| PSMD5 | Proteasome 26S Subunit, Non-ATPase 5 | Protein Coding | 36 | GC09M120815 | 1.510957 | https://www.genecards.org/cgi-bin/carddisp.pl?gene=PSMD5 |
| PSMA8 | Proteasome 20S Subunit Alpha 8 | Protein Coding | 35 | GC18P026133 | 1.510957 | https://www.genecards.org/cgi-bin/carddisp.pl?gene=PSMA8 |
| SEM1 | SEM1 26S Proteasome Subunit | Protein Coding | 30 | GC07M096483 | 1.510957 | https://www.genecards.org/cgi-bin/carddisp.pl?gene=SEM1 |
| PSMB11 | Proteasome Subunit Beta 11 | Protein Coding | 30 | GC14P029580 | 1.510957 | https://www.genecards.org/cgi-bin/carddisp.pl?gene=PSMB11 |
| ATG16L1 | Autophagy Related 16 Like 1 | Protein Coding | 41 | GC02P233237 | 1.510414 | https://www.genecards.org/cgi-bin/carddisp.pl?gene=ATG16L1 |
| UCN3 | Urocortin 3 | Protein Coding | 35 | GC10P005396 | 1.507213 | https://www.genecards.org/cgi-bin/carddisp.pl?gene=UCN3 |
| MME | Membrane Metalloendopeptidase | Protein Coding | 47 | GC03P155024 | 1.503956 | https://www.genecards.org/cgi-bin/carddisp.pl?gene=MME |
| TPM3 | Tropomyosin 3 | Protein Coding | 44 | GC01M154127 | 1.503335 | https://www.genecards.org/cgi-bin/carddisp.pl?gene=TPM3 |
| AKR1A1 | Aldo-Keto Reductase Family 1 Member A1 | Protein Coding | 42 | GC01P045550 | 1.502179 | https://www.genecards.org/cgi-bin/carddisp.pl?gene=AKR1A1 |
| IL17A | Interleukin 17A | Protein Coding | 39 | GC06P052186 | 1.500007 | https://www.genecards.org/cgi-bin/carddisp.pl?gene=IL17A |
| CLU | Clusterin | Protein Coding | 43 | GC08M027596 | 1.499931 | https://www.genecards.org/cgi-bin/carddisp.pl?gene=CLU |
| NKX3-1 | NK3 Homeobox 1 | Protein Coding | 39 | GC08M023678 | 1.499271 | https://www.genecards.org/cgi-bin/carddisp.pl?gene=NKX3-1 |
| CDC25A | Cell Division Cycle 25A | Protein Coding | 45 | GC03M048173 | 1.497304 | https://www.genecards.org/cgi-bin/carddisp.pl?gene=CDC25A |
| MT-CYB | Mitochondrially Encoded Cytochrome B | Protein Coding | 31 | GCMTP014749 | 1.497294 | https://www.genecards.org/cgi-bin/carddisp.pl?gene=MT-CYB |
| TP73 | Tumor Protein P73 | Protein Coding | 44 | GC01P003652 | 1.495579 | https://www.genecards.org/cgi-bin/carddisp.pl?gene=TP73 |
| SHC1 | SHC Adaptor Protein 1 | Protein Coding | 43 | GC01M154962 | 1.493322 | https://www.genecards.org/cgi-bin/carddisp.pl?gene=SHC1 |
| PCK1 | Phosphoenolpyruvate Carboxykinase 1 | Protein Coding | 46 | GC20P057561 | 1.491708 | https://www.genecards.org/cgi-bin/carddisp.pl?gene=PCK1 |
| UCN2 | Urocortin 2 | Protein Coding | 32 | GC03M048561 | 1.491674 | https://www.genecards.org/cgi-bin/carddisp.pl?gene=UCN2 |
| CAPG | Capping Actin Protein, Gelsolin Like | Protein Coding | 38 | GC02M085394 | 1.489531 | https://www.genecards.org/cgi-bin/carddisp.pl?gene=CAPG |
| NANOG | Nanog Homeobox | Protein Coding | 36 | GC12P007787 | 1.489146 | https://www.genecards.org/cgi-bin/carddisp.pl?gene=NANOG |
| MST1R | Macrophage Stimulating 1 Receptor | Protein Coding | 47 | GC03M050642 | 1.488434 | https://www.genecards.org/cgi-bin/carddisp.pl?gene=MST1R |
| TJP1 | Tight Junction Protein 1 | Protein Coding | 42 | GC15M029699 | 1.487676 | https://www.genecards.org/cgi-bin/carddisp.pl?gene=TJP1 |
| GRIN1 | Glutamate Ionotropic Receptor NMDA Type Subunit 1 | Protein Coding | 48 | GC09P137138 | 1.487589 | https://www.genecards.org/cgi-bin/carddisp.pl?gene=GRIN1 |
| MIR18A | MicroRNA 18a | RNA Gene | 18 | GC13P091509 | 1.485204 | https://www.genecards.org/cgi-bin/carddisp.pl?gene=MIR18A |
| KRT18 | Keratin 18 | Protein Coding | 46 | GC12P052948 | 1.482995 | https://www.genecards.org/cgi-bin/carddisp.pl?gene=KRT18 |
| APBA3 | Amyloid Beta Precursor Protein Binding Family A Member 3 | Protein Coding | 36 | GC19M003750 | 1.482293 | https://www.genecards.org/cgi-bin/carddisp.pl?gene=APBA3 |
| MIR19A | MicroRNA 19a | RNA Gene | 19 | GC13P091505 | 1.481949 | https://www.genecards.org/cgi-bin/carddisp.pl?gene=MIR19A |
| PCK2 | Phosphoenolpyruvate Carboxykinase 2, Mitochondrial | Protein Coding | 45 | GC14P024094 | 1.480201 | https://www.genecards.org/cgi-bin/carddisp.pl?gene=PCK2 |
| FGF11 | Fibroblast Growth Factor 11 | Protein Coding | 38 | GC17P010066 | 1.480201 | https://www.genecards.org/cgi-bin/carddisp.pl?gene=FGF11 |
| ADAM8 | ADAM Metallopeptidase Domain 8 | Protein Coding | 40 | GC10M133262 | 1.480073 | https://www.genecards.org/cgi-bin/carddisp.pl?gene=ADAM8 |
| YWHAZ | Tyrosine 3-Monooxygenase/Tryptophan 5-Monooxygenase Activation Protein Zeta | Protein Coding | 45 | GC08M100917 | 1.479139 | https://www.genecards.org/cgi-bin/carddisp.pl?gene=YWHAZ |
| RUNX3 | RUNX Family Transcription Factor 3 | Protein Coding | 42 | GC01M024899 | 1.475269 | https://www.genecards.org/cgi-bin/carddisp.pl?gene=RUNX3 |
| MAP2K6 | Mitogen-Activated Protein Kinase Kinase 6 | Protein Coding | 44 | GC17P069414 | 1.471423 | https://www.genecards.org/cgi-bin/carddisp.pl?gene=MAP2K6 |
| MIR204 | MicroRNA 204 | RNA Gene | 21 | GC09M070809 | 1.471017 | https://www.genecards.org/cgi-bin/carddisp.pl?gene=MIR204 |
| LOC110806263 | TERT 5' Regulatory Region | Biological Region | 2 | GC05P001294 | 1.469406 | https://www.genecards.org/cgi-bin/carddisp.pl?gene=LOC110806263 |
| TSLP | Thymic Stromal Lymphopoietin | Protein Coding | 35 | GC05P111070 | 1.468235 | https://www.genecards.org/cgi-bin/carddisp.pl?gene=TSLP |
| CYBA | Cytochrome B-245 Alpha Chain | Protein Coding | 45 | GC16M088643 | 1.46788 | https://www.genecards.org/cgi-bin/carddisp.pl?gene=CYBA |
| HNRNPL | Heterogeneous Nuclear Ribonucleoprotein L | Protein Coding | 38 | GC19M038836 | 1.466925 | https://www.genecards.org/cgi-bin/carddisp.pl?gene=HNRNPL |
| ABCA1 | ATP Binding Cassette Subfamily A Member 1 | Protein Coding | 45 | GC09M104781 | 1.466703 | https://www.genecards.org/cgi-bin/carddisp.pl?gene=ABCA1 |
| CFI | Complement Factor I | Protein Coding | 44 | GC04M109732 | 1.465706 | https://www.genecards.org/cgi-bin/carddisp.pl?gene=CFI |
| UQCRB | Ubiquinol-Cytochrome C Reductase Binding Protein | Protein Coding | 42 | GC08M096225 | 1.464099 | https://www.genecards.org/cgi-bin/carddisp.pl?gene=UQCRB |
| GLUL | Glutamate-Ammonia Ligase | Protein Coding | 46 | GC01M182378 | 1.463806 | https://www.genecards.org/cgi-bin/carddisp.pl?gene=GLUL |
| DUSP19 | Dual Specificity Phosphatase 19 | Protein Coding | 38 | GC02P183078 | 1.462663 | https://www.genecards.org/cgi-bin/carddisp.pl?gene=DUSP19 |
| PLD1 | Phospholipase D1 | Protein Coding | 46 | GC03M171600 | 1.461573 | https://www.genecards.org/cgi-bin/carddisp.pl?gene=PLD1 |
| ITGB2 | Integrin Subunit Beta 2 | Protein Coding | 48 | GC21M044885 | 1.45943 | https://www.genecards.org/cgi-bin/carddisp.pl?gene=ITGB2 |
| XPO1 | Exportin 1 | Protein Coding | 44 | GC02M061445 | 1.459323 | https://www.genecards.org/cgi-bin/carddisp.pl?gene=XPO1 |
| INSR | Insulin Receptor | Protein Coding | 51 | GC19M007112 | 1.459103 | https://www.genecards.org/cgi-bin/carddisp.pl?gene=INSR |
| EIF3E | Eukaryotic Translation Initiation Factor 3 Subunit E | Protein Coding | 36 | GC08M108163 | 1.458676 | https://www.genecards.org/cgi-bin/carddisp.pl?gene=EIF3E |
| CLDN3 | Claudin 3 | Protein Coding | 39 | GC07M073768 | 1.458466 | https://www.genecards.org/cgi-bin/carddisp.pl?gene=CLDN3 |
| UBE3A | Ubiquitin Protein Ligase E3A | Protein Coding | 46 | GC15M025333 | 1.458459 | https://www.genecards.org/cgi-bin/carddisp.pl?gene=UBE3A |
| HGFAC | HGF Activator | Protein Coding | 39 | GC04P003443 | 1.456414 | https://www.genecards.org/cgi-bin/carddisp.pl?gene=HGFAC |
| TBK1 | TANK Binding Kinase 1 | Protein Coding | 47 | GC12P064451 | 1.456412 | https://www.genecards.org/cgi-bin/carddisp.pl?gene=TBK1 |
| CXXC5 | CXXC Finger Protein 5 | Protein Coding | 37 | GC05P139647 | 1.455951 | https://www.genecards.org/cgi-bin/carddisp.pl?gene=CXXC5 |
| SELP | Selectin P | Protein Coding | 43 | GC01M169558 | 1.454144 | https://www.genecards.org/cgi-bin/carddisp.pl?gene=SELP |
| BLVRA | Biliverdin Reductase A | Protein Coding | 42 | GC07P043758 | 1.454144 | https://www.genecards.org/cgi-bin/carddisp.pl?gene=BLVRA |
| HES1 | Hes Family BHLH Transcription Factor 1 | Protein Coding | 41 | GC03P194136 | 1.454144 | https://www.genecards.org/cgi-bin/carddisp.pl?gene=HES1 |
| ATAD2 | ATPase Family AAA Domain Containing 2 | Protein Coding | 38 | GC08M123319 | 1.45406 | https://www.genecards.org/cgi-bin/carddisp.pl?gene=ATAD2 |
| GCG | Glucagon | Protein Coding | 39 | GC02M162142 | 1.454028 | https://www.genecards.org/cgi-bin/carddisp.pl?gene=GCG |
| MIR182 | MicroRNA 182 | RNA Gene | 20 | GC07M129770 | 1.453603 | https://www.genecards.org/cgi-bin/carddisp.pl?gene=MIR182 |
| GLI1 | GLI Family Zinc Finger 1 | Protein Coding | 45 | GC12P057460 | 1.45185 | https://www.genecards.org/cgi-bin/carddisp.pl?gene=GLI1 |
| COL4A1 | Collagen Type IV Alpha 1 Chain | Protein Coding | 46 | GC13M110148 | 1.451526 | https://www.genecards.org/cgi-bin/carddisp.pl?gene=COL4A1 |
| PSEN1 | Presenilin 1 | Protein Coding | 50 | GC14P073136 | 1.451242 | https://www.genecards.org/cgi-bin/carddisp.pl?gene=PSEN1 |
| FGFR3 | Fibroblast Growth Factor Receptor 3 | Protein Coding | 52 | GC04P001795 | 1.447808 | https://www.genecards.org/cgi-bin/carddisp.pl?gene=FGFR3 |
| USP7 | Ubiquitin Specific Peptidase 7 | Protein Coding | 46 | GC16M008892 | 1.444905 | https://www.genecards.org/cgi-bin/carddisp.pl?gene=USP7 |
| MIR675 | MicroRNA 675 | RNA Gene | 17 | GC11M002267 | 1.44416 | https://www.genecards.org/cgi-bin/carddisp.pl?gene=MIR675 |
| SCG2 | Secretogranin II | Protein Coding | 38 | GC02M223596 | 1.442377 | https://www.genecards.org/cgi-bin/carddisp.pl?gene=SCG2 |
| CCL28 | C-C Motif Chemokine Ligand 28 | Protein Coding | 38 | GC05M043356 | 1.441814 | https://www.genecards.org/cgi-bin/carddisp.pl?gene=CCL28 |
| SLC12A2 | Solute Carrier Family 12 Member 2 | Protein Coding | 47 | GC05P128083 | 1.440475 | https://www.genecards.org/cgi-bin/carddisp.pl?gene=SLC12A2 |
| RUNX1 | RUNX Family Transcription Factor 1 | Protein Coding | 46 | GC21M034787 | 1.439613 | https://www.genecards.org/cgi-bin/carddisp.pl?gene=RUNX1 |
| WWTR1 | WW Domain Containing Transcription Regulator 1 | Protein Coding | 39 | GC03M149517 | 1.439349 | https://www.genecards.org/cgi-bin/carddisp.pl?gene=WWTR1 |
| GYS1 | Glycogen Synthase 1 | Protein Coding | 47 | GC19M057393 | 1.438285 | https://www.genecards.org/cgi-bin/carddisp.pl?gene=GYS1 |
| CYP2S1 | Cytochrome P450 Family 2 Subfamily S Member 1 | Protein Coding | 41 | GC19P041193 | 1.437453 | https://www.genecards.org/cgi-bin/carddisp.pl?gene=CYP2S1 |
| CAPN1 | Calpain 1 | Protein Coding | 47 | GC11P068073 | 1.436825 | https://www.genecards.org/cgi-bin/carddisp.pl?gene=CAPN1 |
| F2RL1 | F2R Like Trypsin Receptor 1 | Protein Coding | 44 | GC05P076818 | 1.436473 | https://www.genecards.org/cgi-bin/carddisp.pl?gene=F2RL1 |
| NCOA3 | Nuclear Receptor Coactivator 3 | Protein Coding | 44 | GC20P047501 | 1.434853 | https://www.genecards.org/cgi-bin/carddisp.pl?gene=NCOA3 |
| NPM1 | Nucleophosmin 1 | Protein Coding | 47 | GC05P171387 | 1.43083 | https://www.genecards.org/cgi-bin/carddisp.pl?gene=NPM1 |
| HDAC6 | Histone Deacetylase 6 | Protein Coding | 50 | GC0XP048801 | 1.429003 | https://www.genecards.org/cgi-bin/carddisp.pl?gene=HDAC6 |
| SOCS3 | Suppressor Of Cytokine Signaling 3 | Protein Coding | 42 | GC17M078356 | 1.429003 | https://www.genecards.org/cgi-bin/carddisp.pl?gene=SOCS3 |
| E2F3 | E2F Transcription Factor 3 | Protein Coding | 40 | GC06P020402 | 1.428841 | https://www.genecards.org/cgi-bin/carddisp.pl?gene=E2F3 |
| PTPN3 | Protein Tyrosine Phosphatase Non-Receptor Type 3 | Protein Coding | 43 | GC09M109375 | 1.427963 | https://www.genecards.org/cgi-bin/carddisp.pl?gene=PTPN3 |
| PROX1 | Prospero Homeobox 1 | Protein Coding | 41 | GC01P213983 | 1.427963 | https://www.genecards.org/cgi-bin/carddisp.pl?gene=PROX1 |
| MIR124-2 | MicroRNA 124-2 | RNA Gene | 18 | GC08P064379 | 1.427075 | https://www.genecards.org/cgi-bin/carddisp.pl?gene=MIR124-2 |
| ACACA | Acetyl-CoA Carboxylase Alpha | Protein Coding | 47 | GC17M037084 | 1.426127 | https://www.genecards.org/cgi-bin/carddisp.pl?gene=ACACA |
| ESRRG | Estrogen Related Receptor Gamma | Protein Coding | 44 | GC01M216503 | 1.426127 | https://www.genecards.org/cgi-bin/carddisp.pl?gene=ESRRG |
| CDK7 | Cyclin Dependent Kinase 7 | Protein Coding | 44 | GC05P069308 | 1.426042 | https://www.genecards.org/cgi-bin/carddisp.pl?gene=CDK7 |
| PRMT1 | Protein Arginine Methyltransferase 1 | Protein Coding | 46 | GC19P049675 | 1.424401 | https://www.genecards.org/cgi-bin/carddisp.pl?gene=PRMT1 |
| PTGES | Prostaglandin E Synthase | Protein Coding | 39 | GC09M129738 | 1.424401 | https://www.genecards.org/cgi-bin/carddisp.pl?gene=PTGES |
| TGFBR1 | Transforming Growth Factor Beta Receptor 1 | Protein Coding | 50 | GC09P099104 | 1.420209 | https://www.genecards.org/cgi-bin/carddisp.pl?gene=TGFBR1 |
| ENTPD1 | Ectonucleoside Triphosphate Diphosphohydrolase 1 | Protein Coding | 44 | GC10P095711 | 1.420209 | https://www.genecards.org/cgi-bin/carddisp.pl?gene=ENTPD1 |
| DNMT3A | DNA Methyltransferase 3 Alpha | Protein Coding | 49 | GC02M025228 | 1.418855 | https://www.genecards.org/cgi-bin/carddisp.pl?gene=DNMT3A |
| MIR140 | MicroRNA 140 | RNA Gene | 22 | GC16P069934 | 1.418855 | https://www.genecards.org/cgi-bin/carddisp.pl?gene=MIR140 |
| ADCYAP1 | Adenylate Cyclase Activating Polypeptide 1 | Protein Coding | 40 | GC18P000895 | 1.417846 | https://www.genecards.org/cgi-bin/carddisp.pl?gene=ADCYAP1 |
| MAP1LC3A | Microtubule Associated Protein 1 Light Chain 3 Alpha | Protein Coding | 40 | GC20P034546 | 1.417846 | https://www.genecards.org/cgi-bin/carddisp.pl?gene=MAP1LC3A |
| MSH2 | MutS Homolog 2 | Protein Coding | 46 | GC02P047402 | 1.416731 | https://www.genecards.org/cgi-bin/carddisp.pl?gene=MSH2 |
| PIGF | Phosphatidylinositol Glycan Anchor Biosynthesis Class F | Protein Coding | 38 | GC02M046580 | 1.416731 | https://www.genecards.org/cgi-bin/carddisp.pl?gene=PIGF |
| CNOT1 | CCR4-NOT Transcription Complex Subunit 1 | Protein Coding | 39 | GC16M058519 | 1.416722 | https://www.genecards.org/cgi-bin/carddisp.pl?gene=CNOT1 |
| YY1 | YY1 Transcription Factor | Protein Coding | 46 | GC14P100238 | 1.414026 | https://www.genecards.org/cgi-bin/carddisp.pl?gene=YY1 |
| CCR5 | C-C Motif Chemokine Receptor 5 | Protein Coding | 44 | GC03P046383 | 1.411766 | https://www.genecards.org/cgi-bin/carddisp.pl?gene=CCR5 |
| ERCC3 | ERCC Excision Repair 3, TFIIH Core Complex Helicase Subunit | Protein Coding | 46 | GC02M127257 | 1.411005 | https://www.genecards.org/cgi-bin/carddisp.pl?gene=ERCC3 |
| CHEK2 | Checkpoint Kinase 2 | Protein Coding | 51 | GC22M028687 | 1.409082 | https://www.genecards.org/cgi-bin/carddisp.pl?gene=CHEK2 |
| MAP4 | Microtubule Associated Protein 4 | Protein Coding | 40 | GC03M047850 | 1.408457 | https://www.genecards.org/cgi-bin/carddisp.pl?gene=MAP4 |
| PARK7 | Parkinsonism Associated Deglycase | Protein Coding | 43 | GC01P008053 | 1.408211 | https://www.genecards.org/cgi-bin/carddisp.pl?gene=PARK7 |
| NR1H2 | Nuclear Receptor Subfamily 1 Group H Member 2 | Protein Coding | 46 | GC19P050329 | 1.408121 | https://www.genecards.org/cgi-bin/carddisp.pl?gene=NR1H2 |
| GRK2 | G Protein-Coupled Receptor Kinase 2 | Protein Coding | 36 | GC11P067266 | 1.407886 | https://www.genecards.org/cgi-bin/carddisp.pl?gene=GRK2 |
| HLA-C | Major Histocompatibility Complex, Class I, C | Protein Coding | 43 | GC06M056461 | 1.407666 | https://www.genecards.org/cgi-bin/carddisp.pl?gene=HLA-C |
| CSNK1D | Casein Kinase 1 Delta | Protein Coding | 48 | GC17M082239 | 1.405337 | https://www.genecards.org/cgi-bin/carddisp.pl?gene=CSNK1D |
| GSTP1 | Glutathione S-Transferase Pi 1 | Protein Coding | 48 | GC11P067583 | 1.403274 | https://www.genecards.org/cgi-bin/carddisp.pl?gene=GSTP1 |
| UGP2 | UDP-Glucose Pyrophosphorylase 2 | Protein Coding | 40 | GC02P063840 | 1.402079 | https://www.genecards.org/cgi-bin/carddisp.pl?gene=UGP2 |
| CDK1 | Cyclin Dependent Kinase 1 | Protein Coding | 43 | GC10P060772 | 1.401386 | https://www.genecards.org/cgi-bin/carddisp.pl?gene=CDK1 |
| CYP19A1 | Cytochrome P450 Family 19 Subfamily A Member 1 | Protein Coding | 47 | GC15M051208 | 1.399272 | https://www.genecards.org/cgi-bin/carddisp.pl?gene=CYP19A1 |
| TLR6 | Toll Like Receptor 6 | Protein Coding | 42 | GC04M038828 | 1.396358 | https://www.genecards.org/cgi-bin/carddisp.pl?gene=TLR6 |
| CALCA | Calcitonin Related Polypeptide Alpha | Protein Coding | 41 | GC11M014945 | 1.396271 | https://www.genecards.org/cgi-bin/carddisp.pl?gene=CALCA |
| DNAH8 | Dynein Axonemal Heavy Chain 8 | Protein Coding | 36 | GC06P070310 | 1.396271 | https://www.genecards.org/cgi-bin/carddisp.pl?gene=DNAH8 |
| PRMT5 | Protein Arginine Methyltransferase 5 | Protein Coding | 41 | GC14M022920 | 1.396214 | https://www.genecards.org/cgi-bin/carddisp.pl?gene=PRMT5 |
| HTR2A | 5-Hydroxytryptamine Receptor 2A | Protein Coding | 46 | GC13M046831 | 1.396027 | https://www.genecards.org/cgi-bin/carddisp.pl?gene=HTR2A |
| ERRFI1 | ERBB Receptor Feedback Inhibitor 1 | Protein Coding | 36 | GC01M008004 | 1.396027 | https://www.genecards.org/cgi-bin/carddisp.pl?gene=ERRFI1 |
| HAS2-AS1 | HAS2 Antisense RNA 1 | RNA Gene | 18 | GC08P121639 | 1.396027 | https://www.genecards.org/cgi-bin/carddisp.pl?gene=HAS2-AS1 |
| TNKS2 | Tankyrase 2 | Protein Coding | 42 | GC10P091798 | 1.393639 | https://www.genecards.org/cgi-bin/carddisp.pl?gene=TNKS2 |
| MSR1 | Macrophage Scavenger Receptor 1 | Protein Coding | 43 | GC08M016107 | 1.392636 | https://www.genecards.org/cgi-bin/carddisp.pl?gene=MSR1 |
| PTPRZ1 | Protein Tyrosine Phosphatase Receptor Type Z1 | Protein Coding | 40 | GC07P121873 | 1.392636 | https://www.genecards.org/cgi-bin/carddisp.pl?gene=PTPRZ1 |
| SLC35C2 | Solute Carrier Family 35 Member C2 | Protein Coding | 36 | GC20M046345 | 1.392424 | https://www.genecards.org/cgi-bin/carddisp.pl?gene=SLC35C2 |
| ANXA2 | Annexin A2 | Protein Coding | 46 | GC15M060347 | 1.390541 | https://www.genecards.org/cgi-bin/carddisp.pl?gene=ANXA2 |
| CIAO3 | Cytosolic Iron-Sulfur Assembly Component 3 | Protein Coding | 26 | GC16M005715 | 1.390362 | https://www.genecards.org/cgi-bin/carddisp.pl?gene=CIAO3 |
| SLC1A2 | Solute Carrier Family 1 Member 2 | Protein Coding | 47 | GC11M035252 | 1.389829 | https://www.genecards.org/cgi-bin/carddisp.pl?gene=SLC1A2 |
| XIAP | X-Linked Inhibitor Of Apoptosis | Protein Coding | 47 | GC0XP123859 | 1.389829 | https://www.genecards.org/cgi-bin/carddisp.pl?gene=XIAP |
| NOTCH4 | Notch Receptor 4 | Protein Coding | 43 | GC06M056539 | 1.389829 | https://www.genecards.org/cgi-bin/carddisp.pl?gene=NOTCH4 |
| WSB1 | WD Repeat And SOCS Box Containing 1 | Protein Coding | 39 | GC17P027294 | 1.389829 | https://www.genecards.org/cgi-bin/carddisp.pl?gene=WSB1 |
| MDK | Midkine | Protein Coding | 41 | GC11P046380 | 1.38829 | https://www.genecards.org/cgi-bin/carddisp.pl?gene=MDK |
| PLOD1 | Procollagen-Lysine,2-Oxoglutarate 5-Dioxygenase 1 | Protein Coding | 40 | GC01P011934 | 1.386285 | https://www.genecards.org/cgi-bin/carddisp.pl?gene=PLOD1 |
| LTA | Lymphotoxin Alpha | Protein Coding | 39 | GC06P070141 | 1.384086 | https://www.genecards.org/cgi-bin/carddisp.pl?gene=LTA |
| EIF2AK4 | Eukaryotic Translation Initiation Factor 2 Alpha Kinase 4 | Protein Coding | 43 | GC15P039934 | 1.383558 | https://www.genecards.org/cgi-bin/carddisp.pl?gene=EIF2AK4 |
| TPM2 | Tropomyosin 2 | Protein Coding | 43 | GC09M035672 | 1.383558 | https://www.genecards.org/cgi-bin/carddisp.pl?gene=TPM2 |
| TRH | Thyrotropin Releasing Hormone | Protein Coding | 40 | GC03P129974 | 1.383394 | https://www.genecards.org/cgi-bin/carddisp.pl?gene=TRH |
| MICA | MHC Class I Polypeptide-Related Sequence A | Protein Coding | 36 | GC06P031399 | 1.380141 | https://www.genecards.org/cgi-bin/carddisp.pl?gene=MICA |
| CSK | C-Terminal Src Kinase | Protein Coding | 45 | GC15P074782 | 1.380104 | https://www.genecards.org/cgi-bin/carddisp.pl?gene=CSK |
| TRPM7 | Transient Receptor Potential Cation Channel Subfamily M Member 7 | Protein Coding | 43 | GC15M050552 | 1.380104 | https://www.genecards.org/cgi-bin/carddisp.pl?gene=TRPM7 |
| CS | Citrate Synthase | Protein Coding | 42 | GC12M056271 | 1.380104 | https://www.genecards.org/cgi-bin/carddisp.pl?gene=CS |
| ITPR1 | Inositol 1,4,5-Trisphosphate Receptor Type 1 | Protein Coding | 46 | GC03P004486 | 1.37776 | https://www.genecards.org/cgi-bin/carddisp.pl?gene=ITPR1 |
| CD248 | CD248 Molecule | Protein Coding | 36 | GC11M066314 | 1.377557 | https://www.genecards.org/cgi-bin/carddisp.pl?gene=CD248 |
| SMG1 | SMG1 Nonsense Mediated MRNA Decay Associated PI3K Related Kinase | Protein Coding | 39 | GC16M018990 | 1.376781 | https://www.genecards.org/cgi-bin/carddisp.pl?gene=SMG1 |
| HNRNPA2B1 | Heterogeneous Nuclear Ribonucleoprotein A2/B1 | Protein Coding | 44 | GC07M026174 | 1.376498 | https://www.genecards.org/cgi-bin/carddisp.pl?gene=HNRNPA2B1 |
| NKX2-1 | NK2 Homeobox 1 | Protein Coding | 44 | GC14M036516 | 1.375627 | https://www.genecards.org/cgi-bin/carddisp.pl?gene=NKX2-1 |
| MIR30A | MicroRNA 30a | RNA Gene | 19 | GC06M071403 | 1.372849 | https://www.genecards.org/cgi-bin/carddisp.pl?gene=MIR30A |
| DRD4 | Dopamine Receptor D4 | Protein Coding | 44 | GC11P001360 | 1.371814 | https://www.genecards.org/cgi-bin/carddisp.pl?gene=DRD4 |
| TUG1 | Taurine Up-Regulated 1 | Protein Coding | 23 | GC22P030969 | 1.371814 | https://www.genecards.org/cgi-bin/carddisp.pl?gene=TUG1 |
| ACOX1 | Acyl-CoA Oxidase 1 | Protein Coding | 44 | GC17M075941 | 1.370713 | https://www.genecards.org/cgi-bin/carddisp.pl?gene=ACOX1 |
| CXCR2 | C-X-C Motif Chemokine Receptor 2 | Protein Coding | 47 | GC02P218125 | 1.37007 | https://www.genecards.org/cgi-bin/carddisp.pl?gene=CXCR2 |
| FMR1 | FMRP Translational Regulator 1 | Protein Coding | 42 | GC0XP147933 | 1.369389 | https://www.genecards.org/cgi-bin/carddisp.pl?gene=FMR1 |
| PRKCZ | Protein Kinase C Zeta | Protein Coding | 47 | GC01P002050 | 1.369172 | https://www.genecards.org/cgi-bin/carddisp.pl?gene=PRKCZ |
| PXN | Paxillin | Protein Coding | 43 | GC12M120210 | 1.369172 | https://www.genecards.org/cgi-bin/carddisp.pl?gene=PXN |
| CFH | Complement Factor H | Protein Coding | 43 | GC01P196621 | 1.36484 | https://www.genecards.org/cgi-bin/carddisp.pl?gene=CFH |
| RYR2 | Ryanodine Receptor 2 | Protein Coding | 46 | GC01P237042 | 1.364206 | https://www.genecards.org/cgi-bin/carddisp.pl?gene=RYR2 |
| NPPC | Natriuretic Peptide C | Protein Coding | 39 | GC02M231921 | 1.364206 | https://www.genecards.org/cgi-bin/carddisp.pl?gene=NPPC |
| NFAT5 | Nuclear Factor Of Activated T Cells 5 | Protein Coding | 41 | GC16P069565 | 1.361325 | https://www.genecards.org/cgi-bin/carddisp.pl?gene=NFAT5 |
| SMARCA2 | SWI/SNF Related, Matrix Associated, Actin Dependent Regulator Of Chromatin, Subfamily A, Member 2 | Protein Coding | 47 | GC09P001980 | 1.359729 | https://www.genecards.org/cgi-bin/carddisp.pl?gene=SMARCA2 |
| TP63 | Tumor Protein P63 | Protein Coding | 46 | GC03P189598 | 1.359729 | https://www.genecards.org/cgi-bin/carddisp.pl?gene=TP63 |
| KLF8 | Kruppel Like Factor 8 | Protein Coding | 37 | GC0XP055909 | 1.359729 | https://www.genecards.org/cgi-bin/carddisp.pl?gene=KLF8 |
| MIR223 | MicroRNA 223 | RNA Gene | 21 | GC0XP066018 | 1.359729 | https://www.genecards.org/cgi-bin/carddisp.pl?gene=MIR223 |
| CD38 | CD38 Molecule | Protein Coding | 43 | GC04P016367 | 1.358505 | https://www.genecards.org/cgi-bin/carddisp.pl?gene=CD38 |
| IL11 | Interleukin 11 | Protein Coding | 38 | GC19M055364 | 1.358333 | https://www.genecards.org/cgi-bin/carddisp.pl?gene=IL11 |
| STK26 | Serine/Threonine Kinase 26 | Protein Coding | 39 | GC0XP132023 | 1.358292 | https://www.genecards.org/cgi-bin/carddisp.pl?gene=STK26 |
| YBX1 | Y-Box Binding Protein 1 | Protein Coding | 37 | GC01P042682 | 1.357685 | https://www.genecards.org/cgi-bin/carddisp.pl?gene=YBX1 |
| APOA1 | Apolipoprotein A1 | Protein Coding | 46 | GC11M116835 | 1.357014 | https://www.genecards.org/cgi-bin/carddisp.pl?gene=APOA1 |
| RETN | Resistin | Protein Coding | 41 | GC19P007669 | 1.355064 | https://www.genecards.org/cgi-bin/carddisp.pl?gene=RETN |
| DIO3 | Iodothyronine Deiodinase 3 | Protein Coding | 39 | GC14P107983 | 1.355064 | https://www.genecards.org/cgi-bin/carddisp.pl?gene=DIO3 |
| ADCY10 | Adenylate Cyclase 10 | Protein Coding | 43 | GC01M167809 | 1.35457 | https://www.genecards.org/cgi-bin/carddisp.pl?gene=ADCY10 |
| HLF | HLF Transcription Factor, PAR BZIP Family Member | Protein Coding | 38 | GC17P055264 | 1.354135 | https://www.genecards.org/cgi-bin/carddisp.pl?gene=HLF |
| TMPRSS6 | Transmembrane Serine Protease 6 | Protein Coding | 42 | GC22M037066 | 1.352937 | https://www.genecards.org/cgi-bin/carddisp.pl?gene=TMPRSS6 |
| CA3 | Carbonic Anhydrase 3 | Protein Coding | 39 | GC08P085373 | 1.352937 | https://www.genecards.org/cgi-bin/carddisp.pl?gene=CA3 |
| TLR3 | Toll Like Receptor 3 | Protein Coding | 49 | GC04P186059 | 1.352154 | https://www.genecards.org/cgi-bin/carddisp.pl?gene=TLR3 |
| CGB5 | Chorionic Gonadotropin Subunit Beta 5 | Protein Coding | 30 | GC19P049043 | 1.352085 | https://www.genecards.org/cgi-bin/carddisp.pl?gene=CGB5 |
| SAT2 | Spermidine/Spermine N1-Acetyltransferase Family Member 2 | Protein Coding | 36 | GC17M007626 | 1.351968 | https://www.genecards.org/cgi-bin/carddisp.pl?gene=SAT2 |
| H3C1 | H3 Clustered Histone 1 | Protein Coding | 31 | GC06P066932 | 1.350926 | https://www.genecards.org/cgi-bin/carddisp.pl?gene=H3C1 |
| PTPN1 | Protein Tyrosine Phosphatase Non-Receptor Type 1 | Protein Coding | 48 | GC20P050510 | 1.350004 | https://www.genecards.org/cgi-bin/carddisp.pl?gene=PTPN1 |
| MFN2 | Mitofusin 2 | Protein Coding | 46 | GC01P011980 | 1.348943 | https://www.genecards.org/cgi-bin/carddisp.pl?gene=MFN2 |
| MIR130A | MicroRNA 130a | RNA Gene | 20 | GC11P057641 | 1.348943 | https://www.genecards.org/cgi-bin/carddisp.pl?gene=MIR130A |
| PRDX6 | Peroxiredoxin 6 | Protein Coding | 44 | GC01P173477 | 1.345624 | https://www.genecards.org/cgi-bin/carddisp.pl?gene=PRDX6 |
| SOST | Sclerostin | Protein Coding | 41 | GC17M043753 | 1.345624 | https://www.genecards.org/cgi-bin/carddisp.pl?gene=SOST |
| HOTTIP | HOXA Distal Transcript Antisense RNA | RNA Gene | 22 | GC07P027198 | 1.345624 | https://www.genecards.org/cgi-bin/carddisp.pl?gene=HOTTIP |
| RSF1 | Remodeling And Spacing Factor 1 | Protein Coding | 34 | GC11M080439 | 1.344864 | https://www.genecards.org/cgi-bin/carddisp.pl?gene=RSF1 |
| MYOCD | Myocardin | Protein Coding | 40 | GC17P012665 | 1.344485 | https://www.genecards.org/cgi-bin/carddisp.pl?gene=MYOCD |
| PPP1R8 | Protein Phosphatase 1 Regulatory Subunit 8 | Protein Coding | 38 | GC01P027830 | 1.343936 | https://www.genecards.org/cgi-bin/carddisp.pl?gene=PPP1R8 |
| KLF6 | Kruppel Like Factor 6 | Protein Coding | 42 | GC10M003779 | 1.341981 | https://www.genecards.org/cgi-bin/carddisp.pl?gene=KLF6 |
| BBC3 | BCL2 Binding Component 3 | Protein Coding | 39 | GC19M047220 | 1.341833 | https://www.genecards.org/cgi-bin/carddisp.pl?gene=BBC3 |
| CYP2J2 | Cytochrome P450 Family 2 Subfamily J Member 2 | Protein Coding | 42 | GC01M059893 | 1.340575 | https://www.genecards.org/cgi-bin/carddisp.pl?gene=CYP2J2 |
| ARG1 | Arginase 1 | Protein Coding | 47 | GC06P131473 | 1.33997 | https://www.genecards.org/cgi-bin/carddisp.pl?gene=ARG1 |
| PRDX5 | Peroxiredoxin 5 | Protein Coding | 43 | GC11P064317 | 1.33997 | https://www.genecards.org/cgi-bin/carddisp.pl?gene=PRDX5 |
| MIR10B | MicroRNA 10b | RNA Gene | 21 | GC02P176150 | 1.33997 | https://www.genecards.org/cgi-bin/carddisp.pl?gene=MIR10B |
| ATF3 | Activating Transcription Factor 3 | Protein Coding | 42 | GC01P212565 | 1.339076 | https://www.genecards.org/cgi-bin/carddisp.pl?gene=ATF3 |
| CD55 | CD55 Molecule (Cromer Blood Group) | Protein Coding | 44 | GC01P207321 | 1.338624 | https://www.genecards.org/cgi-bin/carddisp.pl?gene=CD55 |
| THBD | Thrombomodulin | Protein Coding | 42 | GC20M023026 | 1.338297 | https://www.genecards.org/cgi-bin/carddisp.pl?gene=THBD |
| TRIP11 | Thyroid Hormone Receptor Interactor 11 | Protein Coding | 40 | GC14M091965 | 1.335608 | https://www.genecards.org/cgi-bin/carddisp.pl?gene=TRIP11 |
| COMT | Catechol-O-Methyltransferase | Protein Coding | 48 | GC22P019941 | 1.333337 | https://www.genecards.org/cgi-bin/carddisp.pl?gene=COMT |
| TFF1 | Trefoil Factor 1 | Protein Coding | 42 | GC21M042362 | 1.329595 | https://www.genecards.org/cgi-bin/carddisp.pl?gene=TFF1 |
| AXL | AXL Receptor Tyrosine Kinase | Protein Coding | 49 | GC19P041219 | 1.329122 | https://www.genecards.org/cgi-bin/carddisp.pl?gene=AXL |
| PPARD | Peroxisome Proliferator Activated Receptor Delta | Protein Coding | 45 | GC06P070267 | 1.329014 | https://www.genecards.org/cgi-bin/carddisp.pl?gene=PPARD |
| NOP53 | NOP53 Ribosome Biogenesis Factor | Protein Coding | 27 | GC19P047749 | 1.329014 | https://www.genecards.org/cgi-bin/carddisp.pl?gene=NOP53 |
| TNFRSF10A | TNF Receptor Superfamily Member 10a | Protein Coding | 42 | GC08M023190 | 1.328925 | https://www.genecards.org/cgi-bin/carddisp.pl?gene=TNFRSF10A |
| MIR143 | MicroRNA 143 | RNA Gene | 22 | GC05P149410 | 1.328482 | https://www.genecards.org/cgi-bin/carddisp.pl?gene=MIR143 |
| JAK1 | Janus Kinase 1 | Protein Coding | 51 | GC01M064833 | 1.326997 | https://www.genecards.org/cgi-bin/carddisp.pl?gene=JAK1 |
| LYN | LYN Proto-Oncogene, Src Family Tyrosine Kinase | Protein Coding | 47 | GC08P055879 | 1.326974 | https://www.genecards.org/cgi-bin/carddisp.pl?gene=LYN |
| MED13L | Mediator Complex Subunit 13L | Protein Coding | 38 | GC12M115953 | 1.326881 | https://www.genecards.org/cgi-bin/carddisp.pl?gene=MED13L |
| HCRT | Hypocretin Neuropeptide Precursor | Protein Coding | 39 | GC17M042185 | 1.325128 | https://www.genecards.org/cgi-bin/carddisp.pl?gene=HCRT |
| HIF1A-AS1 | HIF1A Antisense RNA 1 | RNA Gene | 15 | GC14M061681 | 1.325028 | https://www.genecards.org/cgi-bin/carddisp.pl?gene=HIF1A-AS1 |
| PDPK1 | 3-Phosphoinositide Dependent Protein Kinase 1 | Protein Coding | 47 | GC16P002537 | 1.323353 | https://www.genecards.org/cgi-bin/carddisp.pl?gene=PDPK1 |
| FGF7 | Fibroblast Growth Factor 7 | Protein Coding | 39 | GC15P049423 | 1.323353 | https://www.genecards.org/cgi-bin/carddisp.pl?gene=FGF7 |
| ANKRD1 | Ankyrin Repeat Domain 1 | Protein Coding | 40 | GC10M090912 | 1.322758 | https://www.genecards.org/cgi-bin/carddisp.pl?gene=ANKRD1 |
| NGFR | Nerve Growth Factor Receptor | Protein Coding | 43 | GC17P049495 | 1.318843 | https://www.genecards.org/cgi-bin/carddisp.pl?gene=NGFR |
| SYP | Synaptophysin | Protein Coding | 42 | GC0XM049187 | 1.318364 | https://www.genecards.org/cgi-bin/carddisp.pl?gene=SYP |
| RAMP1 | Receptor Activity Modifying Protein 1 | Protein Coding | 42 | GC02P237858 | 1.318364 | https://www.genecards.org/cgi-bin/carddisp.pl?gene=RAMP1 |
| TXNRD1 | Thioredoxin Reductase 1 | Protein Coding | 44 | GC12P104215 | 1.318108 | https://www.genecards.org/cgi-bin/carddisp.pl?gene=TXNRD1 |
| ACHE | Acetylcholinesterase (Cartwright Blood Group) | Protein Coding | 44 | GC07M100889 | 1.318045 | https://www.genecards.org/cgi-bin/carddisp.pl?gene=ACHE |
| ACVRL1 | Activin A Receptor Like Type 1 | Protein Coding | 47 | GC12P051906 | 1.31741 | https://www.genecards.org/cgi-bin/carddisp.pl?gene=ACVRL1 |
| UCP3 | Uncoupling Protein 3 | Protein Coding | 42 | GC11M074000 | 1.31741 | https://www.genecards.org/cgi-bin/carddisp.pl?gene=UCP3 |
| TNFRSF10D | TNF Receptor Superfamily Member 10d | Protein Coding | 40 | GC08M023135 | 1.316732 | https://www.genecards.org/cgi-bin/carddisp.pl?gene=TNFRSF10D |
| ALOX5AP | Arachidonate 5-Lipoxygenase Activating Protein | Protein Coding | 42 | GC13P030713 | 1.316019 | https://www.genecards.org/cgi-bin/carddisp.pl?gene=ALOX5AP |
| BIRC3 | Baculoviral IAP Repeat Containing 3 | Protein Coding | 44 | GC11P102317 | 1.315667 | https://www.genecards.org/cgi-bin/carddisp.pl?gene=BIRC3 |
| NES | Nestin | Protein Coding | 38 | GC01M156668 | 1.315667 | https://www.genecards.org/cgi-bin/carddisp.pl?gene=NES |
| PLCG1 | Phospholipase C Gamma 1 | Protein Coding | 46 | GC20P041136 | 1.313925 | https://www.genecards.org/cgi-bin/carddisp.pl?gene=PLCG1 |
| HSF2 | Heat Shock Transcription Factor 2 | Protein Coding | 42 | GC06P122399 | 1.311534 | https://www.genecards.org/cgi-bin/carddisp.pl?gene=HSF2 |
| MBL2 | Mannose Binding Lectin 2 | Protein Coding | 44 | GC10M052760 | 1.311417 | https://www.genecards.org/cgi-bin/carddisp.pl?gene=MBL2 |
| CASP6 | Caspase 6 | Protein Coding | 46 | GC04M109688 | 1.310655 | https://www.genecards.org/cgi-bin/carddisp.pl?gene=CASP6 |
| EFNA3 | Ephrin A3 | Protein Coding | 42 | GC01P155078 | 1.309419 | https://www.genecards.org/cgi-bin/carddisp.pl?gene=EFNA3 |
| MT2A | Metallothionein 2A | Protein Coding | 40 | GC16P056868 | 1.309419 | https://www.genecards.org/cgi-bin/carddisp.pl?gene=MT2A |
| SF3B4 | Splicing Factor 3b Subunit 4 | Protein Coding | 42 | GC01M149923 | 1.308268 | https://www.genecards.org/cgi-bin/carddisp.pl?gene=SF3B4 |
| PDCD10 | Programmed Cell Death 10 | Protein Coding | 40 | GC03M167683 | 1.308268 | https://www.genecards.org/cgi-bin/carddisp.pl?gene=PDCD10 |
| PFKFB2 | 6-Phosphofructo-2-Kinase/Fructose-2,6-Biphosphatase 2 | Protein Coding | 39 | GC01P207034 | 1.308196 | https://www.genecards.org/cgi-bin/carddisp.pl?gene=PFKFB2 |
| NDRG3 | NDRG Family Member 3 | Protein Coding | 35 | GC20M036651 | 1.308196 | https://www.genecards.org/cgi-bin/carddisp.pl?gene=NDRG3 |
| LATS2 | Large Tumor Suppressor Kinase 2 | Protein Coding | 43 | GC13M020973 | 1.307668 | https://www.genecards.org/cgi-bin/carddisp.pl?gene=LATS2 |
| VIP | Vasoactive Intestinal Peptide | Protein Coding | 43 | GC06P152750 | 1.307668 | https://www.genecards.org/cgi-bin/carddisp.pl?gene=VIP |
| GPT | Glutamic--Pyruvic Transaminase | Protein Coding | 39 | GC08P144502 | 1.307668 | https://www.genecards.org/cgi-bin/carddisp.pl?gene=GPT |
| ODC1 | Ornithine Decarboxylase 1 | Protein Coding | 46 | GC02M010432 | 1.307237 | https://www.genecards.org/cgi-bin/carddisp.pl?gene=ODC1 |
| SIN3A | SIN3 Transcription Regulator Family Member A | Protein Coding | 44 | GC15M075369 | 1.307237 | https://www.genecards.org/cgi-bin/carddisp.pl?gene=SIN3A |
| PTGER1 | Prostaglandin E Receptor 1 | Protein Coding | 39 | GC19M014444 | 1.307237 | https://www.genecards.org/cgi-bin/carddisp.pl?gene=PTGER1 |
| CCL20 | C-C Motif Chemokine Ligand 20 | Protein Coding | 41 | GC02P227829 | 1.306483 | https://www.genecards.org/cgi-bin/carddisp.pl?gene=CCL20 |
| DCD | Dermcidin | Protein Coding | 36 | GC12M054644 | 1.306483 | https://www.genecards.org/cgi-bin/carddisp.pl?gene=DCD |
| OGDH | Oxoglutarate Dehydrogenase | Protein Coding | 43 | GC07P044606 | 1.303594 | https://www.genecards.org/cgi-bin/carddisp.pl?gene=OGDH |
| DNAJA2 | DnaJ Heat Shock Protein Family (Hsp40) Member A2 | Protein Coding | 38 | GC16M046955 | 1.303594 | https://www.genecards.org/cgi-bin/carddisp.pl?gene=DNAJA2 |
| KLF15 | Kruppel Like Factor 15 | Protein Coding | 40 | GC03M126293 | 1.302841 | https://www.genecards.org/cgi-bin/carddisp.pl?gene=KLF15 |
| ESM1 | Endothelial Cell Specific Molecule 1 | Protein Coding | 38 | GC05M054977 | 1.301137 | https://www.genecards.org/cgi-bin/carddisp.pl?gene=ESM1 |
| PPP1CA | Protein Phosphatase 1 Catalytic Subunit Alpha | Protein Coding | 46 | GC11M080119 | 1.301105 | https://www.genecards.org/cgi-bin/carddisp.pl?gene=PPP1CA |
| WNT1 | Wnt Family Member 1 | Protein Coding | 43 | GC12P049053 | 1.301105 | https://www.genecards.org/cgi-bin/carddisp.pl?gene=WNT1 |
| XRCC5 | X-Ray Repair Cross Complementing 5 | Protein Coding | 42 | GC02P216107 | 1.301105 | https://www.genecards.org/cgi-bin/carddisp.pl?gene=XRCC5 |
| KPNA1 | Karyopherin Subunit Alpha 1 | Protein Coding | 40 | GC03M122421 | 1.301105 | https://www.genecards.org/cgi-bin/carddisp.pl?gene=KPNA1 |
| NODAL | Nodal Growth Differentiation Factor | Protein Coding | 40 | GC10M070431 | 1.301105 | https://www.genecards.org/cgi-bin/carddisp.pl?gene=NODAL |
| UCN | Urocortin | Protein Coding | 35 | GC02M027308 | 1.301105 | https://www.genecards.org/cgi-bin/carddisp.pl?gene=UCN |
| CFTR | CF Transmembrane Conductance Regulator | Protein Coding | 48 | GC07P117287 | 1.300216 | https://www.genecards.org/cgi-bin/carddisp.pl?gene=CFTR |
| SUV39H1 | SUV39H1 Histone Lysine Methyltransferase | Protein Coding | 42 | GC0XP049712 | 1.298761 | https://www.genecards.org/cgi-bin/carddisp.pl?gene=SUV39H1 |
| LDLR | Low Density Lipoprotein Receptor | Protein Coding | 47 | GC19P011091 | 1.29704 | https://www.genecards.org/cgi-bin/carddisp.pl?gene=LDLR |
| HUWE1 | HECT, UBA And WWE Domain Containing E3 Ubiquitin Protein Ligase 1 | Protein Coding | 42 | GC0XM053532 | 1.29704 | https://www.genecards.org/cgi-bin/carddisp.pl?gene=HUWE1 |
| CSF1 | Colony Stimulating Factor 1 | Protein Coding | 40 | GC01P109911 | 1.29704 | https://www.genecards.org/cgi-bin/carddisp.pl?gene=CSF1 |
| CALR | Calreticulin | Protein Coding | 48 | GC19P012938 | 1.296596 | https://www.genecards.org/cgi-bin/carddisp.pl?gene=CALR |
| STMN1 | Stathmin 1 | Protein Coding | 41 | GC01M025884 | 1.296596 | https://www.genecards.org/cgi-bin/carddisp.pl?gene=STMN1 |
| MIR150 | MicroRNA 150 | RNA Gene | 21 | GC19M049500 | 1.296596 | https://www.genecards.org/cgi-bin/carddisp.pl?gene=MIR150 |
| SLC8A2 | Solute Carrier Family 8 Member A2 | Protein Coding | 39 | GC19M047428 | 1.296018 | https://www.genecards.org/cgi-bin/carddisp.pl?gene=SLC8A2 |
| CCL4 | C-C Motif Chemokine Ligand 4 | Protein Coding | 38 | GC17P036103 | 1.294513 | https://www.genecards.org/cgi-bin/carddisp.pl?gene=CCL4 |
| FOXP3 | Forkhead Box P3 | Protein Coding | 44 | GC0XM049250 | 1.293219 | https://www.genecards.org/cgi-bin/carddisp.pl?gene=FOXP3 |
| NRP2 | Neuropilin 2 | Protein Coding | 42 | GC02P205681 | 1.286361 | https://www.genecards.org/cgi-bin/carddisp.pl?gene=NRP2 |
| AREG | Amphiregulin | Protein Coding | 40 | GC04P074445 | 1.286111 | https://www.genecards.org/cgi-bin/carddisp.pl?gene=AREG |
| CYP24A1 | Cytochrome P450 Family 24 Subfamily A Member 1 | Protein Coding | 45 | GC20M054153 | 1.285994 | https://www.genecards.org/cgi-bin/carddisp.pl?gene=CYP24A1 |
| SCD | Stearoyl-CoA Desaturase | Protein Coding | 46 | GC10P100347 | 1.283825 | https://www.genecards.org/cgi-bin/carddisp.pl?gene=SCD |
| MIR195 | MicroRNA 195 | RNA Gene | 19 | GC17M007018 | 1.283739 | https://www.genecards.org/cgi-bin/carddisp.pl?gene=MIR195 |
| MIR135B | MicroRNA 135b | RNA Gene | 19 | GC01M205448 | 1.283286 | https://www.genecards.org/cgi-bin/carddisp.pl?gene=MIR135B |
| MMP12 | Matrix Metallopeptidase 12 | Protein Coding | 43 | GC11M102862 | 1.281066 | https://www.genecards.org/cgi-bin/carddisp.pl?gene=MMP12 |
| MIR494 | MicroRNA 494 | RNA Gene | 16 | GC14P108186 | 1.281066 | https://www.genecards.org/cgi-bin/carddisp.pl?gene=MIR494 |
| CD59 | CD59 Molecule (CD59 Blood Group) | Protein Coding | 43 | GC11M033704 | 1.278466 | https://www.genecards.org/cgi-bin/carddisp.pl?gene=CD59 |
| TMBIM6 | Transmembrane BAX Inhibitor Motif Containing 6 | Protein Coding | 35 | GC12P049707 | 1.277593 | https://www.genecards.org/cgi-bin/carddisp.pl?gene=TMBIM6 |
| STK24 | Serine/Threonine Kinase 24 | Protein Coding | 43 | GC13M098445 | 1.277105 | https://www.genecards.org/cgi-bin/carddisp.pl?gene=STK24 |
| PDIA2 | Protein Disulfide Isomerase Family A Member 2 | Protein Coding | 37 | GC16P007956 | 1.275464 | https://www.genecards.org/cgi-bin/carddisp.pl?gene=PDIA2 |
| CDH2 | Cadherin 2 | Protein Coding | 50 | GC18M027950 | 1.27522 | https://www.genecards.org/cgi-bin/carddisp.pl?gene=CDH2 |
| NUDT1 | Nudix Hydrolase 1 | Protein Coding | 41 | GC07P002242 | 1.274936 | https://www.genecards.org/cgi-bin/carddisp.pl?gene=NUDT1 |
| CDK5 | Cyclin Dependent Kinase 5 | Protein Coding | 50 | GC07M151053 | 1.27312 | https://www.genecards.org/cgi-bin/carddisp.pl?gene=CDK5 |
| WWOX | WW Domain Containing Oxidoreductase | Protein Coding | 45 | GC16P078099 | 1.271293 | https://www.genecards.org/cgi-bin/carddisp.pl?gene=WWOX |
| TNKS | Tankyrase | Protein Coding | 42 | GC08P009555 | 1.271005 | https://www.genecards.org/cgi-bin/carddisp.pl?gene=TNKS |
| EIF6 | Eukaryotic Translation Initiation Factor 6 | Protein Coding | 38 | GC20M035278 | 1.271005 | https://www.genecards.org/cgi-bin/carddisp.pl?gene=EIF6 |
| MAP3K1 | Mitogen-Activated Protein Kinase Kinase Kinase 1 | Protein Coding | 47 | GC05P056815 | 1.269023 | https://www.genecards.org/cgi-bin/carddisp.pl?gene=MAP3K1 |
| BTRC | Beta-Transducin Repeat Containing E3 Ubiquitin Protein Ligase | Protein Coding | 44 | GC10P101354 | 1.269023 | https://www.genecards.org/cgi-bin/carddisp.pl?gene=BTRC |
| TGIF1 | TGFB Induced Factor Homeobox 1 | Protein Coding | 44 | GC18P003411 | 1.269023 | https://www.genecards.org/cgi-bin/carddisp.pl?gene=TGIF1 |
| PTGES3 | Prostaglandin E Synthase 3 | Protein Coding | 42 | GC12M056667 | 1.267773 | https://www.genecards.org/cgi-bin/carddisp.pl?gene=PTGES3 |
| PRKCB | Protein Kinase C Beta | Protein Coding | 46 | GC16P024073 | 1.266906 | https://www.genecards.org/cgi-bin/carddisp.pl?gene=PRKCB |
| OPRK1 | Opioid Receptor Kappa 1 | Protein Coding | 43 | GC08M053227 | 1.266906 | https://www.genecards.org/cgi-bin/carddisp.pl?gene=OPRK1 |
| MAP2K2 | Mitogen-Activated Protein Kinase Kinase 2 | Protein Coding | 51 | GC19M004090 | 1.263286 | https://www.genecards.org/cgi-bin/carddisp.pl?gene=MAP2K2 |
| CA2 | Carbonic Anhydrase 2 | Protein Coding | 48 | GC08P085463 | 1.263286 | https://www.genecards.org/cgi-bin/carddisp.pl?gene=CA2 |
| CDKN2B-AS1 | CDKN2B Antisense RNA 1 | RNA Gene | 21 | GC09P021994 | 1.260657 | https://www.genecards.org/cgi-bin/carddisp.pl?gene=CDKN2B-AS1 |
| FASLG | Fas Ligand | Protein Coding | 44 | GC01P172628 | 1.259298 | https://www.genecards.org/cgi-bin/carddisp.pl?gene=FASLG |
| HNRNPA1 | Heterogeneous Nuclear Ribonucleoprotein A1 | Protein Coding | 44 | GC12P054280 | 1.259298 | https://www.genecards.org/cgi-bin/carddisp.pl?gene=HNRNPA1 |
| MCM7 | Minichromosome Maintenance Complex Component 7 | Protein Coding | 43 | GC07M100092 | 1.259298 | https://www.genecards.org/cgi-bin/carddisp.pl?gene=MCM7 |
| FSTL3 | Follistatin Like 3 | Protein Coding | 37 | GC19P000676 | 1.25914 | https://www.genecards.org/cgi-bin/carddisp.pl?gene=FSTL3 |
| NOTCH2 | Notch Receptor 2 | Protein Coding | 47 | GC01M119911 | 1.257615 | https://www.genecards.org/cgi-bin/carddisp.pl?gene=NOTCH2 |
| AFP | Alpha Fetoprotein | Protein Coding | 43 | GC04P073431 | 1.257451 | https://www.genecards.org/cgi-bin/carddisp.pl?gene=AFP |
| EZR | Ezrin | Protein Coding | 43 | GC06M158765 | 1.257451 | https://www.genecards.org/cgi-bin/carddisp.pl?gene=EZR |
| TSGA10 | Testis Specific 10 | Protein Coding | 34 | GC02M098997 | 1.256153 | https://www.genecards.org/cgi-bin/carddisp.pl?gene=TSGA10 |
| MIR107 | MicroRNA 107 | RNA Gene | 18 | GC10M089600 | 1.256153 | https://www.genecards.org/cgi-bin/carddisp.pl?gene=MIR107 |
| STK11 | Serine/Threonine Kinase 11 | Protein Coding | 47 | GC19P001177 | 1.253728 | https://www.genecards.org/cgi-bin/carddisp.pl?gene=STK11 |
| MAPT | Microtubule Associated Protein Tau | Protein Coding | 47 | GC17P045894 | 1.251092 | https://www.genecards.org/cgi-bin/carddisp.pl?gene=MAPT |
| TREM1 | Triggering Receptor Expressed On Myeloid Cells 1 | Protein Coding | 39 | GC06M041267 | 1.251092 | https://www.genecards.org/cgi-bin/carddisp.pl?gene=TREM1 |
| CHGA | Chromogranin A | Protein Coding | 41 | GC14P092929 | 1.249905 | https://www.genecards.org/cgi-bin/carddisp.pl?gene=CHGA |
| MAML3 | Mastermind Like Transcriptional Coactivator 3 | Protein Coding | 36 | GC04M139716 | 1.249696 | https://www.genecards.org/cgi-bin/carddisp.pl?gene=MAML3 |
| MIR133A1 | MicroRNA 133a-1 | RNA Gene | 17 | GC18M021917 | 1.249696 | https://www.genecards.org/cgi-bin/carddisp.pl?gene=MIR133A1 |
| FABP4 | Fatty Acid Binding Protein 4 | Protein Coding | 42 | GC08M081478 | 1.249264 | https://www.genecards.org/cgi-bin/carddisp.pl?gene=FABP4 |
| ADRB2 | Adrenoceptor Beta 2 | Protein Coding | 47 | GC05P148825 | 1.248366 | https://www.genecards.org/cgi-bin/carddisp.pl?gene=ADRB2 |
| PPP1R10 | Protein Phosphatase 1 Regulatory Subunit 10 | Protein Coding | 36 | GC06M030600 | 1.244979 | https://www.genecards.org/cgi-bin/carddisp.pl?gene=PPP1R10 |
| IRS1 | Insulin Receptor Substrate 1 | Protein Coding | 45 | GC02M226731 | 1.243948 | https://www.genecards.org/cgi-bin/carddisp.pl?gene=IRS1 |
| C3 | Complement C3 | Protein Coding | 45 | GC19M006677 | 1.243343 | https://www.genecards.org/cgi-bin/carddisp.pl?gene=C3 |
| PYY | Peptide YY | Protein Coding | 41 | GC17M043952 | 1.243343 | https://www.genecards.org/cgi-bin/carddisp.pl?gene=PYY |
| MAG | Myelin Associated Glycoprotein | Protein Coding | 43 | GC19P035292 | 1.24302 | https://www.genecards.org/cgi-bin/carddisp.pl?gene=MAG |
| PML | PML Nuclear Body Scaffold | Protein Coding | 44 | GC15P073994 | 1.239972 | https://www.genecards.org/cgi-bin/carddisp.pl?gene=PML |
| NT5C1A | 5'-Nucleotidase, Cytosolic IA | Protein Coding | 37 | GC01M039659 | 1.239056 | https://www.genecards.org/cgi-bin/carddisp.pl?gene=NT5C1A |
| MACIR | Macrophage Immunometabolism Regulator | Protein Coding | 23 | GC05P103259 | 1.239056 | https://www.genecards.org/cgi-bin/carddisp.pl?gene=MACIR |
| GLUD1 | Glutamate Dehydrogenase 1 | Protein Coding | 47 | GC10M087050 | 1.238922 | https://www.genecards.org/cgi-bin/carddisp.pl?gene=GLUD1 |
| LOXL1 | Lysyl Oxidase Like 1 | Protein Coding | 41 | GC15P073925 | 1.238922 | https://www.genecards.org/cgi-bin/carddisp.pl?gene=LOXL1 |
| DGKQ | Diacylglycerol Kinase Theta | Protein Coding | 40 | GC04M000958 | 1.238922 | https://www.genecards.org/cgi-bin/carddisp.pl?gene=DGKQ |
| MEG3 | Maternally Expressed 3 | RNA Gene | 29 | GC14P108151 | 1.238922 | https://www.genecards.org/cgi-bin/carddisp.pl?gene=MEG3 |
| BMI1 | BMI1 Proto-Oncogene, Polycomb Ring Finger | Protein Coding | 42 | GC10P022326 | 1.238889 | https://www.genecards.org/cgi-bin/carddisp.pl?gene=BMI1 |
| GRN | Granulin Precursor | Protein Coding | 45 | GC17P044345 | 1.238273 | https://www.genecards.org/cgi-bin/carddisp.pl?gene=GRN |
| CCNG2 | Cyclin G2 | Protein Coding | 35 | GC04P077158 | 1.238273 | https://www.genecards.org/cgi-bin/carddisp.pl?gene=CCNG2 |
| TGFBR3 | Transforming Growth Factor Beta Receptor 3 | Protein Coding | 45 | GC01M091680 | 1.237501 | https://www.genecards.org/cgi-bin/carddisp.pl?gene=TGFBR3 |
| P2RX3 | Purinergic Receptor P2X 3 | Protein Coding | 40 | GC11P057356 | 1.237501 | https://www.genecards.org/cgi-bin/carddisp.pl?gene=P2RX3 |
| UQCRFS1 | Ubiquinol-Cytochrome C Reductase, Rieske Iron-Sulfur Polypeptide 1 | Protein Coding | 44 | GC19M029205 | 1.233328 | https://www.genecards.org/cgi-bin/carddisp.pl?gene=UQCRFS1 |
| MYH9 | Myosin Heavy Chain 9 | Protein Coding | 47 | GC22M036281 | 1.229197 | https://www.genecards.org/cgi-bin/carddisp.pl?gene=MYH9 |
| IL4 | Interleukin 4 | Protein Coding | 43 | GC05P132673 | 1.229197 | https://www.genecards.org/cgi-bin/carddisp.pl?gene=IL4 |
| SLC17A5 | Solute Carrier Family 17 Member 5 | Protein Coding | 42 | GC06M073593 | 1.229197 | https://www.genecards.org/cgi-bin/carddisp.pl?gene=SLC17A5 |
| GAP43 | Growth Associated Protein 43 | Protein Coding | 41 | GC03P115623 | 1.229197 | https://www.genecards.org/cgi-bin/carddisp.pl?gene=GAP43 |
| EIF4E2 | Eukaryotic Translation Initiation Factor 4E Family Member 2 | Protein Coding | 38 | GC02P232550 | 1.229197 | https://www.genecards.org/cgi-bin/carddisp.pl?gene=EIF4E2 |
| IL1RAPL2 | Interleukin 1 Receptor Accessory Protein Like 2 | Protein Coding | 36 | GC0XP104566 | 1.229197 | https://www.genecards.org/cgi-bin/carddisp.pl?gene=IL1RAPL2 |
| CDK4 | Cyclin Dependent Kinase 4 | Protein Coding | 51 | GC12M057743 | 1.228137 | https://www.genecards.org/cgi-bin/carddisp.pl?gene=CDK4 |
| PPP1CB | Protein Phosphatase 1 Catalytic Subunit Beta | Protein Coding | 44 | GC02P028752 | 1.228137 | https://www.genecards.org/cgi-bin/carddisp.pl?gene=PPP1CB |
| TIE1 | Tyrosine Kinase With Immunoglobulin Like And EGF Like Domains 1 | Protein Coding | 42 | GC01P043300 | 1.22708 | https://www.genecards.org/cgi-bin/carddisp.pl?gene=TIE1 |
| DDX3X | DEAD-Box Helicase 3 X-Linked | Protein Coding | 45 | GC0XP041333 | 1.224818 | https://www.genecards.org/cgi-bin/carddisp.pl?gene=DDX3X |
| PTGS1 | Prostaglandin-Endoperoxide Synthase 1 | Protein Coding | 44 | GC09P122370 | 1.224818 | https://www.genecards.org/cgi-bin/carddisp.pl?gene=PTGS1 |
| TRIM28 | Tripartite Motif Containing 28 | Protein Coding | 41 | GC19P058544 | 1.224818 | https://www.genecards.org/cgi-bin/carddisp.pl?gene=TRIM28 |
| PLAGL2 | PLAG1 Like Zinc Finger 2 | Protein Coding | 35 | GC20M032192 | 1.224719 | https://www.genecards.org/cgi-bin/carddisp.pl?gene=PLAGL2 |
| ORAI1 | ORAI Calcium Release-Activated Calcium Modulator 1 | Protein Coding | 42 | GC12P124800 | 1.22313 | https://www.genecards.org/cgi-bin/carddisp.pl?gene=ORAI1 |
| ITGA2 | Integrin Subunit Alpha 2 | Protein Coding | 43 | GC05P052989 | 1.221997 | https://www.genecards.org/cgi-bin/carddisp.pl?gene=ITGA2 |
| CRYAB | Crystallin Alpha B | Protein Coding | 42 | GC11M111908 | 1.221997 | https://www.genecards.org/cgi-bin/carddisp.pl?gene=CRYAB |
| DYNLT1 | Dynein Light Chain Tctex-Type 1 | Protein Coding | 35 | GC06M158636 | 1.219562 | https://www.genecards.org/cgi-bin/carddisp.pl?gene=DYNLT1 |
| MIR455 | MicroRNA 455 | RNA Gene | 18 | GC09P114209 | 1.219562 | https://www.genecards.org/cgi-bin/carddisp.pl?gene=MIR455 |
| NPTN-IT1 | NPTN Intronic Transcript 1 | RNA Gene | 12 | GC15M073566 | 1.219562 | https://www.genecards.org/cgi-bin/carddisp.pl?gene=NPTN-IT1 |
| JAK3 | Janus Kinase 3 | Protein Coding | 50 | GC19M017824 | 1.219163 | https://www.genecards.org/cgi-bin/carddisp.pl?gene=JAK3 |
| AQP4 | Aquaporin 4 | Protein Coding | 44 | GC18M026852 | 1.219163 | https://www.genecards.org/cgi-bin/carddisp.pl?gene=AQP4 |
| HSPA1B | Heat Shock Protein Family A (Hsp70) Member 1B | Protein Coding | 37 | GC06P070159 | 1.216962 | https://www.genecards.org/cgi-bin/carddisp.pl?gene=HSPA1B |
| SELL | Selectin L | Protein Coding | 40 | GC01M169690 | 1.214887 | https://www.genecards.org/cgi-bin/carddisp.pl?gene=SELL |
| SLC16A3 | Solute Carrier Family 16 Member 3 | Protein Coding | 42 | GC17P082217 | 1.2147 | https://www.genecards.org/cgi-bin/carddisp.pl?gene=SLC16A3 |
| PLAGL1 | PLAG1 Like Zinc Finger 1 | Protein Coding | 42 | GC06M143940 | 1.210837 | https://www.genecards.org/cgi-bin/carddisp.pl?gene=PLAGL1 |
| CR1 | Complement C3b/C4b Receptor 1 (Knops Blood Group) | Protein Coding | 43 | GC01P207496 | 1.20881 | https://www.genecards.org/cgi-bin/carddisp.pl?gene=CR1 |
| MIR495 | MicroRNA 495 | RNA Gene | 16 | GC14P108187 | 1.20881 | https://www.genecards.org/cgi-bin/carddisp.pl?gene=MIR495 |
| DYNC1I2 | Dynein Cytoplasmic 1 Intermediate Chain 2 | Protein Coding | 38 | GC02P171687 | 1.208789 | https://www.genecards.org/cgi-bin/carddisp.pl?gene=DYNC1I2 |
| H4-16 | H4 Histone 16 | Protein Coding | 31 | GC12M018283 | 1.208789 | https://www.genecards.org/cgi-bin/carddisp.pl?gene=H4-16 |
| SPARC | Secreted Protein Acidic And Cysteine Rich | Protein Coding | 48 | GC05M151661 | 1.208315 | https://www.genecards.org/cgi-bin/carddisp.pl?gene=SPARC |
| TOP1 | DNA Topoisomerase I | Protein Coding | 46 | GC20P041028 | 1.208315 | https://www.genecards.org/cgi-bin/carddisp.pl?gene=TOP1 |
| HSPA14 | Heat Shock Protein Family A (Hsp70) Member 14 | Protein Coding | 38 | GC10P014847 | 1.206283 | https://www.genecards.org/cgi-bin/carddisp.pl?gene=HSPA14 |
| SPTLC1 | Serine Palmitoyltransferase Long Chain Base Subunit 1 | Protein Coding | 44 | GC09M092002 | 1.20619 | https://www.genecards.org/cgi-bin/carddisp.pl?gene=SPTLC1 |
| GREM1 | Gremlin 1, DAN Family BMP Antagonist | Protein Coding | 41 | GC15P035541 | 1.20619 | https://www.genecards.org/cgi-bin/carddisp.pl?gene=GREM1 |
| MIR138-2 | MicroRNA 138-2 | RNA Gene | 21 | GC16P056867 | 1.20619 | https://www.genecards.org/cgi-bin/carddisp.pl?gene=MIR138-2 |
| NRAS | NRAS Proto-Oncogene, GTPase | Protein Coding | 46 | GC01M114704 | 1.205312 | https://www.genecards.org/cgi-bin/carddisp.pl?gene=NRAS |
| DELEC1 | Deleted In Esophageal Cancer 1 | RNA Gene | 22 | GC09P117741 | 1.205312 | https://www.genecards.org/cgi-bin/carddisp.pl?gene=DELEC1 |
| ITPR2 | Inositol 1,4,5-Trisphosphate Receptor Type 2 | Protein Coding | 44 | GC12M026336 | 1.205137 | https://www.genecards.org/cgi-bin/carddisp.pl?gene=ITPR2 |
| HTR2C | 5-Hydroxytryptamine Receptor 2C | Protein Coding | 46 | GC0XP114584 | 1.204924 | https://www.genecards.org/cgi-bin/carddisp.pl?gene=HTR2C |
| ELF2 | E74 Like ETS Transcription Factor 2 | Protein Coding | 38 | GC04M139028 | 1.200517 | https://www.genecards.org/cgi-bin/carddisp.pl?gene=ELF2 |
| CUL3 | Cullin 3 | Protein Coding | 45 | GC02M224470 | 1.200247 | https://www.genecards.org/cgi-bin/carddisp.pl?gene=CUL3 |
| ROS1 | ROS Proto-Oncogene 1, Receptor Tyrosine Kinase | Protein Coding | 44 | GC06M117287 | 1.199001 | https://www.genecards.org/cgi-bin/carddisp.pl?gene=ROS1 |
| TAGLN2 | Transgelin 2 | Protein Coding | 39 | GC01M159918 | 1.197636 | https://www.genecards.org/cgi-bin/carddisp.pl?gene=TAGLN2 |
| MIR200A | MicroRNA 200a | RNA Gene | 21 | GC01P002924 | 1.197636 | https://www.genecards.org/cgi-bin/carddisp.pl?gene=MIR200A |
| MIR98 | MicroRNA 98 | RNA Gene | 17 | GC0XM053614 | 1.197636 | https://www.genecards.org/cgi-bin/carddisp.pl?gene=MIR98 |
| BIRC2 | Baculoviral IAP Repeat Containing 2 | Protein Coding | 43 | GC11P102347 | 1.196394 | https://www.genecards.org/cgi-bin/carddisp.pl?gene=BIRC2 |
| PPP2CA | Protein Phosphatase 2 Catalytic Subunit Alpha | Protein Coding | 47 | GC05M134194 | 1.193118 | https://www.genecards.org/cgi-bin/carddisp.pl?gene=PPP2CA |
| UCP1 | Uncoupling Protein 1 | Protein Coding | 42 | GC04M140559 | 1.193118 | https://www.genecards.org/cgi-bin/carddisp.pl?gene=UCP1 |
| LIF | LIF Interleukin 6 Family Cytokine | Protein Coding | 39 | GC22M030240 | 1.190804 | https://www.genecards.org/cgi-bin/carddisp.pl?gene=LIF |
| BMP1 | Bone Morphogenetic Protein 1 | Protein Coding | 44 | GC08P022164 | 1.189592 | https://www.genecards.org/cgi-bin/carddisp.pl?gene=BMP1 |
| RPE65 | Retinoid Isomerohydrolase RPE65 | Protein Coding | 42 | GC01M068428 | 1.189592 | https://www.genecards.org/cgi-bin/carddisp.pl?gene=RPE65 |
| ALPP | Alkaline Phosphatase, Placental | Protein Coding | 43 | GC02P232378 | 1.188612 | https://www.genecards.org/cgi-bin/carddisp.pl?gene=ALPP |
| FGF9 | Fibroblast Growth Factor 9 | Protein Coding | 41 | GC13P021671 | 1.188612 | https://www.genecards.org/cgi-bin/carddisp.pl?gene=FGF9 |
| GNAS | GNAS Complex Locus | Protein Coding | 48 | GC20P058839 | 1.186862 | https://www.genecards.org/cgi-bin/carddisp.pl?gene=GNAS |
| TGFBR2 | Transforming Growth Factor Beta Receptor 2 | Protein Coding | 48 | GC03P030623 | 1.186487 | https://www.genecards.org/cgi-bin/carddisp.pl?gene=TGFBR2 |
| CACYBP | Calcyclin Binding Protein | Protein Coding | 37 | GC01P174968 | 1.186431 | https://www.genecards.org/cgi-bin/carddisp.pl?gene=CACYBP |
| CHRM1 | Cholinergic Receptor Muscarinic 1 | Protein Coding | 44 | GC11M079900 | 1.185985 | https://www.genecards.org/cgi-bin/carddisp.pl?gene=CHRM1 |
| SLCO1B3 | Solute Carrier Organic Anion Transporter Family Member 1B3 | Protein Coding | 42 | GC12P020810 | 1.185985 | https://www.genecards.org/cgi-bin/carddisp.pl?gene=SLCO1B3 |
| PDHA1 | Pyruvate Dehydrogenase E1 Subunit Alpha 1 | Protein Coding | 47 | GC0XP019343 | 1.184543 | https://www.genecards.org/cgi-bin/carddisp.pl?gene=PDHA1 |
| ILF3 | Interleukin Enhancer Binding Factor 3 | Protein Coding | 37 | GC19P010655 | 1.180858 | https://www.genecards.org/cgi-bin/carddisp.pl?gene=ILF3 |
| DRD3 | Dopamine Receptor D3 | Protein Coding | 43 | GC03M114128 | 1.179428 | https://www.genecards.org/cgi-bin/carddisp.pl?gene=DRD3 |
| GCM1 | Glial Cells Missing Transcription Factor 1 | Protein Coding | 36 | GC06M056974 | 1.179428 | https://www.genecards.org/cgi-bin/carddisp.pl?gene=GCM1 |
| STIM1 | Stromal Interaction Molecule 1 | Protein Coding | 46 | GC11P003855 | 1.176056 | https://www.genecards.org/cgi-bin/carddisp.pl?gene=STIM1 |
| GADD45A | Growth Arrest And DNA Damage Inducible Alpha | Protein Coding | 42 | GC01P067685 | 1.176056 | https://www.genecards.org/cgi-bin/carddisp.pl?gene=GADD45A |
| VTN | Vitronectin | Protein Coding | 41 | GC17M032763 | 1.174082 | https://www.genecards.org/cgi-bin/carddisp.pl?gene=VTN |
| S100A11 | S100 Calcium Binding Protein A11 | Protein Coding | 40 | GC01M152032 | 1.173792 | https://www.genecards.org/cgi-bin/carddisp.pl?gene=S100A11 |
| ERCC6 | ERCC Excision Repair 6, Chromatin Remodeling Factor | Protein Coding | 43 | GC10M049454 | 1.173589 | https://www.genecards.org/cgi-bin/carddisp.pl?gene=ERCC6 |
| WDR5 | WD Repeat Domain 5 | Protein Coding | 42 | GC09P134135 | 1.169985 | https://www.genecards.org/cgi-bin/carddisp.pl?gene=WDR5 |
| REG1A | Regenerating Family Member 1 Alpha | Protein Coding | 39 | GC02P079120 | 1.16532 | https://www.genecards.org/cgi-bin/carddisp.pl?gene=REG1A |
| TKTL1 | Transketolase Like 1 | Protein Coding | 39 | GC0XP154295 | 1.165304 | https://www.genecards.org/cgi-bin/carddisp.pl?gene=TKTL1 |
| MIR451A | MicroRNA 451a | RNA Gene | 18 | GC17M028861 | 1.165304 | https://www.genecards.org/cgi-bin/carddisp.pl?gene=MIR451A |
| GAPLINC | Gastric Adenocarcinoma Associated, Positive CD44 Regulator, Long Intergenic Non-Coding RNA | RNA Gene | 13 | GC18P003467 | 1.163363 | https://www.genecards.org/cgi-bin/carddisp.pl?gene=GAPLINC |
| DKK1 | Dickkopf WNT Signaling Pathway Inhibitor 1 | Protein Coding | 42 | GC10P052314 | 1.163018 | https://www.genecards.org/cgi-bin/carddisp.pl?gene=DKK1 |
| BOK | BCL2 Family Apoptosis Regulator BOK | Protein Coding | 38 | GC02P241558 | 1.163018 | https://www.genecards.org/cgi-bin/carddisp.pl?gene=BOK |
| RHOT2 | Ras Homolog Family Member T2 | Protein Coding | 37 | GC16P007971 | 1.163018 | https://www.genecards.org/cgi-bin/carddisp.pl?gene=RHOT2 |
| TUBB | Tubulin Beta Class I | Protein Coding | 47 | GC06P070123 | 1.16119 | https://www.genecards.org/cgi-bin/carddisp.pl?gene=TUBB |
| MAPK9 | Mitogen-Activated Protein Kinase 9 | Protein Coding | 46 | GC05M180242 | 1.160972 | https://www.genecards.org/cgi-bin/carddisp.pl?gene=MAPK9 |
| UBE2N | Ubiquitin Conjugating Enzyme E2 N | Protein Coding | 44 | GC12M093406 | 1.160972 | https://www.genecards.org/cgi-bin/carddisp.pl?gene=UBE2N |
| SPON1 | Spondin 1 | Protein Coding | 36 | GC11P013940 | 1.160972 | https://www.genecards.org/cgi-bin/carddisp.pl?gene=SPON1 |
| MIR215 | MicroRNA 215 | RNA Gene | 18 | GC01M220117 | 1.160972 | https://www.genecards.org/cgi-bin/carddisp.pl?gene=MIR215 |
| MIR301B | MicroRNA 301b | RNA Gene | 16 | GC22P030548 | 1.160972 | https://www.genecards.org/cgi-bin/carddisp.pl?gene=MIR301B |
| DDR2 | Discoidin Domain Receptor Tyrosine Kinase 2 | Protein Coding | 48 | GC01P162631 | 1.16026 | https://www.genecards.org/cgi-bin/carddisp.pl?gene=DDR2 |
| PPP5C | Protein Phosphatase 5 Catalytic Subunit | Protein Coding | 43 | GC19P046346 | 1.16026 | https://www.genecards.org/cgi-bin/carddisp.pl?gene=PPP5C |
| NEDD9 | Neural Precursor Cell Expressed, Developmentally Down-Regulated 9 | Protein Coding | 42 | GC06M011183 | 1.16026 | https://www.genecards.org/cgi-bin/carddisp.pl?gene=NEDD9 |
| CXCL9 | C-X-C Motif Chemokine Ligand 9 | Protein Coding | 37 | GC04M076001 | 1.158048 | https://www.genecards.org/cgi-bin/carddisp.pl?gene=CXCL9 |
| SLC38A2 | Solute Carrier Family 38 Member 2 | Protein Coding | 40 | GC12M046358 | 1.156463 | https://www.genecards.org/cgi-bin/carddisp.pl?gene=SLC38A2 |
| RET | Ret Proto-Oncogene | Protein Coding | 51 | GC10P043081 | 1.15619 | https://www.genecards.org/cgi-bin/carddisp.pl?gene=RET |
| NR2F2 | Nuclear Receptor Subfamily 2 Group F Member 2 | Protein Coding | 48 | GC15P096325 | 1.15413 | https://www.genecards.org/cgi-bin/carddisp.pl?gene=NR2F2 |
| CHAT | Choline O-Acetyltransferase | Protein Coding | 45 | GC10P049609 | 1.15413 | https://www.genecards.org/cgi-bin/carddisp.pl?gene=CHAT |
| HMGA1 | High Mobility Group AT-Hook 1 | Protein Coding | 44 | GC06P070252 | 1.15413 | https://www.genecards.org/cgi-bin/carddisp.pl?gene=HMGA1 |
| HBG1 | Hemoglobin Subunit Gamma 1 | Protein Coding | 41 | GC11M006019 | 1.15413 | https://www.genecards.org/cgi-bin/carddisp.pl?gene=HBG1 |
| MIR93 | MicroRNA 93 | RNA Gene | 21 | GC07M101075 | 1.15413 | https://www.genecards.org/cgi-bin/carddisp.pl?gene=MIR93 |
| EPHA3 | EPH Receptor A3 | Protein Coding | 44 | GC03P089077 | 1.152313 | https://www.genecards.org/cgi-bin/carddisp.pl?gene=EPHA3 |
| JUNB | JunB Proto-Oncogene, AP-1 Transcription Factor Subunit | Protein Coding | 40 | GC19P012791 | 1.150226 | https://www.genecards.org/cgi-bin/carddisp.pl?gene=JUNB |
| ELK3 | ETS Transcription Factor ELK3 | Protein Coding | 38 | GC12P096194 | 1.150226 | https://www.genecards.org/cgi-bin/carddisp.pl?gene=ELK3 |
| CASP7 | Caspase 7 | Protein Coding | 47 | GC10P113679 | 1.150198 | https://www.genecards.org/cgi-bin/carddisp.pl?gene=CASP7 |
| PTK6 | Protein Tyrosine Kinase 6 | Protein Coding | 46 | GC20M063528 | 1.150198 | https://www.genecards.org/cgi-bin/carddisp.pl?gene=PTK6 |
| CCT2 | Chaperonin Containing TCP1 Subunit 2 | Protein Coding | 39 | GC12P069585 | 1.149045 | https://www.genecards.org/cgi-bin/carddisp.pl?gene=CCT2 |
| COL4A2 | Collagen Type IV Alpha 2 Chain | Protein Coding | 43 | GC13P110305 | 1.147882 | https://www.genecards.org/cgi-bin/carddisp.pl?gene=COL4A2 |
| SMO | Smoothened, Frizzled Class Receptor | Protein Coding | 47 | GC07P130977 | 1.147517 | https://www.genecards.org/cgi-bin/carddisp.pl?gene=SMO |
| PDE4A | Phosphodiesterase 4A | Protein Coding | 43 | GC19P010416 | 1.147517 | https://www.genecards.org/cgi-bin/carddisp.pl?gene=PDE4A |
| BGLAP | Bone Gamma-Carboxyglutamate Protein | Protein Coding | 38 | GC01P156242 | 1.147517 | https://www.genecards.org/cgi-bin/carddisp.pl?gene=BGLAP |
| SEMA4D | Semaphorin 4D | Protein Coding | 43 | GC09M089360 | 1.147418 | https://www.genecards.org/cgi-bin/carddisp.pl?gene=SEMA4D |
| TET2 | Tet Methylcytosine Dioxygenase 2 | Protein Coding | 43 | GC04P105145 | 1.146156 | https://www.genecards.org/cgi-bin/carddisp.pl?gene=TET2 |
| CD40LG | CD40 Ligand | Protein Coding | 44 | GC0XP136649 | 1.142479 | https://www.genecards.org/cgi-bin/carddisp.pl?gene=CD40LG |
| MAT2A | Methionine Adenosyltransferase 2A | Protein Coding | 44 | GC02P085667 | 1.142479 | https://www.genecards.org/cgi-bin/carddisp.pl?gene=MAT2A |
| SLC19A3 | Solute Carrier Family 19 Member 3 | Protein Coding | 44 | GC02M227685 | 1.142479 | https://www.genecards.org/cgi-bin/carddisp.pl?gene=SLC19A3 |
| SPRY2 | Sprouty RTK Signaling Antagonist 2 | Protein Coding | 44 | GC13M080335 | 1.142479 | https://www.genecards.org/cgi-bin/carddisp.pl?gene=SPRY2 |
| ERCC1 | ERCC Excision Repair 1, Endonuclease Non-Catalytic Subunit | Protein Coding | 43 | GC19M057120 | 1.142479 | https://www.genecards.org/cgi-bin/carddisp.pl?gene=ERCC1 |
| MIR15A | MicroRNA 15a | RNA Gene | 15 | GC13M050049 | 1.142479 | https://www.genecards.org/cgi-bin/carddisp.pl?gene=MIR15A |
| C19orf33 | Chromosome 19 Open Reading Frame 33 | Protein Coding | 28 | GC19P038304 | 1.141717 | https://www.genecards.org/cgi-bin/carddisp.pl?gene=C19orf33 |
| ERCC2 | ERCC Excision Repair 2, TFIIH Core Complex Helicase Subunit | Protein Coding | 46 | GC19M045349 | 1.140211 | https://www.genecards.org/cgi-bin/carddisp.pl?gene=ERCC2 |
| SUV39H2 | SUV39H2 Histone Lysine Methyltransferase | Protein Coding | 41 | GC10P014878 | 1.140211 | https://www.genecards.org/cgi-bin/carddisp.pl?gene=SUV39H2 |
| FLT3 | Fms Related Receptor Tyrosine Kinase 3 | Protein Coding | 50 | GC13M028003 | 1.139851 | https://www.genecards.org/cgi-bin/carddisp.pl?gene=FLT3 |
| SPINT2 | Serine Peptidase Inhibitor, Kunitz Type 2 | Protein Coding | 41 | GC19P038244 | 1.139851 | https://www.genecards.org/cgi-bin/carddisp.pl?gene=SPINT2 |
| TRIM22 | Tripartite Motif Containing 22 | Protein Coding | 38 | GC11P005689 | 1.139851 | https://www.genecards.org/cgi-bin/carddisp.pl?gene=TRIM22 |
| MIR31 | MicroRNA 31 | RNA Gene | 19 | GC09M021635 | 1.139158 | https://www.genecards.org/cgi-bin/carddisp.pl?gene=MIR31 |
| ROR2 | Receptor Tyrosine Kinase Like Orphan Receptor 2 | Protein Coding | 46 | GC09M091564 | 1.138106 | https://www.genecards.org/cgi-bin/carddisp.pl?gene=ROR2 |
| PDGFA | Platelet Derived Growth Factor Subunit A | Protein Coding | 41 | GC07M000497 | 1.136809 | https://www.genecards.org/cgi-bin/carddisp.pl?gene=PDGFA |
| PRDX3 | Peroxiredoxin 3 | Protein Coding | 41 | GC10M119167 | 1.136809 | https://www.genecards.org/cgi-bin/carddisp.pl?gene=PRDX3 |
| KPNB1 | Karyopherin Subunit Beta 1 | Protein Coding | 41 | GC17P047649 | 1.136644 | https://www.genecards.org/cgi-bin/carddisp.pl?gene=KPNB1 |
| ZEBTR | ZEB1 Transcriptional Regulator RNA | RNA Gene | 3 | GC10U902865 | 1.136644 | https://www.genecards.org/cgi-bin/carddisp.pl?gene=ZEBTR |
| DNMT1 | DNA Methyltransferase 1 | Protein Coding | 48 | GC19M010133 | 1.135781 | https://www.genecards.org/cgi-bin/carddisp.pl?gene=DNMT1 |
| USP13 | Ubiquitin Specific Peptidase 13 | Protein Coding | 43 | GC03P179652 | 1.133513 | https://www.genecards.org/cgi-bin/carddisp.pl?gene=USP13 |
| PPP2R1A | Protein Phosphatase 2 Scaffold Subunit Aalpha | Protein Coding | 45 | GC19P052189 | 1.130286 | https://www.genecards.org/cgi-bin/carddisp.pl?gene=PPP2R1A |
| DFFA | DNA Fragmentation Factor Subunit Alpha | Protein Coding | 42 | GC01M010456 | 1.130286 | https://www.genecards.org/cgi-bin/carddisp.pl?gene=DFFA |
| MCM6 | Minichromosome Maintenance Complex Component 6 | Protein Coding | 42 | GC02M135839 | 1.130286 | https://www.genecards.org/cgi-bin/carddisp.pl?gene=MCM6 |
| POLR1C | RNA Polymerase I And III Subunit C | Protein Coding | 41 | GC06P070355 | 1.130286 | https://www.genecards.org/cgi-bin/carddisp.pl?gene=POLR1C |
| DNASE1 | Deoxyribonuclease 1 | Protein Coding | 40 | GC16P003611 | 1.130286 | https://www.genecards.org/cgi-bin/carddisp.pl?gene=DNASE1 |
| CXCL6 | C-X-C Motif Chemokine Ligand 6 | Protein Coding | 37 | GC04P073837 | 1.130286 | https://www.genecards.org/cgi-bin/carddisp.pl?gene=CXCL6 |
| IL20 | Interleukin 20 | Protein Coding | 36 | GC01P206866 | 1.130286 | https://www.genecards.org/cgi-bin/carddisp.pl?gene=IL20 |
| MELTF | Melanotransferrin | Protein Coding | 32 | GC03M196987 | 1.130286 | https://www.genecards.org/cgi-bin/carddisp.pl?gene=MELTF |
| ATP1A1 | ATPase Na+/K+ Transporting Subunit Alpha 1 | Protein Coding | 48 | GC01P116372 | 1.130212 | https://www.genecards.org/cgi-bin/carddisp.pl?gene=ATP1A1 |
| CD40 | CD40 Molecule | Protein Coding | 45 | GC20P046118 | 1.129099 | https://www.genecards.org/cgi-bin/carddisp.pl?gene=CD40 |
| MMP17 | Matrix Metallopeptidase 17 | Protein Coding | 42 | GC12P131828 | 1.129099 | https://www.genecards.org/cgi-bin/carddisp.pl?gene=MMP17 |
| ADM2 | Adrenomedullin 2 | Protein Coding | 32 | GC22P050481 | 1.129099 | https://www.genecards.org/cgi-bin/carddisp.pl?gene=ADM2 |
| CCN3 | Cellular Communication Network Factor 3 | Protein Coding | 31 | GC08P119416 | 1.129099 | https://www.genecards.org/cgi-bin/carddisp.pl?gene=CCN3 |
| MAP2K4 | Mitogen-Activated Protein Kinase Kinase 4 | Protein Coding | 44 | GC17P012020 | 1.12889 | https://www.genecards.org/cgi-bin/carddisp.pl?gene=MAP2K4 |
| ABCA3 | ATP Binding Cassette Subfamily A Member 3 | Protein Coding | 47 | GC16M002275 | 1.127702 | https://www.genecards.org/cgi-bin/carddisp.pl?gene=ABCA3 |
| EPHB4 | EPH Receptor B4 | Protein Coding | 49 | GC07M101134 | 1.122213 | https://www.genecards.org/cgi-bin/carddisp.pl?gene=EPHB4 |
| CDC6 | Cell Division Cycle 6 | Protein Coding | 43 | GC17P040287 | 1.122213 | https://www.genecards.org/cgi-bin/carddisp.pl?gene=CDC6 |
| AKAP12 | A-Kinase Anchoring Protein 12 | Protein Coding | 39 | GC06P151239 | 1.122213 | https://www.genecards.org/cgi-bin/carddisp.pl?gene=AKAP12 |
| OGT | O-Linked N-Acetylglucosamine (GlcNAc) Transferase | Protein Coding | 42 | GC0XP071534 | 1.119842 | https://www.genecards.org/cgi-bin/carddisp.pl?gene=OGT |
| CEBPD | CCAAT Enhancer Binding Protein Delta | Protein Coding | 36 | GC08M047759 | 1.119842 | https://www.genecards.org/cgi-bin/carddisp.pl?gene=CEBPD |
| SLC7A1 | Solute Carrier Family 7 Member 1 | Protein Coding | 42 | GC13M029509 | 1.118026 | https://www.genecards.org/cgi-bin/carddisp.pl?gene=SLC7A1 |
| PTAFR | Platelet Activating Factor Receptor | Protein Coding | 41 | GC01M028147 | 1.117782 | https://www.genecards.org/cgi-bin/carddisp.pl?gene=PTAFR |
| TET3 | Tet Methylcytosine Dioxygenase 3 | Protein Coding | 39 | GC02P073986 | 1.117466 | https://www.genecards.org/cgi-bin/carddisp.pl?gene=TET3 |
| PHB1 | Prohibitin 1 | Protein Coding | 35 | GC17M049406 | 1.117466 | https://www.genecards.org/cgi-bin/carddisp.pl?gene=PHB1 |
| PVT1 | Pvt1 Oncogene | RNA Gene | 24 | GC08P127841 | 1.117466 | https://www.genecards.org/cgi-bin/carddisp.pl?gene=PVT1 |
| SLC4A1 | Solute Carrier Family 4 Member 1 (Diego Blood Group) | Protein Coding | 45 | GC17M044608 | 1.112226 | https://www.genecards.org/cgi-bin/carddisp.pl?gene=SLC4A1 |
| GPX3 | Glutathione Peroxidase 3 | Protein Coding | 41 | GC05P150997 | 1.111585 | https://www.genecards.org/cgi-bin/carddisp.pl?gene=GPX3 |
| DDR1 | Discoidin Domain Receptor Tyrosine Kinase 1 | Protein Coding | 44 | GC06P070126 | 1.109131 | https://www.genecards.org/cgi-bin/carddisp.pl?gene=DDR1 |
| KCNJ8 | Potassium Inwardly Rectifying Channel Subfamily J Member 8 | Protein Coding | 42 | GC12M021764 | 1.109131 | https://www.genecards.org/cgi-bin/carddisp.pl?gene=KCNJ8 |
| PHOX2B | Paired Like Homeobox 2B | Protein Coding | 42 | GC04M041746 | 1.108453 | https://www.genecards.org/cgi-bin/carddisp.pl?gene=PHOX2B |
| MST1 | Macrophage Stimulating 1 | Protein Coding | 43 | GC03M049683 | 1.107489 | https://www.genecards.org/cgi-bin/carddisp.pl?gene=MST1 |
| SCAP | SREBF Chaperone | Protein Coding | 39 | GC03M047413 | 1.107489 | https://www.genecards.org/cgi-bin/carddisp.pl?gene=SCAP |
| POLR2A | RNA Polymerase II Subunit A | Protein Coding | 43 | GC17P010068 | 1.106709 | https://www.genecards.org/cgi-bin/carddisp.pl?gene=POLR2A |
| CPT1A | Carnitine Palmitoyltransferase 1A | Protein Coding | 47 | GC11M068754 | 1.106274 | https://www.genecards.org/cgi-bin/carddisp.pl?gene=CPT1A |
| ENO3 | Enolase 3 | Protein Coding | 46 | GC17P004948 | 1.106274 | https://www.genecards.org/cgi-bin/carddisp.pl?gene=ENO3 |
| BCL9 | BCL9 Transcription Coactivator | Protein Coding | 40 | GC01P147541 | 1.106274 | https://www.genecards.org/cgi-bin/carddisp.pl?gene=BCL9 |
| CXCR6 | C-X-C Motif Chemokine Receptor 6 | Protein Coding | 38 | GC03P046040 | 1.106274 | https://www.genecards.org/cgi-bin/carddisp.pl?gene=CXCR6 |
| ITGA6 | Integrin Subunit Alpha 6 | Protein Coding | 47 | GC02P172427 | 1.103912 | https://www.genecards.org/cgi-bin/carddisp.pl?gene=ITGA6 |
| LPIN1 | Lipin 1 | Protein Coding | 45 | GC02P011677 | 1.103912 | https://www.genecards.org/cgi-bin/carddisp.pl?gene=LPIN1 |
| FOXP1 | Forkhead Box P1 | Protein Coding | 44 | GC03M070926 | 1.103912 | https://www.genecards.org/cgi-bin/carddisp.pl?gene=FOXP1 |
| GAS6 | Growth Arrest Specific 6 | Protein Coding | 42 | GC13M113820 | 1.103912 | https://www.genecards.org/cgi-bin/carddisp.pl?gene=GAS6 |
| SERPINA3 | Serpin Family A Member 3 | Protein Coding | 41 | GC14P094612 | 1.103912 | https://www.genecards.org/cgi-bin/carddisp.pl?gene=SERPINA3 |
| UCHL1 | Ubiquitin C-Terminal Hydrolase L1 | Protein Coding | 48 | GC04P041256 | 1.102204 | https://www.genecards.org/cgi-bin/carddisp.pl?gene=UCHL1 |
| VCP | Valosin Containing Protein | Protein Coding | 47 | GC09M035056 | 1.102204 | https://www.genecards.org/cgi-bin/carddisp.pl?gene=VCP |
| TCP1 | T-Complex 1 | Protein Coding | 39 | GC06M159778 | 1.102204 | https://www.genecards.org/cgi-bin/carddisp.pl?gene=TCP1 |
| RPL8 | Ribosomal Protein L8 | Protein Coding | 39 | GC08M145122 | 1.099511 | https://www.genecards.org/cgi-bin/carddisp.pl?gene=RPL8 |
| PDCD1 | Programmed Cell Death 1 | Protein Coding | 45 | GC02M241849 | 1.098771 | https://www.genecards.org/cgi-bin/carddisp.pl?gene=PDCD1 |
| ITGA4 | Integrin Subunit Alpha 4 | Protein Coding | 46 | GC02P181456 | 1.098756 | https://www.genecards.org/cgi-bin/carddisp.pl?gene=ITGA4 |
| MIR141 | MicroRNA 141 | RNA Gene | 21 | GC12P016442 | 1.098756 | https://www.genecards.org/cgi-bin/carddisp.pl?gene=MIR141 |
| BDKRB2 | Bradykinin Receptor B2 | Protein Coding | 42 | GC14P096205 | 1.094081 | https://www.genecards.org/cgi-bin/carddisp.pl?gene=BDKRB2 |
| PURA | Purine Rich Element Binding Protein A | Protein Coding | 42 | GC05P140076 | 1.094081 | https://www.genecards.org/cgi-bin/carddisp.pl?gene=PURA |
| RGS4 | Regulator Of G Protein Signaling 4 | Protein Coding | 41 | GC01P163038 | 1.094081 | https://www.genecards.org/cgi-bin/carddisp.pl?gene=RGS4 |
| POLI | DNA Polymerase Iota | Protein Coding | 40 | GC18P054274 | 1.094081 | https://www.genecards.org/cgi-bin/carddisp.pl?gene=POLI |
| RHOT1 | Ras Homolog Family Member T1 | Protein Coding | 40 | GC17P032142 | 1.094081 | https://www.genecards.org/cgi-bin/carddisp.pl?gene=RHOT1 |
| KDM4C | Lysine Demethylase 4C | Protein Coding | 39 | GC09P006720 | 1.094081 | https://www.genecards.org/cgi-bin/carddisp.pl?gene=KDM4C |
| TRAK1 | Trafficking Kinesin Protein 1 | Protein Coding | 39 | GC03P042016 | 1.094081 | https://www.genecards.org/cgi-bin/carddisp.pl?gene=TRAK1 |
| GAST | Gastrin | Protein Coding | 38 | GC17P041712 | 1.094081 | https://www.genecards.org/cgi-bin/carddisp.pl?gene=GAST |
| IL1RN | Interleukin 1 Receptor Antagonist | Protein Coding | 46 | GC02P119261 | 1.091825 | https://www.genecards.org/cgi-bin/carddisp.pl?gene=IL1RN |
| DNAJA1 | DnaJ Heat Shock Protein Family (Hsp40) Member A1 | Protein Coding | 39 | GC09P033025 | 1.091825 | https://www.genecards.org/cgi-bin/carddisp.pl?gene=DNAJA1 |
| SENP3 | SUMO Specific Peptidase 3 | Protein Coding | 38 | GC17P010073 | 1.091825 | https://www.genecards.org/cgi-bin/carddisp.pl?gene=SENP3 |
| DAPK1 | Death Associated Protein Kinase 1 | Protein Coding | 47 | GC09P087497 | 1.089849 | https://www.genecards.org/cgi-bin/carddisp.pl?gene=DAPK1 |
| MAX | MYC Associated Factor X | Protein Coding | 44 | GC14M065039 | 1.089849 | https://www.genecards.org/cgi-bin/carddisp.pl?gene=MAX |
| IGFBP5 | Insulin Like Growth Factor Binding Protein 5 | Protein Coding | 40 | GC02M216672 | 1.089587 | https://www.genecards.org/cgi-bin/carddisp.pl?gene=IGFBP5 |
| HSPG2 | Heparan Sulfate Proteoglycan 2 | Protein Coding | 44 | GC01M021822 | 1.089481 | https://www.genecards.org/cgi-bin/carddisp.pl?gene=HSPG2 |
| TCF7L2 | Transcription Factor 7 Like 2 | Protein Coding | 44 | GC10P112950 | 1.089481 | https://www.genecards.org/cgi-bin/carddisp.pl?gene=TCF7L2 |
| MAPK8IP1 | Mitogen-Activated Protein Kinase 8 Interacting Protein 1 | Protein Coding | 43 | GC11P046286 | 1.088004 | https://www.genecards.org/cgi-bin/carddisp.pl?gene=MAPK8IP1 |
| C5AR1 | Complement C5a Receptor 1 | Protein Coding | 42 | GC19P047290 | 1.088004 | https://www.genecards.org/cgi-bin/carddisp.pl?gene=C5AR1 |
| CFL1 | Cofilin 1 | Protein Coding | 43 | GC11M065823 | 1.087541 | https://www.genecards.org/cgi-bin/carddisp.pl?gene=CFL1 |
| P2RX7 | Purinergic Receptor P2X 7 | Protein Coding | 43 | GC12P124795 | 1.082262 | https://www.genecards.org/cgi-bin/carddisp.pl?gene=P2RX7 |
| MYL2 | Myosin Light Chain 2 | Protein Coding | 46 | GC12M110910 | 1.08215 | https://www.genecards.org/cgi-bin/carddisp.pl?gene=MYL2 |
| LAMP3 | Lysosomal Associated Membrane Protein 3 | Protein Coding | 36 | GC03M183122 | 1.08182 | https://www.genecards.org/cgi-bin/carddisp.pl?gene=LAMP3 |
| MIR200C | MicroRNA 200c | RNA Gene | 21 | GC12P016441 | 1.081451 | https://www.genecards.org/cgi-bin/carddisp.pl?gene=MIR200C |
| EIF2S3 | Eukaryotic Translation Initiation Factor 2 Subunit Gamma | Protein Coding | 43 | GC0XP024054 | 1.081339 | https://www.genecards.org/cgi-bin/carddisp.pl?gene=EIF2S3 |
| RHEB | Ras Homolog, MTORC1 Binding | Protein Coding | 46 | GC07M151466 | 1.081261 | https://www.genecards.org/cgi-bin/carddisp.pl?gene=RHEB |
| FTL | Ferritin Light Chain | Protein Coding | 45 | GC19P048965 | 1.081261 | https://www.genecards.org/cgi-bin/carddisp.pl?gene=FTL |
| NRN1 | Neuritin 1 | Protein Coding | 37 | GC06M005997 | 1.081261 | https://www.genecards.org/cgi-bin/carddisp.pl?gene=NRN1 |
| ZNF217 | Zinc Finger Protein 217 | Protein Coding | 37 | GC20M053567 | 1.081261 | https://www.genecards.org/cgi-bin/carddisp.pl?gene=ZNF217 |
| UNC5B | Unc-5 Netrin Receptor B | Protein Coding | 36 | GC10P071212 | 1.081261 | https://www.genecards.org/cgi-bin/carddisp.pl?gene=UNC5B |
| GTF3A | General Transcription Factor IIIA | Protein Coding | 35 | GC13P027427 | 1.081261 | https://www.genecards.org/cgi-bin/carddisp.pl?gene=GTF3A |
| SPAG4 | Sperm Associated Antigen 4 | Protein Coding | 32 | GC20P035615 | 1.081261 | https://www.genecards.org/cgi-bin/carddisp.pl?gene=SPAG4 |
| IKBKG | Inhibitor Of Nuclear Factor Kappa B Kinase Regulatory Subunit Gamma | Protein Coding | 46 | GC0XP154541 | 1.080132 | https://www.genecards.org/cgi-bin/carddisp.pl?gene=IKBKG |
| IFIH1 | Interferon Induced With Helicase C Domain 1 | Protein Coding | 44 | GC02M162267 | 1.079049 | https://www.genecards.org/cgi-bin/carddisp.pl?gene=IFIH1 |
| CD209 | CD209 Molecule | Protein Coding | 40 | GC19M007739 | 1.079049 | https://www.genecards.org/cgi-bin/carddisp.pl?gene=CD209 |
| MX1 | MX Dynamin Like GTPase 1 | Protein Coding | 40 | GC21P041420 | 1.079049 | https://www.genecards.org/cgi-bin/carddisp.pl?gene=MX1 |
| CLEC4M | C-Type Lectin Domain Family 4 Member M | Protein Coding | 38 | GC19P007763 | 1.079049 | https://www.genecards.org/cgi-bin/carddisp.pl?gene=CLEC4M |
| MAVS | Mitochondrial Antiviral Signaling Protein | Protein Coding | 38 | GC20P003850 | 1.079049 | https://www.genecards.org/cgi-bin/carddisp.pl?gene=MAVS |
| SH2D3A | SH2 Domain Containing 3A | Protein Coding | 34 | GC19M006752 | 1.079049 | https://www.genecards.org/cgi-bin/carddisp.pl?gene=SH2D3A |
| SIM1 | SIM BHLH Transcription Factor 1 | Protein Coding | 39 | GC06M100386 | 1.07837 | https://www.genecards.org/cgi-bin/carddisp.pl?gene=SIM1 |
| MIR20B | MicroRNA 20b | RNA Gene | 15 | GC0XM134217 | 1.077191 | https://www.genecards.org/cgi-bin/carddisp.pl?gene=MIR20B |
| EDN3 | Endothelin 3 | Protein Coding | 44 | GC20P059300 | 1.076829 | https://www.genecards.org/cgi-bin/carddisp.pl?gene=EDN3 |
| SOAT1 | Sterol O-Acyltransferase 1 | Protein Coding | 43 | GC01P179262 | 1.076829 | https://www.genecards.org/cgi-bin/carddisp.pl?gene=SOAT1 |
| HP | Haptoglobin | Protein Coding | 42 | GC16P072089 | 1.076829 | https://www.genecards.org/cgi-bin/carddisp.pl?gene=HP |
| ETV4 | ETS Variant Transcription Factor 4 | Protein Coding | 41 | GC17M043527 | 1.076829 | https://www.genecards.org/cgi-bin/carddisp.pl?gene=ETV4 |
| SLC31A1 | Solute Carrier Family 31 Member 1 | Protein Coding | 40 | GC09P113221 | 1.076829 | https://www.genecards.org/cgi-bin/carddisp.pl?gene=SLC31A1 |
| AGR2 | Anterior Gradient 2, Protein Disulphide Isomerase Family Member | Protein Coding | 38 | GC07M016972 | 1.076829 | https://www.genecards.org/cgi-bin/carddisp.pl?gene=AGR2 |
| MIR22 | MicroRNA 22 | RNA Gene | 20 | GC17M001713 | 1.076829 | https://www.genecards.org/cgi-bin/carddisp.pl?gene=MIR22 |
| MIR29B1 | MicroRNA 29b-1 | RNA Gene | 20 | GC07M130877 | 1.076829 | https://www.genecards.org/cgi-bin/carddisp.pl?gene=MIR29B1 |
| ACP1 | Acid Phosphatase 1 | Protein Coding | 41 | GC02P000267 | 1.076325 | https://www.genecards.org/cgi-bin/carddisp.pl?gene=ACP1 |
| PCBP1 | Poly(RC) Binding Protein 1 | Protein Coding | 40 | GC02P070087 | 1.07589 | https://www.genecards.org/cgi-bin/carddisp.pl?gene=PCBP1 |
| BAG2 | BAG Cochaperone 2 | Protein Coding | 38 | GC06P057172 | 1.07589 | https://www.genecards.org/cgi-bin/carddisp.pl?gene=BAG2 |
| CPEB2 | Cytoplasmic Polyadenylation Element Binding Protein 2 | Protein Coding | 33 | GC04P016351 | 1.074971 | https://www.genecards.org/cgi-bin/carddisp.pl?gene=CPEB2 |
| CYP1B1 | Cytochrome P450 Family 1 Subfamily B Member 1 | Protein Coding | 46 | GC02M038066 | 1.073977 | https://www.genecards.org/cgi-bin/carddisp.pl?gene=CYP1B1 |
| P4HA2 | Prolyl 4-Hydroxylase Subunit Alpha 2 | Protein Coding | 44 | GC05M132191 | 1.073977 | https://www.genecards.org/cgi-bin/carddisp.pl?gene=P4HA2 |
| ADAR | Adenosine Deaminase RNA Specific | Protein Coding | 43 | GC01M154582 | 1.073977 | https://www.genecards.org/cgi-bin/carddisp.pl?gene=ADAR |
| IL1R1 | Interleukin 1 Receptor Type 1 | Protein Coding | 43 | GC02P102136 | 1.07384 | https://www.genecards.org/cgi-bin/carddisp.pl?gene=IL1R1 |
| DNM2 | Dynamin 2 | Protein Coding | 47 | GC19P010718 | 1.071759 | https://www.genecards.org/cgi-bin/carddisp.pl?gene=DNM2 |
| IL15 | Interleukin 15 | Protein Coding | 38 | GC04P141636 | 1.071759 | https://www.genecards.org/cgi-bin/carddisp.pl?gene=IL15 |
| PLTP | Phospholipid Transfer Protein | Protein Coding | 41 | GC20M045898 | 1.0712 | https://www.genecards.org/cgi-bin/carddisp.pl?gene=PLTP |
| ESRRB | Estrogen Related Receptor Beta | Protein Coding | 47 | GC14P076310 | 1.070699 | https://www.genecards.org/cgi-bin/carddisp.pl?gene=ESRRB |
| EIF4A1 | Eukaryotic Translation Initiation Factor 4A1 | Protein Coding | 43 | GC17P007572 | 1.070699 | https://www.genecards.org/cgi-bin/carddisp.pl?gene=EIF4A1 |
| CXCR1 | C-X-C Motif Chemokine Receptor 1 | Protein Coding | 41 | GC02M218162 | 1.070699 | https://www.genecards.org/cgi-bin/carddisp.pl?gene=CXCR1 |
| CYP3A4 | Cytochrome P450 Family 3 Subfamily A Member 4 | Protein Coding | 48 | GC07M099759 | 1.067707 | https://www.genecards.org/cgi-bin/carddisp.pl?gene=CYP3A4 |
| SLC11A1 | Solute Carrier Family 11 Member 1 | Protein Coding | 45 | GC02P218382 | 1.067707 | https://www.genecards.org/cgi-bin/carddisp.pl?gene=SLC11A1 |
| CXADR | CXADR Ig-Like Cell Adhesion Molecule | Protein Coding | 41 | GC21P017513 | 1.067707 | https://www.genecards.org/cgi-bin/carddisp.pl?gene=CXADR |
| CEACAM5 | CEA Cell Adhesion Molecule 5 | Protein Coding | 40 | GC19P045093 | 1.067707 | https://www.genecards.org/cgi-bin/carddisp.pl?gene=CEACAM5 |
| INHA | Inhibin Subunit Alpha | Protein Coding | 40 | GC02P219569 | 1.067707 | https://www.genecards.org/cgi-bin/carddisp.pl?gene=INHA |
| TARBP2 | TARBP2 Subunit Of RISC Loading Complex | Protein Coding | 40 | GC12P053499 | 1.067707 | https://www.genecards.org/cgi-bin/carddisp.pl?gene=TARBP2 |
| MAML1 | Mastermind Like Transcriptional Coactivator 1 | Protein Coding | 38 | GC05P179732 | 1.067707 | https://www.genecards.org/cgi-bin/carddisp.pl?gene=MAML1 |
| ANOS1 | Anosmin 1 | Protein Coding | 35 | GC0XM008528 | 1.067707 | https://www.genecards.org/cgi-bin/carddisp.pl?gene=ANOS1 |
| MIR218-1 | MicroRNA 218-1 | RNA Gene | 18 | GC04P020793 | 1.067707 | https://www.genecards.org/cgi-bin/carddisp.pl?gene=MIR218-1 |
| RBBP6 | RB Binding Protein 6, Ubiquitin Ligase | Protein Coding | 38 | GC16P024537 | 1.067643 | https://www.genecards.org/cgi-bin/carddisp.pl?gene=RBBP6 |
| EEF2K | Eukaryotic Elongation Factor 2 Kinase | Protein Coding | 43 | GC16P022217 | 1.065179 | https://www.genecards.org/cgi-bin/carddisp.pl?gene=EEF2K |
| HNF1A | HNF1 Homeobox A | Protein Coding | 43 | GC12P121036 | 1.065179 | https://www.genecards.org/cgi-bin/carddisp.pl?gene=HNF1A |
| SETD7 | SET Domain Containing 7, Histone Lysine Methyltransferase | Protein Coding | 41 | GC04M139495 | 1.065179 | https://www.genecards.org/cgi-bin/carddisp.pl?gene=SETD7 |
| EFNA1 | Ephrin A1 | Protein Coding | 40 | GC01P155127 | 1.065179 | https://www.genecards.org/cgi-bin/carddisp.pl?gene=EFNA1 |
| ASIC5 | Acid Sensing Ion Channel Subunit Family Member 5 | Protein Coding | 31 | GC04M155829 | 1.065179 | https://www.genecards.org/cgi-bin/carddisp.pl?gene=ASIC5 |
| MIR196A2 | MicroRNA 196a-2 | RNA Gene | 21 | GC12P054762 | 1.065179 | https://www.genecards.org/cgi-bin/carddisp.pl?gene=MIR196A2 |
| MIR196A1 | MicroRNA 196a-1 | RNA Gene | 20 | GC17M048632 | 1.065179 | https://www.genecards.org/cgi-bin/carddisp.pl?gene=MIR196A1 |
| BRCA2 | BRCA2 DNA Repair Associated | Protein Coding | 47 | GC13P032315 | 1.061725 | https://www.genecards.org/cgi-bin/carddisp.pl?gene=BRCA2 |
| TTTY15 | Testis-Specific Transcript, Y-Linked 15 | RNA Gene | 15 | GC0YP012538 | 1.060384 | https://www.genecards.org/cgi-bin/carddisp.pl?gene=TTTY15 |
| MATR3 | Matrin 3 | Protein Coding | 40 | GC05P139274 | 1.059524 | https://www.genecards.org/cgi-bin/carddisp.pl?gene=MATR3 |
| RANGAP1 | Ran GTPase Activating Protein 1 | Protein Coding | 40 | GC22M041244 | 1.057117 | https://www.genecards.org/cgi-bin/carddisp.pl?gene=RANGAP1 |
| HEXIM1 | HEXIM P-TEFb Complex Subunit 1 | Protein Coding | 36 | GC17P048084 | 1.057117 | https://www.genecards.org/cgi-bin/carddisp.pl?gene=HEXIM1 |
| PAX2 | Paired Box 2 | Protein Coding | 45 | GC10P100735 | 1.053276 | https://www.genecards.org/cgi-bin/carddisp.pl?gene=PAX2 |
| SART3 | Spliceosome Associated Factor 3, U4/U6 Recycling Protein | Protein Coding | 35 | GC12M108522 | 1.053276 | https://www.genecards.org/cgi-bin/carddisp.pl?gene=SART3 |
| GAD1 | Glutamate Decarboxylase 1 | Protein Coding | 48 | GC02P170813 | 1.052986 | https://www.genecards.org/cgi-bin/carddisp.pl?gene=GAD1 |
| CCR1 | C-C Motif Chemokine Receptor 1 | Protein Coding | 43 | GC03M046218 | 1.052986 | https://www.genecards.org/cgi-bin/carddisp.pl?gene=CCR1 |
| E2F4 | E2F Transcription Factor 4 | Protein Coding | 43 | GC16P067192 | 1.052986 | https://www.genecards.org/cgi-bin/carddisp.pl?gene=E2F4 |
| KCNN2 | Potassium Calcium-Activated Channel Subfamily N Member 2 | Protein Coding | 43 | GC05P114058 | 1.052986 | https://www.genecards.org/cgi-bin/carddisp.pl?gene=KCNN2 |
| FBP2 | Fructose-Bisphosphatase 2 | Protein Coding | 40 | GC09M094558 | 1.052986 | https://www.genecards.org/cgi-bin/carddisp.pl?gene=FBP2 |
| TFF2 | Trefoil Factor 2 | Protein Coding | 39 | GC21M042346 | 1.052986 | https://www.genecards.org/cgi-bin/carddisp.pl?gene=TFF2 |
| FABP12 | Fatty Acid Binding Protein 12 | Protein Coding | 30 | GC08M081524 | 1.052986 | https://www.genecards.org/cgi-bin/carddisp.pl?gene=FABP12 |
| MIR203A | MicroRNA 203a | RNA Gene | 19 | GC14P107933 | 1.052986 | https://www.genecards.org/cgi-bin/carddisp.pl?gene=MIR203A |
| CCL15 | C-C Motif Chemokine Ligand 15 | Protein Coding | 33 | GC17M035996 | 1.051876 | https://www.genecards.org/cgi-bin/carddisp.pl?gene=CCL15 |
| CCT3 | Chaperonin Containing TCP1 Subunit 3 | Protein Coding | 39 | GC01M156308 | 1.050877 | https://www.genecards.org/cgi-bin/carddisp.pl?gene=CCT3 |
| DYRK2 | Dual Specificity Tyrosine Phosphorylation Regulated Kinase 2 | Protein Coding | 43 | GC12P067558 | 1.050765 | https://www.genecards.org/cgi-bin/carddisp.pl?gene=DYRK2 |
| DUSP6 | Dual Specificity Phosphatase 6 | Protein Coding | 46 | GC12M089347 | 1.05001 | https://www.genecards.org/cgi-bin/carddisp.pl?gene=DUSP6 |
| HTR3A | 5-Hydroxytryptamine Receptor 3A | Protein Coding | 44 | GC11P113974 | 1.05001 | https://www.genecards.org/cgi-bin/carddisp.pl?gene=HTR3A |
| MIR137 | MicroRNA 137 | RNA Gene | 18 | GC01M098046 | 1.05001 | https://www.genecards.org/cgi-bin/carddisp.pl?gene=MIR137 |
| SUMO2 | Small Ubiquitin Like Modifier 2 | Protein Coding | 37 | GC17M075165 | 1.049206 | https://www.genecards.org/cgi-bin/carddisp.pl?gene=SUMO2 |
| MPO | Myeloperoxidase | Protein Coding | 48 | GC17M058269 | 1.048752 | https://www.genecards.org/cgi-bin/carddisp.pl?gene=MPO |
| EMD | Emerin | Protein Coding | 43 | GC0XP154379 | 1.047874 | https://www.genecards.org/cgi-bin/carddisp.pl?gene=EMD |
| MYO1B | Myosin IB | Protein Coding | 36 | GC02P191246 | 1.047874 | https://www.genecards.org/cgi-bin/carddisp.pl?gene=MYO1B |
| MTHFR | Methylenetetrahydrofolate Reductase | Protein Coding | 46 | GC01M011785 | 1.046986 | https://www.genecards.org/cgi-bin/carddisp.pl?gene=MTHFR |
| PDGFC | Platelet Derived Growth Factor C | Protein Coding | 40 | GC04M156760 | 1.044657 | https://www.genecards.org/cgi-bin/carddisp.pl?gene=PDGFC |
| NCOR1 | Nuclear Receptor Corepressor 1 | Protein Coding | 42 | GC17M016029 | 1.043718 | https://www.genecards.org/cgi-bin/carddisp.pl?gene=NCOR1 |
| NME2 | NME/NM23 Nucleoside Diphosphate Kinase 2 | Protein Coding | 44 | GC17P051165 | 1.040598 | https://www.genecards.org/cgi-bin/carddisp.pl?gene=NME2 |
| YBX3 | Y-Box Binding Protein 3 | Protein Coding | 37 | GC12M018226 | 1.040598 | https://www.genecards.org/cgi-bin/carddisp.pl?gene=YBX3 |
| CTF1 | Cardiotrophin 1 | Protein Coding | 36 | GC16P037823 | 1.040598 | https://www.genecards.org/cgi-bin/carddisp.pl?gene=CTF1 |
| CACUL1 | CDK2 Associated Cullin Domain 1 | Protein Coding | 31 | GC10M118674 | 1.040598 | https://www.genecards.org/cgi-bin/carddisp.pl?gene=CACUL1 |
| PRKACA | Protein Kinase CAMP-Activated Catalytic Subunit Alpha | Protein Coding | 49 | GC19M014311 | 1.040166 | https://www.genecards.org/cgi-bin/carddisp.pl?gene=PRKACA |
| SLC5A1 | Solute Carrier Family 5 Member 1 | Protein Coding | 47 | GC22P032043 | 1.040166 | https://www.genecards.org/cgi-bin/carddisp.pl?gene=SLC5A1 |
| CDK8 | Cyclin Dependent Kinase 8 | Protein Coding | 46 | GC13P026254 | 1.040166 | https://www.genecards.org/cgi-bin/carddisp.pl?gene=CDK8 |
| TDO2 | Tryptophan 2,3-Dioxygenase | Protein Coding | 42 | GC04P155854 | 1.040166 | https://www.genecards.org/cgi-bin/carddisp.pl?gene=TDO2 |
| NR6A1 | Nuclear Receptor Subfamily 6 Group A Member 1 | Protein Coding | 39 | GC09M124517 | 1.040166 | https://www.genecards.org/cgi-bin/carddisp.pl?gene=NR6A1 |
| RND3 | Rho Family GTPase 3 | Protein Coding | 39 | GC02M150468 | 1.040166 | https://www.genecards.org/cgi-bin/carddisp.pl?gene=RND3 |
| MED26 | Mediator Complex Subunit 26 | Protein Coding | 34 | GC19M016574 | 1.040166 | https://www.genecards.org/cgi-bin/carddisp.pl?gene=MED26 |
| JUND | JunD Proto-Oncogene, AP-1 Transcription Factor Subunit | Protein Coding | 40 | GC19M018279 | 1.04012 | https://www.genecards.org/cgi-bin/carddisp.pl?gene=JUND |
| TRPC4 | Transient Receptor Potential Cation Channel Subfamily C Member 4 | Protein Coding | 43 | GC13M037636 | 1.039258 | https://www.genecards.org/cgi-bin/carddisp.pl?gene=TRPC4 |
| CXCL2 | C-X-C Motif Chemokine Ligand 2 | Protein Coding | 38 | GC04M074097 | 1.039258 | https://www.genecards.org/cgi-bin/carddisp.pl?gene=CXCL2 |
| MIR190A | MicroRNA 190a | RNA Gene | 19 | GC15P087985 | 1.039258 | https://www.genecards.org/cgi-bin/carddisp.pl?gene=MIR190A |
| LDHB | Lactate Dehydrogenase B | Protein Coding | 44 | GC12M021635 | 1.039027 | https://www.genecards.org/cgi-bin/carddisp.pl?gene=LDHB |
| IGF2BP1 | Insulin Like Growth Factor 2 MRNA Binding Protein 1 | Protein Coding | 38 | GC17P048997 | 1.039027 | https://www.genecards.org/cgi-bin/carddisp.pl?gene=IGF2BP1 |
| NPTX1 | Neuronal Pentraxin 1 | Protein Coding | 36 | GC17M080466 | 1.039027 | https://www.genecards.org/cgi-bin/carddisp.pl?gene=NPTX1 |
| ABCC9 | ATP Binding Cassette Subfamily C Member 9 | Protein Coding | 43 | GC12M021797 | 1.037771 | https://www.genecards.org/cgi-bin/carddisp.pl?gene=ABCC9 |
| LAMA3 | Laminin Subunit Alpha 3 | Protein Coding | 43 | GC18P023689 | 1.037771 | https://www.genecards.org/cgi-bin/carddisp.pl?gene=LAMA3 |
| MED12 | Mediator Complex Subunit 12 | Protein Coding | 43 | GC0XP071118 | 1.037771 | https://www.genecards.org/cgi-bin/carddisp.pl?gene=MED12 |
| MED23 | Mediator Complex Subunit 23 | Protein Coding | 40 | GC06M131573 | 1.037771 | https://www.genecards.org/cgi-bin/carddisp.pl?gene=MED23 |
| PER1 | Period Circadian Regulator 1 | Protein Coding | 40 | GC17M009820 | 1.037771 | https://www.genecards.org/cgi-bin/carddisp.pl?gene=PER1 |
| MED15 | Mediator Complex Subunit 15 | Protein Coding | 39 | GC22P030416 | 1.037771 | https://www.genecards.org/cgi-bin/carddisp.pl?gene=MED15 |
| MED14 | Mediator Complex Subunit 14 | Protein Coding | 37 | GC0XM040648 | 1.037771 | https://www.genecards.org/cgi-bin/carddisp.pl?gene=MED14 |
| RAB11FIP4 | RAB11 Family Interacting Protein 4 | Protein Coding | 35 | GC17P031391 | 1.037771 | https://www.genecards.org/cgi-bin/carddisp.pl?gene=RAB11FIP4 |
| CHCHD4 | Coiled-Coil-Helix-Coiled-Coil-Helix Domain Containing 4 | Protein Coding | 34 | GC03M018773 | 1.037771 | https://www.genecards.org/cgi-bin/carddisp.pl?gene=CHCHD4 |
| MT-ND6 | Mitochondrially Encoded NADH:Ubiquinone Oxidoreductase Core Subunit 6 | Protein Coding | 32 | GCMTM014151 | 1.037771 | https://www.genecards.org/cgi-bin/carddisp.pl?gene=MT-ND6 |
| CYTOR | Cytoskeleton Regulator RNA | RNA Gene | 18 | GC02P087801 | 1.037771 | https://www.genecards.org/cgi-bin/carddisp.pl?gene=CYTOR |
| HADHA | Hydroxyacyl-CoA Dehydrogenase Trifunctional Multienzyme Complex Subunit Alpha | Protein Coding | 44 | GC02M026190 | 1.037323 | https://www.genecards.org/cgi-bin/carddisp.pl?gene=HADHA |
| ESRRA | Estrogen Related Receptor Alpha | Protein Coding | 47 | GC11P064305 | 1.03568 | https://www.genecards.org/cgi-bin/carddisp.pl?gene=ESRRA |
| RICTOR | RPTOR Independent Companion Of MTOR Complex 2 | Protein Coding | 43 | GC05M038937 | 1.03568 | https://www.genecards.org/cgi-bin/carddisp.pl?gene=RICTOR |
| MIR205 | MicroRNA 205 | RNA Gene | 19 | GC01P209432 | 1.03568 | https://www.genecards.org/cgi-bin/carddisp.pl?gene=MIR205 |
| IRS4 | Insulin Receptor Substrate 4 | Protein Coding | 39 | GC0XM108720 | 1.035576 | https://www.genecards.org/cgi-bin/carddisp.pl?gene=IRS4 |
| EFTUD2 | Elongation Factor Tu GTP Binding Domain Containing 2 | Protein Coding | 40 | GC17M044852 | 1.03488 | https://www.genecards.org/cgi-bin/carddisp.pl?gene=EFTUD2 |
| GNB1 | G Protein Subunit Beta 1 | Protein Coding | 43 | GC01M001785 | 1.031481 | https://www.genecards.org/cgi-bin/carddisp.pl?gene=GNB1 |
| SRPK1 | SRSF Protein Kinase 1 | Protein Coding | 43 | GC06M056660 | 1.031174 | https://www.genecards.org/cgi-bin/carddisp.pl?gene=SRPK1 |
| TLR9 | Toll Like Receptor 9 | Protein Coding | 43 | GC03M052222 | 1.029424 | https://www.genecards.org/cgi-bin/carddisp.pl?gene=TLR9 |
| KCNK9 | Potassium Two Pore Domain Channel Subfamily K Member 9 | Protein Coding | 44 | GC08M139585 | 1.028109 | https://www.genecards.org/cgi-bin/carddisp.pl?gene=KCNK9 |
| ITGA3 | Integrin Subunit Alpha 3 | Protein Coding | 44 | GC17P050055 | 1.028083 | https://www.genecards.org/cgi-bin/carddisp.pl?gene=ITGA3 |
| CANT1 | Calcium Activated Nucleotidase 1 | Protein Coding | 42 | GC17M078992 | 1.028083 | https://www.genecards.org/cgi-bin/carddisp.pl?gene=CANT1 |
| HMBS | Hydroxymethylbilane Synthase | Protein Coding | 42 | GC11P119084 | 1.028083 | https://www.genecards.org/cgi-bin/carddisp.pl?gene=HMBS |
| LRIG1 | Leucine Rich Repeats And Immunoglobulin Like Domains 1 | Protein Coding | 40 | GC03M066379 | 1.028083 | https://www.genecards.org/cgi-bin/carddisp.pl?gene=LRIG1 |
| UROS | Uroporphyrinogen III Synthase | Protein Coding | 39 | GC10M125784 | 1.028083 | https://www.genecards.org/cgi-bin/carddisp.pl?gene=UROS |
| RRAD | RRAD, Ras Related Glycolysis Inhibitor And Calcium Channel Regulator | Protein Coding | 38 | GC16M066928 | 1.028083 | https://www.genecards.org/cgi-bin/carddisp.pl?gene=RRAD |
| GSK3A | Glycogen Synthase Kinase 3 Alpha | Protein Coding | 47 | GC19M057002 | 1.026612 | https://www.genecards.org/cgi-bin/carddisp.pl?gene=GSK3A |
| WNT10B | Wnt Family Member 10B | Protein Coding | 46 | GC12M048965 | 1.026612 | https://www.genecards.org/cgi-bin/carddisp.pl?gene=WNT10B |
| DIABLO | Diablo IAP-Binding Mitochondrial Protein | Protein Coding | 45 | GC12M122208 | 1.026612 | https://www.genecards.org/cgi-bin/carddisp.pl?gene=DIABLO |
| SMPD1 | Sphingomyelin Phosphodiesterase 1 | Protein Coding | 45 | GC11P006390 | 1.026612 | https://www.genecards.org/cgi-bin/carddisp.pl?gene=SMPD1 |
| CD14 | CD14 Molecule | Protein Coding | 43 | GC05M140631 | 1.026612 | https://www.genecards.org/cgi-bin/carddisp.pl?gene=CD14 |
| CCNC | Cyclin C | Protein Coding | 39 | GC06M099542 | 1.026612 | https://www.genecards.org/cgi-bin/carddisp.pl?gene=CCNC |
| SH3KBP1 | SH3 Domain Containing Kinase Binding Protein 1 | Protein Coding | 39 | GC0XM019552 | 1.026612 | https://www.genecards.org/cgi-bin/carddisp.pl?gene=SH3KBP1 |
| MCAM | Melanoma Cell Adhesion Molecule | Protein Coding | 38 | GC11M119308 | 1.026612 | https://www.genecards.org/cgi-bin/carddisp.pl?gene=MCAM |
| MIR30C1 | MicroRNA 30c-1 | RNA Gene | 21 | GC01P040757 | 1.026612 | https://www.genecards.org/cgi-bin/carddisp.pl?gene=MIR30C1 |
| MIR103A1 | MicroRNA 103a-1 | RNA Gene | 17 | GC05M168560 | 1.026612 | https://www.genecards.org/cgi-bin/carddisp.pl?gene=MIR103A1 |
| ALOX5 | Arachidonate 5-Lipoxygenase | Protein Coding | 47 | GC10P045374 | 1.022861 | https://www.genecards.org/cgi-bin/carddisp.pl?gene=ALOX5 |
| FBXW7 | F-Box And WD Repeat Domain Containing 7 | Protein Coding | 43 | GC04M152321 | 1.022861 | https://www.genecards.org/cgi-bin/carddisp.pl?gene=FBXW7 |
| COX5A | Cytochrome C Oxidase Subunit 5A | Protein Coding | 42 | GC15M074919 | 1.022861 | https://www.genecards.org/cgi-bin/carddisp.pl?gene=COX5A |
| CCT5 | Chaperonin Containing TCP1 Subunit 5 | Protein Coding | 42 | GC05P010236 | 1.022861 | https://www.genecards.org/cgi-bin/carddisp.pl?gene=CCT5 |
| ENPEP | Glutamyl Aminopeptidase | Protein Coding | 42 | GC04P110365 | 1.022861 | https://www.genecards.org/cgi-bin/carddisp.pl?gene=ENPEP |
| NDUFA13 | NADH:Ubiquinone Oxidoreductase Subunit A13 | Protein Coding | 42 | GC19P019515 | 1.022861 | https://www.genecards.org/cgi-bin/carddisp.pl?gene=NDUFA13 |
| TFAM | Transcription Factor A, Mitochondrial | Protein Coding | 42 | GC10P058385 | 1.022861 | https://www.genecards.org/cgi-bin/carddisp.pl?gene=TFAM |
| RPS7 | Ribosomal Protein S7 | Protein Coding | 39 | GC02P003575 | 1.022861 | https://www.genecards.org/cgi-bin/carddisp.pl?gene=RPS7 |
| SCN5A | Sodium Voltage-Gated Channel Alpha Subunit 5 | Protein Coding | 48 | GC03M038549 | 1.02199 | https://www.genecards.org/cgi-bin/carddisp.pl?gene=SCN5A |
| BMX | BMX Non-Receptor Tyrosine Kinase | Protein Coding | 43 | GC0XP015392 | 1.02199 | https://www.genecards.org/cgi-bin/carddisp.pl?gene=BMX |
| TFR2 | Transferrin Receptor 2 | Protein Coding | 43 | GC07M100620 | 1.02199 | https://www.genecards.org/cgi-bin/carddisp.pl?gene=TFR2 |
| PSENEN | Presenilin Enhancer, Gamma-Secretase Subunit | Protein Coding | 42 | GC19P044753 | 1.020911 | https://www.genecards.org/cgi-bin/carddisp.pl?gene=PSENEN |
| RNASET2 | Ribonuclease T2 | Protein Coding | 42 | GC06M166924 | 1.020911 | https://www.genecards.org/cgi-bin/carddisp.pl?gene=RNASET2 |
| KCNN3 | Potassium Calcium-Activated Channel Subfamily N Member 3 | Protein Coding | 42 | GC01M154697 | 1.018618 | https://www.genecards.org/cgi-bin/carddisp.pl?gene=KCNN3 |
| CDK6 | Cyclin Dependent Kinase 6 | Protein Coding | 51 | GC07M092604 | 1.017773 | https://www.genecards.org/cgi-bin/carddisp.pl?gene=CDK6 |
| CTSK | Cathepsin K | Protein Coding | 47 | GC01M150978 | 1.017773 | https://www.genecards.org/cgi-bin/carddisp.pl?gene=CTSK |
| SRPRB | SRP Receptor Subunit Beta | Protein Coding | 36 | GC03P133784 | 1.017773 | https://www.genecards.org/cgi-bin/carddisp.pl?gene=SRPRB |
| PFKM | Phosphofructokinase, Muscle | Protein Coding | 47 | GC12P048105 | 1.0173 | https://www.genecards.org/cgi-bin/carddisp.pl?gene=PFKM |
| MAPKAPK2 | MAPK Activated Protein Kinase 2 | Protein Coding | 46 | GC01P206684 | 1.016433 | https://www.genecards.org/cgi-bin/carddisp.pl?gene=MAPKAPK2 |
| FBLN5 | Fibulin 5 | Protein Coding | 44 | GC14M091869 | 1.016433 | https://www.genecards.org/cgi-bin/carddisp.pl?gene=FBLN5 |
| C3AR1 | Complement C3a Receptor 1 | Protein Coding | 42 | GC12M008058 | 1.016433 | https://www.genecards.org/cgi-bin/carddisp.pl?gene=C3AR1 |
| MYLK3 | Myosin Light Chain Kinase 3 | Protein Coding | 40 | GC16M046871 | 1.016433 | https://www.genecards.org/cgi-bin/carddisp.pl?gene=MYLK3 |
| MIR1281 | MicroRNA 1281 | RNA Gene | 14 | GC22P041092 | 1.015926 | https://www.genecards.org/cgi-bin/carddisp.pl?gene=MIR1281 |
| MIR7703 | MicroRNA 7703 | RNA Gene | 9 | GC14M024144 | 1.015926 | https://www.genecards.org/cgi-bin/carddisp.pl?gene=MIR7703 |
| RTN4 | Reticulon 4 | Protein Coding | 42 | GC02M054934 | 1.014621 | https://www.genecards.org/cgi-bin/carddisp.pl?gene=RTN4 |
| H3C14 | H3 Clustered Histone 14 | Protein Coding | 26 | GC01M150438 | 1.014409 | https://www.genecards.org/cgi-bin/carddisp.pl?gene=H3C14 |
| HSPA2 | Heat Shock Protein Family A (Hsp70) Member 2 | Protein Coding | 42 | GC14P064535 | 1.012547 | https://www.genecards.org/cgi-bin/carddisp.pl?gene=HSPA2 |
| KDM6B | Lysine Demethylase 6B | Protein Coding | 42 | GC17P007834 | 1.012547 | https://www.genecards.org/cgi-bin/carddisp.pl?gene=KDM6B |
| CRHR1 | Corticotropin Releasing Hormone Receptor 1 | Protein Coding | 43 | GC17P045784 | 1.012181 | https://www.genecards.org/cgi-bin/carddisp.pl?gene=CRHR1 |
| P2RY11 | Purinergic Receptor P2Y11 | Protein Coding | 42 | GC19P010184 | 1.012181 | https://www.genecards.org/cgi-bin/carddisp.pl?gene=P2RY11 |
| SEMA4B | Semaphorin 4B | Protein Coding | 37 | GC15P090160 | 1.012181 | https://www.genecards.org/cgi-bin/carddisp.pl?gene=SEMA4B |
| TP53INP1 | Tumor Protein P53 Inducible Nuclear Protein 1 | Protein Coding | 36 | GC08M094925 | 1.012181 | https://www.genecards.org/cgi-bin/carddisp.pl?gene=TP53INP1 |
| MIR410 | MicroRNA 410 | RNA Gene | 17 | GC14P108179 | 1.012181 | https://www.genecards.org/cgi-bin/carddisp.pl?gene=MIR410 |
| RFC2 | Replication Factor C Subunit 2 | Protein Coding | 43 | GC07M074231 | 1.010443 | https://www.genecards.org/cgi-bin/carddisp.pl?gene=RFC2 |
| PDGFRA | Platelet Derived Growth Factor Receptor Alpha | Protein Coding | 51 | GC04P054229 | 1.009307 | https://www.genecards.org/cgi-bin/carddisp.pl?gene=PDGFRA |
| RUVBL1 | RuvB Like AAA ATPase 1 | Protein Coding | 41 | GC03M128064 | 1.009307 | https://www.genecards.org/cgi-bin/carddisp.pl?gene=RUVBL1 |
| CCT4 | Chaperonin Containing TCP1 Subunit 4 | Protein Coding | 38 | GC02M061868 | 1.009307 | https://www.genecards.org/cgi-bin/carddisp.pl?gene=CCT4 |
| SOCS2 | Suppressor Of Cytokine Signaling 2 | Protein Coding | 42 | GC12P093569 | 1.009148 | https://www.genecards.org/cgi-bin/carddisp.pl?gene=SOCS2 |
| PIAS2 | Protein Inhibitor Of Activated STAT 2 | Protein Coding | 42 | GC18M046808 | 1.007866 | https://www.genecards.org/cgi-bin/carddisp.pl?gene=PIAS2 |
| JAG2 | Jagged Canonical Notch Ligand 2 | Protein Coding | 40 | GC14M105140 | 1.007866 | https://www.genecards.org/cgi-bin/carddisp.pl?gene=JAG2 |
| UBQLN1 | Ubiquilin 1 | Protein Coding | 40 | GC09M083659 | 1.007515 | https://www.genecards.org/cgi-bin/carddisp.pl?gene=UBQLN1 |
| OGG1 | 8-Oxoguanine DNA Glycosylase | Protein Coding | 43 | GC03P011602 | 1.006891 | https://www.genecards.org/cgi-bin/carddisp.pl?gene=OGG1 |
| HNRNPD | Heterogeneous Nuclear Ribonucleoprotein D | Protein Coding | 41 | GC04M082352 | 1.00558 | https://www.genecards.org/cgi-bin/carddisp.pl?gene=HNRNPD |
| FOXC1 | Forkhead Box C1 | Protein Coding | 41 | GC06P001610 | 1.00558 | https://www.genecards.org/cgi-bin/carddisp.pl?gene=FOXC1 |
| YTHDF2 | YTH N6-Methyladenosine RNA Binding Protein 2 | Protein Coding | 37 | GC01P028751 | 1.00558 | https://www.genecards.org/cgi-bin/carddisp.pl?gene=YTHDF2 |
| CCAR2 | Cell Cycle And Apoptosis Regulator 2 | Protein Coding | 35 | GC08P022604 | 1.00558 | https://www.genecards.org/cgi-bin/carddisp.pl?gene=CCAR2 |
| MAP2K3 | Mitogen-Activated Protein Kinase Kinase 3 | Protein Coding | 47 | GC17P034165 | 1.004239 | https://www.genecards.org/cgi-bin/carddisp.pl?gene=MAP2K3 |
| ZEB2 | Zinc Finger E-Box Binding Homeobox 2 | Protein Coding | 46 | GC02M144384 | 1.004239 | https://www.genecards.org/cgi-bin/carddisp.pl?gene=ZEB2 |
| TRAF5 | TNF Receptor Associated Factor 5 | Protein Coding | 40 | GC01P211326 | 1.004239 | https://www.genecards.org/cgi-bin/carddisp.pl?gene=TRAF5 |
| PITRM1 | Pitrilysin Metallopeptidase 1 | Protein Coding | 39 | GC10M003138 | 1.004239 | https://www.genecards.org/cgi-bin/carddisp.pl?gene=PITRM1 |
| FTMT | Ferritin Mitochondrial | Protein Coding | 36 | GC05P121851 | 1.002822 | https://www.genecards.org/cgi-bin/carddisp.pl?gene=FTMT |
| MIR485 | MicroRNA 485 | RNA Gene | 18 | GC14P108182 | 1.002822 | https://www.genecards.org/cgi-bin/carddisp.pl?gene=MIR485 |
| PAX3 | Paired Box 3 | Protein Coding | 45 | GC02M222199 | 1.002262 | https://www.genecards.org/cgi-bin/carddisp.pl?gene=PAX3 |
| HTRA2 | HtrA Serine Peptidase 2 | Protein Coding | 44 | GC02P074529 | 1.002262 | https://www.genecards.org/cgi-bin/carddisp.pl?gene=HTRA2 |
| ANKDD1A | Ankyrin Repeat And Death Domain Containing 1A | Protein Coding | 30 | GC15P087001 | 1.002262 | https://www.genecards.org/cgi-bin/carddisp.pl?gene=ANKDD1A |

Supplementary table 2. Differentially expressed hypoxia-related genes between tumor and adjacent normal tissues.

| gene | conMean | treatMean | logFC | pValue |
| --- | --- | --- | --- | --- |
| EGLN3 | 2.370956 | 6.78106 | 1.516042 | 4.56E-16 |
| EPAS1 | 77.26144 | 21.77397 | -1.82714 | 2.73E-54 |
| CA9 | 0.157568 | 3.775633 | 4.582675 | 0.001494 |
| EPO | 0.055854 | 0.797978 | 3.836621 | 3.99E-08 |
| JUN | 163.7453 | 62.55144 | -1.38834 | 1.11E-37 |
| SLC2A1 | 9.61176 | 25.03035 | 1.380806 | 2.21E-30 |
| IGF1 | 1.877095 | 0.380827 | -2.30129 | 2.37E-47 |
| IL6 | 9.343326 | 1.491208 | -2.64746 | 2.09E-32 |
| EDN1 | 16.51297 | 6.505651 | -1.34383 | 7.17E-35 |
| FOS | 429.4156 | 79.7593 | -2.42865 | 1.04E-43 |
| PTGS2 | 4.669382 | 1.534014 | -1.60592 | 8.65E-40 |
| ADM | 12.59772 | 4.61759 | -1.44795 | 9.74E-33 |
| EGFR | 16.7286 | 5.270976 | -1.66618 | 2.67E-58 |
| ANGPTL4 | 27.09689 | 7.664541 | -1.82186 | 1.26E-37 |
| SIAH2 | 14.22779 | 44.63592 | 1.649494 | 6.19E-39 |
| EGR1 | 509.5769 | 83.0174 | -2.61781 | 2.20E-50 |
| LEP | 96.68836 | 1.682883 | -5.84434 | 7.66E-54 |
| CXCL12 | 74.52505 | 20.845 | -1.83802 | 5.38E-54 |
| TEK | 9.27674 | 2.820327 | -1.71776 | 3.32E-46 |
| FGF2 | 9.856209 | 1.610133 | -2.61385 | 2.19E-57 |
| MMP9 | 7.452255 | 46.95062 | 2.655395 | 8.17E-31 |
| PFKFB4 | 0.962903 | 2.241861 | 1.219235 | 3.73E-41 |
| TLR4 | 9.487711 | 3.651121 | -1.37772 | 1.13E-40 |
| ARNT2 | 2.485164 | 9.347445 | 1.911231 | 2.05E-22 |
| MIF | 13.156 | 36.86608 | 1.486573 | 7.06E-31 |
| SPP1 | 25.57757 | 119.5842 | 2.225075 | 6.11E-26 |
| FZD4 | 37.16296 | 6.651284 | -2.48216 | 9.46E-50 |
| CHRDL1 | 84.21698 | 4.531257 | -4.21613 | 1.57E-60 |
| ZFP36 | 338.8834 | 80.2068 | -2.07899 | 2.37E-42 |
| CAT | 94.55895 | 25.96393 | -1.86471 | 9.18E-59 |
| EIF4EBP1 | 20.72008 | 54.01679 | 1.382379 | 1.95E-18 |
| ANGPT1 | 4.1174 | 0.660492 | -2.64012 | 7.68E-52 |
| PLAUR | 2.850974 | 9.154446 | 1.683018 | 8.74E-41 |
| ESR1 | 13.05474 | 40.72953 | 1.641501 | 1.38E-08 |
| DPP4 | 6.328341 | 2.312364 | -1.45246 | 1.47E-34 |
| NDN | 24.47705 | 7.242246 | -1.75692 | 2.41E-55 |
| ERBB2 | 25.14019 | 86.89832 | 1.789333 | 8.86E-14 |
| KCNB1 | 2.361645 | 0.366035 | -2.68974 | 8.41E-56 |
| PPARG | 22.0181 | 2.391431 | -3.20274 | 3.42E-55 |
| CDH5 | 22.52734 | 7.884883 | -1.51452 | 1.30E-46 |
| TYMP | 9.299436 | 32.70865 | 1.814457 | 2.68E-39 |
| ABCB1 | 3.573704 | 0.873909 | -2.03187 | 7.58E-48 |
| CD36 | 141.059 | 9.055138 | -3.96142 | 1.73E-57 |
| S100B | 46.22302 | 7.22357 | -2.67783 | 3.42E-54 |
| FN1 | 38.94658 | 296.4148 | 2.928049 | 2.18E-50 |
| PPARA | 3.249548 | 1.325634 | -1.29356 | 2.23E-45 |
| MMP14 | 43.81605 | 105.6743 | 1.270093 | 1.91E-27 |
| ADIPOQ | 196.1037 | 6.692549 | -4.87292 | 1.79E-49 |
| SNRPB | 61.1312 | 140.5123 | 1.200716 | 1.94E-47 |
| MET | 12.55045 | 4.042174 | -1.63454 | 1.19E-49 |
| VEGFD | 15.66123 | 0.564003 | -4.79535 | 4.70E-66 |
| BIRC5 | 1.22072 | 13.31899 | 3.44768 | 3.93E-56 |
| PLAU | 13.32259 | 32.88579 | 1.30359 | 1.77E-18 |
| TRIB3 | 4.931997 | 14.73741 | 1.579239 | 2.14E-43 |
| GDF15 | 2.262445 | 11.12278 | 2.297563 | 3.79E-16 |
| CCND1 | 51.54904 | 129.3166 | 1.32689 | 3.60E-11 |
| AQP1 | 166.4615 | 41.20162 | -2.01442 | 1.39E-58 |
| MMP1 | 0.217107 | 17.31501 | 6.317471 | 4.98E-50 |
| CYGB | 11.96511 | 5.043407 | -1.24636 | 3.61E-33 |
| F3 | 29.43118 | 9.154945 | -1.68472 | 7.61E-48 |
| AURKA | 1.118717 | 9.50309 | 3.086552 | 5.54E-61 |
| CCN1 | 248.7557 | 81.8697 | -1.60333 | 1.98E-35 |
| PCNA | 33.77744 | 86.90723 | 1.363416 | 4.43E-52 |
| TRPC6 | 1.899188 | 0.789061 | -1.26717 | 3.92E-47 |
| ACO1 | 23.67379 | 6.634773 | -1.83517 | 4.02E-40 |
| PARP1 | 18.67734 | 43.33571 | 1.214267 | 6.79E-55 |
| ENO2 | 4.403077 | 11.7423 | 1.41513 | 1.08E-14 |
| KAT2B | 7.702017 | 3.168133 | -1.2816 | 4.46E-47 |
| MUC1 | 24.38929 | 131.3251 | 2.428822 | 1.49E-30 |
| PFKFB3 | 81.24611 | 23.50756 | -1.78917 | 1.39E-35 |
| POSTN | 55.65325 | 229.9382 | 2.046708 | 7.72E-33 |
| LEPR | 7.983098 | 1.116706 | -2.8377 | 6.36E-59 |
| TXNIP | 622.0039 | 165.9179 | -1.90645 | 1.37E-57 |
| PECAM1 | 64.74103 | 22.50161 | -1.52465 | 1.40E-43 |
| DUSP1 | 302.4901 | 80.76178 | -1.90514 | 3.95E-46 |
| EGF | 4.936181 | 1.769904 | -1.47972 | 7.27E-15 |
| CAV1 | 185.9222 | 19.74364 | -3.23524 | 2.69E-64 |
| HSPB1 | 117.61 | 346.4009 | 1.558432 | 6.95E-24 |
| PHLDA2 | 3.813185 | 9.362327 | 1.295871 | 4.87E-17 |
| PSMD3 | 18.90209 | 50.123 | 1.406927 | 9.41E-38 |
| VIM | 460.5518 | 183.504 | -1.32755 | 2.84E-39 |
| BMP2 | 2.943694 | 0.647123 | -2.18552 | 8.50E-49 |
| DEPP1 | 112.394 | 26.79904 | -2.06831 | 5.60E-46 |
| KCNJ11 | 2.42227 | 5.777247 | 1.254022 | 1.46E-15 |
| CXCL10 | 4.148094 | 52.40519 | 3.659189 | 1.96E-42 |
| CEBPA | 26.07417 | 6.503707 | -2.00329 | 4.91E-22 |
| E2F1 | 1.609154 | 9.828164 | 2.610619 | 5.75E-53 |
| UBE2T | 2.598751 | 23.57977 | 3.181659 | 1.49E-62 |
| MAPK10 | 2.85808 | 0.771693 | -1.88895 | 9.45E-35 |
| FOXM1 | 0.978445 | 10.21762 | 3.384424 | 1.23E-57 |
| PRKN | 1.88282 | 0.562558 | -1.74282 | 2.96E-58 |
| PIK3R1 | 37.49895 | 14.148 | -1.40625 | 4.33E-45 |
| TUFT1 | 6.416269 | 17.73297 | 1.466628 | 1.38E-46 |
| ABCG2 | 2.608225 | 0.945597 | -1.46377 | 1.64E-41 |
| ATG9B | 0.185184 | 0.567158 | 1.614788 | 1.29E-22 |
| CA12 | 20.97365 | 66.88325 | 1.673067 | 1.43E-15 |
| CD34 | 30.72352 | 7.63107 | -2.00939 | 1.13E-58 |
| CDKN3 | 0.812975 | 5.961689 | 2.874439 | 2.36E-60 |
| CCR7 | 1.150025 | 3.692576 | 1.682962 | 8.27E-10 |
| MKI67 | 1.092459 | 8.773531 | 3.005579 | 1.96E-54 |
| AGT | 3.104572 | 10.53214 | 1.762332 | 0.00362 |
| PMAIP1 | 2.645344 | 8.83271 | 1.739401 | 2.20E-25 |
| BMP6 | 5.280729 | 1.575031 | -1.74536 | 3.76E-49 |
| PRNP | 82.6614 | 31.83775 | -1.37648 | 1.80E-51 |
| APOLD1 | 18.43269 | 4.85937 | -1.92343 | 1.40E-31 |
| STAT1 | 28.80679 | 69.12021 | 1.262699 | 2.86E-22 |
| ROBO4 | 10.39501 | 2.988613 | -1.79834 | 1.28E-52 |
| SELE | 5.903002 | 2.325498 | -1.34391 | 3.93E-05 |
| NDRG2 | 55.98843 | 12.1192 | -2.20783 | 5.13E-57 |
| RUNX2 | 1.178529 | 2.793103 | 1.244881 | 7.76E-22 |
| CCNA2 | 1.354245 | 9.417432 | 2.797845 | 4.24E-55 |
| LRP1 | 57.19391 | 19.90205 | -1.52294 | 3.00E-36 |
| TF | 12.07872 | 2.81749 | -2.09999 | 8.19E-52 |
| HOTAIR | 0.504855 | 3.149839 | 2.641338 | 2.10E-17 |
| ASCL2 | 0.688413 | 2.730417 | 1.987775 | 1.75E-19 |
| TUBB3 | 0.074642 | 0.903158 | 3.596927 | 6.57E-47 |
| NR4A1 | 35.72567 | 10.08808 | -1.82431 | 9.29E-30 |
| VWF | 68.28366 | 17.95732 | -1.92697 | 2.22E-46 |
| RYR1 | 2.356337 | 0.546736 | -2.10763 | 5.85E-24 |
| NT5E | 10.29259 | 4.419914 | -1.21952 | 1.38E-29 |
| SIM2 | 0.275192 | 1.336047 | 2.279459 | 1.44E-09 |
| PTGIS | 17.7451 | 4.763403 | -1.89736 | 2.95E-37 |
| TFF3 | 44.54628 | 229.2801 | 2.363734 | 1.29E-08 |
| TRPA1 | 0.032703 | 2.834245 | 6.437415 | 1.50E-22 |
| EDNRB | 9.75679 | 2.254743 | -2.11344 | 1.80E-58 |
| TRPC1 | 2.405424 | 1.029871 | -1.22383 | 1.03E-45 |
| RECK | 8.701436 | 2.73669 | -1.66882 | 5.97E-51 |
| FOXO1 | 17.49902 | 5.198759 | -1.75104 | 3.53E-59 |
| NDNF | 2.825157 | 1.097383 | -1.36426 | 8.22E-37 |
| EDN2 | 0.893741 | 4.718959 | 2.400539 | 1.04E-23 |
| STAT5A | 24.70925 | 9.856285 | -1.32594 | 9.45E-49 |
| COL1A1 | 156.7095 | 738.344 | 2.2362 | 2.93E-38 |
| SLC6A4 | 0.156939 | 2.340951 | 3.898817 | 0.044145 |
| PSME2 | 17.71725 | 41.26326 | 1.219703 | 2.58E-41 |
| HBB | 192.588 | 7.032055 | -4.77543 | 9.04E-52 |
| IL18 | 1.871154 | 4.372457 | 1.224516 | 1.01E-20 |
| SOD3 | 41.60736 | 8.74294 | -2.25065 | 4.37E-48 |
| TMPRSS2 | 7.54001 | 3.260515 | -1.20947 | 5.17E-16 |
| CHEK1 | 1.024708 | 2.876721 | 1.489212 | 2.97E-40 |
| CD24 | 141.5638 | 381.5717 | 1.430502 | 5.46E-14 |
| CCNB1 | 3.832592 | 21.5309 | 2.490016 | 1.63E-60 |
| PTPRB | 7.071262 | 2.117401 | -1.73967 | 1.29E-46 |
| SLC1A3 | 6.817809 | 2.927622 | -1.21958 | 3.21E-19 |
| KCNMB1 | 7.146057 | 1.292363 | -2.46714 | 5.76E-42 |
| NRG1 | 2.553932 | 0.535641 | -2.25338 | 1.78E-32 |
| ACAN | 0.080789 | 1.090096 | 3.754147 | 1.23E-40 |
| NR3C1 | 19.95781 | 6.569678 | -1.60306 | 9.71E-58 |
| COL1A2 | 155.1466 | 468.4966 | 1.594406 | 4.16E-24 |
| KLF2 | 42.2216 | 16.08634 | -1.39215 | 5.56E-33 |
| SLC2A4 | 8.197662 | 0.420616 | -4.28464 | 1.23E-60 |
| ACKR3 | 43.80739 | 13.57829 | -1.68987 | 8.26E-25 |
| H2AX | 7.317854 | 26.23707 | 1.842114 | 1.25E-51 |
| TAL1 | 1.78723 | 0.494767 | -1.8529 | 1.02E-52 |
| MYCN | 0.36937 | 1.226793 | 1.731754 | 0.000508 |
| KIT | 59.00664 | 7.092338 | -3.05654 | 8.44E-45 |
| WT1 | 0.062332 | 1.165924 | 4.225356 | 2.33E-30 |
| INHBA | 1.080225 | 11.2546 | 3.38111 | 2.49E-58 |
| MMP3 | 1.686981 | 7.766624 | 2.202844 | 3.70E-26 |
| SLC16A7 | 4.770912 | 0.536554 | -3.15247 | 9.98E-54 |
| PLA2G4A | 6.435594 | 2.439451 | -1.39952 | 5.81E-51 |
| CGA | 0.029135 | 11.40483 | 8.612671 | 2.82E-18 |
| ANXA1 | 162.76 | 32.89008 | -2.30702 | 1.17E-60 |
| GBE1 | 16.0897 | 6.196791 | -1.37654 | 6.77E-33 |
| CDKN2A | 0.767331 | 5.173855 | 2.753318 | 9.82E-32 |
| TAGLN | 203.9361 | 80.60344 | -1.3392 | 2.48E-38 |
| SOX2 | 0.095262 | 0.974864 | 3.355225 | 0.018444 |
| KCNH2 | 0.213272 | 0.925491 | 2.117524 | 0.000297 |
| CX3CL1 | 66.3438 | 12.0623 | -2.45946 | 1.09E-48 |
| VLDLR | 7.300012 | 2.41352 | -1.59676 | 1.98E-38 |
| ALOX15B | 48.74793 | 19.11933 | -1.35031 | 7.88E-06 |
| IFI27 | 19.65061 | 73.09296 | 1.895158 | 1.19E-08 |
| EZH2 | 1.345085 | 6.316499 | 2.231428 | 1.64E-56 |
| COL3A1 | 261.5722 | 700.9508 | 1.422104 | 8.83E-22 |
| ADAMTS1 | 35.60203 | 8.021159 | -2.15008 | 1.19E-52 |
| MMP13 | 0.173764 | 10.27447 | 5.885787 | 4.34E-56 |
| F12 | 0.511224 | 3.417941 | 2.741099 | 1.41E-45 |
| NR4A3 | 4.844968 | 1.210994 | -2.0003 | 7.20E-22 |
| RGCC | 54.61923 | 19.68176 | -1.47255 | 3.75E-50 |
| IDH2 | 35.1181 | 86.19702 | 1.295423 | 6.87E-38 |
| IL33 | 25.15969 | 4.073117 | -2.62691 | 1.27E-60 |
| MIR27A | 2.726417 | 0.516929 | -2.39897 | 2.56E-40 |
| NTRK2 | 21.00551 | 3.097505 | -2.76159 | 2.84E-59 |
| MME | 30.8313 | 3.274344 | -3.23512 | 1.47E-63 |
| CDC25A | 0.516896 | 2.20605 | 2.09352 | 2.62E-40 |
| PCK1 | 11.52663 | 0.45687 | -4.65704 | 4.10E-46 |
| MST1R | 1.660468 | 4.232292 | 1.349849 | 5.10E-27 |
| KRT18 | 91.16487 | 266.6379 | 1.548332 | 4.50E-32 |
| ADAM8 | 1.122156 | 5.224114 | 2.218913 | 1.30E-45 |
| CFI | 11.00728 | 4.720271 | -1.22152 | 3.04E-43 |
| CLDN3 | 27.13307 | 68.1072 | 1.327755 | 1.29E-15 |
| SELP | 8.217698 | 2.652108 | -1.63159 | 3.96E-40 |
| ATAD2 | 3.560661 | 13.28844 | 1.899955 | 1.38E-43 |
| FGFR3 | 1.411958 | 7.140559 | 2.33834 | 2.07E-18 |
| SCG2 | 0.501336 | 2.239859 | 2.159559 | 1.96E-22 |
| CCL28 | 38.70968 | 6.930657 | -2.48163 | 7.15E-36 |
| SOCS3 | 86.34608 | 25.54118 | -1.75731 | 2.73E-17 |
| CDK1 | 1.47265 | 12.67879 | 3.105931 | 3.07E-60 |
| PTPRZ1 | 1.859166 | 0.611273 | -1.60477 | 4.06E-37 |
| SLC1A2 | 0.707281 | 1.813716 | 1.358594 | 0.026505 |
| NOTCH4 | 6.94817 | 2.602169 | -1.41692 | 5.46E-49 |
| MDK | 34.05502 | 85.60505 | 1.329828 | 3.09E-18 |
| LTA | 0.192672 | 0.693022 | 1.846751 | 5.53E-16 |
| TPM2 | 76.15516 | 29.75724 | -1.3557 | 1.84E-22 |
| TRH | 0.288016 | 13.12596 | 5.51013 | 0.019314 |
| CD248 | 57.78746 | 18.7949 | -1.62042 | 5.28E-27 |
| DRD4 | 0.220709 | 0.599099 | 1.440649 | 4.40E-17 |
| CFH | 16.24081 | 6.408079 | -1.34166 | 2.33E-28 |
| TP63 | 13.306 | 2.095856 | -2.66647 | 8.92E-39 |
| APOA1 | 0.060992 | 0.810062 | 3.731343 | 6.44E-09 |
| HLF | 9.524466 | 1.360249 | -2.80777 | 6.29E-61 |
| TMPRSS6 | 0.440592 | 2.12648 | 2.270951 | 1.39E-07 |
| CA3 | 45.53185 | 1.621731 | -4.81127 | 3.72E-55 |
| KLF6 | 80.31235 | 32.44929 | -1.30744 | 1.58E-38 |
| BBC3 | 1.703061 | 4.228905 | 1.312154 | 4.80E-28 |
| ATF3 | 47.6906 | 11.46394 | -2.0566 | 1.29E-37 |
| TFF1 | 60.30045 | 378.9876 | 2.65191 | 9.79E-08 |
| FGF7 | 4.864147 | 1.519639 | -1.67846 | 2.22E-45 |
| NGFR | 15.2902 | 3.839258 | -1.99371 | 6.41E-40 |
| RAMP1 | 2.314814 | 16.67279 | 2.848527 | 1.35E-35 |
| TNFRSF10D | 3.339168 | 1.030948 | -1.69552 | 9.00E-52 |
| EFNA3 | 2.6712 | 9.164875 | 1.778627 | 3.51E-24 |
| CCL20 | 0.217197 | 1.15222 | 2.407337 | 8.89E-13 |
| KLF15 | 7.139233 | 0.778424 | -3.19714 | 8.79E-59 |
| ESM1 | 0.579625 | 4.161456 | 2.843897 | 1.44E-54 |
| UCN | 0.571613 | 1.550135 | 1.439283 | 6.85E-19 |
| STMN1 | 10.79562 | 35.93135 | 1.734798 | 6.45E-45 |
| FOXP3 | 0.589074 | 2.531126 | 2.103258 | 2.60E-41 |
| SCD | 376.3801 | 141.0466 | -1.41602 | 1.53E-05 |
| MMP12 | 0.766134 | 3.868672 | 2.336169 | 7.59E-06 |
| CDH2 | 0.499243 | 2.710854 | 2.440933 | 2.06E-23 |
| CDK5 | 2.779808 | 7.151413 | 1.363243 | 3.90E-53 |
| EZR | 56.70529 | 131.6327 | 1.214963 | 3.46E-42 |
| TREM1 | 0.345798 | 0.811823 | 1.231234 | 6.78E-13 |
| FABP4 | 1072.007 | 42.63598 | -4.6521 | 2.49E-56 |
| ADRB2 | 5.299286 | 0.827478 | -2.67901 | 1.73E-61 |
| PYY | 0.171719 | 1.023424 | 2.575285 | 5.34E-07 |
| LOXL1 | 6.257398 | 16.9581 | 1.43834 | 2.57E-31 |
| MEG3 | 4.085517 | 1.260195 | -1.69687 | 1.08E-43 |
| TGFBR3 | 33.57688 | 6.292739 | -2.41571 | 1.43E-58 |
| CRYAB | 167.3824 | 26.75508 | -2.64526 | 2.87E-53 |
| SELL | 2.837663 | 8.655001 | 1.608831 | 4.58E-11 |
| SLC16A3 | 1.089484 | 5.194698 | 2.253395 | 7.94E-48 |
| PLAGL1 | 5.195688 | 1.663738 | -1.64289 | 2.26E-51 |
| H4-16 | 0.571965 | 1.589678 | 1.474736 | 2.71E-08 |
| TGFBR2 | 101.4994 | 23.58872 | -2.1053 | 1.14E-59 |
| DDR2 | 27.51152 | 4.84332 | -2.50597 | 3.17E-52 |
| NEDD9 | 15.54228 | 6.273842 | -1.30878 | 8.12E-39 |
| CXCL9 | 4.703473 | 44.14629 | 3.230494 | 5.84E-25 |
| RET | 1.479645 | 11.15175 | 2.913947 | 7.67E-18 |
| HMGA1 | 22.76462 | 64.10094 | 1.493553 | 9.68E-37 |
| PTK6 | 2.098485 | 7.781497 | 1.8907 | 1.43E-34 |
| SLC19A3 | 25.15408 | 0.953567 | -4.72131 | 1.38E-50 |
| SPRY2 | 22.3502 | 4.120036 | -2.43956 | 4.38E-65 |
| C19orf33 | 4.798215 | 30.07585 | 2.648036 | 5.62E-11 |
| FLT3 | 0.776588 | 1.798457 | 1.211538 | 0.003616 |
| SPINT2 | 46.04835 | 112.0328 | 1.2827 | 1.78E-40 |
| PDGFA | 11.03069 | 4.367079 | -1.33678 | 4.88E-28 |
| IL20 | 0.345543 | 3.659439 | 3.404685 | 2.87E-11 |
| ADM2 | 0.923161 | 3.865178 | 2.06588 | 2.45E-41 |
| CCN3 | 9.244314 | 3.396907 | -1.44434 | 1.92E-35 |
| CDC6 | 0.837946 | 6.241191 | 2.896892 | 3.27E-52 |
| AKAP12 | 23.89805 | 4.187265 | -2.51281 | 9.51E-51 |
| PVT1 | 1.526984 | 3.859341 | 1.337669 | 1.89E-24 |
| GPX3 | 266.6348 | 23.5647 | -3.50017 | 1.82E-49 |
| BCL9 | 4.809214 | 11.2859 | 1.230649 | 1.21E-43 |
| PDCD1 | 0.318735 | 1.121473 | 1.814967 | 9.29E-14 |
| RGS4 | 0.869553 | 2.837722 | 1.706387 | 1.19E-23 |
| MYL2 | 56.12067 | 0.537885 | -6.70509 | 8.70E-25 |
| LAMP3 | 1.129996 | 4.227649 | 1.903538 | 5.55E-10 |
| NRN1 | 25.3793 | 5.659967 | -2.16479 | 1.80E-53 |
| UNC5B | 3.182112 | 12.24662 | 1.944327 | 7.78E-51 |
| SPAG4 | 0.915029 | 2.54702 | 1.476921 | 3.03E-26 |
| CD209 | 5.291384 | 0.879467 | -2.58894 | 1.40E-38 |
| MX1 | 7.882447 | 26.47522 | 1.747927 | 1.98E-12 |
| EDN3 | 5.316516 | 0.89194 | -2.57546 | 3.40E-37 |
| AGR2 | 45.52992 | 223.9668 | 2.298398 | 2.20E-16 |
| PLTP | 67.72637 | 27.76543 | -1.28643 | 1.28E-35 |
| SLC11A1 | 0.346348 | 0.905557 | 1.386583 | 2.61E-27 |
| CEACAM5 | 0.844929 | 15.09606 | 4.159198 | 1.62E-21 |
| INHA | 0.297783 | 1.877195 | 2.656244 | 7.41E-08 |
| BRCA2 | 0.380192 | 1.05557 | 1.473223 | 8.17E-34 |
| DUSP6 | 35.80463 | 12.99382 | -1.46232 | 2.22E-49 |
| TDO2 | 0.070918 | 0.888822 | 3.647664 | 2.38E-52 |
| RND3 | 35.72938 | 13.48069 | -1.40622 | 6.56E-45 |
| CXCL2 | 19.20027 | 1.787376 | -3.42521 | 2.05E-59 |
| ABCC9 | 4.73629 | 1.083639 | -2.12787 | 1.41E-37 |
| LAMA3 | 14.90229 | 4.096706 | -1.863 | 1.11E-40 |
| PER1 | 24.21672 | 5.784971 | -2.06562 | 1.35E-51 |
| CANT1 | 17.02797 | 40.18494 | 1.238749 | 1.78E-39 |
| RRAD | 3.585968 | 1.395357 | -1.36173 | 8.48E-27 |
| MCAM | 62.52723 | 15.16256 | -2.04397 | 1.66E-46 |
| BMX | 2.867742 | 0.476941 | -2.58803 | 1.34E-58 |
| TFR2 | 0.086965 | 0.796645 | 3.195428 | 2.66E-41 |
| FBLN5 | 36.36915 | 11.03137 | -1.7211 | 7.56E-53 |
| PDGFRA | 13.5432 | 5.301999 | -1.35296 | 2.27E-47 |
| SOCS2 | 6.864517 | 2.969146 | -1.20911 | 6.49E-36 |
| ZEB2 | 4.948921 | 1.874788 | -1.40039 | 4.87E-33 |
| ANKDD1A | 2.10684 | 0.819326 | -1.36257 | 4.73E-46 |

Supplementary table 3. Nine prognosis-related hypoxia genes in the training set.

| id | coef | HR | HR.95L | HR.95H | pvalue |
| --- | --- | --- | --- | --- | --- |
| ALOX15B | -0.227238557 | 0.796730694 | 0.653883544 | 0.970784177 | 0.02419067 |
| CA9 | -0.136731415 | 0.872204456 | 0.724259513 | 1.050370204 | 0.149365813 |
| CD24 | 0.123720268 | 1.131699255 | 0.957752607 | 1.337238024 | 0.146220675 |
| CHEK1 | 0.682787484 | 1.97938756 | 1.156919591 | 3.38655784 | 0.012704685 |
| FOXM1 | -0.331436333 | 0.71789186 | 0.513195245 | 1.004235187 | 0.05295514 |
| HOTAIR | 0.26036428 | 1.297402618 | 1.027622718 | 1.638007338 | 0.028592699 |
| KCNJ11 | -0.235613199 | 0.790086221 | 0.617135708 | 1.011505621 | 0.061594056 |
| NEDD9 | -0.539122538 | 0.583259817 | 0.403472888 | 0.843159536 | 0.004140135 |
| PSME2 | -0.386823763 | 0.679210786 | 0.465576501 | 0.990873232 | 0.044691367 |


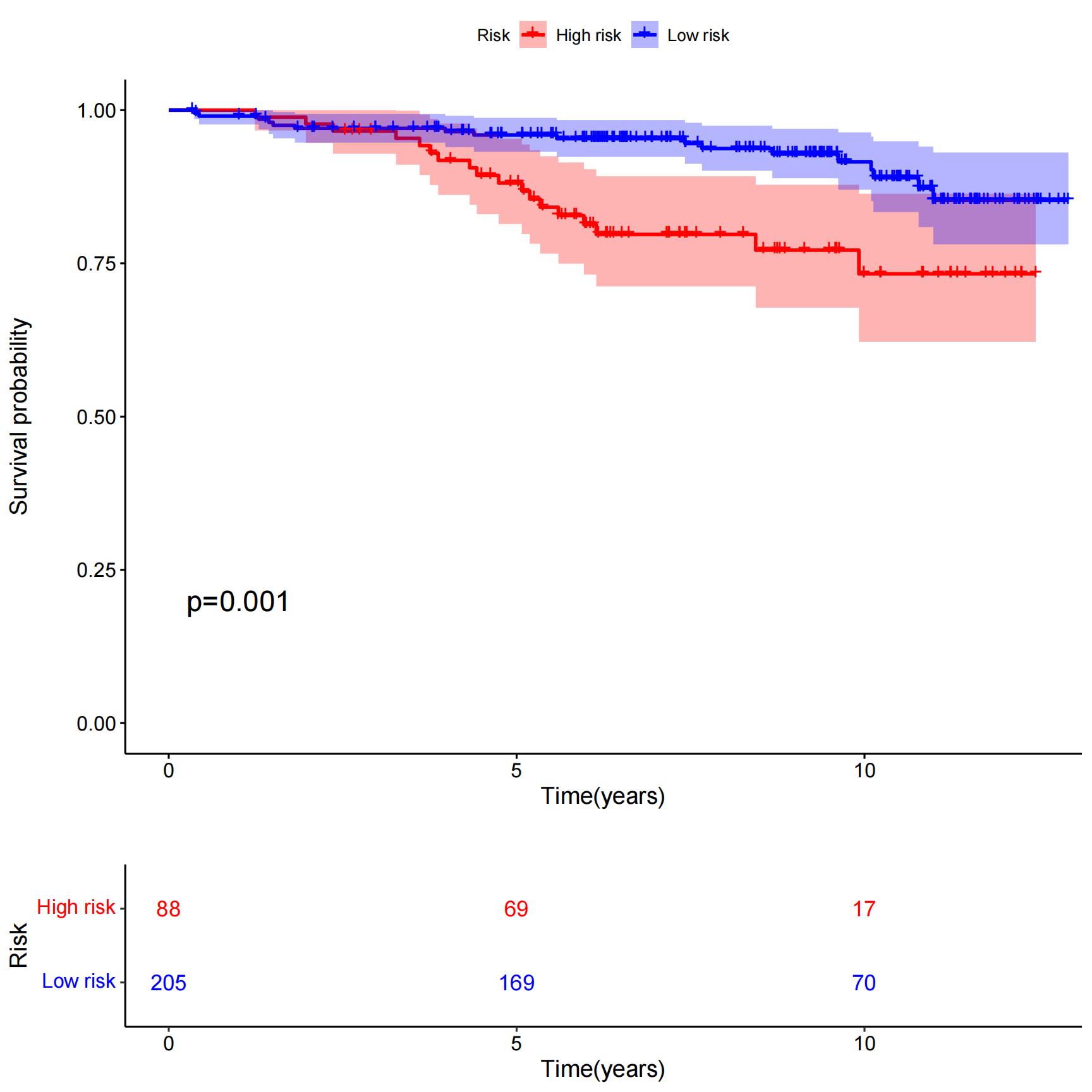


Supplementary figure 1. The signature was verified in GSE131769 with P=0.001


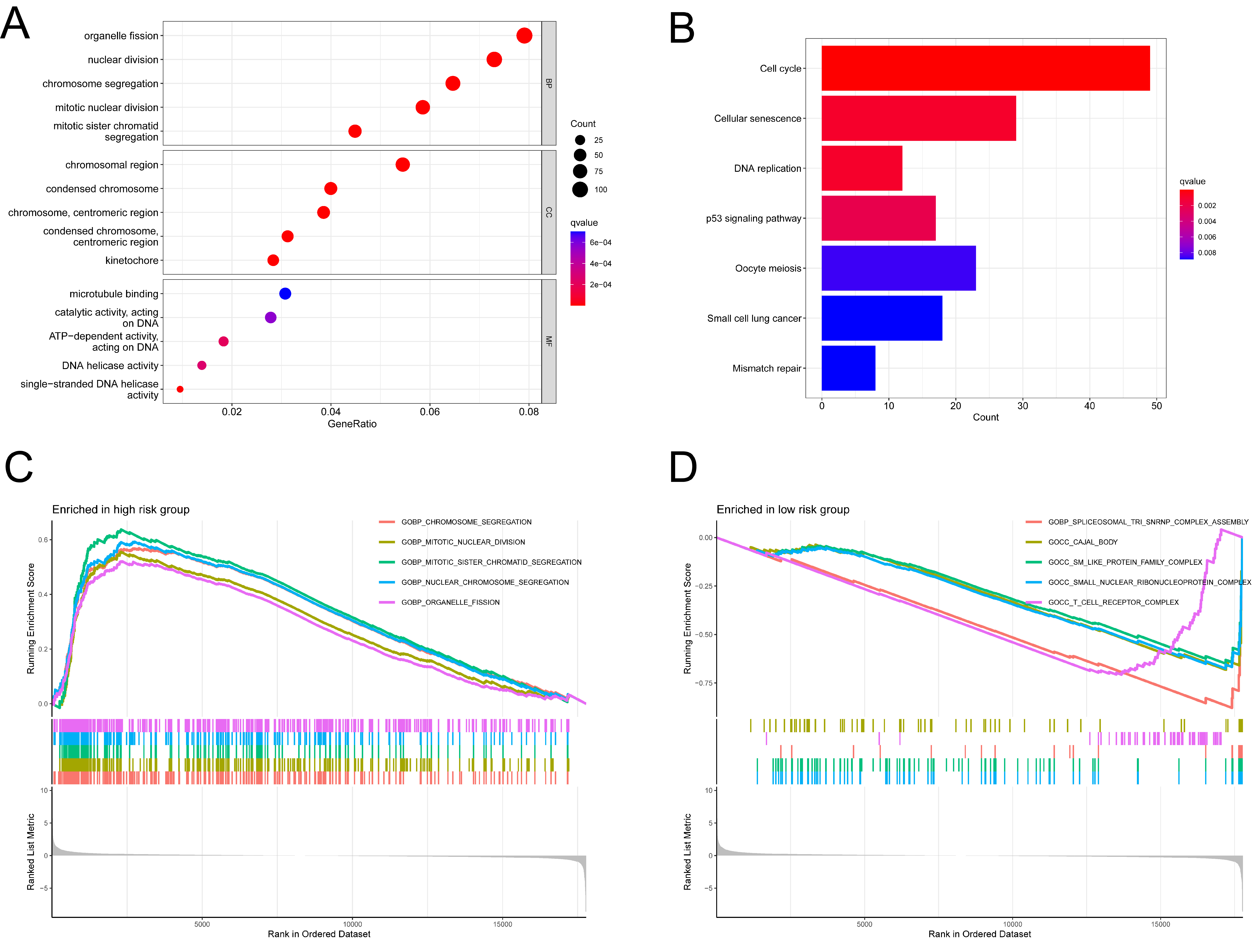


Supplementary figure 2. Functional enrichment analysis. (A.GO analysis, B.KEGG pathway analysis, C. GSEA analysis in high-risk group, D. GSEA analysis in low-risk group)
